# EDA Complex‐Driven Desaturation of Heterocyclic Carbonyl Compounds Enabled by HFIP

**DOI:** 10.1002/anie.202514539

**Published:** 2025-10-29

**Authors:** Rakesh Maiti, Robin Cauwenbergh, Aritra Nath, Ana B. R. Guimarães, Yuman Qin, Feliu Maseras, Shoubhik Das

**Affiliations:** ^1^ University of Bayreuth Universitätstr. 30 95447 Bayreuth Germany; ^2^ University of Antwerp University of Antwerp Antwerp 2020 Belgium; ^3^ Institute of Chemical Research of Catalonia (ICIQ‐CERCA) The Barcelona Institute of Science and Technology Avgda, Països Catalans Tarragona 16–43007 Spain

**Keywords:** Dehydrogenation, EDA‐complex, Heterocyclic carbonyls, DFT, Sustainable protocol

## Abstract

Recently, electron donor–acceptor (EDA) complex‐mediated organic synthetic strategies have emerged as powerful tools for diverse bond‐forming transformations; however, their efficiency often diminishes when ionic reactants are involved. This limitation arises from the requirement of polar solvents such as DMSO or DMF to solubilize ionic species for the formation of effective EDA complex. Consequently, these solvents engage in competing EDA complex formation or disrupt ionization equilibria. In parallel, there is a pressing necessity of modern and efficient strategy to achieve dehydrogenation reactions, which are in general limited by the drawbacks of traditional approaches. To address both, herein, we disclose an innovative desaturation strategy based on the formation of an EDA complex between a dihydrogenated organic substrate and an N‐methoxy pyridinium salt. In our study, solubility issues, which are associated with the pyridinium salt, are effectively addressed by using hexafluoroisopropanol (HFIP). Beyond enhancing solubility, HFIP also functions as a transient H‐shuttle, significantly reducing the activation energy for this transformation. This cooperative interplay between HFIP and the pyridinium salt enables the efficient and selective desaturation of a broad range of heterocyclic carbonyl compounds—including quinolinones, coumarins, and flavones—which are valuable scaffolds in pharmaceutical and agrochemical research. At the end, detailed mechanistic studies with the aid of experiments as well as DFT studies clearly disclose the mechanism as well as the important role of HFIP in this reaction.

## Graphical abstract

In this work, we report a novel light‐mediated desaturation strategy enabled by electron donor–acceptor complex formation. DFT and mechanistic studies reveal that the reaction proceeds via single‐electron transfer, hydrogen atom transfer, and base‐mediated deprotonation sequentially with pyridinium salt serving dually as oxidant and base, and hexafluoroisopropanol functioning as solvent and radical mediator. Notably, this strategy demonstrates broad substrate scope, enabling efficient synthesis of quinolinones, coumarins, and flavones, and also facilitates the late‐stage functionalization of drug molecules.

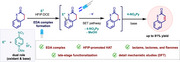

EDA complex‐mediated organic synthesis has emerged as a powerful strategy that harnesses the ground‐state association between an electron acceptor (A) and a donor (D) to generate reactive radical species upon the irradiation of visible light (Scheme 1a).^[^ ^1^, ^2^, ^3^, ^4^ ^]^ This methodology has attracted significant interest due to its operational simplicity, ability to activate inert substrates, and avoids the necessity of exogenous photocatalysts.^[^ ^2^, ^5^ ^]^ As a result, extensive efforts have been directed for various bond‐forming reactions through the formation of an EDA complex.^[^ ^6^, ^7^, ^8^, ^9^, ^10^, ^11^, ^12^, ^13^, ^14^, ^15^, ^16^, ^17^, ^18^, ^19^, ^20^ ^]^ However, a key limitation lies in the solubility of the components, especially when ionic reagents are involved in the process.^[^ ^2^, ^21^, ^22^ ^]^ Although ionic species are well‐suited for EDA complex formation due to their strong aggregation tendencies, they typically require polar solvents. (e.g., DMSO, DMF) for dissolution.^[^ ^10^, ^12^, ^23^, ^24^, ^25^ ^]^ In consequence, these solvents engage into competing EDA interactions or interfere with the ionization equilibrium, which ultimately lowers the efficiency of the corresponding reaction.^[^ ^23^, ^24^, ^25^ ^]^ To address this, recent strategies employ HFIP as a solvent, whose strong hydrogen‐bonding ability (pK_a_ = 9.3) enhances the solubility of ionic species and stabilizes the overall reaction environment.^[^ ^26^, ^27^ ^]^ Additionally, HFIP's low nucleophilicity (negative inductive effect of F‐atoms), redox stability (stable C─F bond), and capacity for cation stabilization (high dielectric constant: *ε* = 15.7) facilitate efficient EDA‐mediated radical processes.^[^ ^28^, ^29^ ^]^ Moreover, HFIP's intrinsic tendency to form small, dynamic H‐bonded clusters with reagents increases the local concentration of reactive species, thereby enhancing the reaction rate.^[^ ^30^, ^31^, ^32^ ^]^ Simultaneously, this compact microenvironment facilitates rapid intracluster H‐exchange, making HFIP an exceptional medium for H‐shuttling.^[^ ^27^ ^]^ As a result, the overall activation energy of a reaction is significantly lowered by using HFIP.^[^ ^30^, ^31^, ^32^ ^]^

**Scheme 1:**
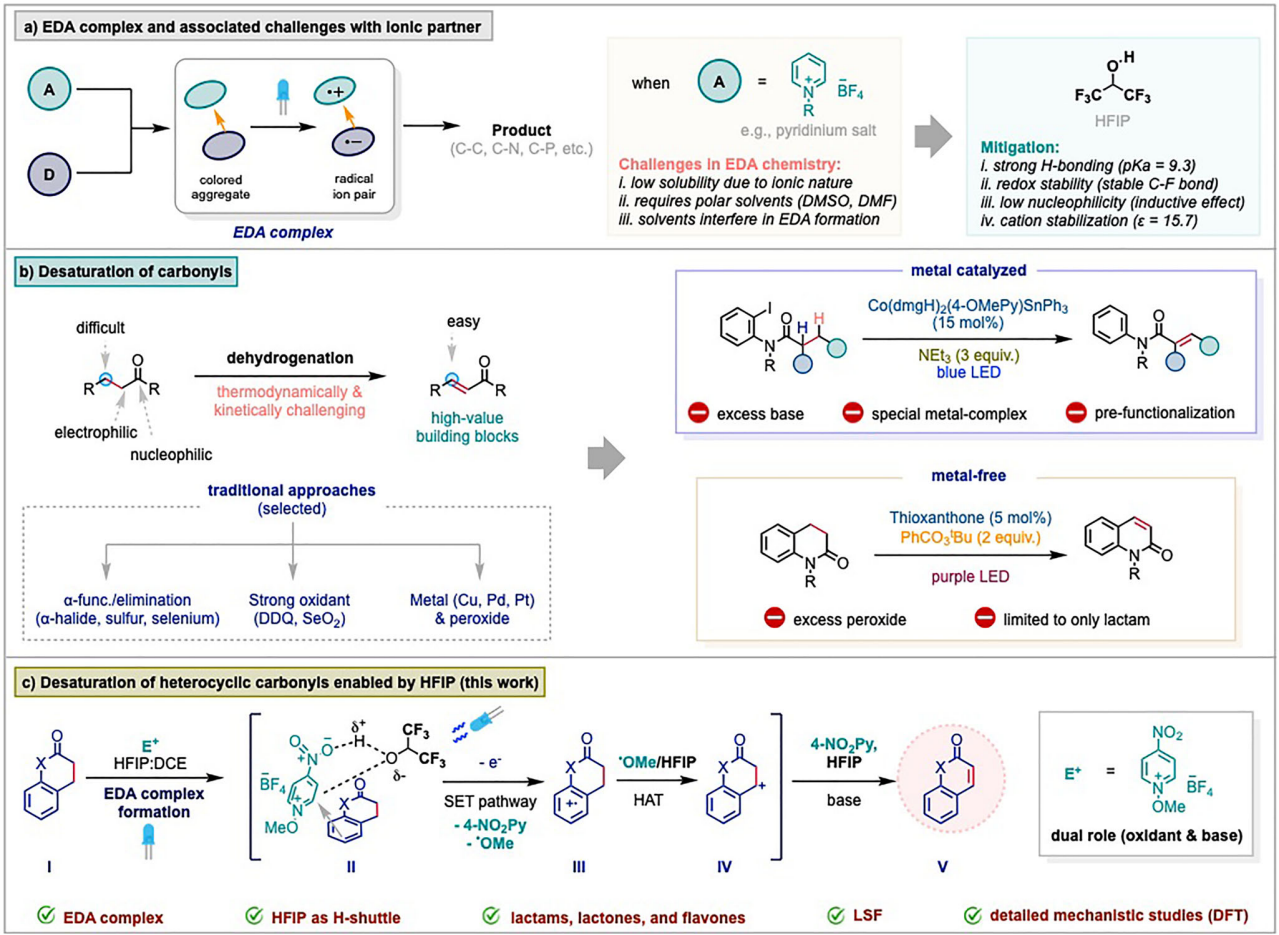
EDA complex chemistry and desaturation of carbonyls

Desaturation of carbonyl compounds is a fundamental transformation in organic synthesis, involving the removal of two hydrogen atoms from a carbon adjacent to a carbonyl group (Scheme 1b).^[^ ^33^, ^34^, ^35^, ^36^ ^]^ Despite its apparent simplicity, this transformation possesses significant thermodynamic and kinetic challenges, owing to the high reactivity and potential energy of the resulting unsaturated products.^[^ ^34^ ^]^ Consequently, there is a continued interest in developing improved and sustainable methods to overcome the limitations of traditional protocols where the use of strong oxidants,^[^ ^37^, ^38^ ^]^ metals,^[^ ^39^, ^40^, ^41^, ^42^, ^43^, ^44^ ^]^ and toxic organo‐selenium or organosulfur reagents is essential.^[^ ^45^, ^46^, ^47^ ^]^ In this regard, radical‐mediated strategies^[^ ^48^ ^]^—particularly photoredox catalysis and electrocatalysis—have emerged as attractive alternatives. However, the developed methods still possess inherent limitations (Scheme 1b). For example, electrocatalytic methods often require pre‐functionalized substrates (e.g., TMS derivatives of carbonyls).^[^ ^33^ ^]^ On the other hand, photoredox strategies often exhibit low selectivity due to competing side reactions involving acidic α‐hydrogens, resulting in reduced yields of the desired product.^[^ ^49^ ^]^ This challenge has led to the development of only two photoredox‐based desaturation protocols so far: a) a specially designed photoactive cobalt complex, [Co(dmgH)_2_(4‐OMePy)SnPh_3_], combined with excess Et_3_N to enable a selective HAT‐process;^[^ ^49^ ^]^ and b) a thioxanthone‐catalyzed approach, uses an excessive amount of tert‐butyl perbenzoate (PhCO_3_^t^Bu), however, is limited to lactams (Scheme 1b).^[^ ^50^ ^]^

Considering all these facts, herein, we report an EDA‐based desaturation strategy, which has been promoted by HFIP (Scheme 1c). In this system, the EDA complex undergoes HAT‐mediated formation of carbocation **IV**, followed by a base‐induced E1 elimination, circumventing the direct abstraction of two adjacent hydrogen atoms, which typically demands a higher activation energy.^[^ ^49^, ^50^ ^]^ This method accommodates a broad substrate scope, including quinolinones, coumarins, and flavones, without the need for any stoichiometric external oxidant or reductant.^[^ ^49^ ^]^ Central to this concept is the use of a quaternary *N*‐methoxy pyridinium (NAP) salt which plays a dual role: initiating the formation of an EDA complex with the heterocyclic carbonyl substrate and enabling the subsequent E1 elimination.^[^ ^2^, ^20^, ^21^, ^22^ ^]^ To the best of our knowledge, this work provides a unique entry into dehydrogenation chemistry, particularly under the influence of HFIP as the solvent. Owing to its high polarity, HFIP effectively overcomes the solubility issues commonly encountered with the ionic pyridinium salts in EDA‐mediated organic reactions.^[^ ^26^, ^51^ ^]^ In addition, the strong H‐bonding ability of HFIP stabilizes the radical cationic intermediate **III** through H‐bond interactions.^[^ ^52^ ^]^ Beyond HFIP's role as solvent, this work reveals for the first time that the weak C–H bond in HFIP (BDE = 97.6 kcal mol^−1^) can serve as a transient H‐shuttling platform between the methoxy radical and the substrate, thereby facilitating the HAT step (**III → IV**) and lowering the overall energy barrier of the desaturation process (as confirmed by DFT calculations). Overall, this HFIP‐promoted EDA complex‐mediated approach enables selective and efficient desaturation of heterocyclic carbonyl compounds and expands the synthetic toolbox for accessing complex, biologically relevant frameworks such as nybomycin (an antibiotic), methoxsalen^[^ ^53^ ^]^ (for eczema treatment) and wogonin^[^ ^54^ ^]^ (an anticonvulsant).

At the outset of the project, **1a** and **2a** were chosen as model substrates for the formation of an EDA complex and to optimize the reaction conditions (Table 1). Guided by our mechanistic hypothesis, the reaction was initially performed in HFIP as the solvent. To our delight, stirring a mixture of **1a** and **2a** in HFIP under the irradiation of 390 nm light at 45 °C afforded the dehydrogenated product **3a** in 50% yield (Table 1 **, entry 1**). To confirm the unique role of HFIP in promoting this transformation through enhanced solubility and stabilization of radicals/transition states via H‐bonding,^[^ ^55^ ^]^ we examined highly polar solvents such as DMSO, DMA, and DMF. These solvents, however, yielded only trace amounts of product (Table 1 **, entry 2**), likely due to their potential to directly engage in EDA complex formation and interfere with the ionization of **2a**.^[^ ^23^, ^24^, ^25^ ^]^ To improve the yield further, we explored solvent mixtures incorporating HFIP and chlorinated solvents. This is because chlorinated solvents are considered to be beneficial due to their radical inertness (attributed to strong C–Cl bonds).^[^ ^56^ ^]^ Additionally, we hypothesize that the excess amount of HFIP could unfavorably coordinate with **2a**, and thereby, can disrupt the formation of intermediate **II**. In this context, the chlorinated solvent was envisioned to modulate the HFIP environment, promoting the formation of smaller and more mobile H‐bonded clusters, thus enhancing the efficiency for the formation of the EDA complex.^[^ ^55^ ^]^ This rationale led to the use of a DCE/HFIP mixture, which gratifyingly increased the yield of **3a** to 58% (Table 1 **, entry 3**). Motivated by this result, we next examined a series of pyridinium salts under the optimized conditions to obtain improved yield. Notably, replacing the ethoxy group with a methoxy group improved the yield to 64% (Table 1 **, entry 4**). In contrast, modifying the ─CN substituent on the pyridinium ring with less electron‐withdrawing groups (e.g., ─Cl, ─F, ─OAc) or switching to isoquinoline‐based analogs resulted in drastically diminished yields (0%–19%; Table 1 **, entry 5**). Remarkably, incorporation of a strongly electron‐withdrawing nitro group provided the highest yield of 71% (Table 1 **, entry 6**). Additionally, various other parameters were systematically screened, including the amount of pyridinium salt, the DCE/HFIP ratio, and reaction concentration. Nevertheless, the conditions described in Table 1 **, entry 6** emerged as the optimal set for this transformation (for a comprehensive overview, please see Supporting Information; Section 3: Optimization Studies). Once pyridinium salt **2h** was established as the most effective for the reaction, we further evaluated the role of HFIP by substituting it with another fluorinated solvent, i.e., trifluoroethanol (TFE), using **2h** as the acceptor in EDA complex formation (Table 1 **, entry 7**). Under these conditions, the yield of the desired product **3a** decreased to 26%, demonstrating the key influence of HFIP in the process. Subsequently, to provide evidence that polar solvents such as DMSO, DMF, and DMA impede efficient EDA complex formation, mixtures of each with HFIP were tested. As anticipated, these trials resulted in uniformly lower yields (Table 1 **, entries 8–10**), supporting our hypothesis regarding the detrimental impact of these polar solvents on the outcome. Encouraged by these findings, a series of control experiments was further carried out under optimized conditions to validate the essential role of each of the reaction components. Lowering the reaction temperature to approximately 25 °C by using a fan led to a reduced yield of 53% (Table 1 **, entry 11**), indicating that the elevated temperature enhanced the reaction efficiency. This could be rationalized from the fact that elevated temperature accelerated the base‐promoted E1 elimination, which was the rate‐determining step in this reaction (see mechanistic part for details).^[^ ^57^ ^]^ Notably, replacing the light source to lower wavelengths (*λ*_max_ = 456 nm or 522 nm), conducting the reaction in the dark, or at 60 °C (Table 1, **entry 12**), did not lead to any detectable product formation. These results clearly indicate that effective excitation of the EDA complex occurs near 390 nm, as confirmed by UV–vis spectroscopy and DFT calculations. Furthermore, the reaction is not thermally driven as no product formation was observed under dark conditions at 60 °C. In addition, no product was found in the absence of the pyridinium salt, which clearly demonstrated the key role of this reagent in this dehydrogenation reaction (Table 1, **entry 13**).

**Table 1:** Optimization of the reaction conditions.^a)^

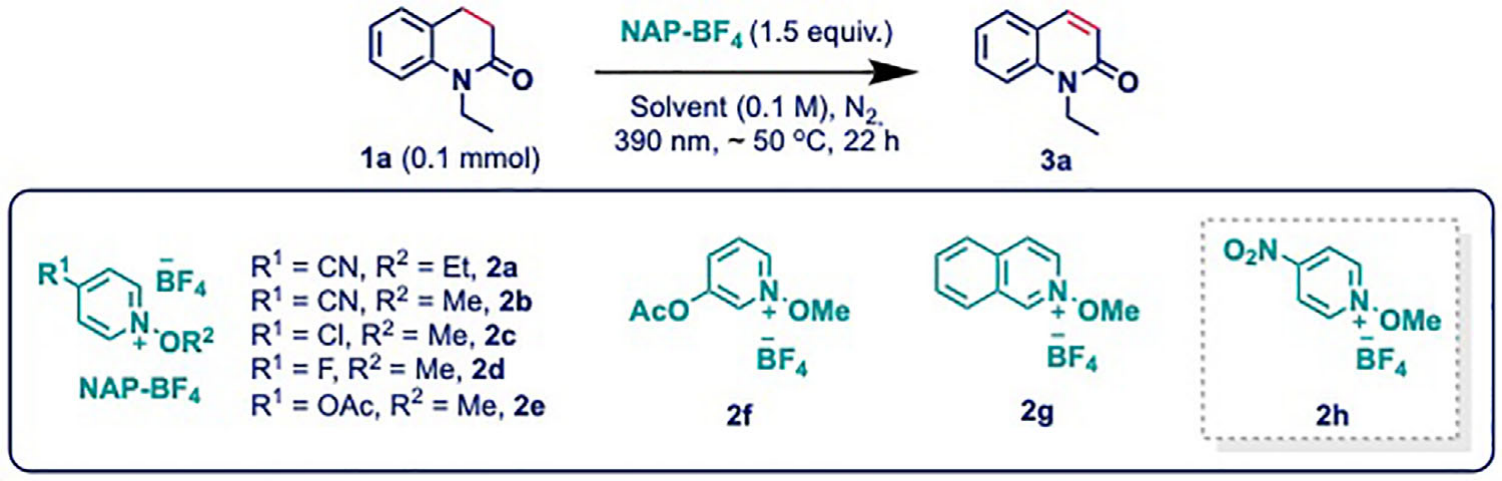			
Entry	Salt	Reaction conditions	Yield (%)
1	2a	HFIP	50
2	2a	DMSO, DMA or DMF	trace
3	2a	DCE:HFIP (7:3)	58
4	2a	DCE:HFIP (7:3)	64
5	2c‐2g	DCE:HFIP (7:3)	0–19
**6**	**2h**	**DCE:HFIP (7:3)**	**71**
7	2h	TFE	26
8	2h	DMSO:HFIP (7:3)	8
9	2h	DMF:HFIP (7:3)	3
10	2h	DMA:HFIP (7:3)	3
11^b)^	2h	cooling to ± 25 oC	53
12^b)^	2h	*λ*_max_ = 456 nm or *λ*_max_ = 522 nm or in dark or 60 °C	n.d.
13	–	DCE: HFIP (7:3)	n.d.

^a)^ Reaction conditions: **1** (0.1 mmol, 1 equiv.), **2** (1.5 equiv.). Yield was determined by ^1^H NMR using 1,3,5‐trimethoxybenzene as the internal standard.

^b)^ DCE:HFIP (7:3) was used as the solvent. n.d. = not detected.

With the optimized reaction conditions in our hand, the substrate scope of this dehydrogenation reaction was explored to evaluate its generality (Scheme 2). A broad array of 3,4‐dihydroquinolinones were successfully converted to the corresponding quinolinones. Initially, various *N*‐aryl substituted 3,4‐dihydroquinolinones were examined (**3b–3h**) and the reaction exhibited minimal sensitivity to electronic or positional effects, with both electron‐donating (–OMe, –Me) and electron‐withdrawing (–F, –CN) groups yielding the desired products (**3b–3f**) in 52%–75% yields. To our delight, the free amide variant was compatible upon slight modification of the reaction conditions, specifically by adding LiCl. Herein, LiCl coordinated with the free amide and assisted in effective EDA‐complex formation. Substrates with free amides bearing different substitution patterns also provided the desired quinolinones (**3i–3l**) with consistent yield (55%–67% yield). Additionally, a substrate bearing a substituent at the *α*‐position afforded **3m** in 38% yield, demonstrating the method's tolerance for steric hindrance. Importantly, halogenated substrates (**3g, 3h, 3j**) were well accommodated, providing valuable synthetic handles for further derivatization.

**Scheme 2:**
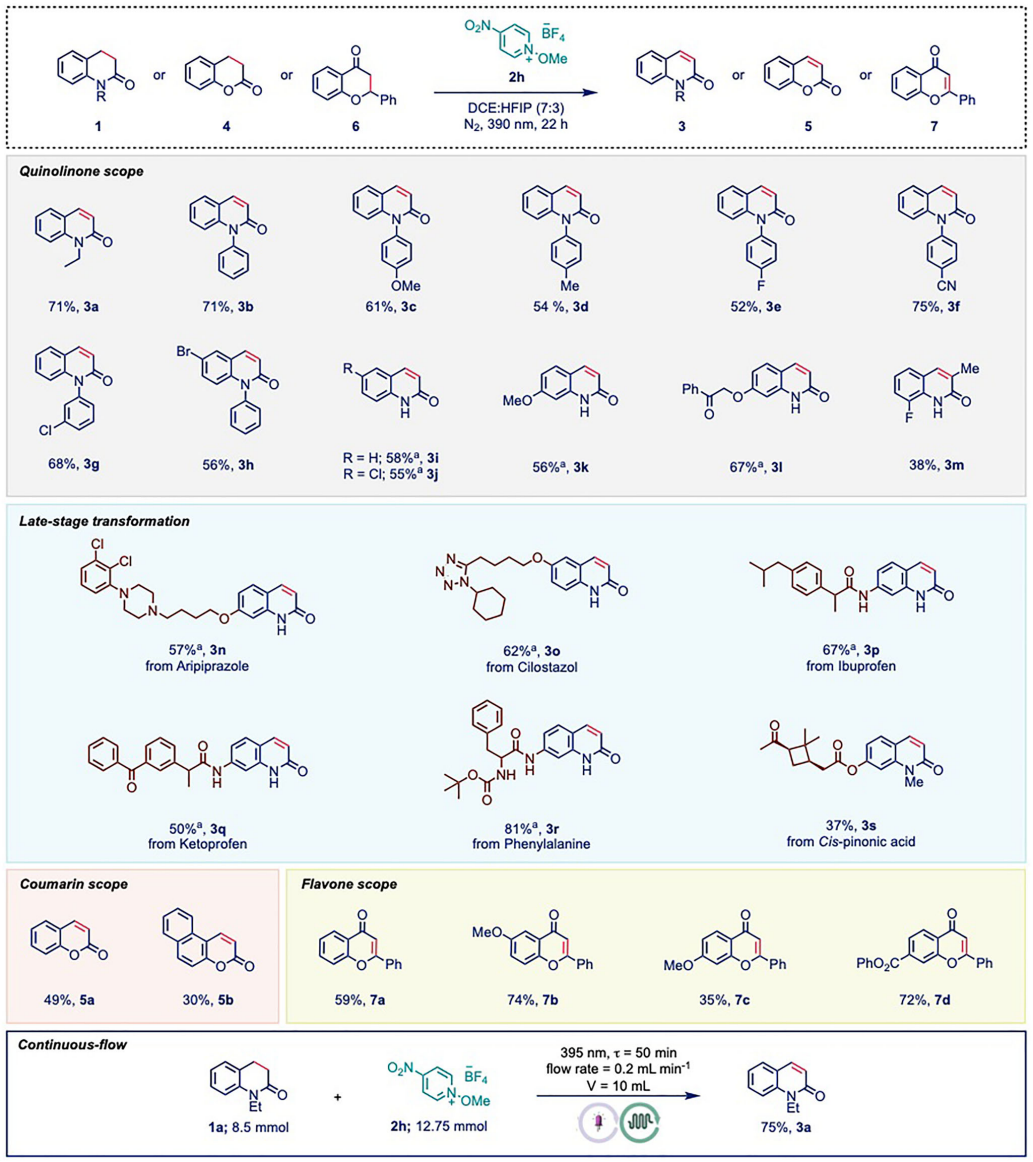
Substrate scope for the EDA‐promoted desaturation reactions. Reaction conditions: 1 or 4 or 6 (0.1 mmol), 2c (1.5 equiv.), DCE:HFIP (0.1 M, 7:3), under nitrogen atmosphere and 40 W 390 nm Kessil lamp irradiation for 22 h. Yields were determined by 1H NMR using 1,3,5‐trimethoxybenzene as the internal standard. aLiCl (1 equiv.) was added.

Given the significance of quinolinone scaffolds in biologically active molecules, we pursued late‐stage transformation (LST) of structurally complex, pharmaceutically relevant derivatives.^[^ ^54^, ^58^, ^59^, ^60^, ^61^, ^62^, ^63^, ^64^ ^]^ This strategy is advantageous for structure–activity relationship (SAR) development and optimization of physicochemical properties such as solubility and stability.^[^ ^62^, ^63^ ^]^ Considering these advantages, aripiprazole and cilostazol were applied under our optimized reaction conditions, and to our delight, they underwent smooth transformation to provide **3n** and **3o** in 57% and 62% yields, respectively. Similarly, ibuprofen and ketoprofen derivatives afforded **3p** (67%) and **3q** (50%) in good yields. Furthermore, substrates derived from D‐phenylalanine and cis‐pinonic acid furnished the corresponding quinolinones **3r** and **3s** in 81% and 37% yields, respectively. These results highlighted the method's versatility and potential utility in medicinal chemistry. Encouraged by these results, we extended the scope beyond dihydroquinolinones to evaluate the broader applicability of our method across other heterocyclic frameworks. Notably, 3,4‐dihydrocoumarins and 4‐chromanones underwent efficient dehydrogenation to furnish the corresponding coumarins (**5a–5b**) and flavones (**7a–7d**) in moderate to excellent yields. These results underscored the versatility of the developed protocol and its potential as a general approach for accessing a wide range of unsaturated heteroaromatic carbonyl scaffolds. Furthermore, to demonstrate the practical applicability of our method, we scaled up the reaction in a Vapourtec UV‐150 photochemical flow reactor (10 mL cartridge) under 395 nm irradiation. On an 8.5 mmol scale, the reaction afforded the dehydrogenated product 3a in 75% yield after 62 h (residence time: 50 min).

Reaction conditions: **1**

After demonstrating the robustness of our catalytic system, we turned our attention to elucidate the underlying mechanism of the dehydrogenation reaction. To probe the involvement of radical intermediates, radical‐quenching experiment was performed using TEMPO (Scheme 3a). The addition of 1 equiv of TEMPO led to a significant decrease in product yield from 71% to 28%, and complete suppression of product formation was observed upon increasing the amount to 2 equiv. Furthermore, to investigate the key step in the transformation following excitation of the EDA complex, which generally represents the highest energy barrier in EDA‐mediated reactions (confirmed computationally; Scheme 4),^[^ ^1^, ^2^ ^]^ a series of kinetic isotope effect (KIE) studies was conducted. The benzylic dideuterated substrate afforded product **3i‐D** in 59% yield, with a KIE of 0.98, indicating that benzylic C–H abstraction does not play a significant role in governing chemo‐selectivity and reaction efficiency under the current conditions (Scheme 3b). In contrast, KIE experiments using α‐CH nondeuterated, monodeuterated (C3‐D), and dideuterated (C3‐D_2_) substrates under identical conditions (Scheme 3c) showed that while the first two gave 71% yield, the dideuterated substrate afforded only 14% yield. The observed KIE (K_H_/K_D_ = 5.07) supports an E1‐type elimination mechanism, identifying base‐assisted α‐C–H cleavage as the key step to afford the desired product. To further validate our findings, we repeated the above reaction and collected data at an intermediate time point (15 h) to obtain more accurate measurements. In this experiment, a similar KIE of 4.22 (K_H_/K_D_) was observed, further supporting that the E1‐type elimination step remains the key step throughout the reaction. Additionally, methanol was detected as a by‐product, suggesting the involvement of a methoxy radical in the HAT step (Scheme 3c). Next, we examined the kinetics of the desaturation reaction and found that the reaction was completed within 22 h under the irradiation of 390 nm Kessil lamp. Subsequently, the yield of product **3a** began to decline, was consistent with the general kinetics observed for desaturation reactions (Scheme 3d). In addition, an on–off light experiment (Scheme 3e) was conducted and confirmed that a continuous irradiation of light was essential for driving the reaction. Further mechanistic insights of the reaction were obtained through spectroscopic studies aimed at confirming the formation of the EDA complex. The classical UV–vis spectroscopy revealed a clear red shift upon mixing compound **1a** with the pyridinium salt (**2h**), consistent with EDA complex formation (Scheme 3f).

**Scheme 3:**
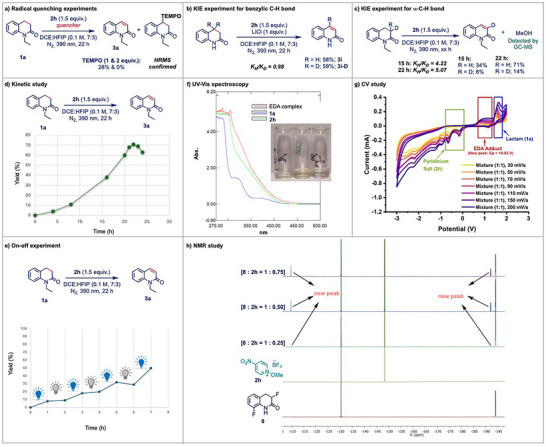
Detailed mechanistic studies for the dehydrogenation reactions.

**Scheme 4:**
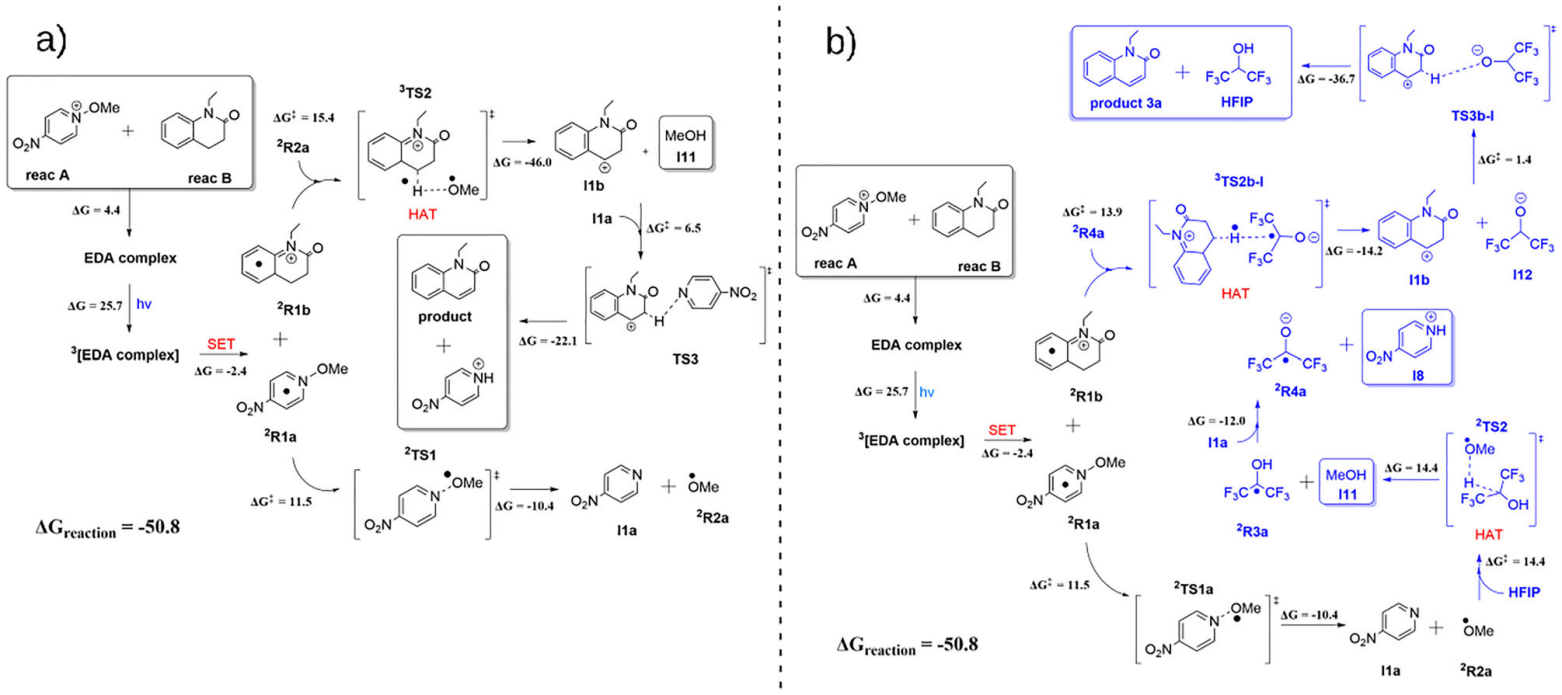
a) Computed mechanism for the reaction mechanism without the presence of HFIP. b) Revised computed mechanism with the presence of HFIP molecule. Energies are in kcal mol^−1^.

Complementary CV analyses revealed the appearance of a new peak at + 0.83 V in the mixture of **1a** and **2h**, further validating the presence of a discrete EDA complex (Scheme 3g). Moreover, ^19^F NMR analysis of the mixture of compound **8** and **2h** displayed new peaks, indicating the formation of a new species, attributed to the EDA adduct (Scheme 3h).

To elucidate the reaction mechanism further and understand the role of HFIP in enhancing the reaction yield, we carried out a series of DFT calculations with the B3LYP‐D3BJ SMD (DCE) method. Full computational details are supplied in the Supporting Information. A summary of all calculations is available in the ioChem‐BD repository.^[^ ^65^, ^66^, ^67^, ^68^ ^]^ We used the experimental setup of **entry 11** in Table 1 as the model conditions. We chose a temperature of 25 °C because this is the usual case in DFT calculations, the mechanism should be the same, and the change with the optimal conditions of **entry 6** in Table 1 is minor. We first studied the photochemical step and TD‐DFT calculations showed that only the EDA complex exhibited significant absorption, which was consistent with the experimental UV–vis data. The excitation process corresponded to a single electron transfer from reactant **2h** to reactant **1a**, as indicated by the natural population analysis (NPA) reported in the Supporting Information.

We next examined the free energy profile in the absence of HFIP, the reaction scheme is shown in Scheme 4a. After the excitation, there are two separate radicals, **^2^R1a** and **^2^R1b**. Their presence was moreover corroborated by TEMPO trapping experiments. Radical **^2^R1a** underwent methoxy release via a transition state (ΔG^‡^ = 11.5 kcal mol^−1^), forming intermediate **I1a** and methoxy radical **^2^R2a**. This methoxy radical then abstracted a hydrogen atom from other SET fragment **^2^R1b**, yielding methanol and cationic intermediate **I1b**. Finally, **I1a**, the most basic species in solution, abstracted a proton, affording product **3a** and protonated **I1a**. In the overall reaction, the two hydrogen atoms of reactant **B** were abstracted by a methoxy radical and by a base formed in the reaction medium.

We next studied the role of HFIP in this reaction. At first, we used HFIP as a reaction medium in the solvation model; however, the changes in the energies were negligible (see Supporting Information). We then included one explicit molecule of HFIP in the computational model. The revised mechanism, which explains the increased yield, is presented in Scheme 4b. The first step is the same, leading to the formation of a methoxy radical. The fate of this radical is different now as it reacts with an HFIP molecule (absent in the simplified model) via a HAT step with a barrier of 14.4 kcal mol^−1^. The resulting HFIP‐based radical **^2^R3a** then must first undergo deprotonation by intermediate **1a**, yielding **^2^R4a** before re‐entering the reaction following the pattern in the simplified model. In this case, it should be noted that the change of the barrier in the HAT step is lowered from 15.4 to 14.4 kcal mol^−1^. This signifies that HFIP is a transient H‐atom shuttle,^[^ ^27^ ^]^ which acts as a catalyst for the process, albeit a moderately efficient one, lowering the barrier by 1.0 kcal mol^−1^. The agreement with experiment is further confirmed by microkinetic modelling calculations^[^ ^68^ ^]^ (fully presented in the Supporting Information), which shows a decrease in yield from 53% to 21% when removing the explicit HFIP molecule from the model.

In summary, we have developed a photoinduced, EDA complex‐mediated strategy for the desaturation of a broad range of heterocyclic carbonyl compounds, including quinolinone, coumarin, and flavone scaffolds. This method addresses longstanding challenges in radical‐mediated carbonyl desaturation by employing an alternative mechanism involving carbocation generation followed by base‐induced E1 elimination, rather than the conventional dual hydrogen radical abstraction pathway. At the heart of this strategy is the formation of an EDA‐complex between the dihydrogenated substrate and an *N*‐methoxy pyridinium salt, which initiates a HAT process under mild, photochemical conditions. Notably, the pyridinium salt serves a dual function as both oxidant and base, streamlining the reaction and reducing the reliance on additional reagents. To address solubility challenges often encountered with ionic pyridinium salts, HFIP was employed as the solvent. HFIP not only enhances solubility via strong H‐bonding but also facilitates the HAT step via as a H‐shuttling platform. This unique role of HFIP, supported by DFT calculations and mechanistic studies, substantially lowers the activation energy of the transformation. Compared to conventional metal‐based or oxidant‐intensive protocols, our metal‐free, light‐driven approach offers a more sustainable, modular, and environmentally benign alternative. Collectively, these findings establish a robust platform for EDA‐mediated desaturation and highlight the untapped potential of HFIP as a H‐shuttle in radical‐mediated processes.

## Conflict of Interests

The authors declare no conflict of interest.

## Supporting information



Supporting Information

## Data Availability

The data that support the findings of this study are available on request from the corresponding author. The data are not publicly available due to privacy or ethical restrictions.
