# Supplementary material for: EDA Complex‐Driven Desaturation of Heterocyclic Carbonyl Compounds Enabled by HFIP
Source: Angew Chem Int Ed Engl. 2025 Oct 29;64(49):e202514539. doi: 10.1002/anie.202514539 (PMC12668311; doi:10.1002/anie.202514539)
Supplement: Supplementary file 1 — Supporting Information [file ANIE-64-e202514539-s001.pdf]

Angewandte Chemie
Eine Zeitschrift der Gesellschaft Deutscher Chemiker

Supporting Information

EDA Complex-Driven Desaturation of Heterocyclic Carbonyl Compounds Enabled by HFIP

Rakesh Maiti,[1‡] Robin Cauwenbergh,[2‡] Aritra Nath,[1] Ana B. R. Guimarães,[3] Yuman Qin[1], Feliu Maseras,[3] and Shoubhik Das[1,2]*

[1] R. Maiti, A. Nath, Y. Qin and S. Das*
University of Bayreuth, Universitätstr. 30, Bayreuth, Germany, 95447.
E-mail: Shoubhik.Das@uni-bayreuth.de

[2] R. Cauwenbergh
University of Antwerp, University of Antwerp, Antwerp 2020, Belgium.

[3] A. B. R. Guimarães and F. Maseras*
Institute of Chemical Research of Catalonia (ICIQ-CERCA), The Barcelona Institute of Science and Technology, Avgda, Països Catalans 16 – 43007 Tarragona, Spain
E-mail: fmaseras@iciq.es
‡ These authors contributed equally to this work

Table of Contents

1. **Materials and methods** 3
2. **Experimental section** 6
   2.1. **Dehydrogenation protocol** 6
   2.2. **Literature protocols** 6
   2.3. **Synthesis of substrates** 6
3. **Optimization studies** 11
4. **Mechanistic investigations** 13
   4.1. **Radical scavenger experiment** 13

KIE experiment: 14

   4.2. **On-off experiment** 15
   4.3. **Electrochemical Measurements:** 16
   4.4. **NMR study** 18
   4.5. **UV-Vis analysis:** 19
5. **Computational methods** 20
   5.1. **TD-DFT Calculations** 20
   5.2. **TD-DFT Transitions for the EDA Complex** 20
   5.3. **Natural population analysis of the excited state** 22
   5.4. **Reactions and Plausible Mechanisms** 22
6. **Cartesian Coordinates in Ångstrom and potential energies in hartree** 26
7. **Characterisations** 34
8. **NMR spectra** 39
9. **References** 86

1. Materials and methods

Materials. Chemicals and solvents were obtained from commercial suppliers (Sigma Aldrich, TCI, Thermo Fisher Scientific, J&K Scientific, Fluorochem and BLD pharmatech) and used without further purification. Non-commercial starting materials were prepared as described below according to literature procedures. All reactions were carried out under Nitrogen atmosphere using Schlenk techniques.

Analytical Methods. Thin Layer Chromatography (TLC) was performed using TLC plates from Merck (SiO_2, Kieselgel 60 F254 neutral, on aluminum with fluorescence indicator) and compounds were visualized by UV detection (254 nm). Flash column chromatographic purification of products was accomplished using an automated chromatography system with on-line UV detection (Biotage® or Combiflsh®Rf) using commercial SilicaFlash Cartridges and the indicated solvent and gradient system.

Gas chromatography-mass spectrometry (GC-MS) samples were prepared by dissolving 0.1-5 mg of the compound in acetone (GC-MS quality) and further diluted to a concentration of 10^{-5} - 10^{-6} M. The samples were subsequently filtered using a CHROMAFIL® PET-20/25 syringe filter and 3 µL were injected. The apparatus was an Agilent Technologies 7890 A GC System coupled to an Agilent Technologies 5975 C inert MSD with triple-axis detector. As column an Optima 725820.30 30 m × 250 µm × 0.25 µm was selected. Carrier gas was helium. Inlet temperature heater: 225 °C. Oven program: 70 °C for 3 min, then heating 5 °C min-1 to 160 °C and heating at 160 °C for 2 min. A one-minute post-run at 280 °C ends the oven program.

Nuclear Magnetic Resonance (NMR) spectra were recorded on a Bruker Avance III 400 (101 MHz for ^{13}C) Fourier Transform NMR spectrometer at 300 K, using tetramethylsilane (TMS) as the internal standard (^{1}H: δ = 0.00 ppm, ^{13}C: δ = 0.0 ppm). Chemical shifts (δ) are given in ppm and coupling constants (*J*) are reported in Hertz (Hz). Multiplicities are described as s (singlet), d (doublet), t (triplet), q (quartet), p (pentet), br s (broad singlet) and m (multiplet) or combinations thereof. ^{13}C NMR spectra were recorded with complete proton decoupling. ^{1}H NMR yields were determined by addition of a known amount of an internal standard (1,3,5-trimethoxybenzene) and dissolving everything in a suitable deuterated solvent, followed by ^{1}H NMR analysis.

High resolution mass spectrometry (HRMS) samples were prepared by dissolving 0.1-5 mg of the compound in DMSO or CH_3CN/H_2O and further diluted to a concentration of 10-5 - 10-6 M. Formic acid (0.1%) was added prior to injection. 10 µL of each sample was injected using the CapLC system (Waters, Manchester, UK) and electrosprayed using a standard electrospray source. Samples were injected with an interval of 3 minutes. Positive ion mode accurate mass spectra were acquired using a Q-TOF II instrument (Waters, Manchester, UK). The MS was calibrated prior to use with a 0.1% H_3PO_4 solution. The spectra were lock mass corrected using the known mass of the nearest H_3PO_4 cluster or a known background ion. Analytes were detected as protonated or as a sodium adduct. All measured masses are within a difference of 5 ppm compared to the calculated mass unless specified otherwise.

Computational Details. Geometry optimizations and energy calculations were performed using Gaussian 16.[1] Optimizations in solution employed the B3LYP functional[2] with Grimme's D3 dispersion correction and Becke–Johnson damping[3], in combination with the Def2-SVP basis set.[4] Solvent effects were modeled using the SMD implicit solvation model[5] with dichloroethane (DCE), the dominant solvent in the reaction medium. Solvation with hexafluoroisopropanol (HFIP) was applied only for the energy comparisons between both solvents for the rate determining step of the reaction. Frequency calculations at the same level were used to confirm all stationary points as minima (no imaginary frequencies) or transition states (one imaginary frequency along the reaction coordinate). Quasi-harmonic entropy corrections (with a 100 cm^{-1} cutoff)[6] and standard state corrections (1 M) were applied using GoodVibes[7] to obtain Gibbs free energies. For the final reported free energies, potential energies were refined with single-point energies were refined using the Def2-TZVPP basis set.[4] TD-DFT calculations were performed at the PBEPBE/Def2-TZVPP level with SMD solvation (DCE). Microkinetic modeling was carried out with COPASI v4.44.[8] Optimized 3D geometries were visualized using GaussView 6,[1] and energy profiles were plotted with mechaSVG[9]. All computational data are available at ioChem-BD.

Photocatalytic setup. The reaction setup is depicted in **Figure S1**. The reaction setup consists of commercially available KESSil lamps, which were purchased from Laser 2000 (UK) Ltd, with precise wavelengths of 390 nm (or 427 nm or 456 nm for control experiments). Magnetic stirring was performed at 500 rpm. The light intensity of Kessil lamp (λ = 390 nm) was 0.32 W/cm^2 and the distance between tube and light was maintained between 1 to 2 cm.

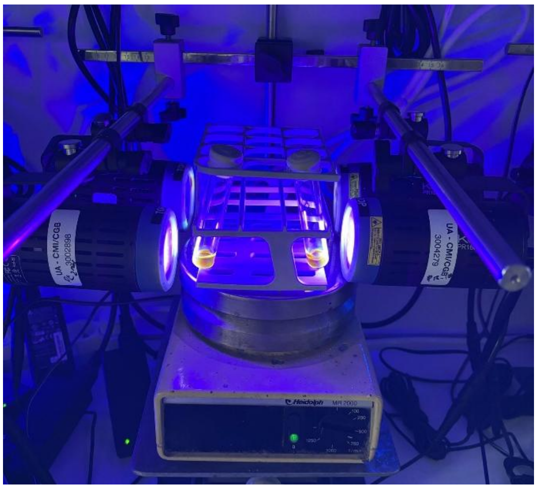

Figure S1: Kessil reaction setup

The Blue and the green LED setups are depicted in **Figure S2**. The reaction setups consist of a self-constructed light source configuration, made up of a crystallizing dish with a diameter of 140 mm. Inside of the crystallizing dish, commercially available 5 m LED-Strip is glued with separable LED elements. In total, 3 m LED strip is used in a crystallizing dish, with a total power of 24 W. Light intensity of the light source can be adjusted by a self-constructed dimmer. Construction of the reaction setup and the dimmer was performed by the electronic services of the faculty for chemistry of the Georg-August-Universität Göttingen. Cooling of the setup is performed by a commercially available 120 mm computer fan. The light intensities were measured to be 13000 lux for the blue LED setup and 35000 for the green LED setup.

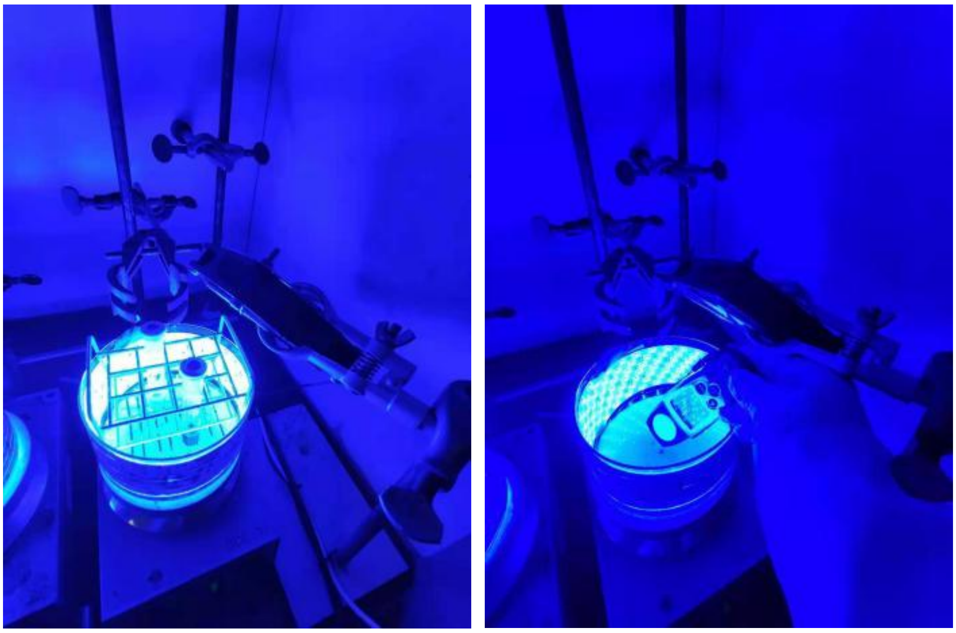

Figure S2: Blue LED reaction setup. Similar set-up was used for the green LED.

The emission spectra of the light setups were measured with a UV-Vis probe from Ocean optics (P200-5-UV-Vis). For the blue LED setup, the emission spectra showed the clear wavelength band between 404 and 553 nm with a maximum at 456 nm (**Figure S3**, left). For the green setup a clear wavelength band between 470 and 625 nm with a maximum at 522 nm was seen (**Figure S3**, right). The light intensity was measured by a lux meter, which can measure the value of different light intensity directly. Please note the distance between the sensor and light should be always same. In our case, we measured the light intensity in the center of the crystallizing dish.

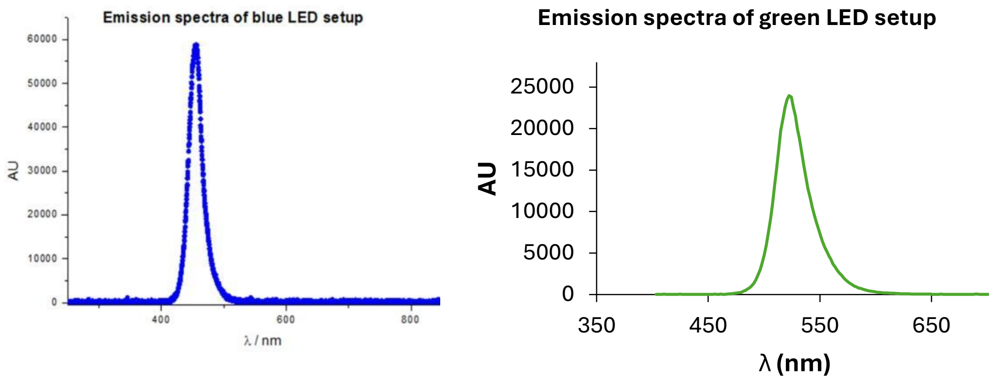

Figure S3: LED emission spectra.

2. Experimental section

2.1. Dehydrogenation protocol

General procedure A. A solution of substrate (0.1 mmol) and 1-methoxy-4-nitropyridin-1-ium tetrafluoroborate (1.5 equiv.) were put under nitrogen atmosphere using Schlenck techniques. Under nitrogen atmosphere, HFIP (0.3M) and DCE (0.7 M) were added. The reaction was stirred for 22 hours, before water was added. The mixture was extracted 3 times with EtOAc and concentrated under reduced pressure.

General procedure B. A solution of substrate (0.1 mmol), LiCl (1 equiv.) and 1-methoxy-4-nitropyridin-1-ium tetrafluoroborate (1.5 equiv.) were put under nitrogen atmosphere using Schlenck techniques. Under nitrogen atmosphere, HFIP (0.3M) and DCE (0.7 M) were added. The reaction was stirred for 22 hours, before water was added. The mixture was extracted 3 times with EtOAc and concentrated under reduced pressure.

2.2. Literature protocols

General procedure C. A solution of 3,4-dihydroquinolin-2(1*H*)-one (15 mmol) in anhydrous DMF (0.7 M) was cooled to 0 °C, under N_2 atmosphere. To this solution was added NaH (60% dispersion in mineral oil, 1.5 equiv.) and the solution was stirred for 30 minutes. Iodoalkane was added, and the solution was stirred at room temperature for 40 hours. After, the reaction was quenched with H_2O and extracted with EtOAc (3x). The combined organic layers were washed with saturated NH_4Cl, dried over anhydrous Na_2SO_4, filtered and concentrated under reduced pressure. Product was purified by flash column chromatography to give the *N*-alkylated product.

General procedure D. A solution of 3,4-dihydroquinolin-2(1*H*)-one (7.79 mmol), CuI (0.1 equiv.), K_2CO_3 (1.1 equiv.) and the bromoaryl compound (2 equiv.) in anhydrous DMF was refluxed for 48 hours under N_2 atmosphere. After completion, the mixture was cooled to room temperature, diluted with H_2O and extracted with EtOAc (3x). The combined organic layers were washed with saturated NH_4Cl, dried over anhydrous Na_2SO_4 and concentrated under reduced pressure. The product was purified by flash column chromatography to give the *N*-arylated product.

General procedure E. A solution of K_2CO_3 (x equiv.), 7-hydroxy-3,4-dihydroquinolin-2(1*H*)-one (1 equiv.) was put under nitrogen atmosphere using Schlenck techniques. Under the nitrogen atmosphere, the solvent DMF (0.3 M) was added. The resulting mixture was stirred at r. t. for 30 min. To the solution was added the alkyl halide (2 mol, 1 equiv.) in DMF (1 M) in one portion and was stirred continuously at room temperature for 24 h. After, HCl (0.4 M) was added and was extracted with ethyl acetate (3 times). The combined organic layers were dried over anhydrous Na_2SO_4, filtered and concentrated in vacuo. Products were purified by flash column chromatography.

General procedure F. A solution of DMAP (0.02 equiv.), acid (3 mmol), EDC (0.5 equiv.) and 7-amino-3,4-dihydroquinolin-2(1*H*)-one (1 equiv.) or 7-hydroxy-2-phenylchroman-4-one (1 equiv.) was put under nitrogen atmosphere using Schlenck techniques. Under the nitrogen atmosphere, DCM (0.2 M) was added. The resulting mixture was stirred at r.t. for 24 h. Then, the resulting mixture is concentrated *in vacuo*. Products were purified by flash column chromatography with ethyl acetate and *n*-hexane as solvents.

2.3. Synthesis of substrates

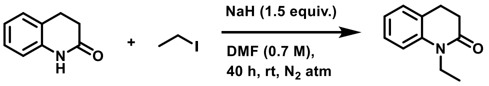

1-Ethyl-3,4-dihydroquinolin-2(1*H*)-one: The compound was synthesized using general procedure C using 3,4-dihydroquinolin-2(1*H*)-one and iodomethane. Column chromatography (gradient 5% to 20% EtOAc/heptane) gave title compound as a colourless oil (Isolated yield: 90%). Spectral date were consistent with previous literature.[10]
^{1}H NMR (400 MHz, $CDCl_3$) δ 7.28 – 7.21 (m, 1H), 7.16 (dd, *J* = 7.4, 1.4 Hz, 1H), 7.07 – 6.90 (m, 2H), 3.99 (q, *J* = 7.1 Hz, 2H), 2.88 (dd, *J* = 8.6, 6.1 Hz, 2H), 2.69 – 2.57 (m, 2H), 1.26 (t, J = 7.1 Hz, 3H). **^{13}C NMR** (101 MHz, $CDCl_3$): δ 170.0, 139.7, 128.1, 127.6, 126.7, 122.8, 114.8, 37.5, 32.1, 25.7, 12.9. **MS (TLC-MS)** : m/z [M+Na]$^+$: 198.

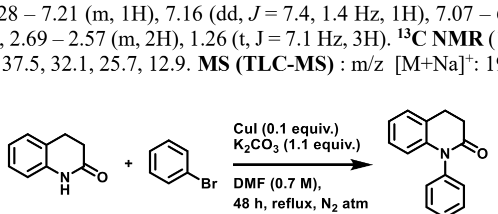

1-Phenyl-3,4-dihydroquinolin-2(1*H*)-one: The compound was synthesized using general procedure D using 3,4-dihydroquinolin-2(1*H*)-one and bromobenzene. Column chromatography (gradient 5% to 20% EtOAc/heptane) gave title compound as a white solid (Isolated yield: 70%). Spectral date were consistent with previous literature.[11]
^{1}H NMR (400 MHz, $CDCl_3$) δ 7.53 – 7.48 (m, 2H), 7.43 – 7.39 (m, 1H), 7.25 – 7.19 (m, 3H), 7.01 (dtd, *J* = 22.2, 7.4, 1.5 Hz, 2H), 6.35 (dd, *J* = 8.0, 1.1j Hz, 1H), 3.07 (dd, *J* = 8.5, 6.2 Hz, 2H), 2.85 – 2.81 (m, 2H). **^{13}C NMR** (101 MHz, $CDCl_3$): δ 170.3, 141.9, 138.6, 130.0, 129.2, 128.3, 127.9, 127.3, 125.8, 123.1, 117.2, 32.4, 25.8. **MS (TLC-MS) :** m/z [M+Na]$^+$: 246.

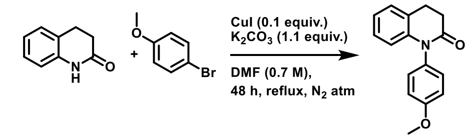

1-(4-Methoxyphenyl)-3,4-dihydroquinolin-2(1*H*)-one: The compound was synthesized using general procedure D using 3,4-dihydroquinolin-2(1*H*)-one and 4-bromoanisole. Column chromatography (gradient 5% to 30% EtOAc/heptane) gave title compound as a white solid (Isolated yield: 71%). Spectral date were consistent with previous literature.[11]
^{1}H NMR (400 MHz, $CDCl_3$) δ 7.22 – 7.16 (m, 1H), 7.16 – 7.08 (m, 2H), 7.07 – 6.87 (m, 4H), 6.40 (dd, J = 8.0, 1.2 Hz, 1H), 3.85 (s, 3H), 3.06 (dd, J = 8.6, 6.1 Hz, 2H), 2.86 – 2.73 (m, 2H).). **^{13}C NMR** (101 MHz, $CDCl_3$): δ 170.6, 159.4, 142.1, 131.1, 130.1, 127.8, 127.3, 125.8, 123.0, 117.1, 115.3, 55.6, 32.4, 25.8. **MS (TLC-MS) :** m/z [M+Na]$^+$: 276.

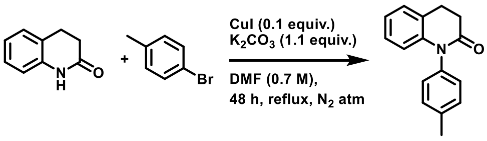

1-(P-tolyl)-3,4-dihydroquinolin-2(1*H*)-one: The compound was synthesized using general procedure D using 3,4-dihydroquinolin-2(1*H*)-one and 4-bromotoluene. Column chromatography (gradient 5% to 20% EtOAc/heptane) gave title compound as a white solid (Isolated yield: 80%). Spectral date were consistent with previous literature.[11]
^{1}H NMR (400 MHz, $CDCl_3$) δ 7.30 (d, *J* = 8.0 Hz, 2H), 7.19 (dd, *J* = 7.1, 1.6 Hz, 1H), 7.14 – 7.09 (m, 2H), 7.03 (td, *J* = 7.8, 1.7 Hz, 1H), 6.97 (td, *J* = 7.4, 1.3 Hz, 1H), 6.38 (dd, *J* = 8.0, 1.2 Hz, 1H), 3.06 (dd, *J* = 8.7, 6.1 Hz, 2H), 2.88 – 2.75 (m, 2H), 2.41 (s, 3H). **^{13}C NMR** (101 MHz, $CDCl_3$): δ 170.4, 141.9, 138.2, 135.9, 130.7, 128.8, 127.9, 127.2, 125.7, 123.0, 117.1, 32.4, 25.8, 21.3. **MS (TLC-MS) :** m/z [M+Na]$^+$: 260.

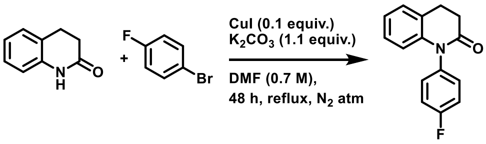

1-(4-Fluorophenyl)-3,4-dihydroquinolin-2(1*H*)-one: The compound was synthesized using general procedure D using 3,4-dihydroquinolin-2(1*H*)-one and 1-bromo-4-fluorobenzene. Column chromatography (gradient 5% to 20% EtOAc/heptane) gave title compound as a white solid (Isolated yield: 77%). Spectral date were consistent with previous literature.[11]
^{1}H NMR (400 MHz, $CDCl_3$) δ 7.24 – 7.16 (m, 5H), 7.06 (td, *J* = 7.8, 1.7 Hz, 1H), 6.99 (td, *J* = 7.4, 1.3 Hz, 1H), 6.35 (dd, *J* = 8.0, 1.3 Hz, 1H), 3.06 (dd, *J* = 8.6, 6.1 Hz, 2H), 2.86 – 2.78 (m, 2H). **^{13}C NMR** (101 MHz, $CDCl_3$): δ 170.3, 162.1 (d, *J* = 247.7 Hz), 141.6, 134.3 (d, *J* = 3.3 Hz), 130.8 (d, *J* = 8.6 Hz), 127.9 (s), 127.2, 125.7, 123.1, 116.9 (d, *J* = 9.0 Hz), 116.7, 32.2, 25.6. **^{19}F NMR** (377 MHz, $CDCl_3$) δ -113.4 (tt, *J* = 7.7, 5.5 Hz) **MS (TLC-MS) :** m/z [M+Na]$^+$: 264.

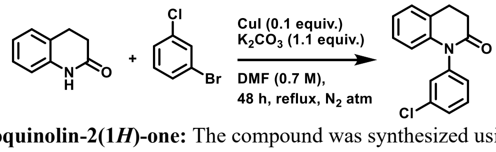

1-(3-Chlorophenyl)-3,4-dihydroquinolin-2(1*H*)-one: The compound was synthesized using general procedure D using 3,4-dihydroquinolin-2(1*H*)-one and 1-bromo-3-chlorobenzene. Column chromatography (gradient 5% to 20% EtOAc/heptane) gave title compound as a white solid (Isolated yield: 80%). Spectral date were consistent with previjous literature.[12]
^{1}H NMR (400 MHz, $CDCl_3$) δ 7.47 – 7.37 (m, 2H), 7.26 (dd, *J* = 4.0, 2.0 Hz, 1H), 7.23 – 7.19 (m, 1H), 7.15 (dt, *J* = 7.5, 1.7 Hz, 1H), 7.06 (td, *J* = 7.8, 1.7 Hz, 1H), 7.00 (td, *J* = 7.4, 1.3 Hz, 1H), 6.37 (dd, *J* = 8.0, 1.3 Hz, 1H), 3.06 (dd, *J* = 8.6, 6.1 Hz, 2H), 2.90 – 2.72 (m, 2H). **^{13}C NMR** (101 MHz, $CDCl_3$): δ 170.2, 135.3, 135.0, 130.9, 130.7, 129.5, 127.9, 127.3, 125.8, 123.3, 117.0, 25.6. **MS (TLC-MS) :** m/z [M+Na]$^+$: 280.

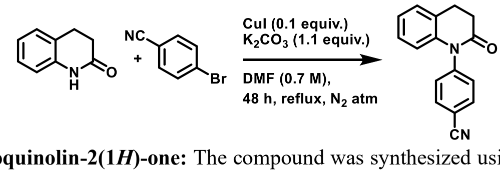

1-(4-Cyanophenyl)-3,4-dihydroquinolin-2(1*H*)-one: The compound was synthesized using general procedure D using 3,4-dihydroquinolin-2(1*H*)-one and 4-bromobenzonitrile. Column chromatography (gradient 5% to 40% EtOAc/heptane) gave title compound as a white solid (Isolated yield: 76%). Spectral data were consistent with those reported previously. [13]
^{1}H NMR (400 MHz, $CDCl_3$) δ 7.80-7.77 (m, 2H), 7.41-7.38 (m, 2H), 7.25-7.23 (m, 1H), 7.10-7.01 (m, 2H), 6.33 (dd, *J* = 7.8, 1.1 Hz, 1H), 3.09-3.06 (m, 2H), 2.84-2.81 (m, 2H). **^{13}C NMR** (101 MHz, $CDCl_3$): δ 170.0, 142.9, 140.8, 133.6, 130.1, 128.1, 127.4, 126.2, 123.7, 118.2, 117.1, 111.9. **MS (TLC-MS) :** m/z [M+Na]$^+$: 271.

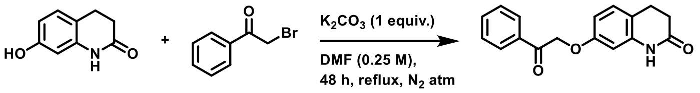

7-(2-Oxo-2-phenylethoxy)-3,4-dihydroquinolin-2(1*H*)-one: The compound was synthesized using general procedure E using 7-hydroxy-3,4-dihydroquinolin-2(1*H*)-one, 2-bromo-1-phenylethan-1-one and K_2CO_3 (1 equiv.). Column chromatography (gradient 10% to 40% EtOAc/heptane) gave title compound as a white solid (Isolated yield: 50%). Spectral data were consistent with those reported previously.[14]

^{1}H NMR (400 MHz, $CDCl_3$) δ 7.99 (m, 2H), 7.70 (s, 1H), 7.63 (m, 1H), 7.51 (m, 2H), 7.05 (d, *J* = 8.3 Hz, 1H), 6.54 (dd, *J* = 8.3, 2.5 Hz, 1H), 6.37 (d, *J* = 2.5 Hz, 1H), 5.25 (s, 2H), 2.91 – 2.87 (m, 2H), 2.60 (dd, *J* = 8.3, 6.7 Hz, 2H). **^{13}C NMR** (101 MHz, $CDCl_3$): δ 194.5, 171.4, 157.8, 138.4, 134.7, 134.1, 129.0, 129.0, 128.3, 117.1, 108.8, 102.9, 71.1, 31.1, 24.8. **MS (TLC-MS) :** m/z [M+Na]$^+$: 304.

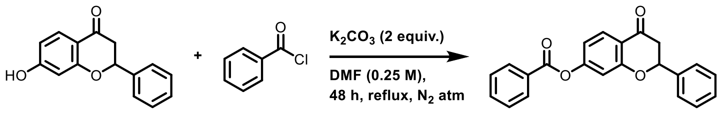

4-Oxo-2-phenylchroman-7-yl benzoate: The compound was synthesized using general procedure E using 7-hydroxy-2-phenylchroman-4-one, benzoyl chloride and K_2CO_3 (2 equiv.). Column chromatography (gradient 5% to 20% EtOAc/heptane) gave title compound as a white solid (Isolated yield: 40%). Spectral data were consistent with those reported previously.[15]

^{1}H NMR (400 MHz, $CDCl_3$) δ 8.20 – 8.17 (m, 2H), 8.01 (d, *J* = 8.6 Hz, 1H), 7.67 – 7.63 (m, 1H), 7.54 – 7.38 (m, 7H), 6.99 (d, *J* = 2.0 Hz, 1H), 6.94 (dd, *J* = 8.6, 2.2 Hz, 1H), 5.53 (dd, *J* = 13.2, 2.9 Hz, 1H), 3.10 (dd, *J* = 16.9, 13.2 Hz, 1H), 2.91 (dd, *J* = 16.9, 3.0 Hz, 1H). **^{13}C NMR** (101 MHz, $CDCl_3$): δ 191.0, 164.5, 162.7, 157.1, 138.6, 134.1, 130.4, 130.3, 129.0, 129.0, 128.8, 128.7, 126.3, 119.1, 116.0, 111.5, 80.2, 44.6 **MS (TLC-MS) :** m/z [M+Na]$^+$: 367.

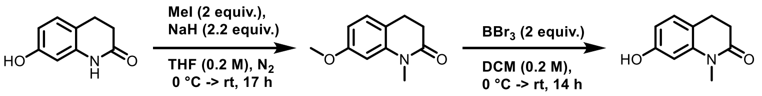

7-Methoxy-1-methyl-3,4-dihydroquinolin-2(1*H*)-one: A 50 mL round bottom flask was charged with 7-hydroxy-3,4-dihydroquinolin-2(1*H*)-one (5 mmol) and added THF (0.6 M) under nitrogen atmosphere. Then the solution was cooled to 0 °C and added NaH (11 mmol, 2.2 equiv.). After 15 minutes of stirring, iodomethane (2 equiv.) was added to the mixture and continued to stir for 17 hours at room temperature. After completion of the reaction, the solution was quenched by MeOH (1 mL) and water. The crude product was purified via silica gel chromatography (Heptane/EtOAc = 1:1) and gave title compound as a white solid (isolated yield 34%). Spectral data were consistent with those reported previously.[16]

^{1}H NMR (400 MHz, $CDCl_3$): δ 7.06 (dd, *J* = 7.8, 0.8 Hz, 1H), 6.56 – 6.53 (m, 2H), 3.81 (s, 3H), 3.33 (s, 3H), 2.83 (dd, *J* = 8.6, 6.1 Hz, 2H), 2.63 (dd, *J* = 8.5, 6.2 Hz, 2H). **^{13}C NMR** (101 MHz, $CDCl_3$): δ 170.7, 159.4, 141.8, 128.3, 118.6, 106.7, 102.6, 55.6, 32.2, 29.7, 24.8. **MS (TLC-MS) :** m/z [M+Na]$^+$: 214.

***N*-Methyl-7-hydroxy-3,4-dihydroquinolin-2(1*H*)-one:** To a 0 °C solution of freshly purified product 7-methoxy-1-methyl-3,4-dihydroquinolin-2(*1H*)-one (2 mmol) in DCM (10 mL) was dropwise added BBr_3 (0.40 mL, 2.0 equiv). Then the resulting mixture was slowly warmed up to 23 °C and stirred for 14 h. The reaction mixture was concentrated under reduced pressure by rotary evaporation. Purification by flash column chromatography on silica gel (EtOAc/DCM/hexanes = 1:1:1) afforded the product 7-hydroxy-1-methyl-3,4-dihydroquinolin-2(1H)-one as a yellow solid (Isolated yield: 77 %). Spectral data were consistent with those reported previously.[16]

^{1}H NMR (400 MHz, $CDCl_3$): δ 9.37 (s, 1H), 6.98 (d, *J* = 8.1 Hz, 1H), 6.49 (d, *J* = 2.3 Hz, 1H), 6.41 (dd, *J* = 8.0, 2.3 Hz, 1H), 3.19 (s, 3H), 2.75 – 2.71 (m, 2H), 2.49 – 2.47 (m, 2H). **^{13}C NMR** (101 MHz, $CDCl_3$): δ 169.4, 156.7, 141.2, 128.1, 116.1, 108.7, 102.6, 31.7, 28.9, 23.8. **MS (TLC-MS) :** m/z [M+Na]$^+$: 200.

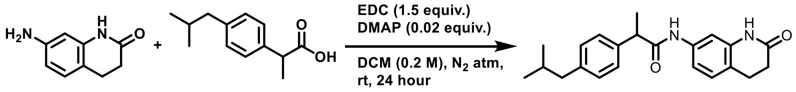

2-(4-Isobutylphenyl)-*N*-(2-oxo-1,2,3,4-tetrahydroquinolin-7-yl)propenamide: The compound was synthesized using general procedure F using 7-amino-3,4-dihydroquinolin-2(1*H*)-one and ibuprofen (2-(4-isobutylphenyl) propionic acid). Column chromatography (gradient 10% to 40% EtOAc/heptane) gave title compound as a white solid (Isolated yield: 40%).

^{1}H NMR (400 MHz, $CDCl_3$ δ 8.99 (s, 1H), 7.63 (s, 1H), 7.25 (d, *J* = 7.2 Hz, 2H), 7.10 (d, *J* = 8.1 Hz, 3H), 7.02 – 6.94 (m, 2H), 3.70 (q, *J* = 7.1 Hz, 1H), 2.82 (t, *J* = 7.5 Hz, 2H), 2.52 (dd, *J* = 8.3, 6.7 Hz, 2H), 2.43 (d, *J* = 7.2 Hz, 2H), 1.83 (dp, *J* = 13.6, 6.8 Hz, 1H), 1.54 (d, *J* = 7.1 Hz, 3H), 0.88 (d, *J* = 6.6 Hz, 6H). **^{13}C NMR** (101 MHz, $CDCl_3$): δ 173.0, 172.3, 140.9, 138.2, 137.6, 129.7, 129.3, 128.1, 127.4, 119.2, 114.3, 107.2, 47.6, 45.0, 30.7, 30.1, 24.7, 22.4, 18.6. **MS (TLC-MS) :** m/z [M+Na]$^+$: 373.

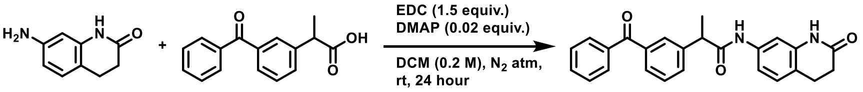

2-(3-benzoylphenyl)-*N*-(2-oxo-1,2,3,4-tetrahydroquinolin-7-yl)propanamide: The compound was synthesized using general procedure F using 7-amino-3,4-dihydroquinolin-2(1*H*)-one and ketoprofen (2-(3-benzoylphenyl)propanoic acid). Column chromatography (gradient 20% to 60% EtOAc/heptane) gave title compound as a white solid (Isolated yield: 43%).
^{1}H NMR (400 MHz, $CDCl_3$) δ 8.48 (s, 1H), 7.84 (s, 2H), 7.78 – 7.76 (m, 2H), 7.64 (dd, *J* = 7.6, 1.5 Hz, 2H), 7.59 – 7.55 (m, 1H), 7.47 – 7.41 (m, 3H), 7.15 (s, 1H), 7.01 – 6.96 (m, 2H), 3.80 (q, *J* = 7.0 Hz, 1H), 2.84 (t, *J* = 7.5 Hz, 2H), 2.54 (dd, *J* = 8.4, 6.7 Hz, 2H), 1.58 (d, *J* = 7.1 Hz, 3H). **^{13}C NMR** (101 MHz, $CDCl_3$): δ 196.7, 172.0, 171.7, 141.7, 138.1, 137.7, 137.4, 137.3, 132.7, 131.5, 130.1, 129.4, 129.2, 128.8, 128.4, 128.2, 119.5, 114.3, 107.2, 47.8, 30.8, 24.8, 18.8. **MS (TLC-MS) :** m/z $[M+Na]^+$: 421.

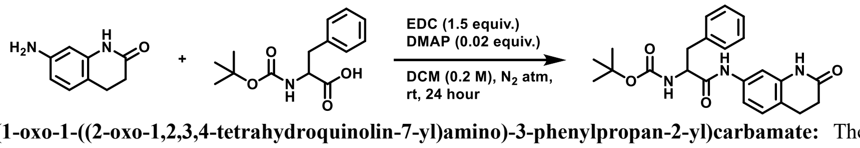

Tert-butyl (1-oxo-1-((2-oxo-1,2,3,4-tetrahydroquinolin-7-yl)amino)-3-phenylpropan-2-yl)carbamate: The compound was synthesized using general procedure F using 7-amino-3,4-dihydroquinolin-2(1*H*)-one and phenylalanine. Column chromatography (gradient 20% to 60% EtOAc/heptane) gave title compound as a white solid (Isolated yield: 50%).
^{1}H NMR (400 MHz, $CDCl_3$) δ 8.85 (s, 1H), 8.13 (s, 1H), 7.43 (s, 1H), 7.36 – 7.12 (m, 5H), 6.99 (d, *J* = 8.1 Hz, 1H), 6.62 (d, *J* = 6.6 Hz, 1H), 6.07 (s, 1H), 4.50 (d, *J* = 6.6 Hz, 1H), 3.19 – 3.08 (m, 2H), 2.89 (t, *J* = 7.5 Hz, 2H), 2.63 – 2.59 (m, 2H), 1.40 (s, 9H). **^{13}C NMR** (101 MHz, $CDCl_3$): δ 172.3, 171.7, 137.9, 137.0, 129.5, 129.3, 128.6, 127.7, 126.7, 119.1, 113.9, 107.3, 80.2, 57.5, 38.0, 30.8, 28.4, 25.0. **MS (TLC-MS) :** m/z $[M+Na]^+$: 432.

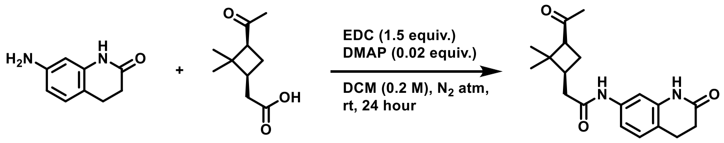

2-(3-acetyl-2,2-dimethylcyclobutyl)-*N*-(2-oxo-1,2,3,4-tetrahydroquinolin-7-yl)acetamide: The compound was synthesized using general procedure F using 7-amino-3,4-dihydroquinolin-2(1*H*)-one and pinonic acid. Column chromatography (gradient 20% to 60% EtOAc/heptane) gave title compound as a white solid (isolated yield: 56%).
^{1}H NMR (400 MHz, DMSO) δ 10.05 (s, 1H), 9.78 (s, 1H), 7.22 (d, *J* = 1.6 Hz, 1H), 7.08 – 7.02 (m, 2H), 2.93 (dd, *J* = 10.1, 7.5 Hz, 1H), 2.81 – 2.77 (m, 2H), 2.43 – 2.38 (m, 2H), 2.36 – 2.28 (m, 1H), 2.27 – 2.19 (m, 2H), 1.99 (s, 3H), 1.89 (dd, *J* = 10.5 Hz, 1H), 1.77 (m, 1H), 1.27 (s, 3H), 0.82 (s, 3H). **^{13}C NMR** (101 MHz, DMSO) δ 207.5, 170.7, 170.6, 138.9, 138.8, 128.1, 118.6, 113.2, 106.7, 53.7, 43.4, 38.7, 37.8, 31.1, 30.4, 30.2, 24.8, 23.2, 17.7. **MS (TLC-MS) :** m/z $[M+Na]^+$: 351.

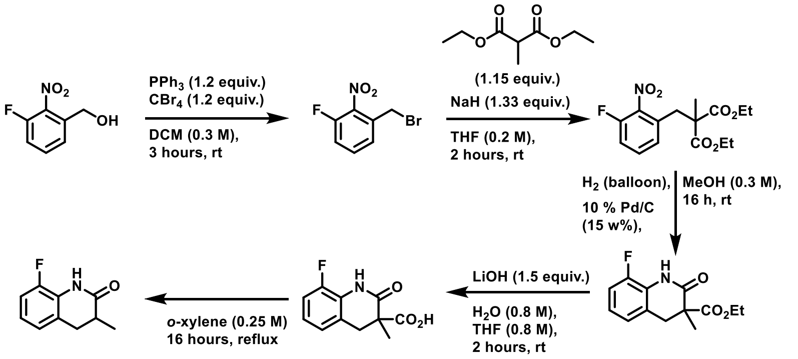

1-(Bromomethyl)-3-fluoro-2-nitrobenzene: To a solution of (3-fluoro-2-nitrophenyl)methanol (1 gram) in DCM (0.3 M) was added CBr_4 (1.2 equivalents) and triphenylphosphine (1.2 equivalents) in portions under nitrogen atmosphere. The reaction was stirred at room temperature for 3 hours before it was quenched with saturated aqueous NH_4Cl and extracted with DCM (3 times), dried with Na_2SO_4, filtered and concentrated. The crude mixture was purified by column chromatography (0-10% EtOAc:heptane) to provide 1-(bromomethyl)-3-fluoro-2-nitrobenzene as a yellow oil.[17]
Diethyl 2-(3-fluoro-2-nitrobenzyl)-2-methylmalonate: To a solution of 1,3-diethyl 2-fluoroproanedioate (1.15 equivalents) in THF (0.2 M) at 0 °C was added sodium hydride (60%, 1.33 equivalents) in portions and was stirred for 30 minutes at room temperature. 1-(Bromomethyl)-3-fluoro-2-nitrobenzene (1 equivalent) was added and this was stirred at room temperature for 2 hours. Saturated aqueous NH_4Cl (40 mL) and extracted with EtOAc (3 x 30 mL), dried with Na_2SO_4, filtered and concentrated. The crude material was purified using column chromatography (0-50% EtOAc: hept) to provide diethyl 2-fluoro-2-(3-fluoro-2-nitrobenzyl)malonate as a light yellow oil.[17]

Ethyl 8-fluoro-3-methyl-2-oxo-1,2,3,4-tetrahydroquinoline-3-carboxylate: To a solution of diethyl 2-fluoro-2-(3-fluoro-2-nitrobenzyl)malonate (1 equivalent) in MeOH (0.3 M) was added 10% Pd/C (15 w% compared to SM), and the reaction was stirred at RT for 16h under H_2 (balloon). The suspension was filtered through Celite and washed with EtOAc (3 times). The combined fractions were concentrated to form a light yellow solid, which was used without purification in the next step.[17]

8-Fluoro-3-methyl-2-oxo-1,2,3,4-tetrahydroquinoline-3-carboxylic acid: To a solution of ethyl 3,8-difluoro-2-oxo-1,2,3,4-tetrahydroquinoline-3-carboxylate (1 equivalent) in THF (0.8 M) was added LiOH (1.5 equivalents) in H_2O (1 M) and this was stirred at rt for 2h. The pH was adjusted with saturated aqueous citric acid, extracted with EtOAc (3 x 20 mL), dried with Na_2SO_4, filtered and concentrated in vacuo to gain a white solid.[17]

8-Fluoro-3-methyl-3,4-dihydroquinolin-2(*1H*)-one: A solution of 3,8-difluoro-2-oxo-1,2,3,4-tetrahydroquinoline-3-carboxylic acid (1 equivalent) in *o*-xylene (0.25 M) was stirred at reflux °C for 16 h, then cooled, concentrated and purified by FCC (0-50%) to provide the product as a light yellow solid.[17]

^{1}H NMR (400 MHz, DMSO-d6): δ 10.01 (s, 1H), 7.04 (m, 2H), 6.94 (qd, *J* = 8.3, 5.1 Hz, 1H), 3.00 (dt, *J* = 15.6, 5.1 Hz, 1H), 2.70 (dd, *J* = 15.6, 11.7 Hz, 1H), 2.56 (m, 1H), 1.13 (d, *J* = 6.8 Hz, 3H). **^{13}C NMR (101 MHz, DMSO-d6)**: δ 173.0, 149.7 (d), 127.1 (d), 126.5 (d), 123.9 (d), 122.7 (d), 114.1 (d), 34.5, 33.1 (d), 15.5. **^{19}F NMR (377 MHz, DMSO-d6)**: δ -131.34 (dd, *J* = 10.9, 5.1 Hz). **MS (TLC-MS) :** m/z [M+Na]$^+$: 202.

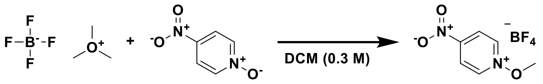

1-Methoxy-4-nitropyridine tetrafluoroborate: Trimethyloxonium tetrafluoroborate (1.1 equivalents) was added to a solution of the 4-nitro-1-oxido-pyridin-1-ium (1 equivalent) in DCM (0.3 M). The mixture was stirred at room temperature overnight, before methanol was added. The solvents were removed in vacuo, and the corresponding *N*-alkoxypyridinium tetrafluoroborate was recrystallized in MeOH. The collected solid was washed with Et_2O.

^{1}H NMR (400 MHz, CD_3CN) δ 9.32 (d, *J* = 7.6 Hz, 1H), 8.77 (d, *J* = 7.5 Hz, 1H), 4.53 (s, 1H). **^{13}C NMR** (101 MHz, CD_3CN) δ 145.2, 124.9, 118.3, 71.4. **^{19}F NMR** (376 MHz, CD_3CN) δ -151.41. **HRMS** (ESI) [cation]$^+$ calculated for $[C_6H_7N_2O_3]^+$ requires m/z 155.0451, found m/z 155.0454.

3. Optimization studies

Table S1: Screening of different solvents for the dehydrogenation of *N*-ethyl-3,4-dihydro-2-quinolinone.[a]

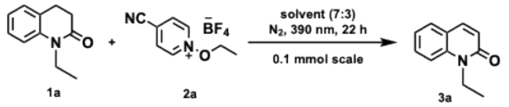

Entry	Solvent	Yield 3 (%)[b]
1	DCM	41
2	DCE	48
3	DMSO	Trace
4	DMA	Trace
5	DMF	Trace
6	Toluene	8
7	MeCN	9
8	Acetone	9
9	THF	8
10	HFIP	50
11	DCE:HFIP (1:9)	45
12	DCE:HFIP (2:8)	46
13	DCE:HFIP (3:7)	52
14	DCE:HFIP (4:6)	50
15	DCE:HFIP (5:5)	48
16	DCE:HFIP (6:4)	50
**17**	**DCE:HFIP (7:3)**	**58**
18	DCE:HFIP (8:2)	52
19	DCE:HFIP (9:1)	50
20	DCE:HFIP (7:3, 0.05 M)	44
21	DCE:HFIP (7:3, 0.2 M)	57

[a]Reaction conditions: **1** (0.1 mmol) and **2** (1.5 equiv.) in solvent (0.1 M) under N_2 atmosphere and light irradiation (390 nm 40 W KESSIL) for 22 hours. [b]Yield determined by ^{1}H NMR using 1,3,5-trimethyoxybenzene as the internal standard.

Table S2: Screening of different *N*-alkoxypyridinium tetrafluoroborates for the dehydrogenation of *N*-ethyl-3,4-dihydro-2-quinolinone.[a]

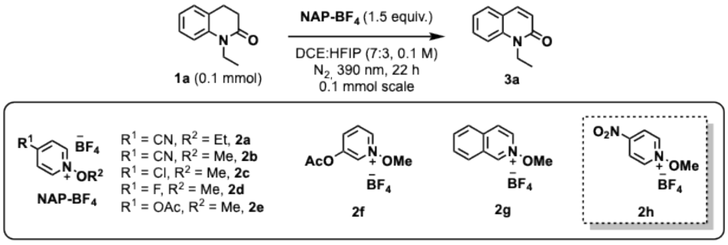

Entry	*N*-alkoxypyridinium tetrafluoroborate	Yield 3 (%)[b]
1	2a	58
2	2b	64
3	2c	13
4	2d	6
5	2e	n.d.
6	2f	16
7	2g	19
**8**	**2h**	**71**
9	2h (1 equiv.)	43
10	2h (2 equiv.)	72
11	2h (2.5 equiv.)	70
12	2h (3 equiv.)	61

[a]Reaction conditions: **1** (0.1 mmol) and *N*-alkoxypyridinium tetrafluoroborate (1.5 equiv.) in DCE:HFIP (7:3, 0.1 M) under N_2 atmosphere and light irradiation (390 nm 40 W KESSIL) for 22 hours. [b]Yield determined by ^{1}H NMR using 1,3,5-trimethyoxybenzene as the internal standard. n.d. = not detected

Table S3: Screening of reaction times for the dehydrogenation of *N*-ethyl-3,4-dihydro-2-quinolinone.[a]

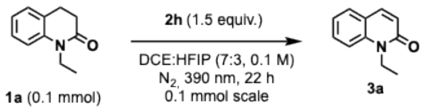

Entry	Time (h)	Yield 3 (%)[b]
1	0	0
2	4	4
3	8	11
4	16	38
5	20	60
6	21	68
7	22	71
8	23	69
9	24	63

[a]Reaction conditions: **1** (0.1 mmol) and **4** (1.5 equiv.) in DCE:HFIP (7:3, 0.1 M) under N_2 atmosphere and light irradiation (390 nm 40 W KESSIL) for x hours. [b]Yield determined by ^{1}H NMR using 1,3,5-trimethyoxybenzene as the internal standard.

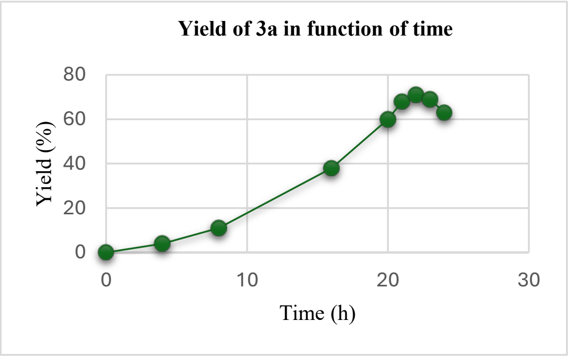

Figure S4: Yield of **3a** in function of time.

Table S4: Screening of various conditions for the dehydrogenation of *N*-ethyl-3,4-dihydro-2-quinolinone.[a]

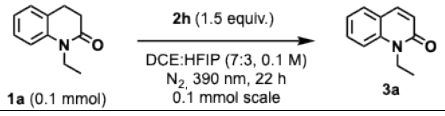

Entry	Deviation	Yield 3a (%)[b]
**1**	**None**	**71**
2	Cooling to ± 25 °C by using a fan	53
3	Aerobic conditions	46
4	LiCl (1 equiv.)	67
5	blue LED	Trace
6	green LED	n.d.
7	no light	n.d.
8	no light, 60 °C	n.d.
9	no **4h**	n.d.

[a]Reaction conditions: **1** (0.1 mmol) and **4** (1.5 equiv.) in DCE:HFIP (7:3, 0.1 M) under N_2 atmosphere and light irradiation (390 nm 40 W KESSIL) for x hours. [b]Yield determined by ^{1}H NMR using 1,3,5-trimethyoxybenzene as the internal standard.

4. Flow experiment

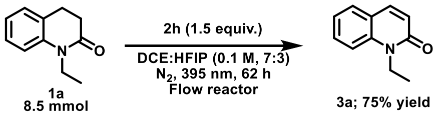

To further support the scalability and safety advantages, we investigated a continuous flow setup. A UV-150 photochemical reactor (Vapourtec Ltd.) equipped with a 10 mL reactor cartridge (Part No. 50-1580) was employed. The system operated in a closed-loop circulation mode with a reactant flow rate of 0.2 mL min^{-1} under N_2 atmosphere, under irradiation with a monochromatic LED lamp (λ_{max} = 395 nm, 6 W (Gen-1)). Utilizing this setup in flow and using otherwise identical conditions to the batch process, we successfully scaled up the reaction up to 8.5 mmol (85 ml of solvent mixture, DCE:HFIP (7:3)), the corresponding dehydrogenated product **3a** was obtained in 75% yield after 62 h under continuous-flow conditions (residence time: 50 min). This setup demonstrates the feasibility of safe, scalable operation under continuous flow conditions, supporting the synthetic applicability of our method.

5. Mechanistic investigations

5.1. Radical scavenger experiment

An oven-dried 10 mL microwave vial equipped with a magnetic stirring bar was charged with TEMPO (1 or 2 equiv.), **1a** (0.1 mmol) and 1-methoxy-4-nitropyridin-1-ium tetrafluoroborate (**2h**, 1.5 equiv.). The reaction vessel was then subjected to three cycles of evacuation and backfilling with nitrogen atmosphere (3 minutes per cycle) using standard Schlenk techniques. Under nitrogen atmosphere, HFIP (0.3M) and DCE (0.7 M) were added. The reaction mixture was stirred at room temperature under Kessil lamp irradiation (λ_{max} = 390 nm). Lastly, the solvent was removed under reduced pressure, and the formation of the dehydrogenated product was detected using ^{1}H NMR

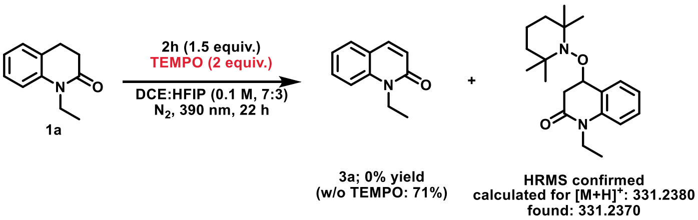

where 1,3,5-trimethylbenzene was used as the internal standard to calculate the yield. However, 28% and no desired product peaks were detected in the ^{1}H NMR spectra in case of 1 equiv. and 2 equiv. of TEMPO respectively. To trap the TEMPO adduct, we have performed the reaction with 2 equiv. of TEMPO by following the above-described procedure. The TEMPO adduct was confirmed by HRMS analysis using the crude reaction mixture.

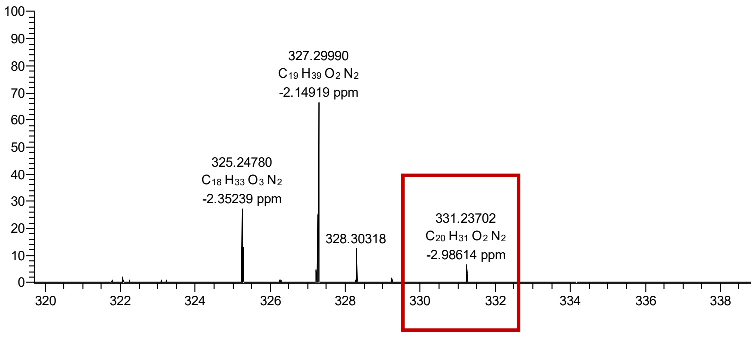

Furthermore, a quenching experiment with $CuCl_2$ was conducted using the above-described procedure. Here, $CuCl_2$ (2 equiv.) was used in the place of TEMPO. In ^{1}HNMR spectra analysis, no desired product was detected.

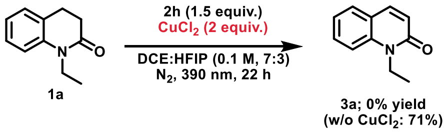

5.2. KIE experiment

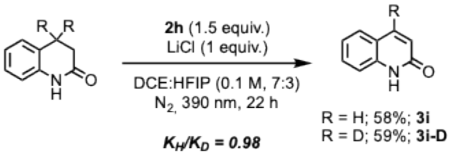

General procedure B is followed for benzylic CH bond KIE experiment. A solution of dideuterated (benzylic-D_2) derivatives of **1i** (0.1 mmol), LiCl (1 equiv.) and 1-methoxy-4-nitropyridin-1-ium tetrafluoroborate (**2h**, 1.5 equiv.) were put under nitrogen atmosphere using Schlenk techniques. Under nitrogen atmosphere, HFIP (0.3M) and DCE (0.7 M) were added. The reaction was stirred for 22 hours, before water was added. The mixture was then analysed by using ^{1}H NMR where 1,3,5-trimethylbenzene was used as the internal standard to calculate the yield.

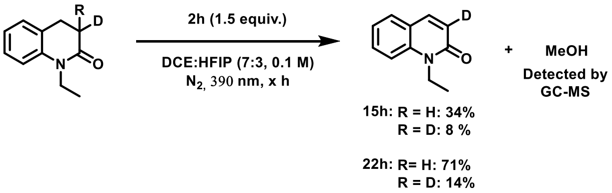

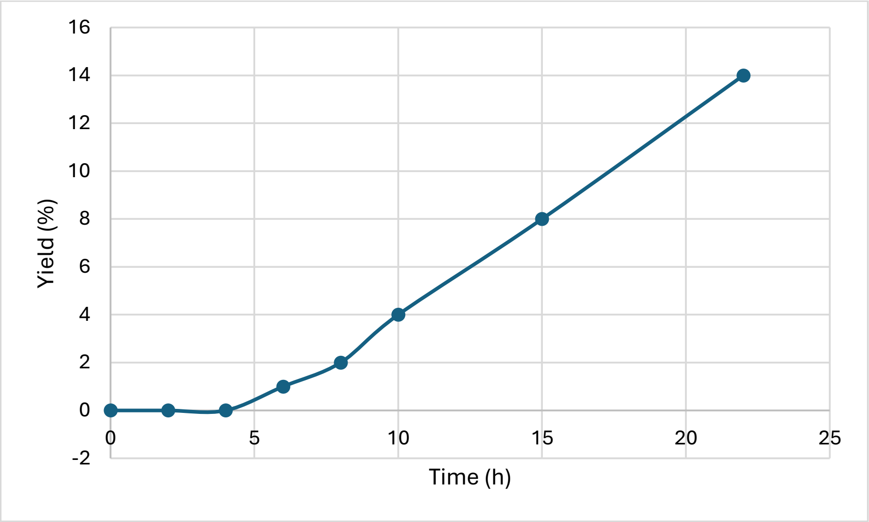

General procedure A is followed for α-CH bond KIE experiment. A solution of non-deuterated, monodeuterated (C3-D), and dideuterated (C3-D_2) derivatives of 1a (0.1 mmol) and 1-methoxy-4-nitropyridin-1-ium tetrafluoroborate (**2h**, 1.5 equiv.) were put under nitrogen atmosphere using Schlenck techniques. Under nitrogen atmosphere, HFIP (0.3M) and DCE (0.7 M) were added. The reaction was stirred for 15 hours, before water was added. The mixture was then analysed by using ^{1}H NMR where 1,3,5-trimethylbenzene was used as the internal standard to calculate the yield.

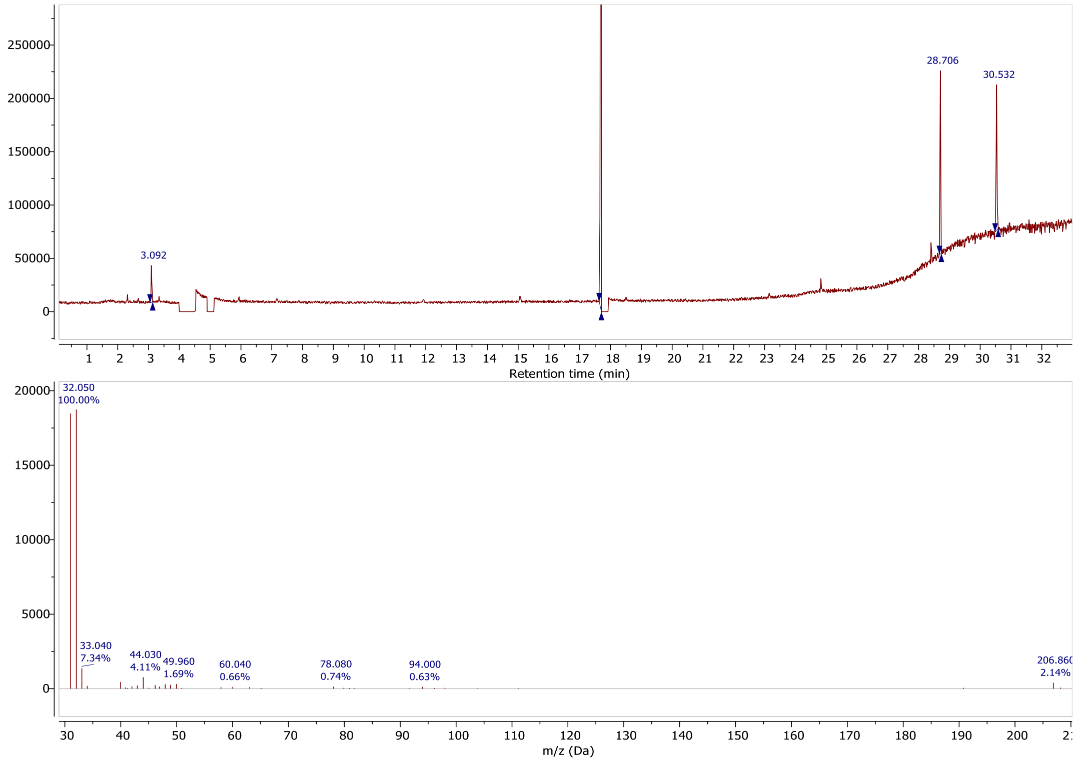

Figure S5: GC-MS spectrum for the detection of methanol.

5.3. On-off experiment

A solution of **1a** (0.1 mmol) and 1-methoxy-4-nitropyridin-1-ium tetrafluoroborate (**2h**, 1.5 equiv.) were put under nitrogen atmosphere using Schlenk techniques. Under nitrogen atmosphere, HFIP (0.3M) and DCE (0.7 M) were added. The reaction mixture was stirred at room temperature under Kessil lamp irradiation (λ_{max} = 390 nm). The on-off experiment was conducted at the specified time. The reaction mixture was taken out and analysed using ^{1}HNMR with 1,3,5-trimethyoxybenzene as the internal standard to calculate the yield of the product.

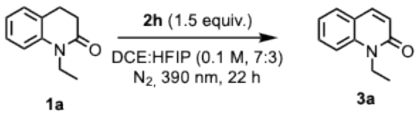

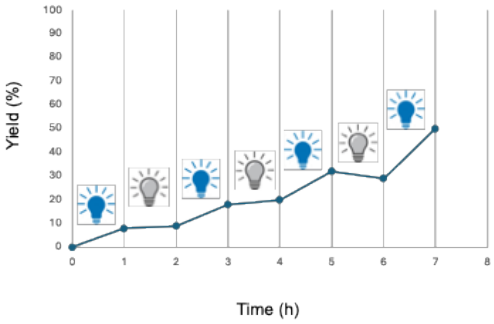

5.4. Electrochemical Measurements:

The electrochemical redox potentials were determined through the cyclic voltammetry study using a Metrohm Autolab potentiostat-galvanostat PGSTAT204 fitted with a glassy carbon (GC) as the working electrode (diameter = 3 mm), an Ag/AgCl (3 M KCl) as the reference electrode and a Pt-foil as the counter electrode, attached to a PC using Nova v2.1.5 software. 0.2 mmol samples were dissolved in 15 mL of 0.1 M Lithium perchlorate in dry and degassed acetonitrile:HFIP (7:3) solvent at room temperature (25 ± 2 °C) for the CV studies, whereas 0.1 M Lithium perchlorate in dry and degassed acetonitrile solution was considered as blank **(Figure S6)**. Reductions were measured by scanning potentials in the negative direction and oxidations in the positive direction. The obtained value was calculated with the reference of Ag/AgCl (3 M KCl).

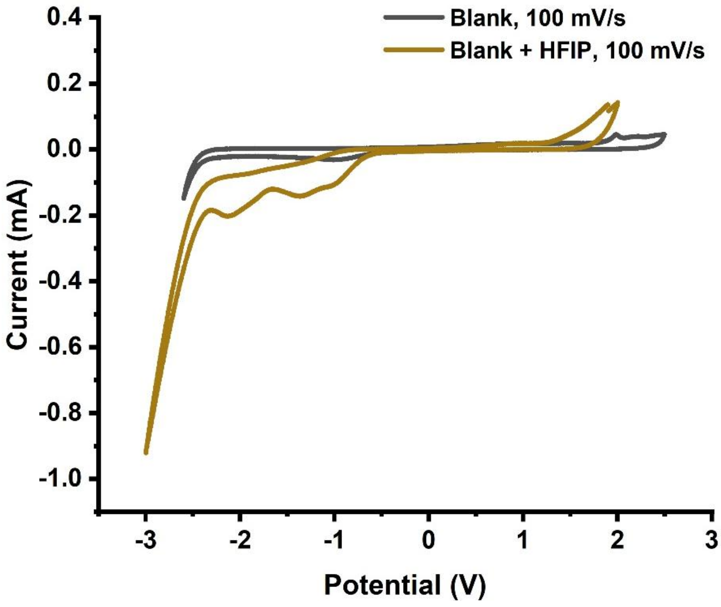

Figure S6: Cyclic Voltammogram of 0.1 M $LiClO_4$ in MeCN (Blank) and 0.1 M $LiClO_4$ in MeCN:HFIP (7:3) (Blank+HFIP)

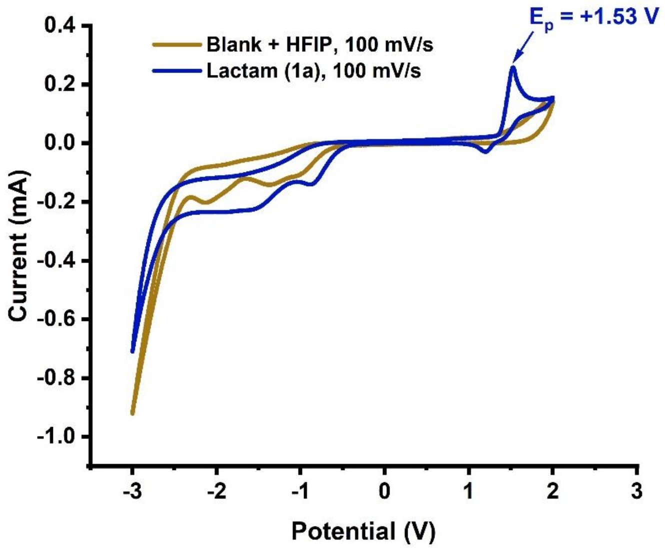

Figure S7: Cyclic Voltammogram of Lactam(1a)

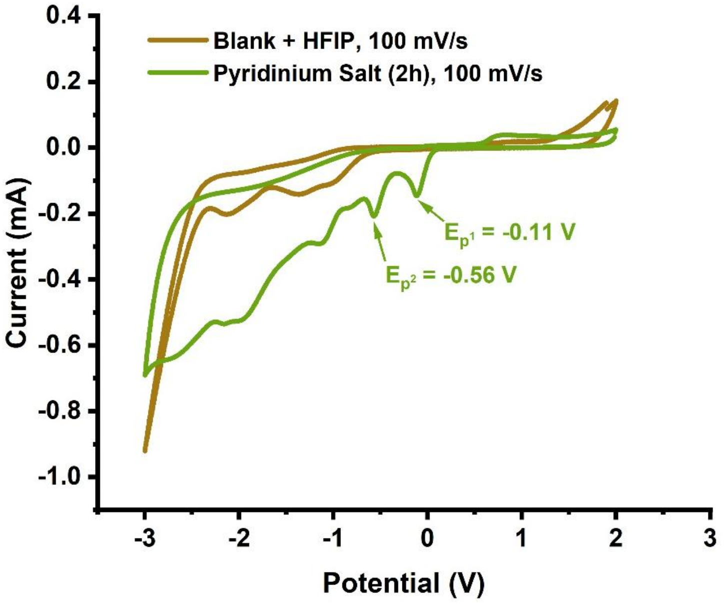

Figure S8: Cyclic Voltammogram of Pyridinium Salt(2h)

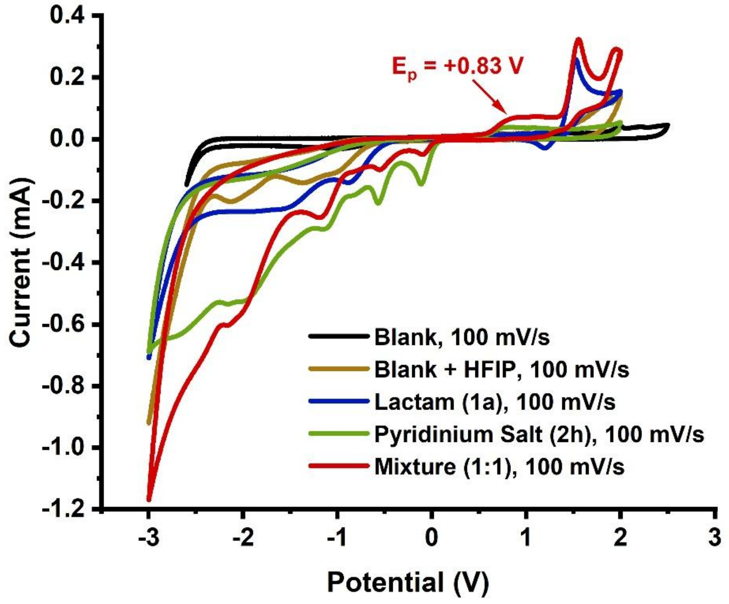

Figure S9: Cyclic Voltammogram of Lactam **(1a),** Pyridinium Salt **(2h)** and their mixture (1:1) at 100 mV/s scan rate

Initially the redox potentials of Lactam **(1a)** and Pyridinium Salt **(2h)** were measured under electrochemical condition. The peak potential (E_p) responsible for the oxidation of Lactam is +1.53 V vs. Ag/AgCl as shown in **Figure S7**, whereas the reduction of Pyridinium Salt exhibits two peak potentials (E_p^1 and E_p^2), observed at -0.11 V and -0.56 V vs. Ag/AgCl respectively, as presented in **Figure S8**. On the other hand, the cyclic voltammetry experiment of the 1:1 mixture of Lactam **(1a)** and Pyridinium Salt **(2h)** **(Figure S9)** delivers a new peak at +0.83 V which indicates the formation of a new species—an EDA adduct between Lactam **(1a)** and Pyridinium Salt **(2h)**. Therefore, the reaction proceeds through the photochemical oxidation of the EDA adduct formed between two substrates.

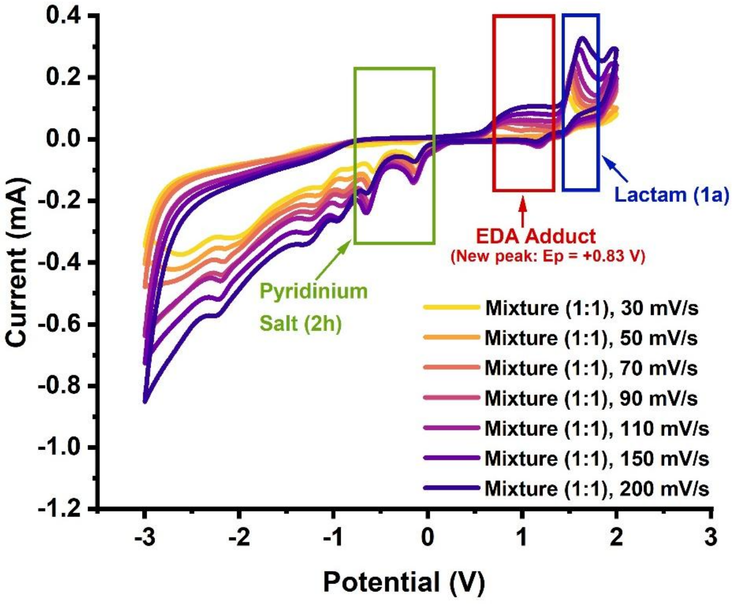

Figure S10: Cyclic Voltammogram of the mixture (1:1) of Lactam **(1a)** and Pyridinium Salt **(2h)** at different scan rates

As shown in **Figure S10**, the cyclic voltammetry of the mixture (1:1) of Lactam **(1a)** and Pyridinium Salt **(2h)** showed not only individual peaks of Lactam **(1a)** and Pyridinium Salt **(2h)** but also one new peak with lower oxidation potential at +0.83 V, which increased linearly with increasing the scan rate from 30 mV/s to 200 mV/s. Therefore, we proposed that this new peak was generated by the EDA adduct between Lactam **(1a)** and Pyridinium Salt **(2h)**.

5.5. NMR study

A solution of compound **8** (0.1 mmol) and varying equivalents (0.25, 0.5, and 0.75 equiv.) of 1-methoxy-4-nitropyridin-1-ium tetrafluoroborate (**2h**) was prepared under a nitrogen atmosphere using standard Schlenk techniques. Under an inert atmosphere, HFIP (0.3 M) and DCE (0.7 M) were added to the mixture, which was then stirred at room temperature. Subsequently, ^{19}F NMR spectra were recorded from three reaction vials. As shown in **Figure S11**, the appearance of a new fluorine signal in both concentration-dependent and time-dependent ^{19}F NMR spectra supports the formation of an EDA complex between **8** and **2h**.

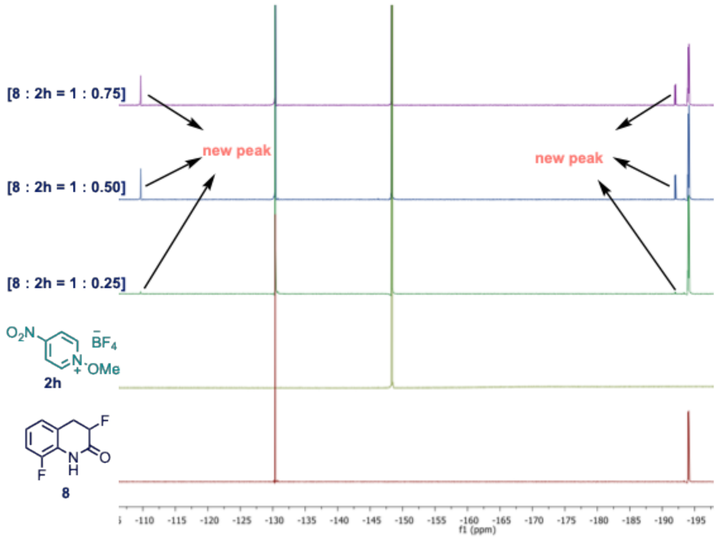

Figure S11: NMR study of **8** and **2h** mixture

5.6. UV-Vis analysis:

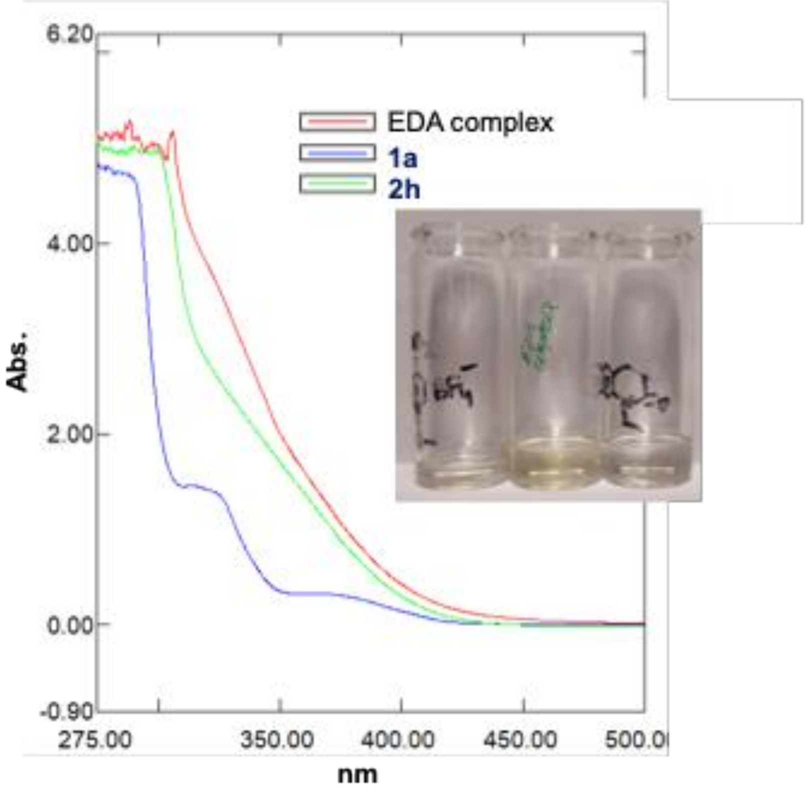

Figure S12: UV-Vis spectroscopy

6. Computational methods

6.1. TD-DFT Calculations

To identify the photoactive species and the productive transitions relevant under blue LED irradiation, we computed the absorption spectra of both individual reactants and the EDA complex. Although reactant 2h exhibited a transition at 404 nm, this corresponds to a non-productive lone pair (n) → π transition. In contrast, the EDA complex shows a π → π* transition at 394 nm, involving electron donation from the π-system of 1a to the π*-system of 2h. This transition falls within the emission range of the blue LED and confirms the EDA complex as the photoactive species.

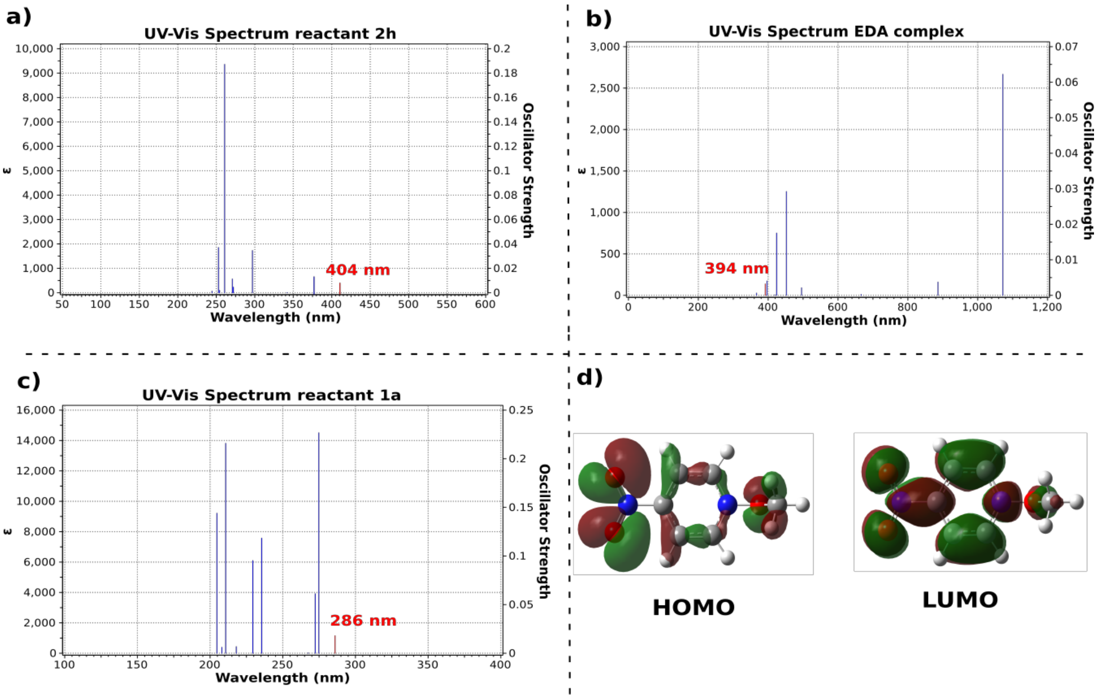

Figure S13: TD-DFT spectra for (a) reactant 2h; (b) EDA complex; (c) reactant 1a; (d) HOMO-LUMO orbitals related to the 404 nm transition for reactant 2h.

6.2. TD-DFT Transitions for the EDA Complex

Excited state 1: Energy: 1071.99 nm (Oscillator Strength:0.0622)
HOMO → LUMO (Coeff: 0.71);
HOMO-1 → LUMO (Coeff: -0.07);
HOMO-2 → LUMO+1 (Coeff: -0.02);
HOMO-3 → LUMO (Coeff: -0.01);
HOMO→ LUMO+1 (Coeff: -0.01)

Excited state 2: Energy: 886.27 nm (Oscillator Strength: 0.0037)
HOMO-1→LUMO (Coeff: 0.71);
HOMO → LUMO (Coeff: 0.07)

Excited state 3: Energy: 666.2 nm (Oscillator Strength: 0.0002)
HOMO-2 → LUMO (Coeff: 0.71);
HOMO-12 → LUMO (Coeff: 0.011)

Excited state 4: Energy: 495.71 nm (Oscillator Strength: 0.0021)
HOMO → LUMO+1 (Coeff: 0.71);
HOMO→ LUMO+2 (Coeff: 0.03);
HOMO → LUMO (Coeff: 0.01);
HOMO-2 → LUMO+1 (Coeff: -0.01)

Excited state 5: Energy: 452.2 nm (Oscillator Strength: 0.0292)
HOMO → LUMO+2 (Coeff: 0.70);
HOMO-4 → LUMO (Coeff: 0.04;
HOMO-13→ LUMO (Coeff: -0.04);
HOMO-3 → LUMO (Coeff: -0.03);

HOMO-12→ LUMO (Coeff: -0.03);
HOMO→ LUMO+1 (Coeff: -0.03);
HOMO → LUMO+4 (Coeff: 0.02);
HOMO-2→ LUMO+1 (Coeff: 0.02);
HOMO → LUMO+3 (Coeff: -0.01);
HOMO-14 → LUMO (Coeff: 0.01;
HOMO-1 → LUMO+2 (Coeff: -0.01);
HOMO-8 → LUMO+1 (Coeff: -0.01);
HOMO-2→ LUMO+3 (Coeff: -0.01);
HOMO-9 → LUMO+1 (Coeff: 0.01)

Excited state 6: Energy: 424.2 nm (Oscillator Strength: 0.0175)
HOMO-3 → LUMO (Coeff: 0.70);
HOMO-4 → LUMO (Coeff: 0.06);
HOMO → LUMO+3 (Coeff: -0.05);
HOMO-5 → LUMO (Coeff: 0.04);
HOMO → LUMO+4 (Coeff: 0.03);
HOMO-8→ LUMO (Coeff: 0.03);
HOMO-11→ LUMO (Coeff: -0.03);
HOMO→ LUMO+2 (Coeff: 0.03);
HOMO-12→ LUMO (Coeff: 0.02);
HOMO-7→ LUMO (Coeff: -0.02);
HOMO-13→ LUMO (Coeff: -0.02);
HOMO-1→ LUMO+1 (Coeff: -0.02);
HOMO-2→ LUMO+3 (Coeff: -0.02);
HOMO→ LUMO (Coeff: 0.01);
HOMO-2→ LUMO+1 (Coeff: -0.01);
HOMO-8→ LUMO+2 (Coeff: -0.01);
HOMO-9→ LUMO (Coeff: -0.01);
HOMO-9→ LUMO+2 (Coeff: 0.01);
HOMO-13→ LUMO+1 (Coeff: -0.01);
HOMO-2→ LUMO +4 (Coeff: -0.01)

Excited state 7: Energy: 419.02 nm (Oscillator Strength: 0.0001)
HOMO-1→ LUMO+1 (Coeff: 0.71);
HOMO-3 → LUMO (Coeff: 0.02)

Excited state 8: Energy: 396.51 nm (Oscillator Strength: 0.004)
HOMO-4 → LUMO (Coeff: 0.70);
HOMO-1→ LUMO+2 (Coeff: 0.11);
HOMO-3→ LUMO (Coeff: -0.06);
HOMO → LUMO+2 (Coeff: -0.04);
HOMO-6 → LUMO (Coeff: -0.04);
HOMO-5→ LUMO (Coeff: -0.03);
HOMO-4→ LUMO+2 (Coeff: -0.03);
HOMO-13→ LUMO (Coeff: 0.03);
HOMO-12→ LUMO (Coeff: 0.02);
HOMO-7→ LUMO (Coeff: -0.02);
HOMO-3→ LUMO+2 (Coeff: 0.02);
HOMO-2→ LUMO+1 (Coeff: -0.01);
HOMO-14→ LUMO (Coeff: -0.01)

Excited state 9: Energy: 394.96 nm (Oscillator Strength: 0.0005)
HOMO-1→ LUMO+2 (Coeff: 0.70);
HOMO-4→ LUMO (Coeff: -0.10);
HOMO→ LUMO+2 (Coeff: 0.02);
HOMO-13→ LUMO (Coeff: -0.01);
HOMO-5→ LUMO (Coeff: 0.01)

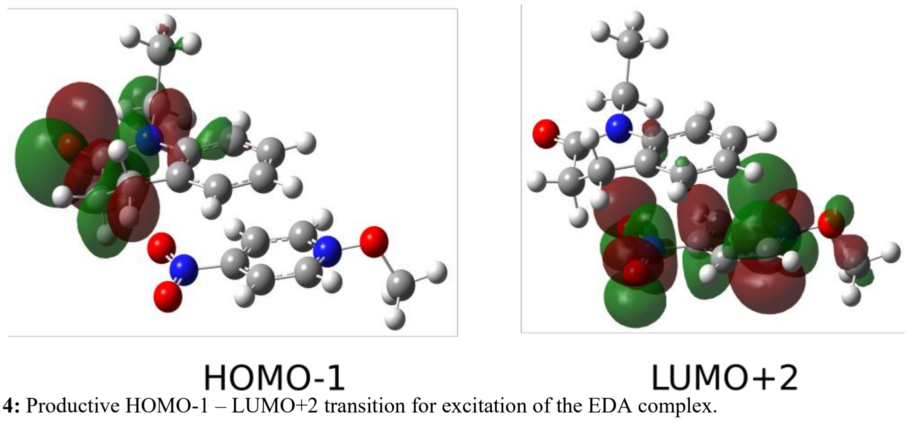

Figure S14: Productive HOMO-1 – LUMO+2 transition for excitation of the EDA complex.

6.3. Natural population analysis of the excited state

Natural Population Analysis (NPA) confirmed the charge-transfer nature of the complex, with 1a as the electron acceptor and 2h as the donor. This was also evident from the immediate colour change (to pale yellow) upon mixing the colourless reactants, indicating formation of a charge-transfer complex. As illustrated in **Figure S15** excitation from the singlet to the triplet state promotes charge separation, enabling the single-electron transfer (SET) process.

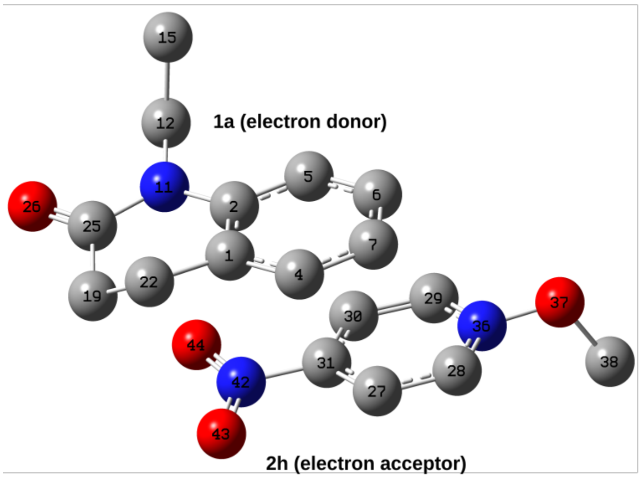

Calculated NPA charges:

Label	EDA complex singlet	EDA complex triplet	$\Delta_{triplet\text{-}singlet}$	
**C (1)**	**-0.039**	**0.096**	**0.135**	1a (electron donor)
C (2)	0.148	0.225	0.077	
C (4)	-0.2	-0.216	-0.016	
C (5)	-0.253	-0.205	0.048	
C (6)	-0.219	-0.184	0.035	
**C (7)**	**-0.23**	**-0.093**	**0.137**	
**N (11)**	**-0.406**	**-0.283**	**0.123**	
C (25)	0.648	0.722	0.074	
C (19)	-0.487	-0.505	-0.018	
C (22)	-0.413	-0.432	-0.019	
O (26)	-0.627	-0.537	0.09	
SUM	-2.078	-1.412	0.666	
C(29)	0.074	0.024	-0.05	2h (electron acceptor)
C (30)	-0.181	-0.235	-0.054	
C (31)	0.11	0.081	-0.029	
C (27)	-0.175	-0.232	-0.057	
C (28)	0.092	0.024	-0.068	
N (36)	0.024	-0.026	-0.05	
O (37)	-0.304	-0.32	-0.016	
C (38)	-0.275	-0.273	0.002	
**N (42)**	**0.501**	**0.375**	**-0.126**	
**O (44)**	**-0.343**	**-0.492**	**-0.149**	
**O (43)**	**-0.35**	**-0.492**	**-0.142**	
SUM	-0.827	-1.566	-0.739	
	**EDA complex total charge**		**1.405**	

Figure S15. Calculated NPA charges for the EDA complex at the ground-state singlet and ground-state triplet states.

6.4. Reactions and Plausible Mechanisms

We calculated the energy differences for the highest energy demanding step (after the photoexcitation) for the elucidated mechanism without HFIP (Scheme 4 (b)) with DCE and HFIP as solvents.

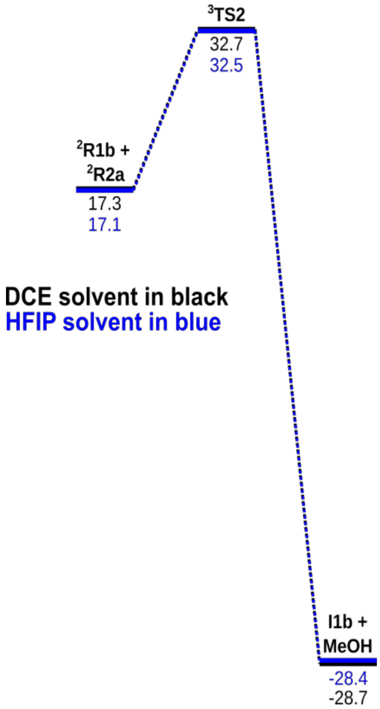

Figure S16. Energy profile for RDS using DCE and HFIP as solvents in kcal/mol.

To evaluate alternative mechanistic pathways, three possible side reactions were computed (**Figure S16**). The pathway highlighted in blue emerged as the most plausible. It closely resembles the mechanism in Scheme 4b, with the key difference being that methanol, rather than I1a, acts as the proton acceptor in the final step. This results in a slightly higher energy barrier, as methanol is less basic than I1a.

In the second pathway, HFIP serves as the proton acceptor. However, this route is also less favorable thermodynamically, as the protonated form of HFIP is significantly higher in energy than its neutral counterpart.

The third mechanism involves HFIP forming a radical intermediate (^{2}R4a), which would then undergo a single electron transfer (SET). However, this step is highly endergonic ($\Delta G = +134$ kcal/mol), rendering the pathway energetically inaccessible under the reaction conditions.

Having established Mechanism, I as the most favorable pathway between the three, we further investigated the competition between radicals ^{2}R3a and ^{2}R4a in the hydrogen atom transfer (HAT) step. Although ^{2}R3a is less thermodynamically stable, it exhibits a significantly lower activation barrier ($\Delta G^\ddagger = 4.8$ kcal/mol) compared to ^{2}R4a ($\Delta G^\ddagger = 47.6$ kcal/mol). This suggests that ^{2}R3a is the kinetically preferred species for the HAT process, further supporting its role in the productive reaction pathway.

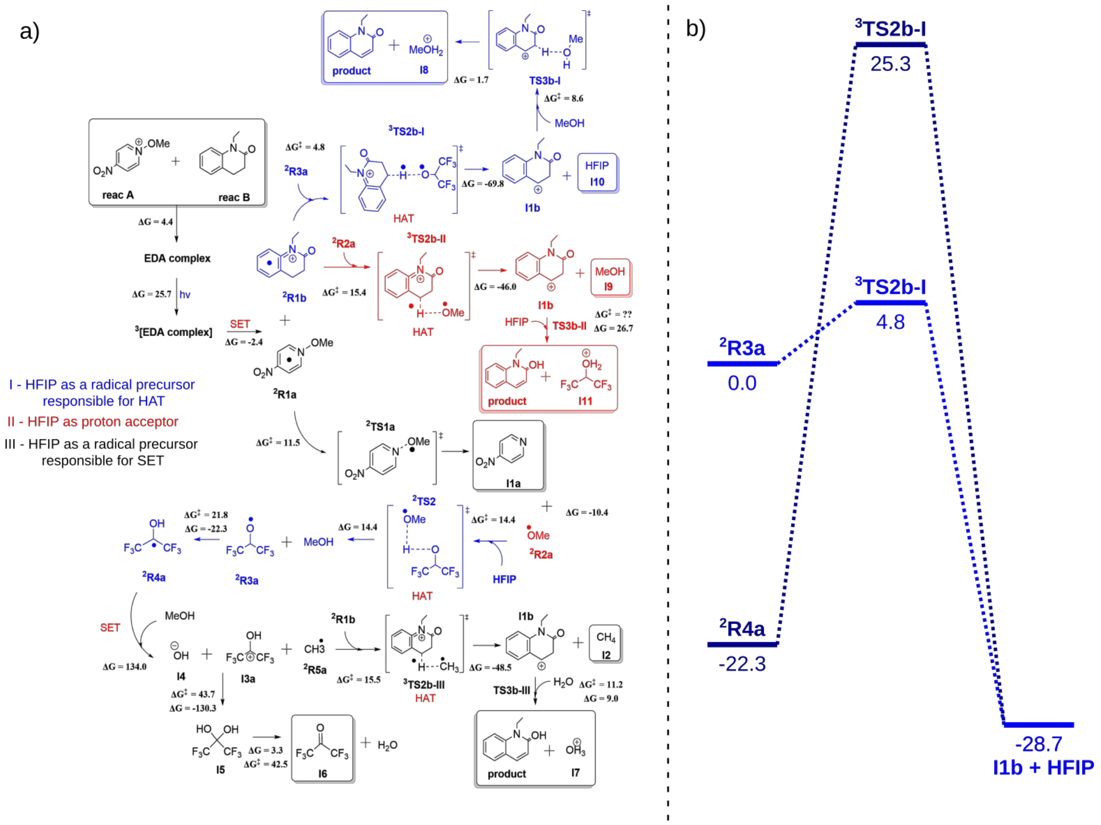

Figure S17: (a) Three possible reaction mechanisms computed for the dehydrogenation of reactant 1a; (b) Energy profile for the competition between ²R3a and ²R3b for the HAT. All energies are reported in kcal/mol.

6.5. Microkinetic Modelling

The rate constant for the photochemical step was adjusted to correspond to a ΔG‡ of 16.9 kcal/mol, allowing the model to reproduce the experimental reaction time for product 3a under the conditions of entry 10 (**Table 1**). Microkinetic simulations using this barrier yielded a theoretical product formation of 53%. Applying the same rate constant to a system using only DCE as the solvent resulted in a lower yield of 21%. This difference highlights the critical role of HFIP in the reaction: it not only accelerates the overall rate but also alters the mechanism by lowering the rate-determining step by 1 kcal/mol.

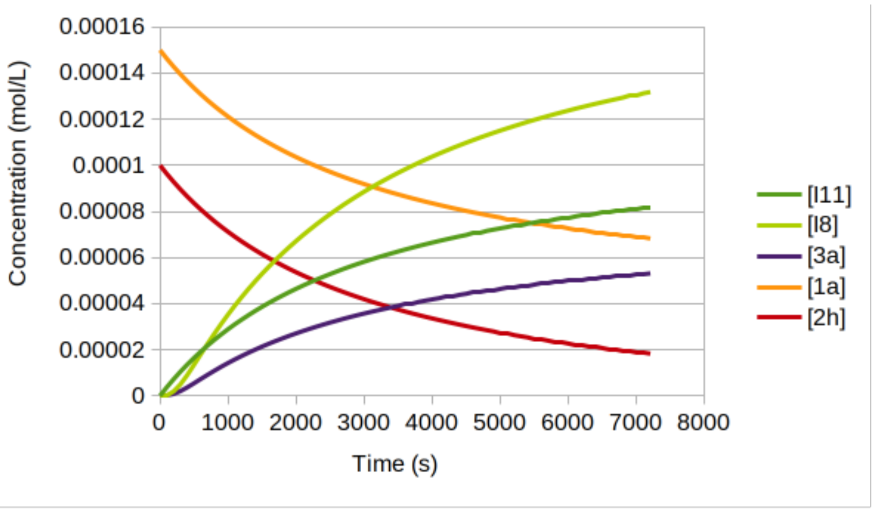

Figure S18: Microkinetic modeling for the dehydrogenation reaction between reactant 1a and 2h with HFIP.

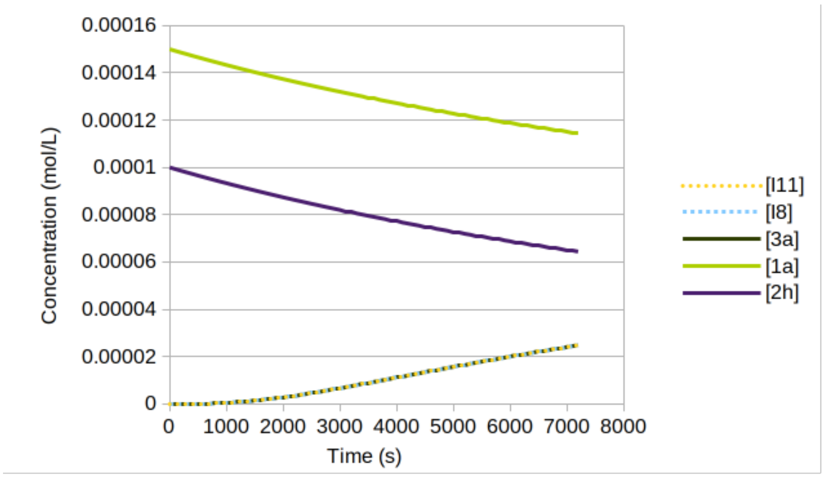

Figure S19: Microkinetic modeling for the dehydrogenation reaction between reactant 1a and 2h without HFIP in the mechanism.

7. Cartesian Coordinates in Ångstrom and potential energies in hartree

ts_methanol_protonation
Energy (POTENTIAL) = -671.515102491 Eh
Imaginary frequency = 1

Atom	X	Y	Z	H	1.2888	-2.3401	1.0333
C	-1.2844	1.0212	0.2347	H	2.8635	-2.6801	0.2703
C	-0.4246	-0.0623	-0.1624	H	2.6952	-1.2948	1.3795
H	-3.3116	1.6742	0.6158	C	0.6709	2.4736	0.3973
C	-2.6931	0.8273	0.3114	H	0.6905	3.0233	-0.7973
C	-1.0245	-1.3061	-0.4590	C	-0.7069	2.2542	0.5616
C	-2.4013	-1.4538	-0.3775	H	-1.3496	3.0714	0.9017
C	-3.2512	-0.3929	0.0068	H	1.1417	3.3066	0.9335
H	-0.4206	-2.1629	-0.7469	C	1.5703	1.3271	0.1362
H	-2.8358	-2.4275	-0.6165	O	2.7853	1.4231	0.1596
H	-4.3300	-0.5463	0.0623	O	0.9557	3.5706	-1.9843
N	0.9415	0.1247	-0.2339	C	2.2192	4.2759	-1.9613
C	1.8415	-0.9876	-0.5925	H	2.1160	5.0836	-1.2259
H	1.3726	-1.5572	-1.4043	H	3.0366	3.6016	-1.6677
H	2.7502	-0.5229	-0.9913	H	2.4070	4.7053	-2.9551
C	2.1859	-1.8756	0.5959	H	0.9886	2.8514	-2.6447

ts_methane_formation
Energy (POTENTIAL) = -596.263157045 Eh
Imaginary frequency = 1

Atom	X	Y	Z				
C	0.6215	0.6134	0.3064	H	-3.4520	0.6318	1.2662
C	-0.7583	0.6253	-0.1425	H	-4.7498	-0.4104	0.6213
H	2.2841	1.8330	0.8855	H	-3.4263	-1.1299	1.5768
C	1.2511	1.8316	0.5333	C	0.6904	-1.8377	-0.2047
C	-1.4095	1.8763	-0.3915	H	0.9009	-1.7678	-1.2893
C	-0.7414	3.0586	-0.1751	H	1.0640	-2.8051	0.1502
C	0.5904	3.0442	0.3015	C	1.3221	-0.6689	0.5147
H	-2.4374	1.8968	-0.7453	H	2.5081	-0.5272	0.0078
H	-1.2412	4.0092	-0.3672	H	1.5538	-0.8582	1.5736
H	1.1120	3.9869	0.4798	C	-0.8012	-1.8353	-0.0654
N	-1.4413	-0.5496	-0.2528	O	-1.4907	-2.8025	0.1340
C	-2.9023	-0.5887	-0.4619	C	3.7415	-0.4004	-0.6388
H	-3.1576	0.1550	-1.2256	H	3.5094	0.2399	-1.4980
H	-3.1292	-1.5797	-0.8672	H	4.3834	0.0598	0.1213
C	-3.6716	-0.3563	0.8347	H	3.9967	-1.4367	-0.8896

ts_methanol_formation
Energy (POTENTIAL) = -904.191725982 Eh
Imaginary frequency = 1

Atom	X	Y	Z	C	-1.2620	-1.2287	-0.0001
O	0.0286	2.6706	0.3441	F	-3.4504	1.7909	0.1157
C	-0.6234	3.8302	-0.0671	F	-3.8878	-0.1657	0.9379
H	-0.6019	3.9784	-1.1600	F	-3.6460	0.0849	-1.2016
H	-1.6679	3.8596	0.2995	F	0.0501	-1.3471	0.2351
H	-0.1048	4.6783	0.4232	F	-1.9128	-2.0096	0.8729
C	-3.1890	0.4807	-0.0058	F	-1.5018	-1.6991	-1.2309
C	-1.6769	0.2483	0.1371	O	-1.0011	0.9927	-0.7991
H	-1.4142	0.5354	1.1728	H	-0.1801	1.8356	-0.3188

2h
Energy (POTENTIAL) = -567.303294387 Eh
Imaginary frequency = 0

Atom	X	Y	Z	N	-3.9088	3.7433	-0.0475
C	-5.8972	2.4815	-0.0946	O	-2.5434	3.7699	-0.0282
C	-4.5132	2.5408	-0.1248	C	-1.9872	3.9354	-1.3581
C	-4.5647	4.9148	0.0747	H	-2.3086	4.8953	-1.7904
C	-5.9495	4.9086	0.1120	H	-0.9024	3.9322	-1.1979
C	-6.5947	3.6795	0.0208	H	-2.2834	3.0933	-2.0021
H	-6.4105	1.5230	-0.1567	N	-8.0802	3.6445	0.0515

H	-3.8598	1.6713	-0.2043	O	-8.6108	2.5667	-0.1352
H	-3.9500	5.8134	0.1407	O	-8.6533	4.6966	0.2585
H	-6.5034	5.8408	0.2131				

²R2a
Energy (POTENTIAL) = -114.968625228 Eh
Imaginary frequency = 0

Atom	X	Y	Z	H	-1.0354	0.5392	0.9135
O	0.7042	-0.1317	0.0012	H	-1.0336	0.5312	-0.9192
C	-0.6368	0.0467	-0.0003	H	-1.0821	-0.9828	0.0026

Water
Energy (POTENTIAL) = -76.3653786949 Eh
Imaginary frequency = 0

Atom	X	Y	Z	H	1.0505	-0.9685	0.0000
O	0.0829	-1.0268	0.0000	H	-0.1851	-0.0952	0.0000

²R1a
Energy (POTENTIAL) = -567.459661586 Eh
Imaginary frequency = 0

Atom	X	Y	Z	N	-1.5822	0.0055	-0.3066
C	0.4329	-1.2250	-0.1288	O	-2.9420	0.0079	-0.4887
C	-0.9268	-1.1985	-0.2671	C	-3.6427	-0.0052	0.7673
C	-0.9261	1.2080	-0.2389	H	-3.4021	0.8922	1.3599
C	0.4336	1.2305	-0.1001	H	-4.7078	-0.0024	0.5006
C	1.1499	0.0016	-0.0327	H	-3.4020	-0.9147	1.3411
H	0.9676	-2.1719	-0.0976	N	2.5439	-0.0006	0.1182
H	-1.5560	-2.0839	-0.3558	O	3.1383	-1.1007	0.1698
H	-1.5547	2.0956	-0.3070	O	3.1389	1.0978	0.1960
H	0.9688	2.1761	-0.0469				

ts_water_protonation
Energy (POTENTIAL) = -632.237964059 Eh
Imaginary frequency = 1

Atom	X	Y	Z	C	0.7543	3.1992	-0.4236
C	-0.9874	-0.9801	-0.4442	H	-0.2812	3.2470	-0.7941
C	-0.6941	0.3169	0.1004	H	1.0124	4.1825	-0.0009
H	-2.5086	-2.4434	-0.9121	H	1.4218	3.0070	-1.2771
C	-2.3283	-1.4518	-0.4914	C	1.3965	-1.3285	-0.8247
C	-1.7755	1.0960	0.5689	C	0.0706	-1.7546	-0.9468
C	-3.0700	0.6011	0.5087	H	-0.1493	-2.7280	-1.3952
C	-3.3627	-0.6753	-0.0192	H	2.1736	-1.7986	-1.4386
H	-1.6116	2.0912	0.9747	C	1.6924	0.0549	-0.3983
H	-3.8852	1.2268	0.8800	O	2.8084	0.5418	-0.4515
H	-4.3931	-1.0324	-0.0533	O	2.1025	-2.6074	1.3796
N	0.6093	0.7724	0.1448	H	1.6888	-2.1910	2.1644
C	0.9240	2.1241	0.6425	H	3.0686	-2.4758	1.4794
H	0.2974	2.3200	1.5211	H	1.7038	-2.0139	0.3291
H	1.9668	2.0876	0.9773				

²R4a
Energy (POTENTIAL) = -788.567431155 Eh
Imaginary frequency = 0

Atom	X	Y	Z	F	2.1708	0.3110	1.0640
C	1.3436	-0.1885	0.1309	F	-2.1899	0.8823	-0.3403
C	0.0793	0.5976	0.0465	F	-1.2717	-0.8771	-1.2264
C	-1.2485	-0.0626	-0.1526	F	-1.6166	-0.8084	0.8999
F	2.0323	-0.1832	-1.0301	O	0.2404	1.9048	-0.1690
F	1.0830	-1.4676	0.4396	H	-0.6227	2.3501	-0.2671

²R1b
Energy (POTENTIAL) = -556.464430567 Eh
Imaginary frequency = 0

Atom	X	Y	Z	H	-2.6632	1.3408	-0.7456
C	1.0841	-0.9650	0.2041	C	-1.7786	2.3249	0.9671
C	0.4194	0.2734	-0.1469	H	-0.7895	2.5877	1.3713
H	2.9732	-1.9125	0.5428	H	-2.3493	3.2535	0.8156
C	2.4695	-0.9801	0.2807	H	-2.3107	1.7089	1.7072
C	1.2032	1.4511	-0.3760	C	-1.0272	-2.1817	-0.2777
C	2.5733	1.3938	-0.2866	H	-0.7995	-2.3391	-1.3477
C	3.2190	0.1765	0.0387	H	-1.7144	-2.9748	0.0405
H	0.7216	2.3893	-0.6401	C	0.2620	-2.1673	0.5326
H	3.1667	2.2895	-0.4771	H	0.8443	-3.0833	0.3671
H	4.3085	0.1416	0.1023	H	0.0191	-2.1342	1.6113
N	-0.9395	0.3164	-0.2380	C	-1.7605	-0.8796	-0.1880
C	-1.6690	1.5938	-0.3665	O	-2.9568	-0.7578	-0.1314
H	-1.1630	2.2008	-1.1265				

I7
Energy (POTENTIAL) = -76.7660112128 Eh
Imaginary frequency = 0

Atom	X	Y	Z	H	0.8792	0.4668	0.2135
O	0.0079	0.0584	-0.0281	H	-0.7227	0.6845	0.2135
H	-0.0156	-0.1151	-1.0044				

I11
Energy (POTENTIAL) = -115.639029217 Eh
Imaginary frequency = 0

Atom	X	Y	Z	H	-4.7601	-0.1233	0.5695
O	-2.9666	-0.3118	-0.5194	H	-3.3526	-0.8074	1.4202
C	-3.6599	-0.0500	0.6806	H	-3.2387	0.3557	-1.1655
H	-3.4297	0.9459	1.1092				

I5
Energy (POTENTIAL) = -864.396921577 Eh
Imaginary frequency = 0

Atom	X	Y	Z	F	-2.3435	0.6853	-0.3280
C	1.2882	-0.1135	-0.0743	F	-1.4839	-1.2718	-0.6796
C	0.0006	0.6147	-0.5473	F	-1.2546	-0.3201	1.2511
C	-1.2901	-0.1043	-0.0680	O	0.0725	1.8772	0.0111
F	2.3443	0.4357	-0.6942	H	-0.7042	2.3882	-0.2788
F	1.2493	-1.4102	-0.3988	O	-0.0682	0.6022	-1.9276
F	1.4794	-0.0147	1.2404	H	0.7101	1.0691	-2.2806

^{2}R3a
Energy (POTENTIAL) = -788.545900029 Eh
Imaginary frequency = 0

Atom	X	Y	Z	F	2.3511	0.4658	0.6108
C	1.3059	-0.1907	0.1026	F	-2.3400	0.6728	0.2023
C	0.0110	0.6140	0.3636	F	-1.2894	-0.3957	-1.3596
C	-1.2914	-0.1123	-0.0503	F	-1.4495	-1.2575	0.6216
F	1.5136	-0.3473	-1.2109	O	0.0861	1.8540	-0.1578
F	1.2547	-1.4035	0.6631	H	-0.0532	0.7443	1.4735

ts_rad_HFIP_change
Energy (POTENTIAL) = -788.493143114 Eh
Imaginary frequency = 1

Atom	X	Y	Z	F	2.3302	0.5897	0.5007
C	1.3002	-0.1796	0.1467	F	-2.3528	0.5964	0.3287
C	-0.0050	0.5981	0.2596	F	-1.4139	-0.2586	-1.4232
C	-1.3033	-0.1141	-0.0912	F	-1.3645	-1.3312	0.4594
F	1.5010	-0.5847	-1.1209	O	0.0388	1.9310	0.0056
F	1.2951	-1.2673	0.9244	H	-0.0621	1.4986	1.1606

I6
Energy (POTENTIAL) = -787.991102106 Eh
Imaginary frequency = 0

Atom	X	Y	Z	F	0.7308	1.2914	0.4518

C	-0.2829	0.6663	-0.1321	F	-3.9197	1.5656	-0.3476
C	-1.5942	1.4727	0.0656	F	-2.8117	-0.0289	-1.3169
C	-2.9175	0.7086	-0.2059	F	-3.1863	-0.1070	0.8187
F	-0.0217	0.5420	-1.4372	O	-1.5854	2.6162	0.4070
F	-0.4035	-0.5579	0.3935				

3a
Energy (POTENTIAL) = -555.463761349 Eh
Imaginary frequency = 0

Atom	X	Y	Z	H	-5.2935	7.5270	2.1496
C	-2.9082	4.4071	3.0160	H	-6.7604	6.6213	2.5578
C	-3.6157	5.6403	2.9295	C	-5.8713	7.6218	4.2502
H	-0.9840	3.4384	2.9863	H	-4.8822	7.8833	4.6573
C	-1.5010	4.3990	2.9198	H	-6.4465	8.5528	4.1247
C	-2.8749	6.8319	2.7627	H	-6.3938	6.9937	4.9880
C	-1.4881	6.7918	2.6721	C	-5.0114	3.2307	3.3088
C	-0.7883	5.5760	2.7472	H	-5.6078	2.3296	3.4601
H	-3.3778	7.7952	2.7111	C	-3.6609	3.1982	3.2051
H	-0.9400	7.7284	2.5434	H	-3.1178	2.2512	3.2691
H	0.3011	5.5603	2.6746	C	-5.7615	4.4785	3.2349
N	-5.0042	5.6396	3.0114	O	-6.9844	4.5362	3.3529
C	-5.7612	6.8945	2.9153				

I10
Energy (POTENTIAL) = -789.225641705 Eh
Imaginary frequency = 0

Atom	X	Y	Z	F	-1.7688	-0.0893	-1.1435
C	-2.1437	-0.6782	-0.0004	F	0.3535	0.6870	2.3946
C	-1.5846	0.0465	1.2305	F	0.4562	-1.1817	1.3027
H	-1.9227	-0.5249	2.1107	F	0.4809	0.7033	0.2352
C	-0.0517	0.0593	1.2820	O	-2.0380	1.3662	1.2112
F	-3.4831	-0.6659	0.0432	H	-2.5775	1.5260	1.9997
F	-1.7462	-1.9581	-0.0478				

I4
Energy (POTENTIAL) = -75.7750520653 Eh
Imaginary frequency = 0

Atom	X	Y	Z
O	1.3797	1.1433	0.0000
H	2.3623	1.1433	0.0000

ts_HFIP_regeneration
Energy (POTENTIAL) = -1345.01529455 Eh
Imaginary frequency = 1

Atom	X	Y	Z	C	0.7095	-1.6706	-0.4731
C	1.1191	0.8113	-0.3736	H	0.3937	-1.8825	0.5659
C	2.4254	0.5329	0.1887	H	0.2848	-2.4515	-1.1139
H	-0.2791	2.3600	-0.8586	C	0.2774	-0.2841	-0.8796
C	0.6904	2.1315	-0.4171	H	-0.9420	-0.0736	-0.3120
C	3.1979	1.6057	0.7440	H	-0.0188	-0.1683	-1.9310
C	2.7216	2.8975	0.7091	C	2.2018	-1.8297	-0.4583
C	1.4723	3.1713	0.1153	O	2.8006	-2.8131	-0.8082
H	4.1744	1.4085	1.1798	C	-2.1188	0.2579	0.3132
H	3.3168	3.7083	1.1315	C	-3.0236	0.6596	-0.8394
H	1.1016	4.1975	0.0734	C	-2.5228	-1.0140	1.0494
N	2.9442	-0.7259	0.1108	O	-1.9283	1.3329	1.1325
C	4.3379	-1.0311	0.4925	F	-4.2476	1.0182	-0.4272
H	4.5607	-0.4903	1.4194	F	-3.1575	-0.3491	-1.7088
H	4.3663	-2.1026	0.7147	F	-2.4998	1.7071	-1.4961
C	5.3295	-0.6941	-0.6165	F	-1.6851	-1.1897	2.0927
H	5.3197	0.3792	-0.8588	F	-3.7675	-0.9576	1.5423
H	6.3414	-0.9640	-0.2783	F	-2.4396	-2.0916	0.2641
H	5.1043	-1.2665	-1.5278	H	-1.3970	1.0761	1.9078

triplet_EDA_complex
Energy (POTENTIAL) = -1123.95091422 Eh
Imaginary frequency = 0

Atom	X	Y	Z	H	3.7565	1.3771	-2.4811
C	2.5984	1.3098	-0.6878	H	4.5950	0.7224	-1.0687
C	1.8718	0.4385	0.2080	C	2.8944	-1.5423	-0.8488
H	2.9172	3.3376	-1.2999	O	3.2030	-2.6978	-0.7059
C	2.3682	2.6808	-0.6223	C	-1.9538	0.9825	-0.7067
C	0.9471	1.0089	1.1378	C	-3.0552	1.1634	0.0864
C	0.7484	2.3695	1.1695	C	-3.1437	-1.1797	0.5830
C	1.4586	3.2176	0.2880	C	-2.0454	-1.4159	-0.2009
H	0.3824	0.3698	1.8112	C	-1.4205	-0.3253	-0.8637
H	0.0302	2.7943	1.8729	H	-1.4837	1.8291	-1.2009
H	1.2912	4.2962	0.3205	H	-3.5257	2.1280	0.2765
N	2.0764	-0.9094	0.1681	H	-3.6792	-1.9499	1.1378
C	1.5211	-1.8119	1.1935	H	-1.6518	-2.4241	-0.3085
H	0.4783	-1.5283	1.3723	N	-3.6268	0.0900	0.7061
H	1.5214	-2.8109	0.7493	O	-4.6863	0.3087	1.5480
C	2.3427	-1.7974	2.4774	C	-5.9466	0.1846	0.8599
H	2.3549	-0.8014	2.9451	H	-6.0686	-0.8313	0.4519
H	1.9023	-2.5090	3.1924	H	-6.7067	0.3748	1.6284
H	3.3799	-2.1062	2.2794	H	-6.0272	0.9334	0.0557
C	3.2548	-0.6862	-2.0212	N	-0.2832	-0.5306	-1.6351
H	2.3497	-0.6546	-2.6499	O	0.2677	0.4639	-2.1920
H	4.0602	-1.1876	-2.5709	O	0.1939	-1.6974	-1.7298
C	3.6261	0.7301	-1.6031				

EDA_complex
Energy (POTENTIAL) = -1123.99518748 Eh
Imaginary frequency = 0

Atom	X	Y	Z	H	-0.1232	-2.5949	2.6408
C	0.4699	-0.6626	1.9025	H	-0.6482	-1.2242	3.6285
C	-0.0044	0.5291	1.3031	C	-2.3551	-0.1387	1.6041
H	2.1893	-1.8578	2.3650	O	-3.5429	0.1429	1.6217
C	1.8408	-0.9296	1.9043	C	2.0521	-1.3144	-1.4884
C	0.9271	1.4300	0.7504	C	3.2047	-0.6544	-1.8884
C	2.2946	1.1556	0.7877	C	1.9194	1.1078	-2.8471
C	2.7620	-0.0321	1.3555	C	0.7428	0.4994	-2.4537
H	0.5928	2.3566	0.2889	C	0.8322	-0.7142	-1.7743
H	2.9951	1.8731	0.3536	H	2.1161	-2.2598	-0.9538
H	3.8308	-0.2549	1.3802	H	4.2160	-1.0122	-1.6932
N	-1.3881	0.8111	1.2892	H	1.9764	2.0589	-3.3763
C	-1.8619	2.1531	0.9313	H	-0.2171	0.9675	-2.6653
H	-1.3675	2.4720	0.0027	N	3.0956	0.5116	-2.5510
H	-2.9292	2.0482	0.7103	O	4.2435	1.1832	-2.8641
C	-1.6575	3.1714	2.0468	C	4.7724	0.7884	-4.1549
H	-0.5956	3.2822	2.3158	H	4.0540	1.0302	-4.9526
H	-2.0324	4.1577	1.7299	H	5.6839	1.3879	-4.2671
H	-2.2102	2.8689	2.9500	H	5.0176	-0.2845	-4.1571
C	-1.8627	-1.5428	1.8745	N	-0.4103	-1.3788	-1.3327
H	-1.7939	-2.0513	0.8985	O	-0.3174	-2.5321	-0.9496
H	-2.6455	-2.0544	2.4491	O	-1.4385	-0.7280	-1.3830
C	-0.5167	-1.5697	2.5863				

^{2}R5a
Energy (POTENTIAL) = -39.8087122915 Eh
Imaginary frequency = 0

Atom	X	Y	Z	H	-0.9352	0.5734	-0.9488
C	-0.9586	0.0316	-0.0006	H	-0.9987	-1.0601	0.0037
H	-0.9386	0.5811	0.9432				

I1a
Energy (POTENTIAL) = -452.481261048 Eh
Imaginary frequency = 0

Atom	X	Y	Z	H	2.9904	0.5000	-1.8902
C	1.1125	0.8665	-0.8880	H	2.5319	-1.6704	1.6164
C	2.3624	0.2566	-1.0270	H	0.2595	-0.6777	2.0362
C	2.1085	-0.9449	0.9142	N	2.8509	-0.6264	-0.1507

C	0.8446	-0.4013	1.1603	N	-0.9676	1.1256	0.4344
C	0.3643	0.5145	0.2303	O	-1.3567	1.9253	-0.4003
H	0.7373	1.5835	-1.6168	O	-1.5965	0.7933	1.4252

ts_I1a_protonation
Energy (POTENTIAL) = -1008.36221752 Eh
Imaginary frequency = 1

Atom	X	Y	Z	C	-1.6516	1.0247	1.6972
C	-2.4354	1.3216	0.5856	H	-1.4376	1.8126	2.4254
C	-2.7536	0.2659	-0.3453	H	-0.8394	-0.5570	2.9028
H	-2.6842	3.4095	1.1043	C	-1.5768	-1.3777	1.0320
C	-2.9470	2.6385	0.3771	O	-1.3585	-2.5482	1.2786
C	-3.5883	0.5818	-1.4410	C	3.3254	-1.1004	-0.2205
C	-4.0617	1.8738	-1.6068	C	2.0659	-1.1356	0.3754
C	-3.7459	2.9171	-0.7046	C	2.2061	1.0966	1.0092
H	-3.8844	-0.1841	-2.1535	C	3.4762	1.2319	0.4478
H	-4.7079	2.0861	-2.4622	C	4.0145	0.1061	-0.1667
H	-4.1403	3.9210	-0.8685	H	3.7512	-1.9769	-0.7055
N	-2.2683	-1.0051	-0.1402	H	1.4716	-2.0515	0.3876
C	-2.5997	-2.1069	-1.0633	H	1.7123	1.9394	1.5012
H	-2.5630	-1.7126	-2.0871	H	4.0218	2.1738	0.4871
H	-1.7899	-2.8388	-0.9595	N	1.5393	-0.0600	0.9699
C	-3.9357	-2.7668	-0.7530	N	5.3626	0.1907	-0.7787
H	-4.7738	-2.0569	-0.8261	O	5.7478	-0.7692	-1.4223
H	-4.1168	-3.5827	-1.4699	O	5.9968	1.2142	-0.5955
H	-3.9281	-3.1961	0.2597	H	0.0804	-0.1058	1.4488
C	-1.0750	-0.2595	1.8723				

ts_methanol_formation
Energy (POTENTIAL) = -671.424977076 Eh
Imaginary frequency = 1

Atom	X	Y	Z	H	-3.4260	-1.2801	-0.2246
C	-0.1552	1.0833	1.0070	H	-3.3848	-2.8276	-1.1130
C	-0.8292	0.3380	-0.0363	H	-2.7024	-2.7317	0.5322
H	0.1502	3.0108	1.8748	C	1.3409	-0.8843	1.3717
C	-0.3803	2.4477	1.1058	H	2.0817	-0.6314	0.5900
C	-1.7161	1.0265	-0.9262	H	1.8379	-1.5026	2.1277
C	-1.9160	2.3818	-0.7946	C	0.7429	0.3747	1.9440
C	-1.2515	3.1031	0.2213	H	1.6950	1.1936	2.1895
H	-2.2347	0.4826	-1.7114	H	0.3045	0.2545	2.9478
H	-2.5877	2.8996	-1.4811	C	0.2981	-1.7273	0.6912
H	-1.4121	4.1788	0.3185	O	0.2364	-2.9273	0.7410
N	-0.6534	-1.0102	-0.1326	O	2.5066	2.0428	2.5529
C	-1.4755	-1.8472	-1.0308	C	2.7293	1.8751	3.9184
H	-1.5829	-1.3212	-1.9860	H	3.5058	2.6145	4.2000
H	-0.8998	-2.7598	-1.2128	H	1.8385	2.0905	4.5401
C	-2.8293	-2.1848	-0.4136	H	3.1254	0.8745	4.1791

I1b
Energy (POTENTIAL) = -555.867446908 Eh
Imaginary frequency = 0

Atom	X	Y	Z	H	-1.1445	2.0943	-1.1842
C	1.1846	-0.9502	0.1376	H	-2.6359	1.2224	-0.8083
C	0.4516	0.2849	-0.1151	C	-1.8743	2.3736	0.8556
H	3.1332	-1.8902	0.3460	H	-0.9184	2.6897	1.2997
C	2.6252	-0.9431	0.1537	H	-2.4481	3.2774	0.5992
C	1.2072	1.4601	-0.3239	H	-2.4427	1.8109	1.6111
C	2.5903	1.4122	-0.2989	C	-0.9683	-2.1540	0.3459
C	3.3180	0.2136	-0.0636	H	-1.2895	-2.8318	-0.4728
H	0.7174	2.4149	-0.4948	C	0.4939	-2.1102	0.3626
H	3.1424	2.3406	-0.4641	H	1.0397	-3.0384	0.5562
H	4.4085	0.2317	-0.0534	H	-1.3502	-2.6588	1.2527
N	-0.9142	0.2924	-0.1403	C	-1.6950	-0.8472	0.1650

C	-1.6698	1.5370	-0.3996	O	-2.8983	-0.7643	0.2519

I3a
Energy (POTENTIAL) = -788.340774976 Eh
Imaginary frequency = 0

Atom	X	Y	Z	F	1.4249	-0.8427	1.1318
C	1.3472	-0.1677	0.0024	F	-2.3462	0.7421	0.1222
C	-0.0001	0.6176	-0.0001	F	-1.4614	-0.7816	-1.1435
C	-1.3682	-0.1270	0.0030	F	-1.3680	-0.9738	1.0179
F	2.3344	0.7113	-0.0805	O	-0.0322	1.8545	-0.0080
F	1.3653	-0.9798	-1.0340	H	0.8564	2.3235	-0.0141

ts_formation_propanediol
Energy (POTENTIAL) = -864.041521860 Eh
Imaginary frequency = 1

Atom	X	Y	Z	F	-2.3423	-0.5028	-0.3422
C	1.3491	0.2555	0.0600	F	-1.3536	0.4350	1.3436
C	-0.0001	-0.3752	-0.4062	F	-1.4401	1.4564	-0.5730
C	-1.3407	0.2820	0.0247	O	-0.0554	-0.9472	-1.5431
F	2.3249	-0.5932	-0.2498	H	0.8191	-1.2162	-1.9247
F	1.3483	0.4917	1.3556	O	-0.0076	-1.7367	0.8928
F	1.5093	1.3874	-0.6086	H	-0.7833	-2.3578	0.7256

I8
Energy (POTENTIAL) = -452.925230132 Eh
Imaginary frequency = 0

Atom	X	Y	Z	H	3.3636	0.9084	-0.0072
C	5.3736	-2.3931	-0.0083	H	5.9115	0.9936	-0.0073
C	3.9871	-2.3603	-0.0083	N	3.3553	-1.1731	-0.0080
C	3.9871	0.0141	-0.0075	N	7.5313	-1.1731	-0.0083
C	5.3736	0.0469	-0.0076	O	8.0835	-2.2564	-0.0064
C	6.0424	-1.1731	-0.0080	O	8.0835	-0.0898	-0.0092
H	5.9115	-3.3398	-0.0085	H	2.3306	-1.1731	-0.0080
H	3.3636	-3.2546	-0.0086				

I8
Energy (POTENTIAL) = -116.047273498 Eh
Imaginary frequency = 0

Atom	X	Y	Z	H	-0.2384	2.7748	-0.5370
O	-1.7589	1.4437	-0.0903	H	-0.0160	1.1081	-1.2401
C	-0.7158	1.9272	-1.0392	H	-1.3906	1.1641	0.7827
H	-1.2621	2.2422	-1.9340	H	-2.2937	0.6944	-0.4489

ts_ketone_formation
Energy (POTENTIAL) = -864.321753430 Eh
Imaginary frequency = 1

Atom	X	Y	Z	F	2.2152	0.7776	0.1212
C	-1.3618	-0.3132	0.0923	F	1.4769	-1.0389	1.0492
C	-0.1209	0.5723	0.3916	F	1.2641	-0.7446	-1.0889
C	1.2341	-0.1343	0.1024	O	-0.2630	1.7811	-0.6126
F	-2.4737	0.3650	0.3711	H	0.5392	1.9827	-1.1452
F	-1.3235	-1.4052	0.8674	O	-0.1647	1.2790	1.4794
F	-1.4197	-0.7087	-1.1843	H	-0.2376	2.2150	0.4652

1a
Energy (POTENTIAL) = -556.674043057 Eh
Imaginary frequency = 0

Atom	X	Y	Z	H	-6.8614	6.6212	2.9144
C	-2.9368	4.4145	3.0299	C	-5.6268	7.4546	4.4810
C	-3.6608	5.6089	2.8095	H	-4.5729	7.6896	4.6962
H	-0.9979	3.4961	3.0973	H	-6.2059	8.3877	4.5689

C	-1.5436	4.4291	2.9308	H	-5.9862	6.7538	5.2512
C	-2.9593	6.7900	2.5126	C	-5.0229	3.1607	2.6018
C	-1.5646	6.7838	2.4275	H	-4.8205	3.0871	1.5177
C	-0.8472	5.6044	2.6332	H	-5.6701	2.3181	2.8774
H	-3.4937	7.7235	2.3433	C	-3.7104	3.1718	3.3760
H	-1.0415	7.7144	2.1939	H	-3.1162	2.2720	3.1615
H	0.2430	5.5971	2.5640	H	-3.9276	3.1617	4.4607
N	-5.0755	5.6000	2.9101	C	-5.8155	4.4306	2.8310
C	-5.8067	6.8591	3.0896	O	-7.0356	4.4283	2.9176
H	-5.4997	7.5724	2.3103				

ts_rad_OMe_release
Energy (POTENTIAL) = -567.437357890 Eh
Imaginary frequency = 1

Atom	X	Y	Z	N	1.6170	0.0014	-0.2010
C	-0.4285	1.2178	-0.0058	O	2.7213	0.0027	-1.5333
C	0.9449	1.1785	-0.1517	C	4.0133	0.0029	-1.0030
C	0.9461	-1.1763	-0.1529	H	4.2372	-0.8975	-0.3980
C	-0.4274	-1.2171	-0.0071	H	4.6994	0.0037	-1.8711
C	-1.1213	-0.0000	0.0667	H	4.2365	0.9026	-0.3968
H	-0.9583	2.1661	0.0707	N	-2.5622	-0.0008	0.2349
H	1.5492	2.0854	-0.2176	O	-3.1349	1.0828	0.3018
H	1.5512	-2.0825	-0.2198	O	-3.1339	-1.0851	0.3006
H	-0.9562	-2.1660	0.0684				

I2
Energy (POTENTIAL) = -40.4889438849 Eh
Imaginary frequency = 0

Atom	X	Y	Z	H	0.9035	-0.5099	-0.3665
C	0.0000	-0.0000	0.0004	H	-0.0101	1.0374	-0.3662
H	-0.8934	-0.5274	-0.3665	H	0.0000	-0.0002	1.1008

8. Characterisations

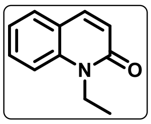

1-Ethylquinolin-2(1*H*)-one (3a):

Prepared according to the general procedure A. The product was purified by an automated flash chromatography system using a heptane/ethyl acetate gradient (from 30% ethyl acetate to 50% ethyl acetate) to yield yellow oil (yield 71%).

^{1}H NMR (400 MHz, $CDCl_3$) δ 7.64 (d, *J* = 9.4 Hz, 1H), 7.55 (ddd, *J* = 7.4, 4.3, 2.6 Hz, 2H), 7.37 (d, *J* = 8.9 Hz, 1H), 7.20 (td, *J* = 7.4, 1.0 Hz, 1H), 6.68 (d, *J* = 9.4 Hz, 1H), 4.35 (q, *J* = 7.2 Hz, 2H), 1.35 (t, *J* = 7.2 Hz, 3H); **^{13}C NMR** (101 MHz, $CDCl_3$) δ 161.9, 139.0, 138.9, 130.6, 129.0, 121.9, 121.8, 121.0, 114.0, 37.3, 12.7. **HRMS** (ESI) $[M+H]^+$ calculated for $[C_{11}H_{11}N_1O_1]$ requires m/z 174.0913, found m/z 174.0921.

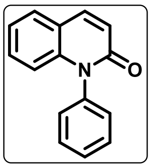

1-Phenylquinolin-2(1*H*)-one (3b):

Prepared according to the general procedure A. The product was purified by an automated flash chromatography system using a heptane/ethyl acetate gradient (from 30% ethyl acetate to 50% ethyl acetate) to yield brow solid (yield 71%).

^{1}H NMR (400 MHz, $CDCl_3$) δ 7.77 (d, *J* = 9.6 Hz, 1H), 7.62 – 7.55 (m, 3H), 7.54 – 7.47 (m, 1H), 7.35 – 7.24 (m, 3H), 7.18 (td, *J* = 7.5, 1.1 Hz, 1H), 6.78 (d, *J* = 9.6 Hz, 1H), 6.65 (d, *J* = 8.5 Hz, 1H); **^{13}C NMR** (101 MHz, $CDCl_3$) δ 162.3, 141.2, 139.8, 137.7, 130.2, 130.2, 128.9, 128.9, 128.3, 122.3, 122.3, 120.3, 116.0. **HRMS** (ESI) $[M+H]^+$ calculated for $[C_{15}H_{11}N_1O_1]$ requires m/z 222.0913, found m/z 222.0916.

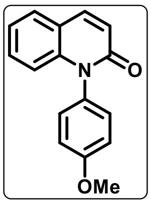

1-(4-Methoxyphenyl)quinolin-2(1*H*)-one (3c):

Prepared according to the general procedure A. The product was purified by an automated flash chromatography system using a heptane/ethyl acetate gradient (from 20% ethyl acetate to 60% ethyl acetate) to yield yellow solid (yield 61%).

^{1}H NMR (400 MHz, $CDCl_3$) δ 7.76 (d, *J* = 9.5 Hz, 1H), 7.57 (dd, *J* = 7.7, 1.6 Hz, 1H), 7.33 (ddd, *J* = 8.7, 7.2, 1.6 Hz, 1H), 7.21 – 7.15 (m, 3H), 7.12 – 7.07 (m, 2H), 6.77 (d, *J* = 9.6 Hz, 1H), 6.71 (d, *J* = 8.5 Hz, 1H), 3.88 (s, 3H); ^{13}C NMR (101 MHz, $CDCl_3$) δ 162.6, 159.7, 141.6, 139.7, 130.2, 129.8, 128.3, 122.2, 122.2, 120.4, 116.0, 115.5, 55.5. **HRMS** (ESI) $[M+H]^+$ calculated for $[C_{16}H_{13}N_1O_2]$ requires m/z 252.1019, found m/z 252.1010.

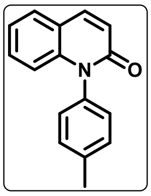

1-(P-tolyl)quinolin-2(1*H*)-one (3d):

Prepared according to the general procedure A. The product was purified by an automated flash chromatography system using a heptane/ethyl acetate gradient (from 30% ethyl acetate to 50% ethyl acetate) to yield yellow solid (yield 54%).

^{1}H NMR (400 MHz, CDCl3) δ δ 7.76 (d, J = 9.6 Hz, 1H), 7.57 (dd, J = 7.7, 1.5 Hz, 1H), 7.39 (d, J = 7.9 Hz, 2H), 7.32 (ddd, J = 8.6, 7.2, 1.6 Hz, 1H), 7.21 – 7.13 (m, 3H), 6.78 (d, J = 9.5 Hz, 1H), 6.69 (d, J = 8.5 Hz, 1H), 2.46 (s, 3H); **^{13}C NMR** (101 MHz, $CDCl_3$) δ 162.4, 141.3, 139.7, 138.9, 135.0, 130.9, 130.1, 128.5, 128.3, 122.3, 122.2, 120.4, 116.1, 21.3. **HRMS** (ESI) $[M+H]^+$ calculated for $[C_{16}H_{13}N_1O_1]$ requires m/z 236.1070, found m/z 236.1078.

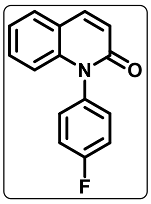

1-(4-Fluorophenyl)quinolin-2(1*H*)-one (3e):

Prepared according to the general procedure A. The product was purified by an automated flash chromatography system using a heptane/ethyl acetate gradient (from 30% ethyl acetate to 50% ethyl acetate) to yield yellow solid (yield 52%).

^{1}H NMR (400 MHz, $CDCl_3$) δ 7.78 (d, *J* = 9.6 Hz, 1H), 7.59 (dd, *J* = 7.7, 1.5 Hz, 1H), 7.35 (ddd, *J* = 8.7, 7.2, 1.6 Hz, 1H), 7.29 – 7.25 (m, 4H), 7.20 (td, *J* = 7.5, 1.1 Hz, 1H), 6.76 (d, *J* = 9.6 Hz, 1H), 6.66 (d, *J* = 8.5 Hz, 1H); **^{13}C NMR** (101 MHz, $CDCl_3$) δ **^{13}C NMR** (101 MHz, $CDCl_3$) δ 162.7(d, $J_{C\text{-}F}$= 249.6 HZ), 162.3, 141.2, 140.0, 133.5 (d, $J_{C\text{-}F}$= 3.4 Hz), 130.7 (d, $J_{C\text{-}F}$ = 8.8 Hz), 130.3, 128.4, 122.5, 122.2, 120.4, 117.3 (d, $J_{C\text{-}F}$ = 22.9 Hz), 115.8. **^{19}F NMR.** δ -112.3 (p, *J* = 6.6 Hz). **HRMS** (ESI) $[M+H]^+$ calculated for $[C_{15}H_{10}N_1O_1F_1]$ requires m/z 240.0819, found m/z 240.0812.

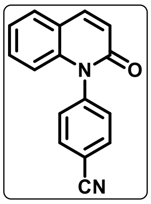

1-(4-Cyanophenyl)quinolin-2(1*H*)-one (3f):

Prepared according to the general procedure A. The product was purified by an automated flash chromatography system using a heptane/ethyl acetate gradient (from 40% ethyl acetate to 60% ethyl acetate) to yield yellow solid (yield 75%).

^{1}H NMR (400 MHz, $CDCl_3$) 7.91 – 7.89 (m, 2H), 7.82 (d, *J* = 9.6 Hz, 1H), 7.63 (d, *J* = 7.0 Hz, 1H), 7.45 (d, *J* = 8.3 Hz, 2H), 7.39 – 7.35 (m, 1H), 7.25 (t, *J* = 7.5 Hz, 1H), 6.76 (d, *J* = 9.6 Hz, 1H), 6.59 (d, *J* = 8.5 Hz, 1H).; **^{13}C NMR** (101 MHz, $CDCl_3$) δ. 141.9, 140.5, 140.3, 134.1, 130.6, 130.4, 128.8, 123.0, 121.9, 120.4, 118.0, 115.3, 113.1. **HRMS** (ESI) $[M+H]^+$ calculated for $[C_{16}H_{10}N_2O]$ requires m/z 247.0866, found m/z 247.0866.

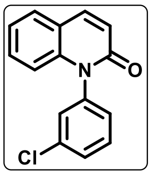

1-(3-Chlorophenyl)quinolin-2(1*H*)-one (3g):
Prepared according to the general procedure A. The product was purified by an automated flash chromatography system using a heptane/ethyl acetate gradient (from 30% ethyl acetate to 50% ethyl acetate) to yield yellow solid (yield 68%).
^{1}H NMR (400 MHz, $CDCl_3$) δ 7.78 (d, *J* = 9.5 Hz, 1H), 7.59 (dd, *J* = 7.8, 1.5 Hz, 1H), 7.56 – 7.48 (m, 2H), 7.36 (ddd, *J* = 8.7, 7.2, 1.5 Hz, 1H), 7.31 (t, *J* = 1.9 Hz, 1H), 7.21 (td, *J* = 7.6, 1.3 Hz, 2H), 6.76 (d, *J* = 9.6 Hz, 1H), 6.65 (d, *J* = 8.4 Hz, 1H); **^{13}C NMR** (101 MHz, $CDCl_3$) δ 162.1, 140.8, 140.1, 138.8, 135.7, 131.2, 130.4, 129.4, 129.3, 128.5, 127.3, 122.6, 122.1, 120.3, 115.7. **HRMS** (ESI) $[M+H]^+$ calculated for $[C_{15}H_{10}N_1O_1Cl_1]$ requires m/z 256.0524, found m/z 256.0524.

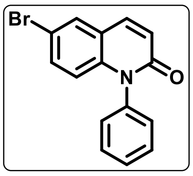

6-Bromo-1-phenylquinolin-2(1*H*)-one (3h):
Prepared according to the general procedure . The product was purified by an automated flash chromatography system using a heptane/ethyl acetate gradient (from 30% ethyl acetate to 50% ethyl acetate) to yield brown solid (yield 56%).
^{1}H NMR (400 MHz, $CDCl_3$) δ 7.71 (d, *J* = 2.2 Hz, 1H), 7.69 (d, *J* = 9.6 Hz, 1H), 7.60 (dd, *J* = 8.3, 6.6 Hz, 2H), 7.56 – 7.49 (m, 1H), 7.39 (dd, *J* = 9.0, 2.2 Hz, 1H), 7.27 (d, *J* = 1.5 Hz, 1H), 7.25 (d, *J* = 1.6 Hz, 1H), 6.80 (d, *J* = 9.5 Hz, 1H), 6.53 (d, *J* = 9.0 Hz, 1H); **^{13}C NMR** (101 MHz, $CDCl_3$) δ 161.8, 140.2, 138.5, 137.3, 132.9, 130.4, 130.3, 129.2, 128.7, 123.5, 121.7, 117.7, 115.1. **HRMS** (ESI) $[M+H]^+$ calculated for $[C_{15}H_{10}N_1O_1Br_1]$ requires m/z 300.0019, found m/z 300.0033.

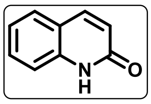

Quinolin-2(1*H*)-one (3i):
Prepared according to the general procedure **B**. The product was purified by an automated flash chromatography system using a heptane/ethyl acetate gradient (from 30% ethyl acetate to 50% ethyl acetate) to yield a yellow solid (yield 58%).
^{1}H NMR (400 MHz, $CDCl_3$) δ 12.43 (s, 1H), 7.82 (d, *J* = 9.5 Hz, 1H), 7.54 (ddd, *J* = 24.0, 7.4, 1.4 Hz, 2H), 7.47 – 7.42 (m, 1H), 7.22 (ddd, *J* = 8.1, 7.1, 1.3 Hz, 1H), 6.73 (d, *J* = 9.5 Hz, 1H); **^{13}C NMR** (101 MHz, $CDCl_3$) δ 164.7, 141.1, 138.6, 130.7, 127.8, 122.7, 121.5, 120.0, 116.3. **HRMS** (ESI) $[M+3H]^+$ calculated for $[C_9H_7N_1O_1]$ requires m/z 148.0757, found m/z 148.0751.

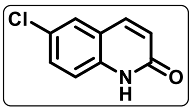

6-Chloroquinolin-2(1*H*)-one (3j):
Prepared according to the general procedure B. The product was purified by an automated flash chromatography system using a heptane/ethyl acetate gradient (from 30% ethyl acetate to 50% ethyl acetate) to yield yellow solid (yield 55%).
^{1}H NMR (400 MHz, DMSO) δ 11.84 (s, 1H), 7.88 (d, *J* = 9.6 Hz, 1H), 7.78 (d, *J* = 2.4 Hz, 1H), 7.53 (dd, *J* = 8.8, 2.4 Hz, 1H), 7.31 (d, *J* = 8.8 Hz, 1H), 6.56 (d, *J* = 9.6 Hz, 1H); **^{13}C NMR** (101 MHz, DMSO) δ 162.1, 139.6, 138.1, 130.6, 127.3, 126.0, 123.7, 120.7, 117.4. **HRMS** (ESI) $[M+H]^+$ calculated for $[C_9H_6N_1O_1Cl_1]$ requires m/z 180.0211, found m/z 180.0216.

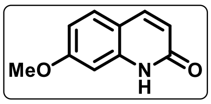

7-Methoxyquinolin-2(1*H*)-one (3k):
Prepared according to the general procedure B. The product was purified by an automated flash chromatography system using a heptane/ethyl acetate gradient (from 30% ethyl acetate to 50% ethyl acetate) to yield yellow solid (yield 56%).
^{1}H NMR (400 MHz, DMSO) δ 11.57 (s, 1H), 7.80 (d, *J* = 9.5 Hz, 1H), 7.56 (d, *J* = 8.4 Hz, 1H), 6.83 – 6.77 (m, 2H), 6.30 (d, *J* = 9.5 Hz, 1H), 3.81 (s, 3H); **^{13}C NMR** (101 MHz, DMSO) δ 162.7, 161.5, 141.2, 140.5, 129.7, 119.0, 113.8, 111.01, 98.5, 55.8. **HRMS** (ESI) $[M+H]^+$ calculated for $[C_{10}H_9N_1O_2]$ requires m/z 176.0706, found m/z 176.0708.

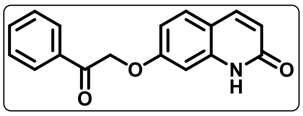

7-(2-Oxo-2-phenylethoxy)quinolin-2(1*H*)-one (3l):
Prepared according to the general procedure B. The product was purified by an automated flash chromatography system using a heptane/ethyl acetate gradient (from 30% ethyl acetate to 50% ethyl acetate) to yield yellow solid (yield 67%).
^{1}H NMR (400 MHz, DMSO) δ 11.48 (s, 1H), 8.09 – 8.02 (m, 2H), 7.81 (d, *J* = 9.5 Hz, 1H), 7.72 (t, *J* = 7.4 Hz, 1H), 7.65 – 7.55 (m, 3H), 6.87 (dd, *J* = 8.7, 2.5 Hz, 1H), 6.78 (d, *J* = 2.5 Hz, 1H), 6.31 (d, *J* = 9.5 Hz, 1H), 5.67 (s, 2H); **^{13}C NMR** (101 MHz, $CDCl_3$) δ 194.6, 162.6, 160.2, 140.9, 140.4, 134.8, 134.4, 129.7, 129.4, 128.3, 119.3, 114.1, 111.0, 99.8, 70.8. **HRMS** (ESI) $[M+H]^+$ calculated for $[C_{17}H_{13}N_1O_3]$ requires m/z 280.0968, found m/z 280.0975.

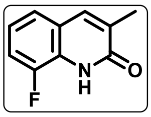

8-Fluoro-3-methylquinolin-2(*1H*)-one (3m):
Prepared according to the general procedure B. The product was purified by an automated flash chromatography system using a heptane/ethyl acetate gradient (from 10% ethyl acetate to 20% ethyl acetate) to yield white solid (38%).

^{1}H NMR (400 MHz, $CDCl_3$) δ 9.61 (s, 1H), 7.61 (p, *J* = 1.4 Hz, 1H), 7.27 (d, *J* = 7.5 Hz, 1H), 7.19 (ddd, *J* = 10.4, 8.1, 1.3 Hz, 1H), 7.10 (td, *J* = 8.0, 5.1 Hz, 1H), 2.28 (d, *J* = 1.3 Hz, 3H). **^{13}C NMR** (101 MHz, $CDCl_3$) δ **^{19}F NMR** (376 MHz, $CDCl_3$) δ -135.79 (dd, *J* = 10.7, 5.0 Hz). **^{13}C NMR** (101 MHz, $CDCl_3$) δ 162.6, 149.0 (d, *J* = 245.2 Hz), 136.4 (d, *J* = 2.9 Hz), 132.1, 126.3 (d, *J* = 13.2 Hz), 122.4 (d, J = 3.6 Hz), 122.0 (d, *J* = 7.0 Hz), 122.0 (d, *J* = 3.0 Hz), 114.2 (d, *J* = 17.1 Hz), 16.94. **HRMS** (ESI) $[M+H]^+$ calculated for [$C_{10}H_8NOF$] requires m/z 178.0663, found m/z 178.0675.

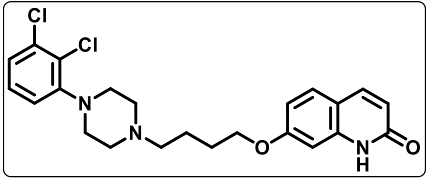

7-(4-(4-(2,3-dichlorophenyl)piperazin-1-yl)butoxy)quinolin-2(1H)-one (3n):
Prepared according to the general procedure B. The product was purified by an automated flash chromatography system using a heptane/ethyl acetate gradient (from 30% ethyl acetate to 50% ethyl acetate) to yield yellow solid (yield 57%).
^{1}H NMR (400 MHz, DMSO) δ 11.57 (s, 1H), 7.80 (d, *J* = 9.5 Hz, 1H), 7.55 (d, *J* = 8.7 Hz, 1H), 7.29 (d, *J* = 6.6 Hz, 2H), 7.12 (dd, *J* = 6.5, 3.1 Hz, 1H), 6.86 (d, *J* = 2.4 Hz, 1H), 6.79 (dd, *J* = 8.7, 2.4 Hz, 1H), 6.29 (d, *J* = 9.4 Hz, 1H), 4.04 (t, *J* = 6.3 Hz, 2H), 3.02 (s, 4H), 2.69 (s, 4H), 2.56 (s, 2H), 1.77 (dt, *J* = 12.0, 6.4 Hz, 2H), 1.66 (t, *J* = 7.5 Hz, 2H); **^{13}C NMR** (101 MHz, DMSO) δ 162.8, 160.9, 151.3, 141.1, 140.5, 133.1, 129.7, 129.0, 126.4, 125.0, 120.0, 118.9, 113.8, 111.4, 99.2, 68.0, 57.4, 52.9, 50.8, 26.9, 22.6. **HRMS** (ESI) $[M+H]^+$ calculated for [$C_{23}H_{25}N_3O_2Cl_2$] requires m/z 446.1397, found m/z 446.1411.

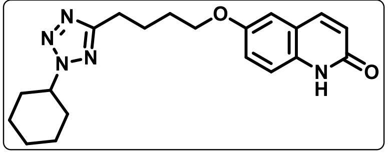

6-(4-(1-Cyclohexyl-1*H*-tetrazol-5-yl)butoxy)quinolin-2(1*H*)-one (3o):
Prepared according to the general procedure B. The product was purified by an automated flash chromatography system using a heptane/ethyl acetate gradient (from 30% ethyl acetate to 60% ethyl acetate) to yield yellow solid (yield 62%).
^{1}H NMR (400 MHz, $CDCl_3$) δ 12.59 (s, 1H), 7.73 (d, *J* = 9.5 Hz, 1H), 7.39 (d, *J* = 8.9 Hz, 1H), 7.11 (dd, *J* = 8.9, 2.7 Hz, 1H), 6.96 (d, *J* = 2.7 Hz, 1H), 6.71 (d, *J* = 9.5 Hz, 1H), 4.12 (ddd, *J* = 15.2, 10.2, 4.9 Hz, 1H), 4.05 (t, *J* = 6.0 Hz, 2H), 2.93 (t, *J* = 7.5 Hz, 2H), 2.15 – 1.81 (m, 11H), 1.48 – 1.27 (m, 3H). **^{13}C NMR** (101 MHz, $CDCl_3$) δ 164.3, 154.5, 153.6, 140.6, 133.4, 121.9, 120.6, 117.7, 109.8, 67.8, 57.8, 33.0, 28.6, 25.5, 24.9, 24.1, 23.2. **HRMS** (ESI) $[M+H]^+$ calculated for [$C_{20}H_{25}N_5O_2$] requires m/z 368.2081, found m/z 368.2131.

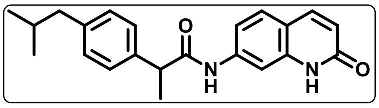

2-(4-Isobutylphenyl)-*N*-(2-oxo-1,2-dihydroquinolin-7-yl)propenamide (3p):
Prepared according to the general procedure A. The product was purified by an automated flash chromatography system using a heptane/ethyl acetate gradient (from 40% ethyl acetate to 60% ethyl acetate) to yield yellow solid (yield 67%).
^{1}H NMR (400 MHz, DMSO) δ 11.67 (s, 1H), 10.28 (s, 1H), 7.86 – 7.66 (m, 2H), 7.54 (d, *J* = 8.6 Hz, 1H), 7.37 – 7.24 (m, 3H), 7.11 (d, *J* = 8.0 Hz, 2H), 6.34 (dd, *J* = 9.5, 1.4 Hz, 1H), 3.84 (q, *J* = 7.0 Hz, 1H), 2.40 (d, *J* = 7.1 Hz, 2H), 1.91 – 1.69 (m, 1H), 1.42 (d, *J* = 7.0 Hz, 3H), 0.84 (d, *J* = 6.6 Hz, 6H); **^{13}C NMR** (101 MHz, DMSO) δ 173.3, 162.7, 141.5, 140.3, 140.2, 140.0, 139.4, 129.4, 128.8, 127.4, 120.2, 115.5, 114.2, 104.8, 46.1, 44.7, 30.0, 22.6, 19.1. **HRMS** (ESI) $[M+H]^+$ calculated for [$C_{22}H_{24}N_2O_2$] requires m/z 349.1911, found m/z 349.1919.

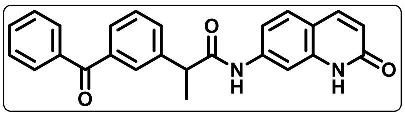

2-(3-benzoylphenyl)-*N*-(2-oxo-1,2-dihydroquinolin-7-yl)propenamide (3q):
Prepared according to the general procedure A. The product was purified by an automated flash chromatography system using a heptane/ethyl acetate gradient (from 30% ethyl acetate to 60% ethyl acetate) to yield yellow solid (yield 50%).
^{1}H NMR (400 MHz, DMSO) δ 11.68 (s, 1H), 10.39 (s, 1H), 7.84 (t, *J* = 1.8 Hz, 1H), 7.80 – 7.70 (m, 5H), 7.70 – 7.65 (m, 1H), 7.62 (dt, *J* = 7.7, 1.5 Hz, 1H), 7.58 – 7.50 (m, 4H), 7.31 (dd, *J* = 8.5, 2.0 Hz, 1H), 6.35 (dd, *J* = 9.4, 1.2 Hz, 1H), 4.00 (d, *J* = 7.0 Hz, 1H), 1.49 (d, *J* = 6.9 Hz, 3H); **^{13}C NMR** (101 MHz, DMSO) δ 196.1, 172.7, 162.7, 142.6, 141.3, 140.3, 140.2, 137.5, 133.1, 132.1, 130.1, 129.2, 129.0, 128.9, 128.8, 120.3, 115.7, 114.2, 104.9, 46.3, 19.1. **HRMS** (ESI) $[M+H]^+$ calculated for [$C_{25}H_{20}N_2O_3$] requires m/z 397.1547, found m/z 397.1542.

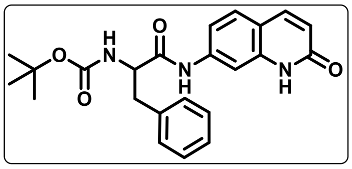

Tert-butyl (1-oxo-1-((2-oxo-1,2-dihydroquinolin-7-yl)amino)-3-phenylpropan-2-yl)carbamate (3r):
Prepared according to the general procedure B. The product was purified by an automated flash chromatography system using a heptane/ethyl acetate gradient (from 30% ethyl acetate to 50% ethyl acetate) to yield yellow solid (yield 81%).
^{1}H NMR (400 MHz, DMSO) δ 11.71 (d, *J* = 2.0 Hz, 1H), 10.29 (s, 1H), 7.89 – 7.69 (m, 2H), 7.57 (d, *J* = 8.5 Hz, 1H), 7.38 – 7.25 (m, 5H), 7.20 (t, *J* = 7.1 Hz,

1H), 7.13 (s, 1H), 6.36 (dd, *J* = 9.4, 1.7 Hz, 1H), 4.37 (td, *J* = 9.2, 4.8 Hz, 1H), 3.08 – 2.77 (m, 2H), 1.33 (s, 9H); **^{13}C NMR** (101 MHz, DMSO) δ 171.7, 162.7, 155.9, 141.2, 140.3, 140.2, 138.3, 129.7, 128.8, 128.5, 126.8, 120.3, 115.6, 114.3, 105.0, 78.6, 57.1, 37.8, 28.6. **HRMS** (ESI) $[M+H]^+$ calculated for $[C_{23}H_{25}N_3O_4]$ requires m/z 408.1918, found m/z 408.1911.

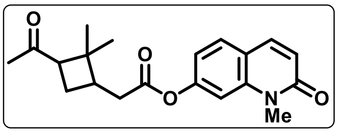

1-Methyl-2-oxo-1,2-dihydroquinolin-7-yl 2-(3-acetyl-2,2-dimethylcyclobutyl)acetate (3s):
Prepared according to the general procedure A. The product was purified by an automated flash chromatography system using a heptane/ethyl acetate gradient (from 20% ethyl acetate to 40% ethyl acetate) to yield yellow solid (yield 37%). **^{1}H NMR** (400 MHz, $CDCl_3$) δ 7.63 (d, *J* = 9.5 Hz, 1H), 7.55 (t, *J* = 8.2 Hz, 1H), 7.07 (d, *J* = 1.9 Hz, 1H), 6.94 (dd, *J* = 8.4, 2.1 Hz, 1H), 6.66 (dd, *J* = 11.0, 6.1 Hz, 1H), 3.65 (d, *J* = 8.7 Hz, 3H), 2.94 (dd, *J* = 10.1, 7.6 Hz, 1H), 2.80 – 2.43 (m, 3H), 2.17 – 1.97 (m, 5H), 1.38 (s, 3H), 0.95 (s, 3H). **^{13}C NMR** (101 MHz, $CDCl_3$) δ 207.4, 171.1, 162.4, 152.5, 141.2, 138.5, 129.9, 121.4, 118.6, 116.1, 107.4, 54.3, 43.4, 38.1, 35.3, 30.4, 30.3, 29.7, 23.1, 17.6. **HRMS** (ESI) $[M+H]^+$ calculated for $[C_{20}H_{23}NO_4]$ requires m/z 342.1700, found m/z 342.1691.

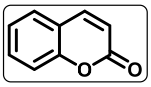

2*H*-Chromen-2-one (5a):
Prepared according to the general procedure A. The product was purified by an automated flash chromatography system using a heptane/ethyl acetate gradient (from 5% ethyl acetate to 20% ethyl acetate) to yield yellow solid (yield 49%).
^{1}H NMR (400 MHz, $CDCl_3$) δ 7.71 (d, *J* = 9.5 Hz, 1H), 7.53 (ddd, *J* = 8.3, 7.3, 1.3 Hz, 1H), 7.49 (dd, *J* = 7.7, 1.5 Hz, 1H), 7.34 (d, *J* = 8.3 Hz, 1H), 7.29 (d, *J* = 7.6 Hz, 1H), 6.43 (d, *J* = 9.5 Hz, 1H); **^{13}C NMR** (101 MHz, $CDCl_3$) δ 160.8, 154.1, 143.4, 131.8, 127.9, 124.4, 118.9, 116.9, 116.8. **HRMS** (ESI) $[M+H]^+$ calculated for $[C_9H_6O_2]$ requires m/z 147.0441, found m/z 147.0444.

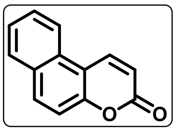

3*H*-Benzo[f]chromen-3-one (5b):
Prepared according to the general procedure A. The product was purified by an automated flash chromatography system using a heptane/ethyl acetate gradient (from 5% ethyl acetate to 20% ethyl acetate) to yield yellow solid (yield 30%).
^{1}H NMR (400 MHz, $CDCl_3$) δ 8.45 (d, *J* = 9.8 Hz, 1H), 8.20 (d, *J* = 8.4 Hz, 1H), 7.96 (d, *J* = 9.0 Hz, 1H), 7.89 (dd, *J* = 8.1, 1.3 Hz, 1H), 7.68 (ddd, *J* = 8.4, 6.9, 1.4 Hz, 1H), 7.56 (ddd, *J* = 8.1, 6.9, 1.1 Hz, 1H), 7.43 (d, *J* = 9.0 Hz, 1H), 6.55 (d, *J* = 9.8 Hz, 1H); **^{13}C NMR** (101 MHz, $CDCl_3$) δ 160.8, 153.9, 139.0, 133.1, 130.3, 129.0, 129.0, 128.3, 126.0, 121.3, 117.0, 115.6, 113.0. **HRMS** (ESI) $[M+H]^+$ calculated for $[C_{13}H_8O_2]$ requires m/z 197.0597, found m/z 197.0591.

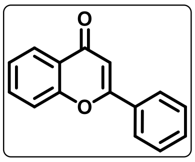

2-Phenyl-4*H*-chromen-4-one (7a):
Prepared according to the general procedure A. The product was purified by an automated flash chromatography system using a heptane/ethyl acetate gradient (from 30% ethyl acetate to 50% ethyl acetate) to yield yellow solid (yield 59%).
^{1}H NMR (400 MHz, $CDCl_3$) δ 8.23 (dd, *J* = 7.9, 1.7 Hz, 1H), 7.95 – 7.89 (m, 2H), 7.69 (ddd, *J* = 8.7, 7.1, 1.7 Hz, 1H), 7.59 – 7.48 (m, 4H), 7.41 (ddd, *J* = 8.1, 7.1, 1.1 Hz, 1H), 6.82 (s, 1H); **^{13}C NMR** (101 MHz, $CDCl_3$) δ 178.4, 163.4, 156.3, 133.8, 131.8, 131.6, 129.0, 126.3, 125.7, 125.2, 124.0, 118.1, 107.6. **HRMS** (ESI) $[M+H]^+$ calculated for $[C_{15}H_{10}O_2]$ requires m/z 223.0754, found m/z 223.0746.

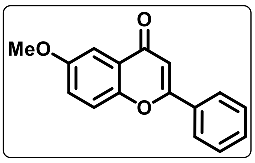

6-Methoxy-2-phenyl-4*H*-chromen-4-one (7b):
Prepared according to the general procedure A. The product was purified by an automated flash chromatography system using a heptane/ethyl acetate gradient (from 30% ethyl acetate to 50% ethyl acetate) to yield yellow solid (yield 74%).
^{1}H NMR (400 MHz, $CDCl_3$) δ 8.11 (d, *J* = 8.7 Hz, 1H), 7.91 – 7.83 (m, 2H), 7.50 (dt, *J* = 5.5, 2.8 Hz, 3H), 6.99 – 6.88 (m, 2H), 6.74 (s, 1H), 3.91 (s, 3H); **^{13}C NMR** (101 MHz, $CDCl_3$) δ 177.8, 164.2, 163.0, 158.0, 131.8, 131.4, 129.0, 127.0, 126.1, 117.8, 114.4, 107.5, 100.4, 55.8. **HRMS** (ESI) $[M+H]^+$ calculated for $[C_{16}H_{12}O_3]$ requires m/z 253.0859, found m/z 253.0852.

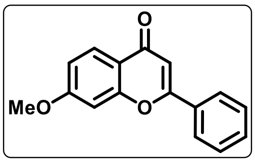

7-Methoxy-2-phenyl-4*H*-chromen-4-one (7c):
Prepared according to the general procedure A. The product was purified by an automated flash chromatography system using a heptane/ethyl acetate gradient (from 10% ethyl acetate to 30% ethyl acetate) to yield yellow solid (yield 35%).
^{1}H NMR (400 MHz, $CDCl_3$) δ 7.94 – 7.88 (m, 2H), 7.60 (d, *J* = 3.1 Hz, 1H), 7.55 – 7.48 (m, 4H), 7.29 (dd, *J* = 9.1, 3.1 Hz, 1H), 6.82 (s, 1H), 3.91 (s, 3H); **^{13}C NMR** (101 MHz, $CDCl_3$) δ 178.3, 163.2, 157.1, 151.1, 131.9, 131.5, 129.0, 126.3, 124.6, 123.8, 119.5, 106.9, 104.9, 56.0.**HRMS** (ESI) $[M+H]^+$ calculated for $[C_{16}H_{12}O_3]$ requires m/z 253.0859, found m/z 253.0865.

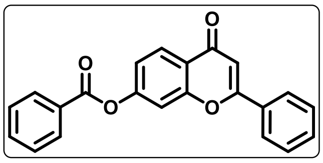

4-Oxo-2-phenyl-4*H*-chromen-7-yl benzoate (7d):

Prepared according to the general procedure A. The product was purified by an automated flash chromatography system using a heptane/ethyl acetate gradient (from 30% ethyl acetate to 50% ethyl acetate) to yield yellow solid (yield 72%).

^{1}H NMR (400 MHz, $CDCl_3$) δ 8.29 (d, J = 8.7 Hz, 1H), 8.24 – 8.19 (m, 2H), 7.92 – 7.87 (m, 2H), 7.70 – 7.63 (m, 1H), 7.57 – 7.48 (m, 6H), 7.29 (dd, J = 8.7, 2.2 Hz, 1H), 6.82 (s, 1H); **^{13}C NMR** (101 MHz, $CDCl_3$) δ 177.7, 164.4, 163.7, 156.8, 155.0, 134.1, 131.7, 131.6, 130.3, 129.1, 128.8, 128.8, 127.2, 126.3, 121.8, 119.5, 111.3, 107.7. **HRMS** (ESI) $[M+H]^+$ calculated for $[C_{22}H_{14}O_4]$ requires m/z 343.0965, found m/z 343.0979.

9. NMR spectra

1-Ethyl-3,4-dihydroquinolin-2(1H)-one

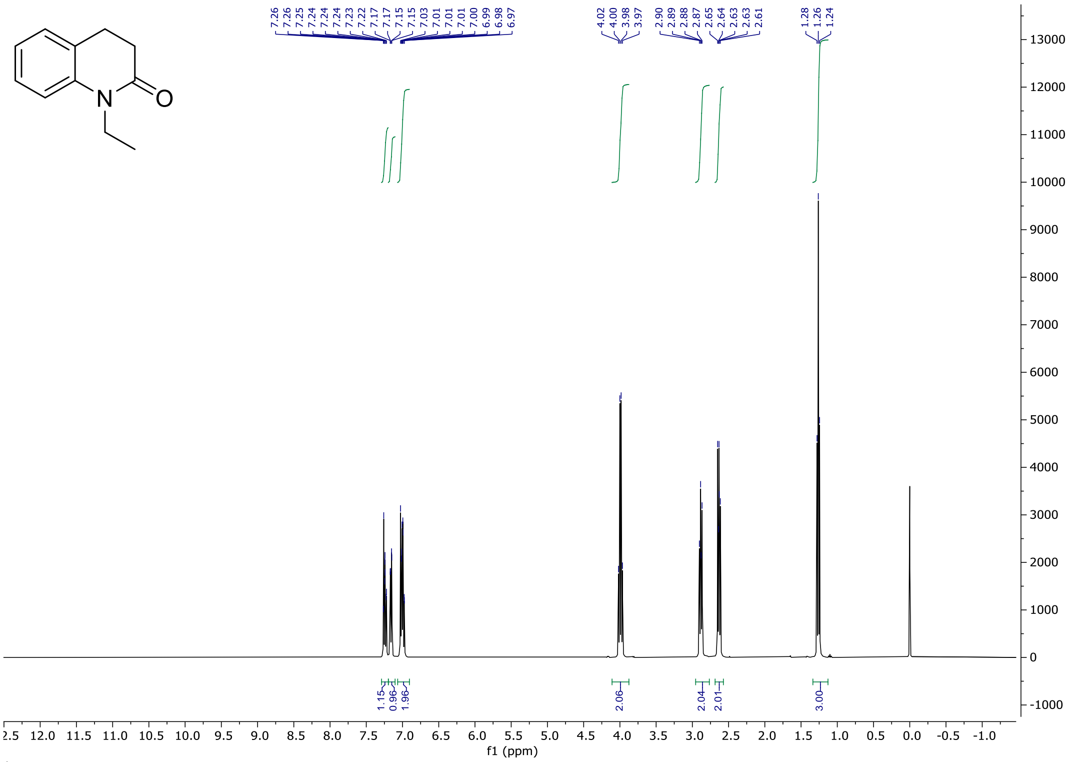

^{1}H NMR in $CDCl_3$.

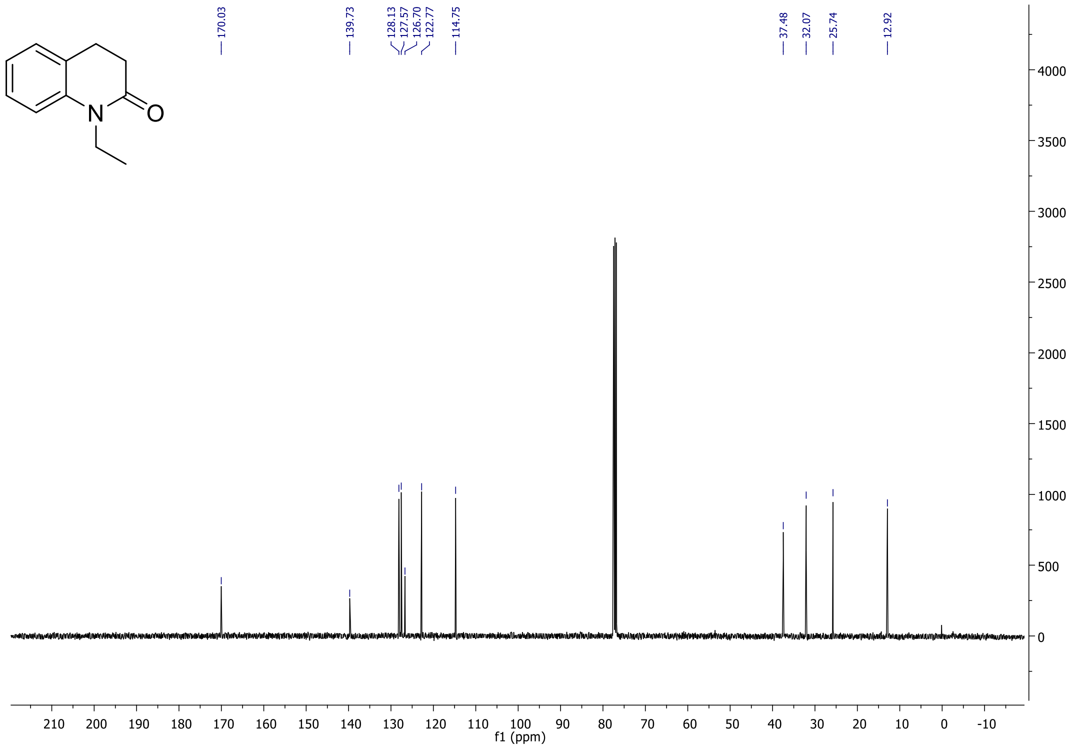

^{13}C NMR in $CDCl_3$.

1-Phenyl-3,4-dihydroquinolin-2(1*H*)-one

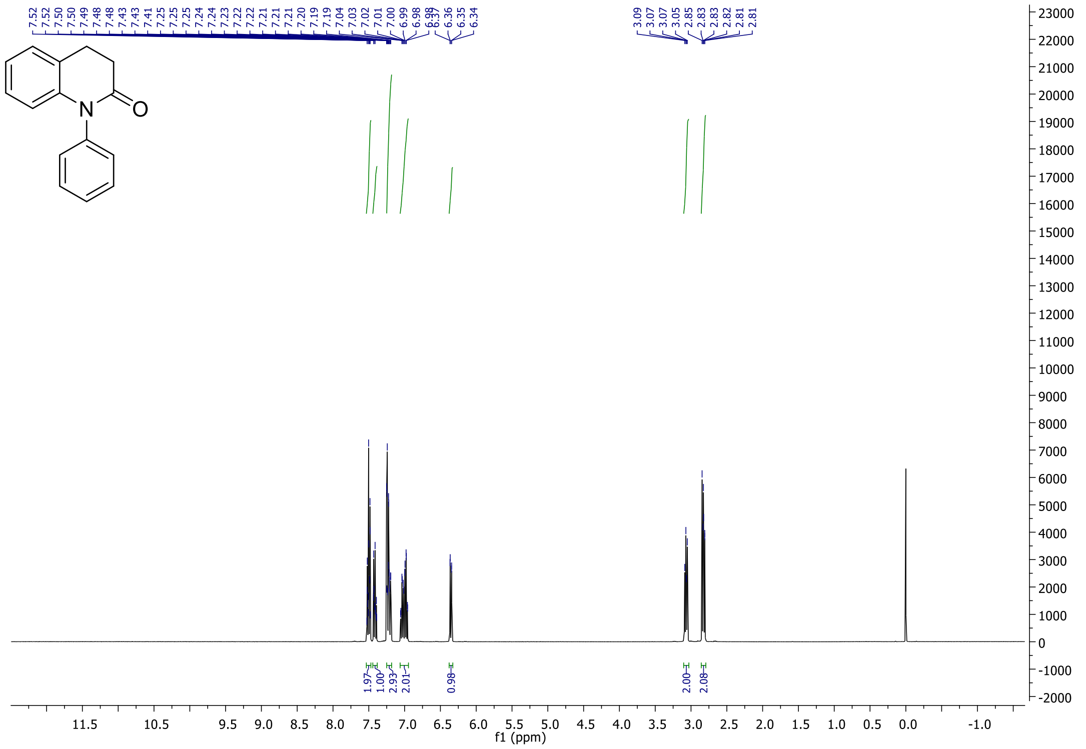

^{1}H NMR in $CDCl_3$.

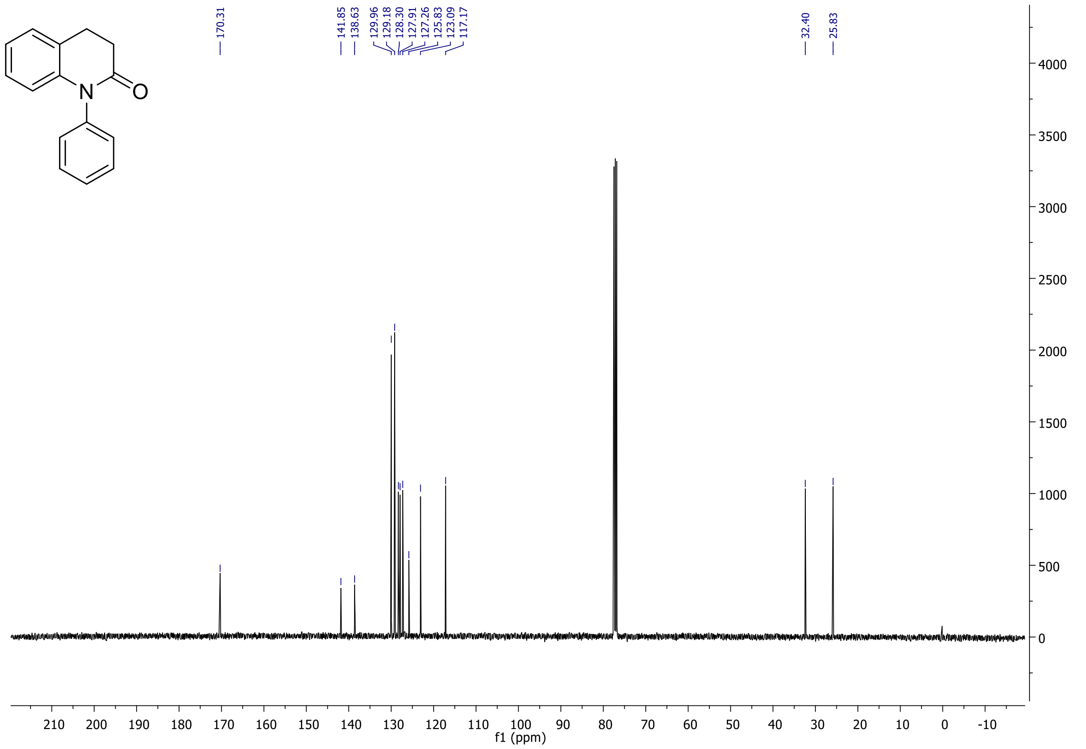

^{13}C NMR in $CDCl_3$.

1-(4-Methoxyphenyl)-3,4-dihydroquinolin-2(1*H*)-one

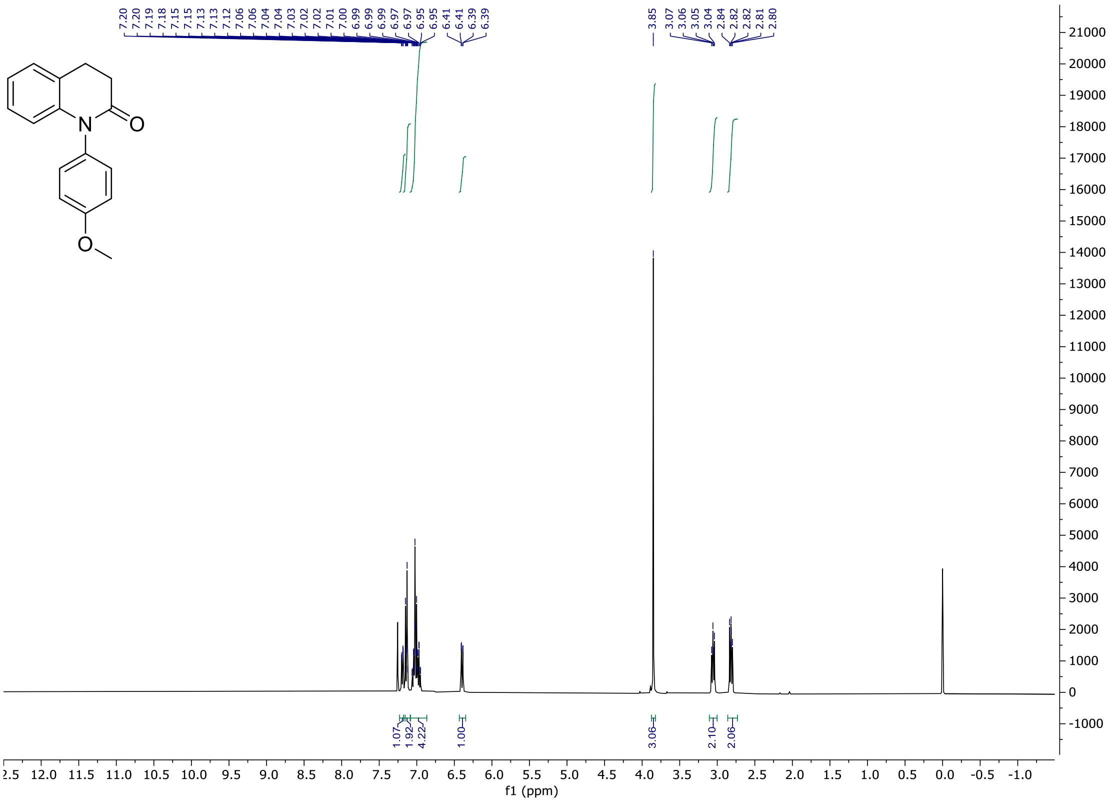

^{1}H NMR in $CDCl_3$.

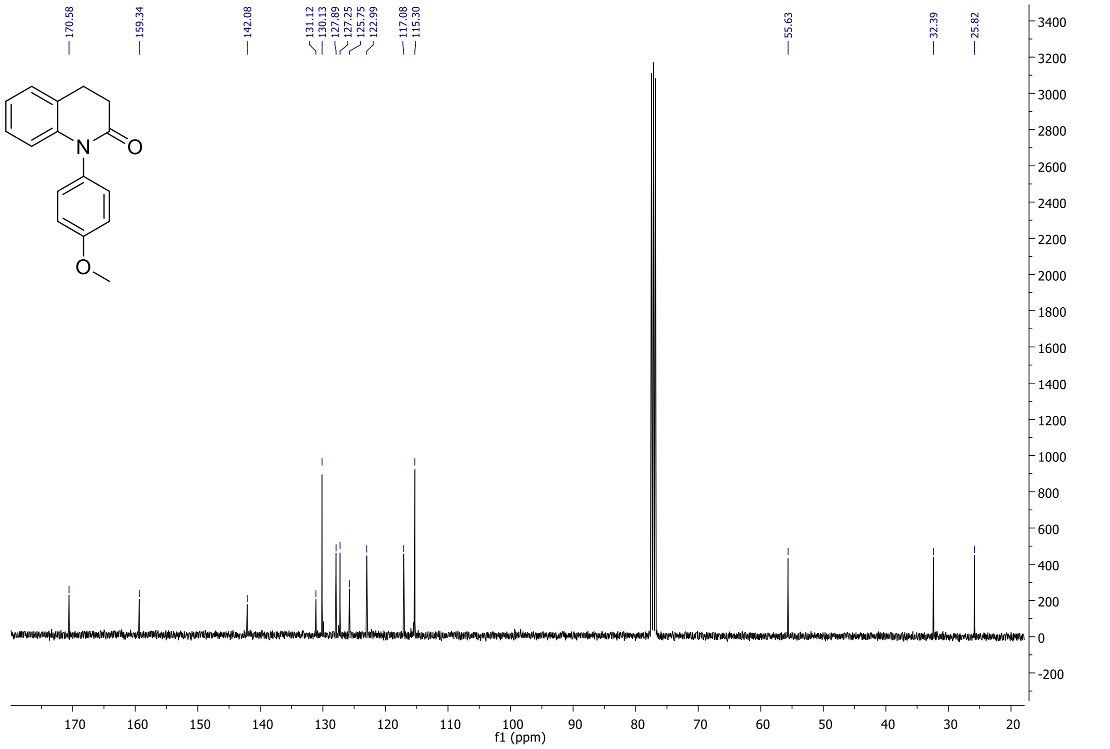

^{13}C NMR in $CDCl_3$.

1-(P-tolyl)-3,4-dihydroquinolin-2(1*H*)-one

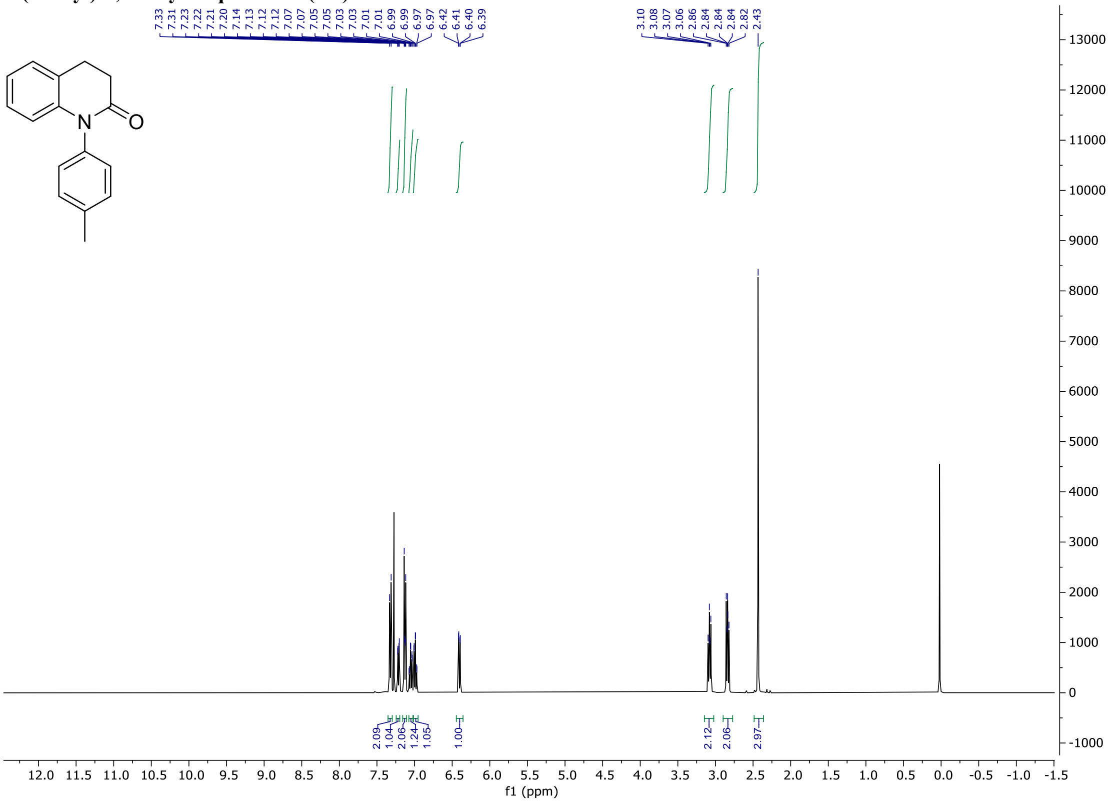

^{1}H NMR in CDCl3.

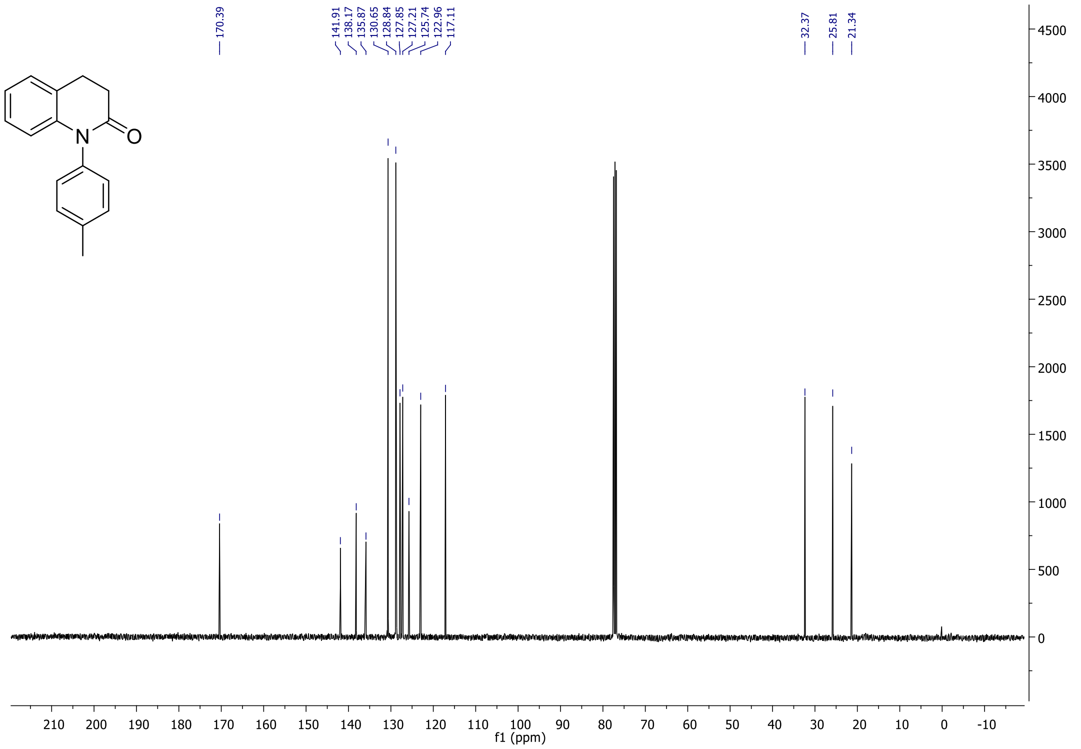

^{13}C NMR in CDCl3.

1-(4-Fluorophenyl)-3,4-dihydroquinolin-2(1*H*)-one

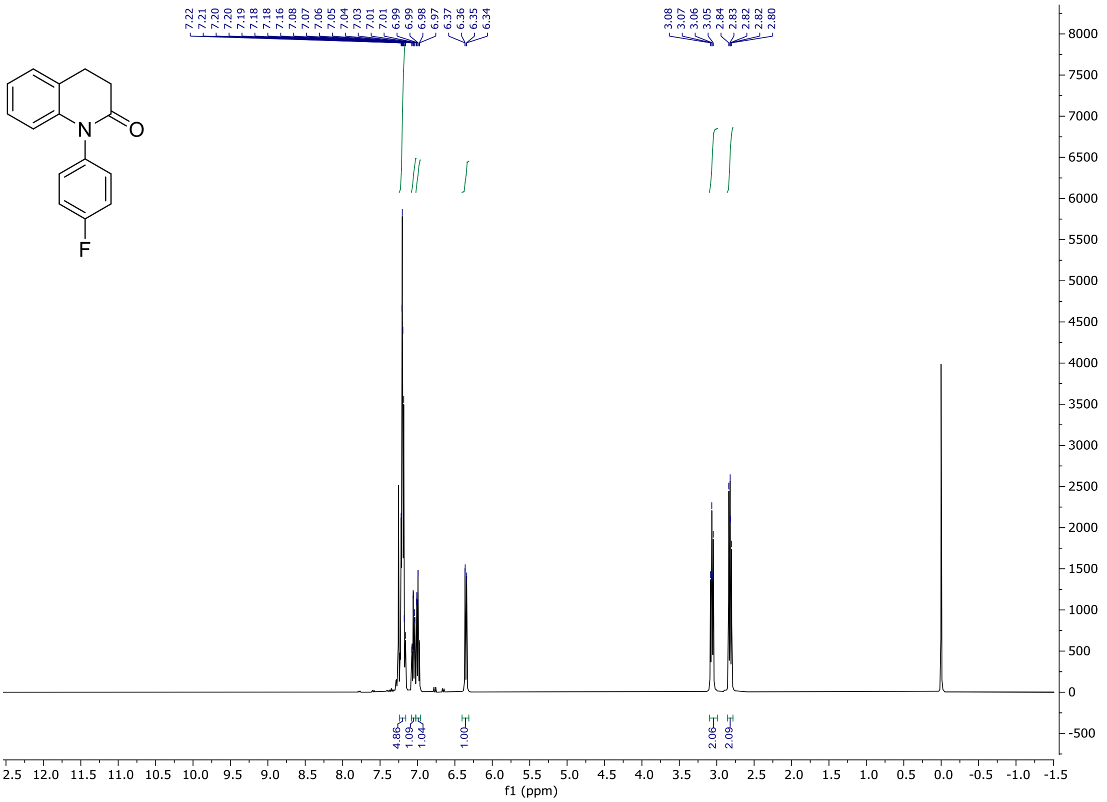

^{1}H NMR in $CDCl_3$.

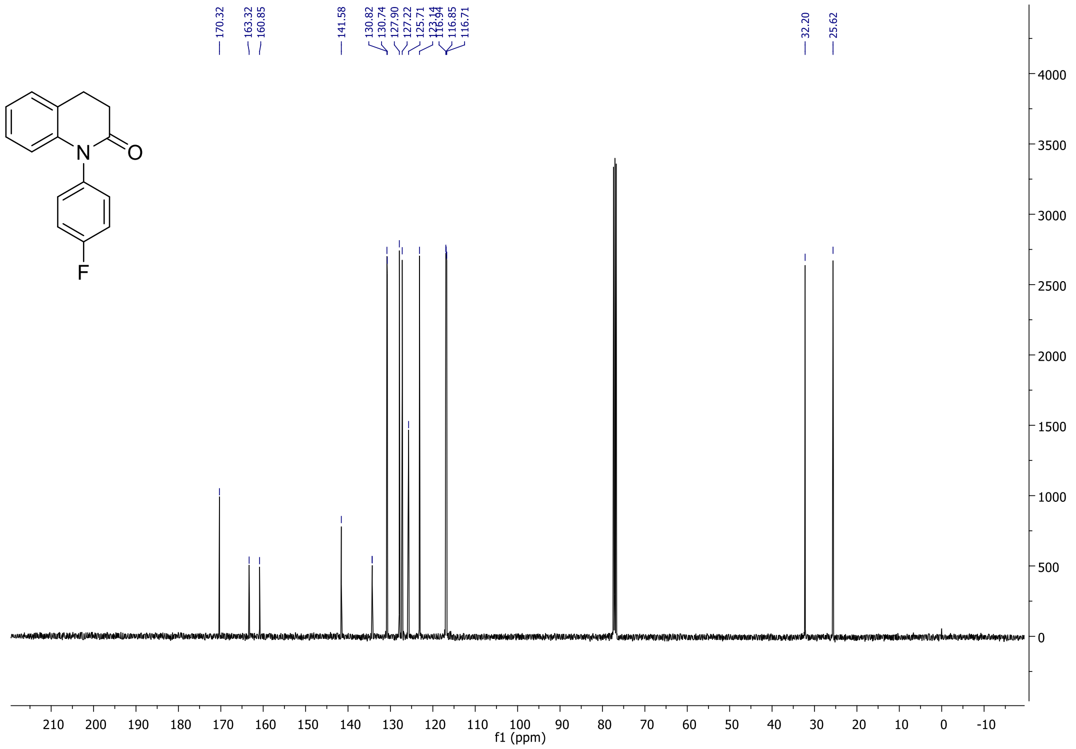

^{13}C NMR in $CDCl_3$.

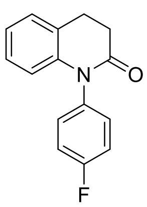

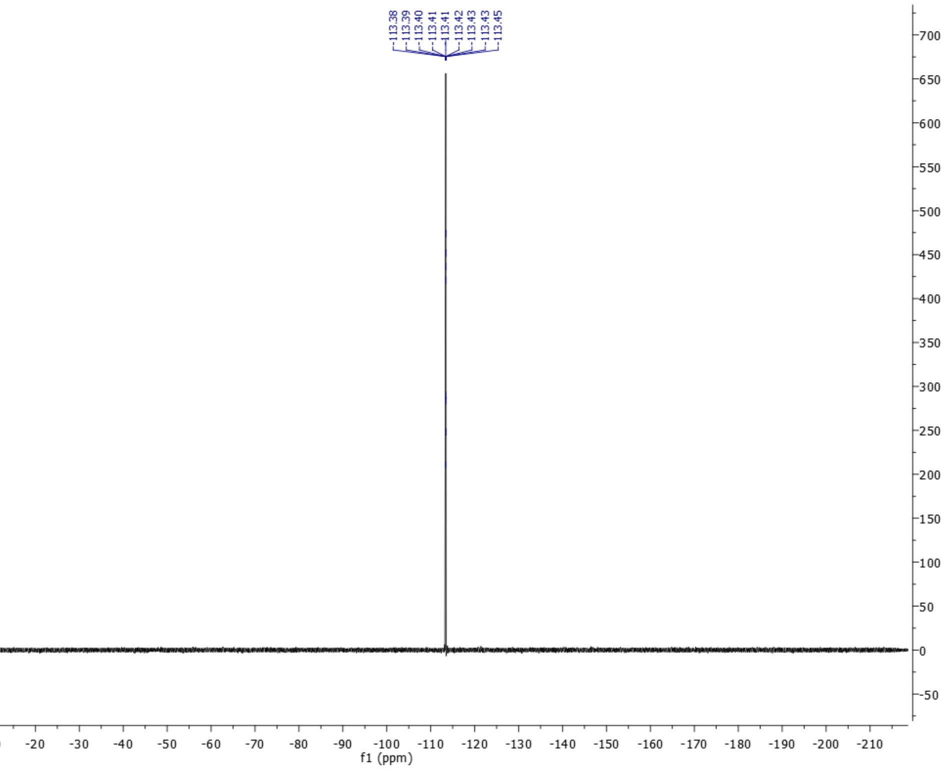

^{19}F NMR in $CDCl_3$.

1-(4-Cyanophenyl)-3,4-dihydroquinolin-2(1*H*)-one

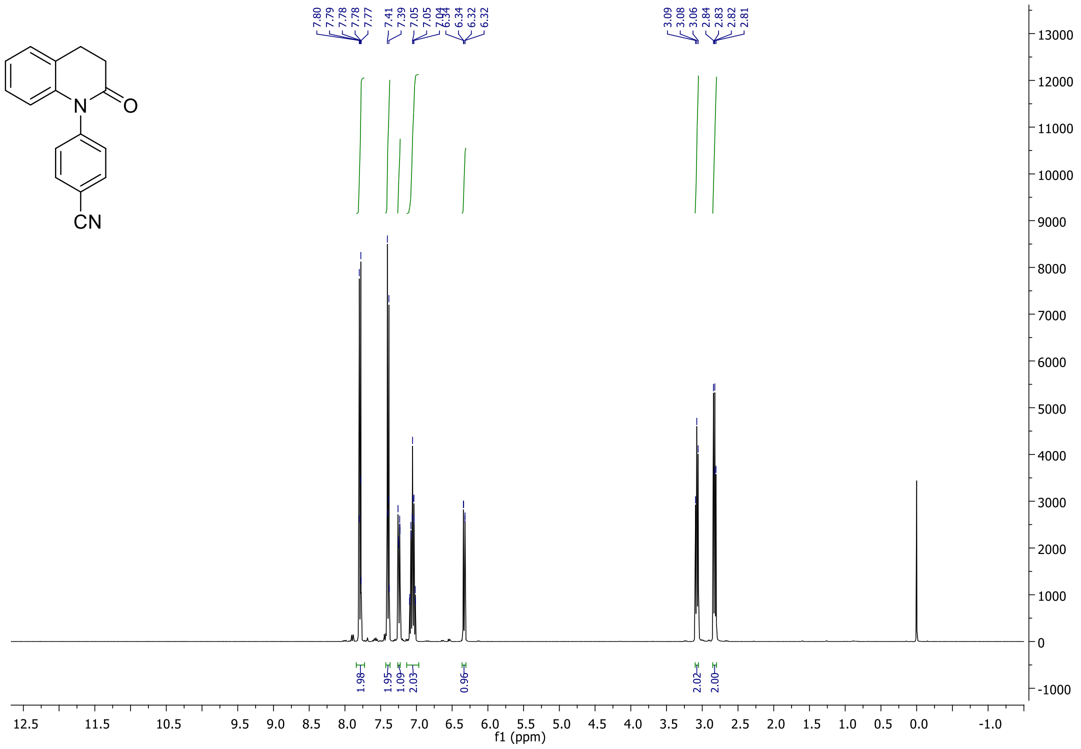

^{1}H NMR in CDCl3.

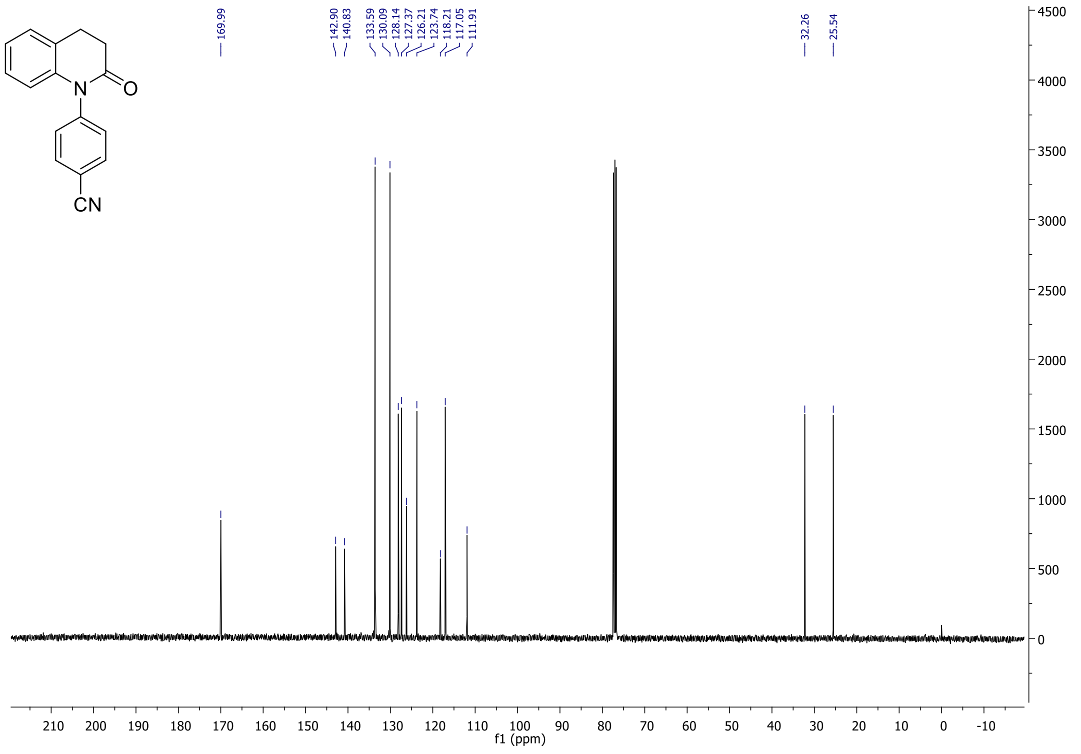

^{13}C NMR in CDCl3.

1-(3-Chlorophenyl)-3,4-dihydroquinolin-2(1*H*)-one

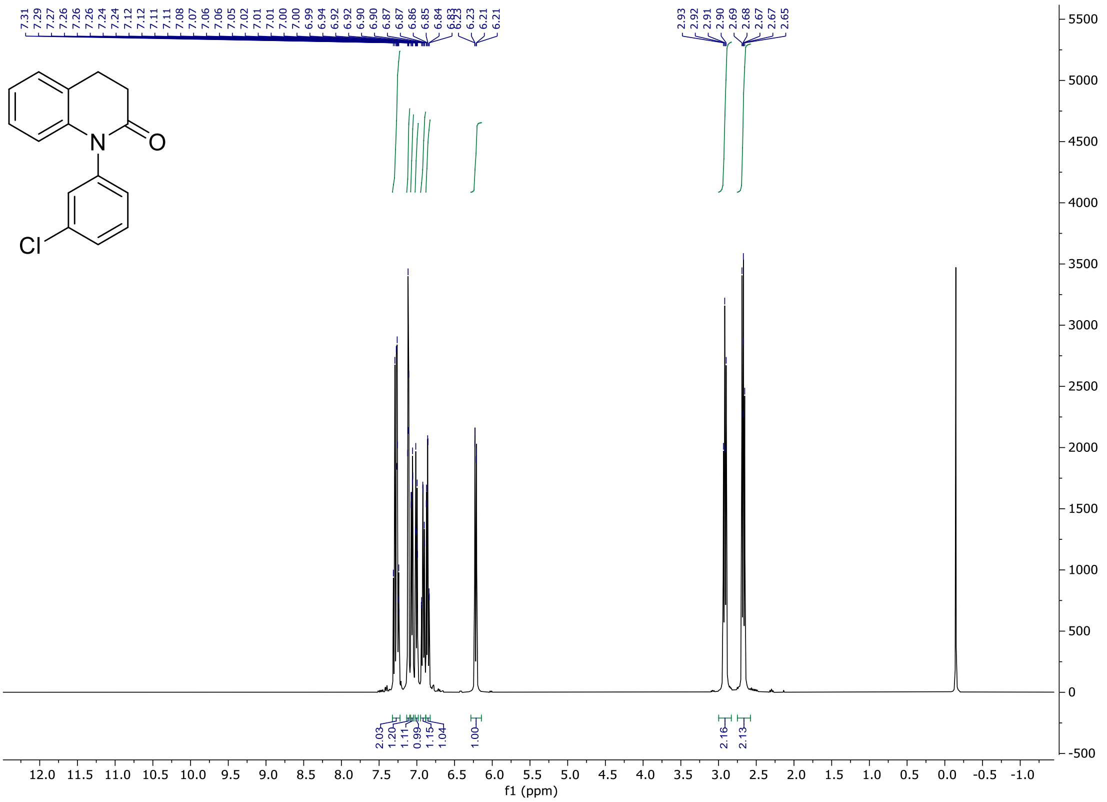

^{1}H NMR in $CDCl_3$.

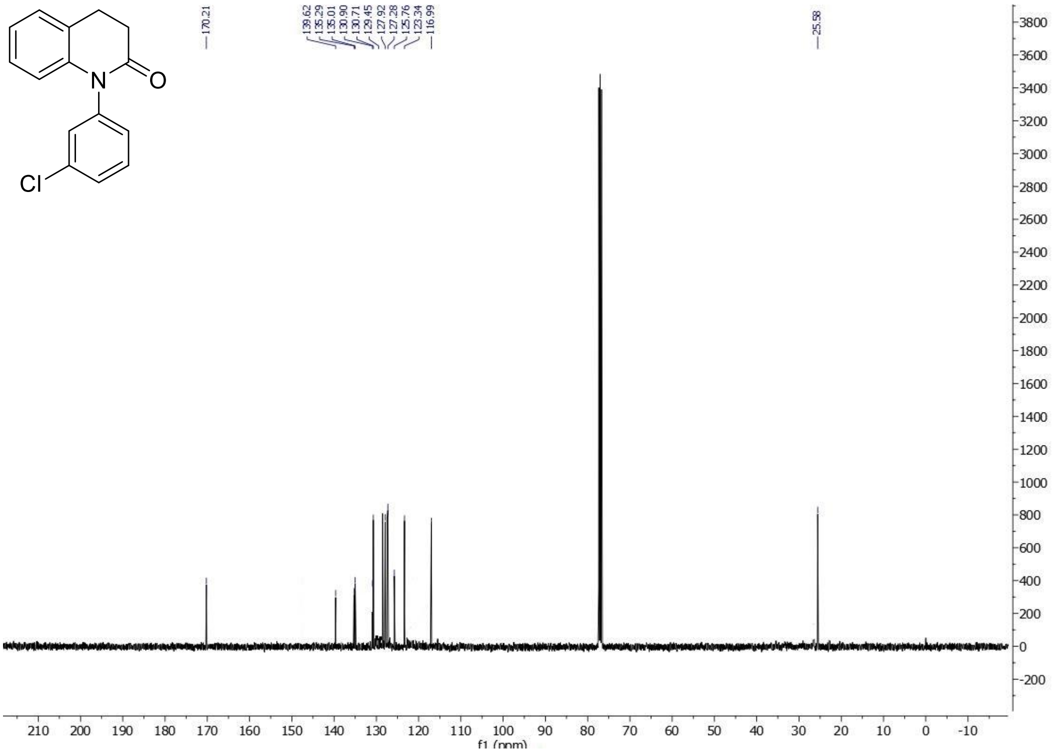

^{13}C NMR in $CDCl_3$.

7-(2-Oxo-2-phenylethoxy)-3,4-dihydroquinolin-2(1*H*)-one

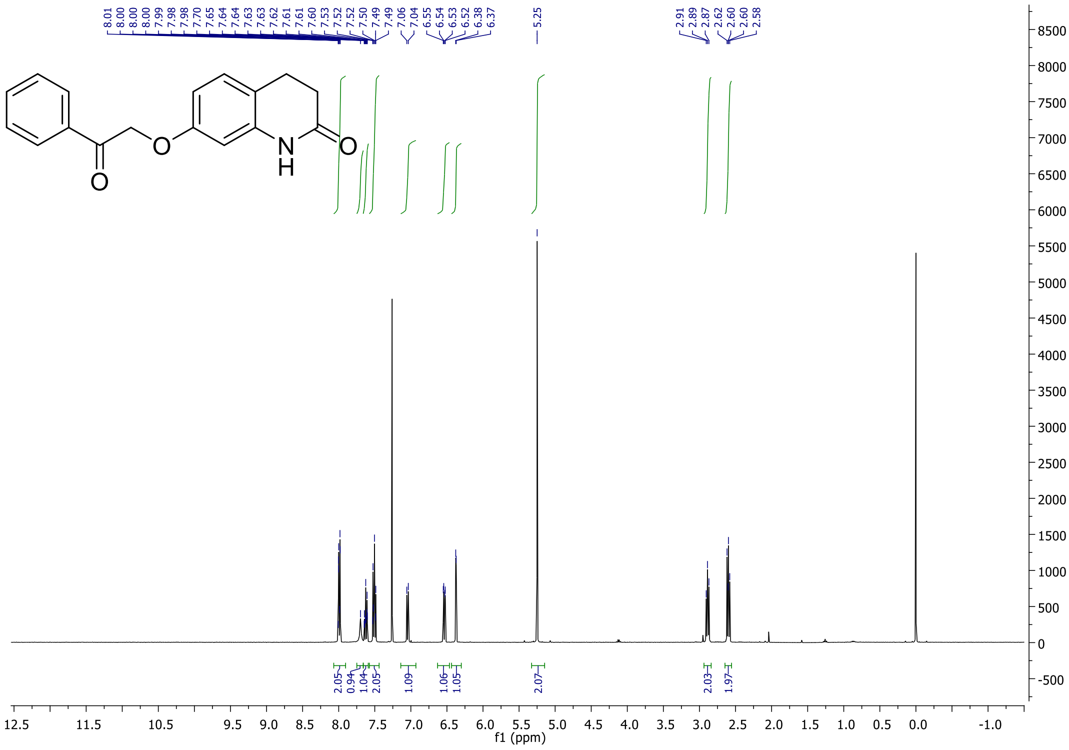

^{1}H NMR in $CDCl_3$.

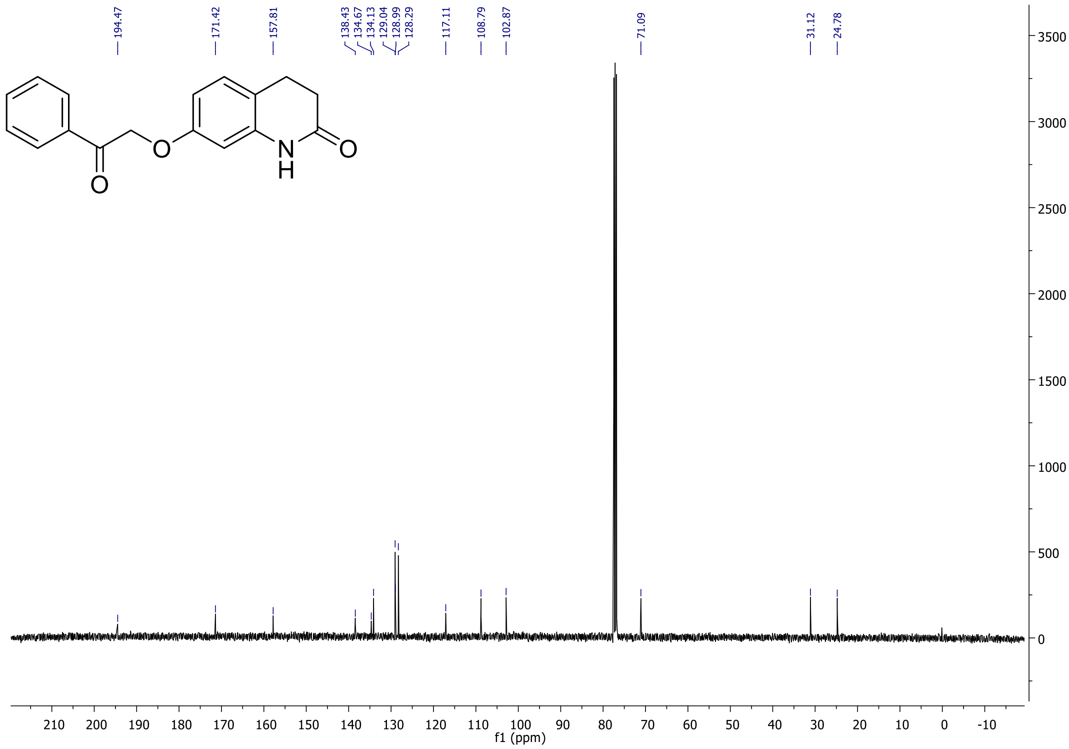

^{13}C NMR in $CDCl_3$.

4-Oxo-2-phenylchroman-7-yl benzoate

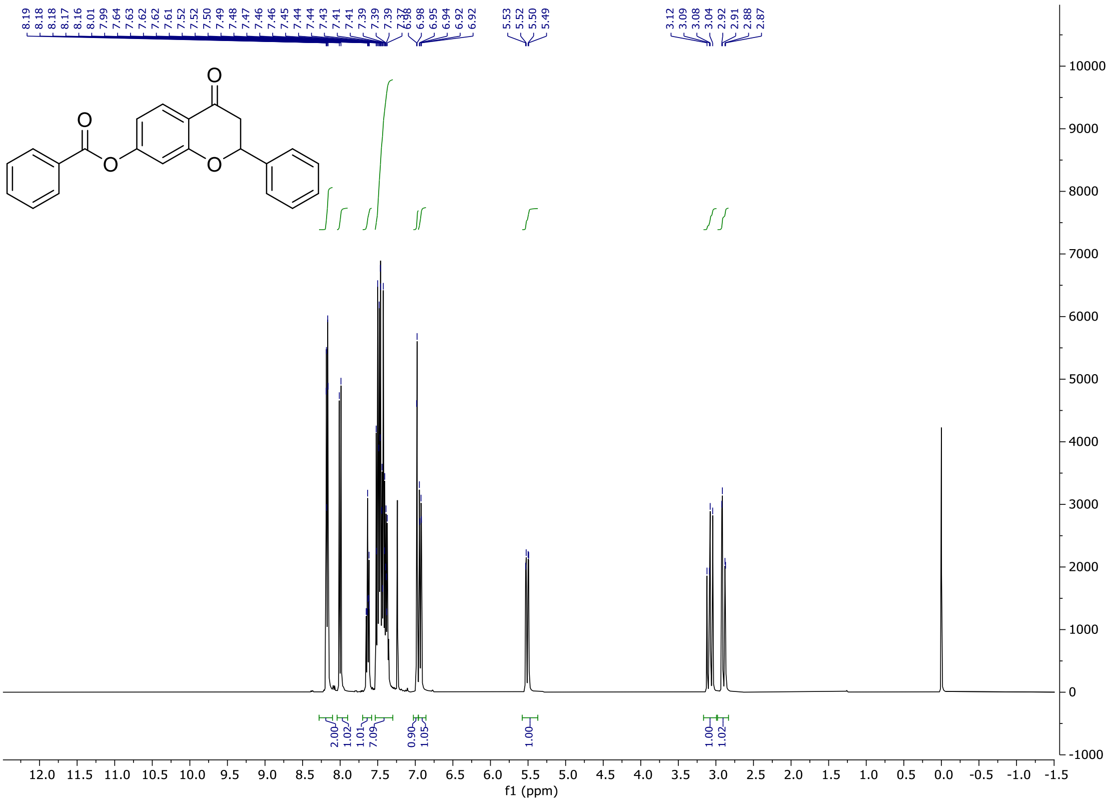

^{1}H NMR in $CDCl_3$.

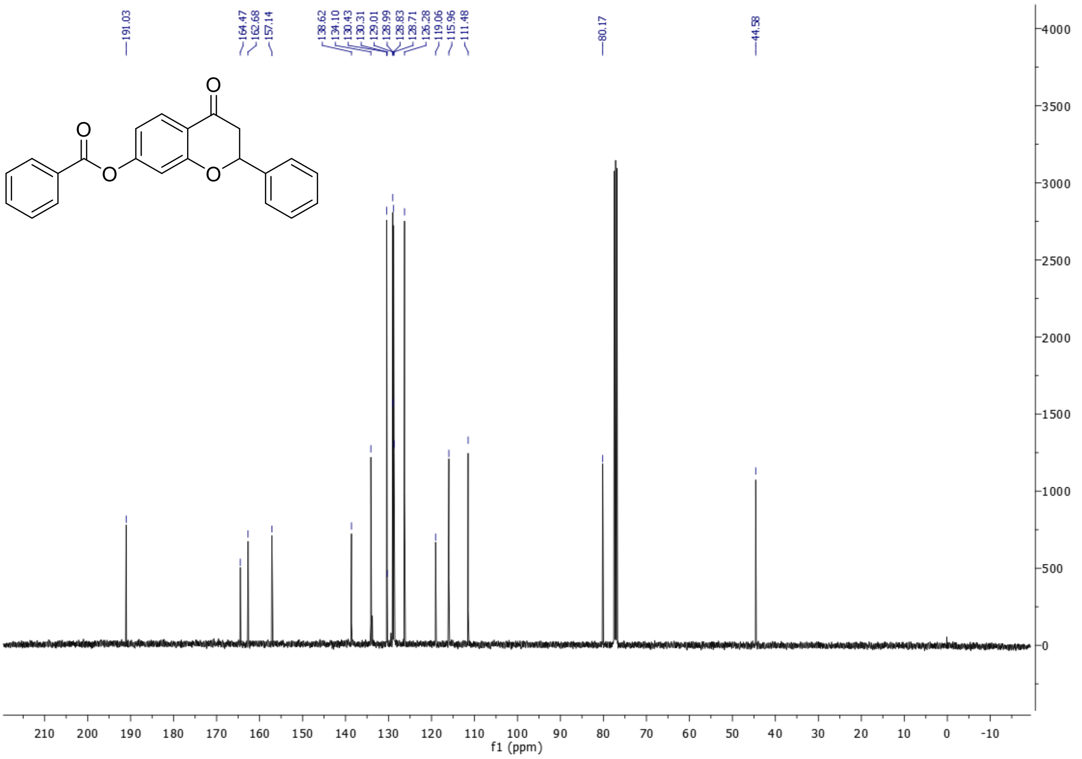

^{13}C NMR in $CDCl_3$.

7-Methoxy-1-methyl-3,4-dihydroquinolin-2(1*H*)-one

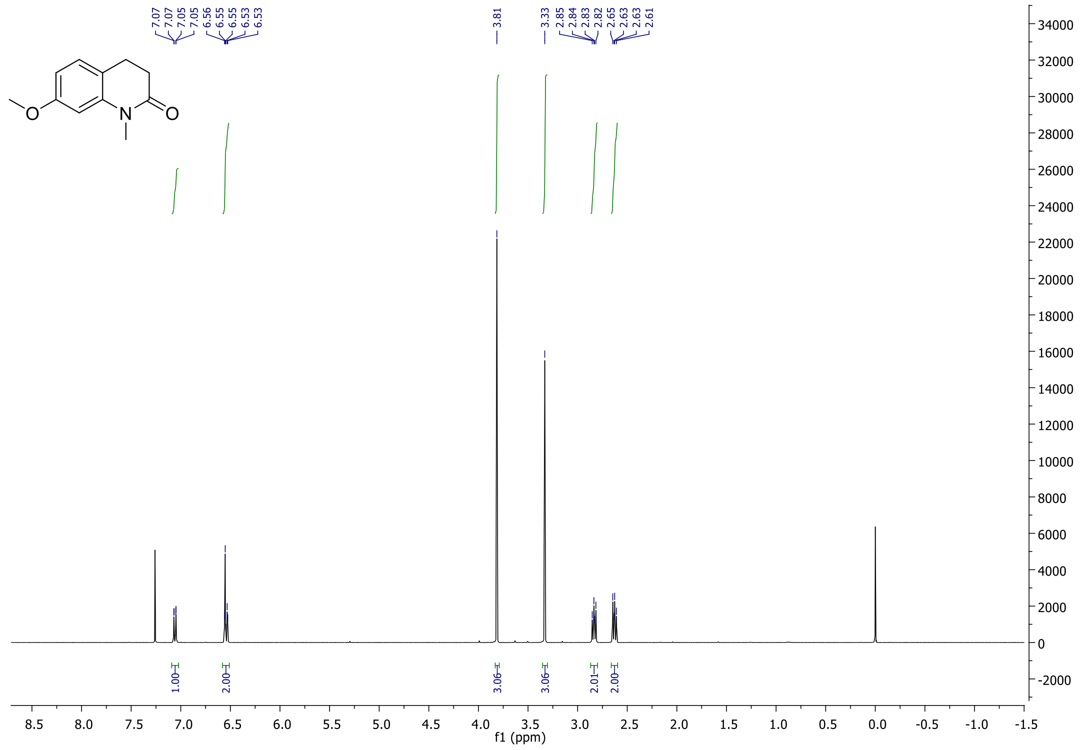

^{1}H NMR in $CDCl_3$.

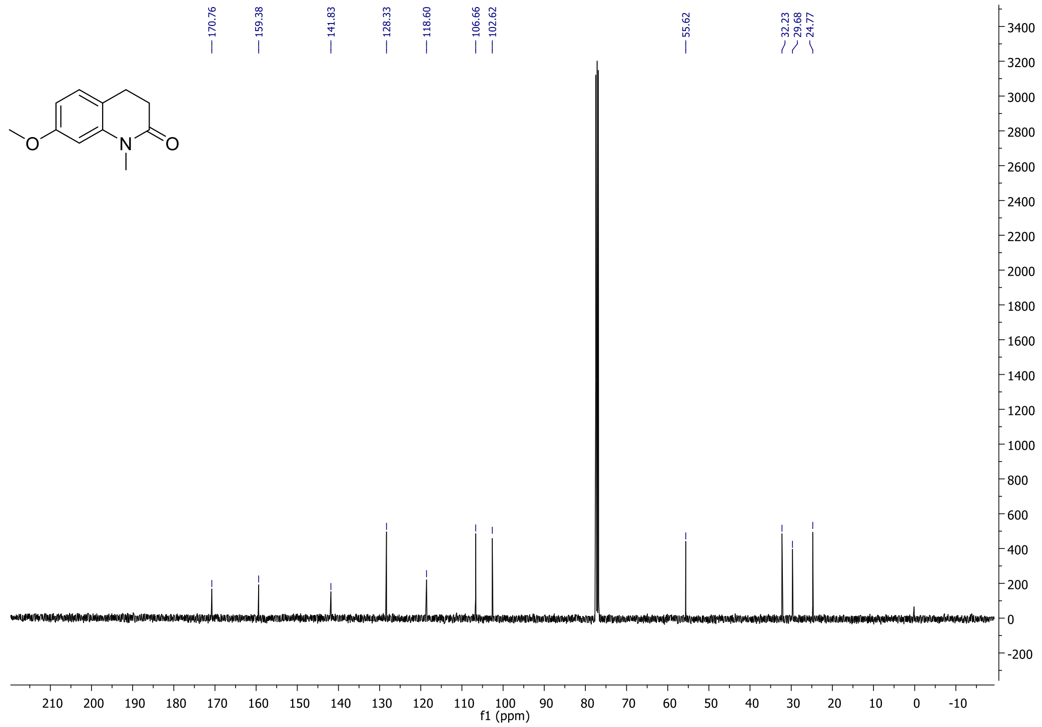

^{13}C NMR in $CDCl_3$.

7-Hydroxy-1-methyl-3,4-dihydroquinolin-2(1*H*)-one

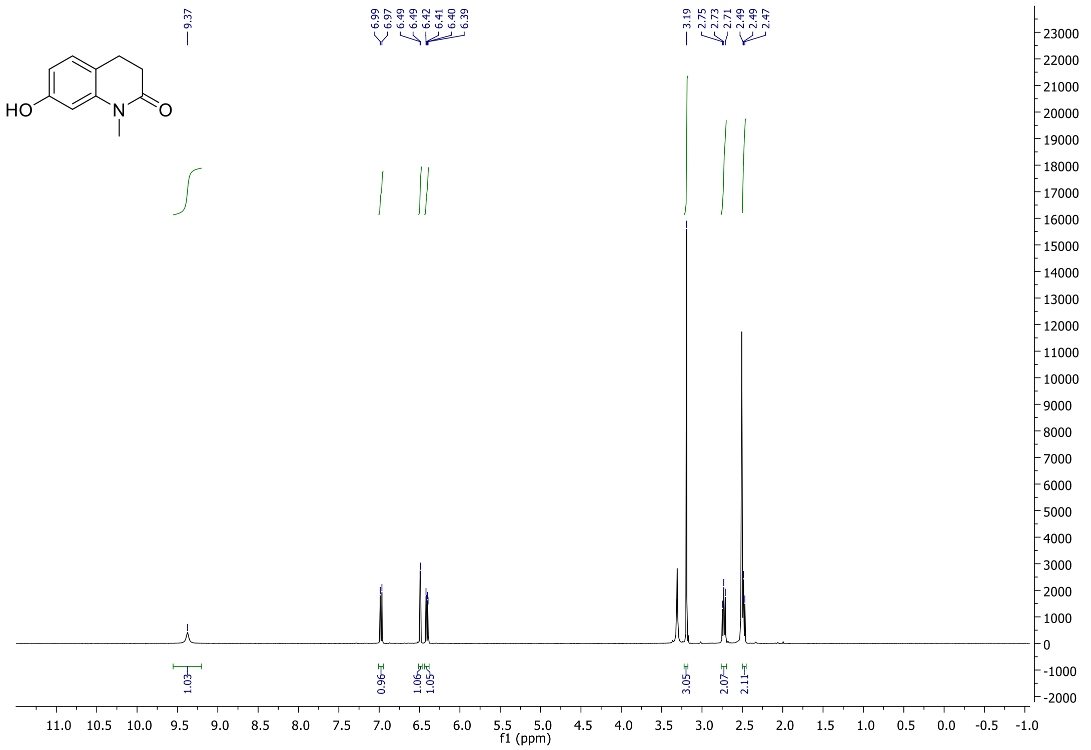

^{1}H NMR in DMSO-d6.

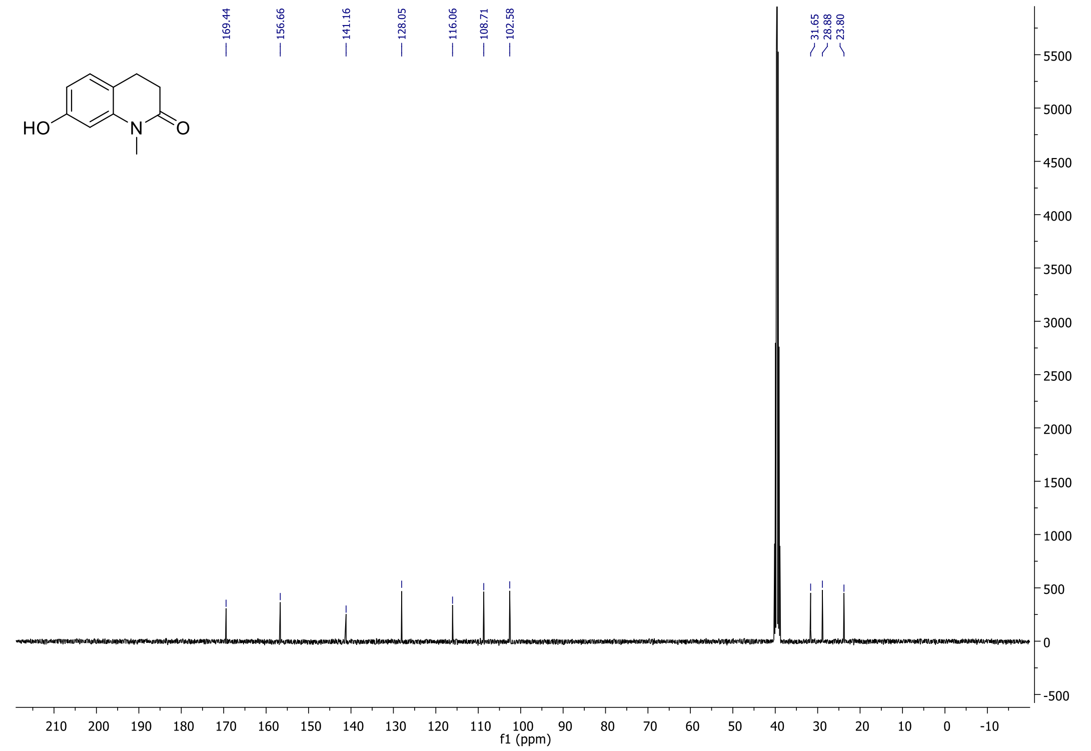

^{13}C NMR in DMSO-d6.

2-(4-Isobutylphenyl)-*N*-(2-oxo-1,2,3,4-tetrahydroquinolin-7-yl)propenamide

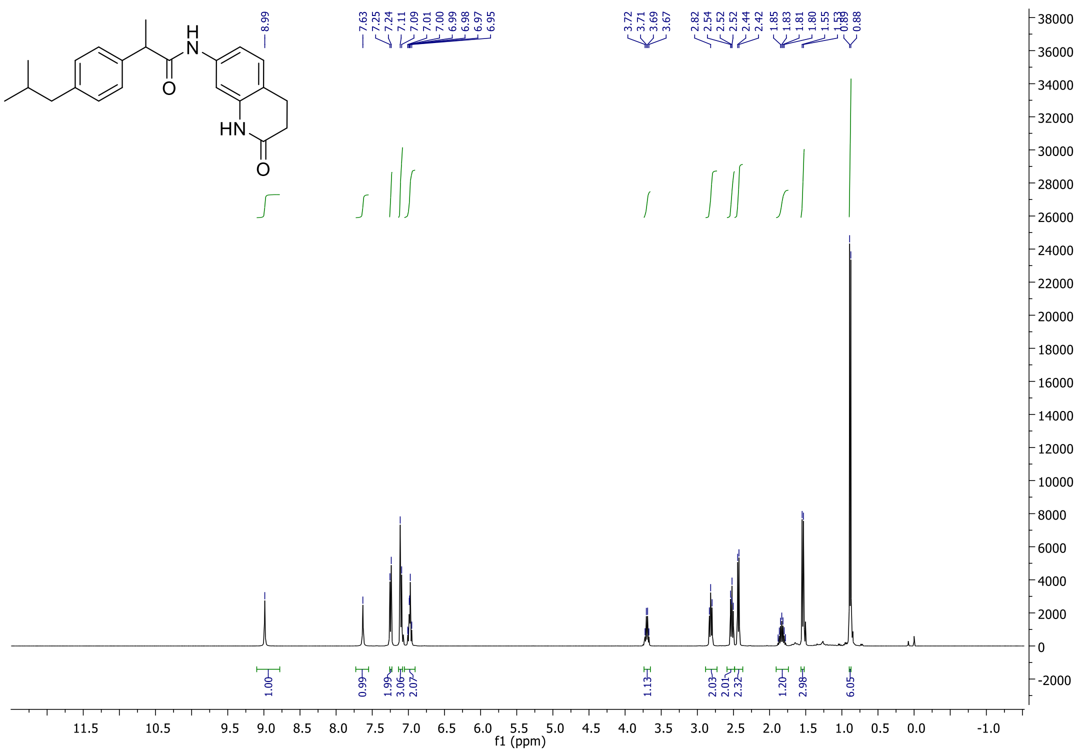

^{1}H NMR in $CDCl_3$.

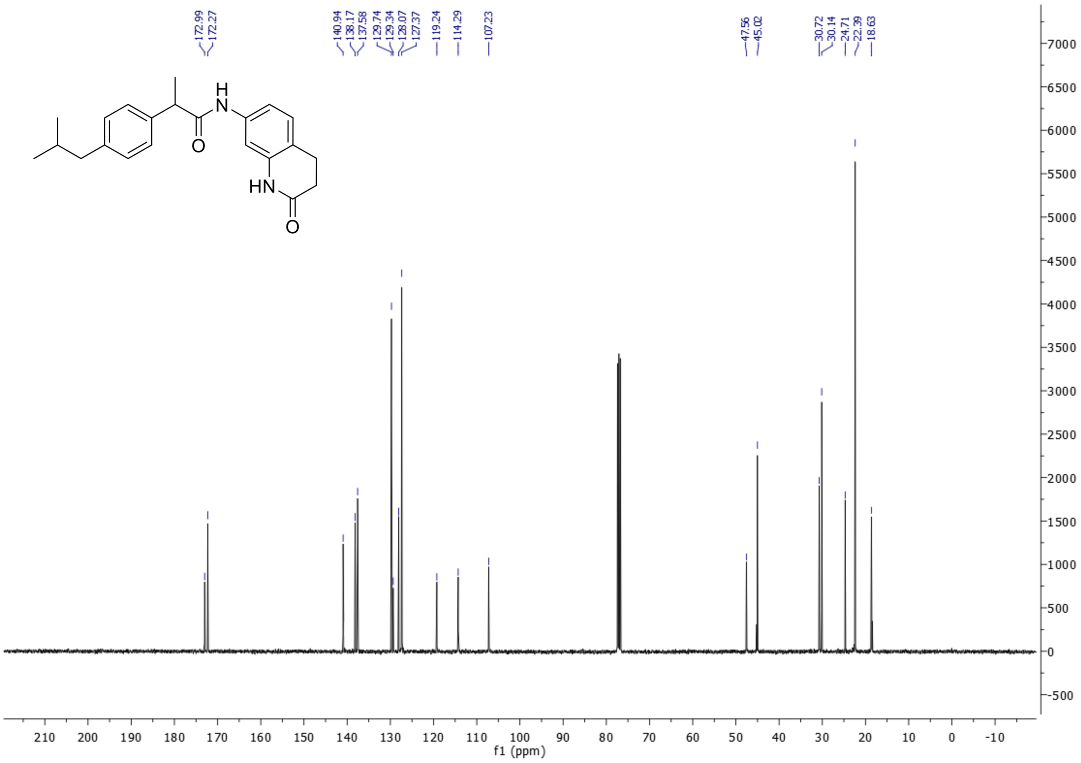

^{13}C NMR in $CDCl_3$.

2-(3-Benzoylphenyl)-*N*-(2-oxo-1,2-dihydroquinolin-7-yl)propenamide

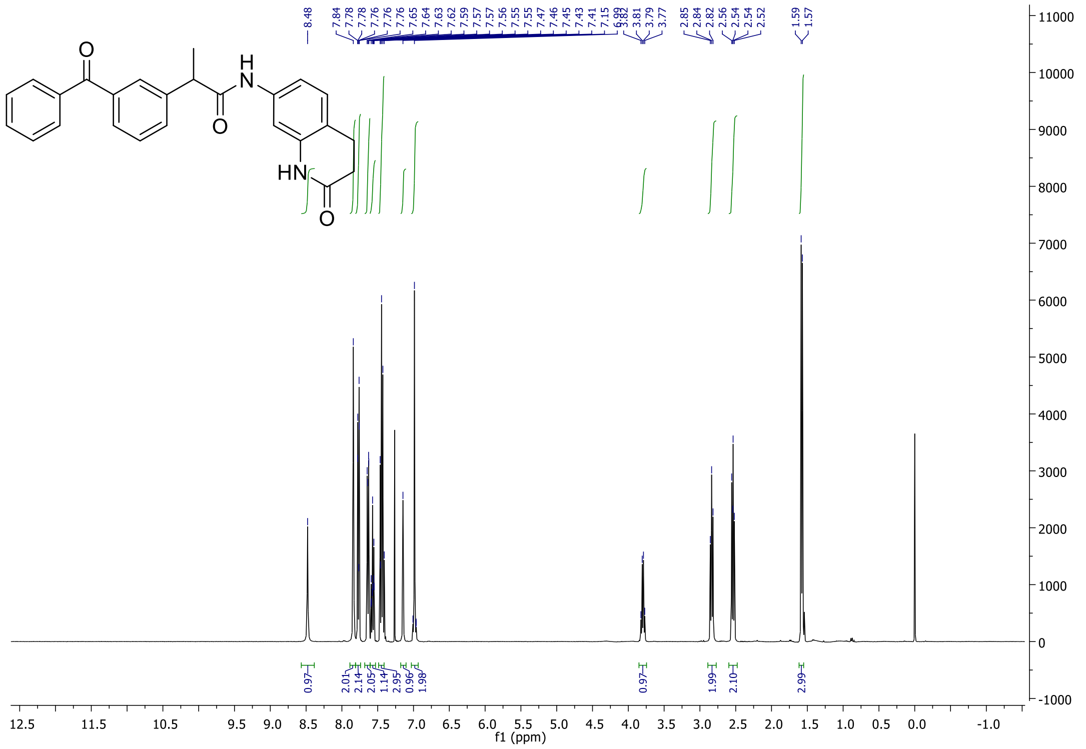

^{1}H NMR in $CDCl_3$.

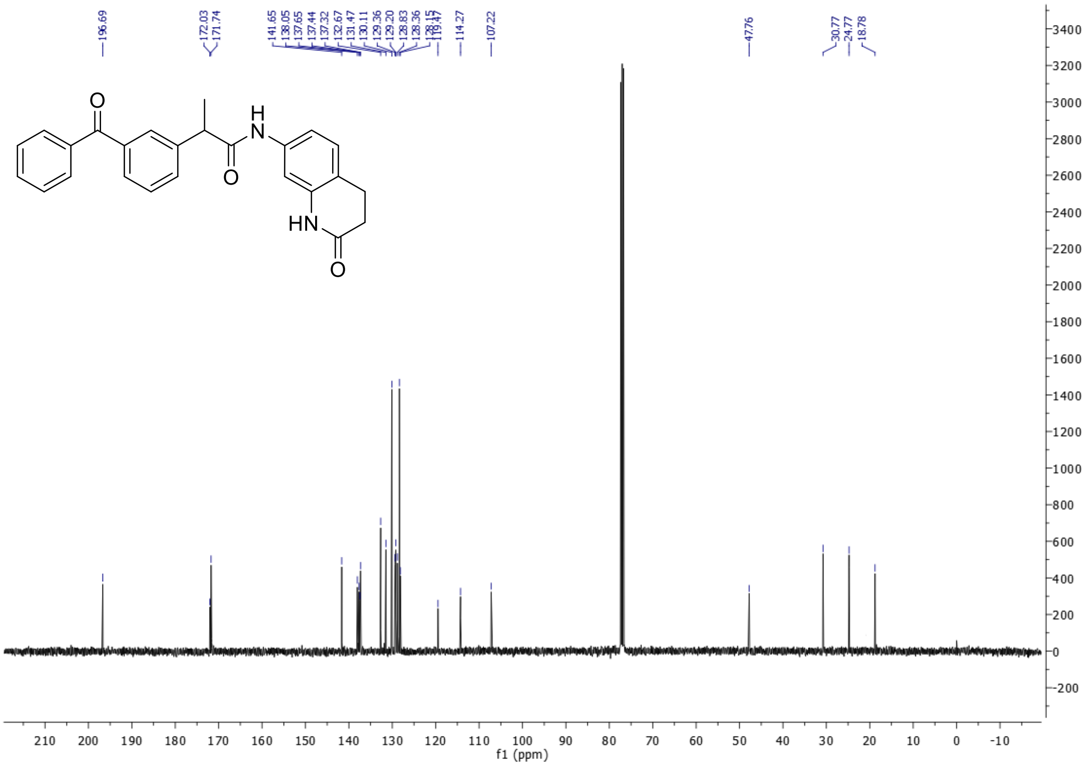

^{13}C NMR in $CDCl_3$.

Tert-butyl (1-oxo-1-((2-oxo-1,2,3,4-tetrahydroquinolin-7-yl)amino)-3-phenylpropan-2-yl)carbamate

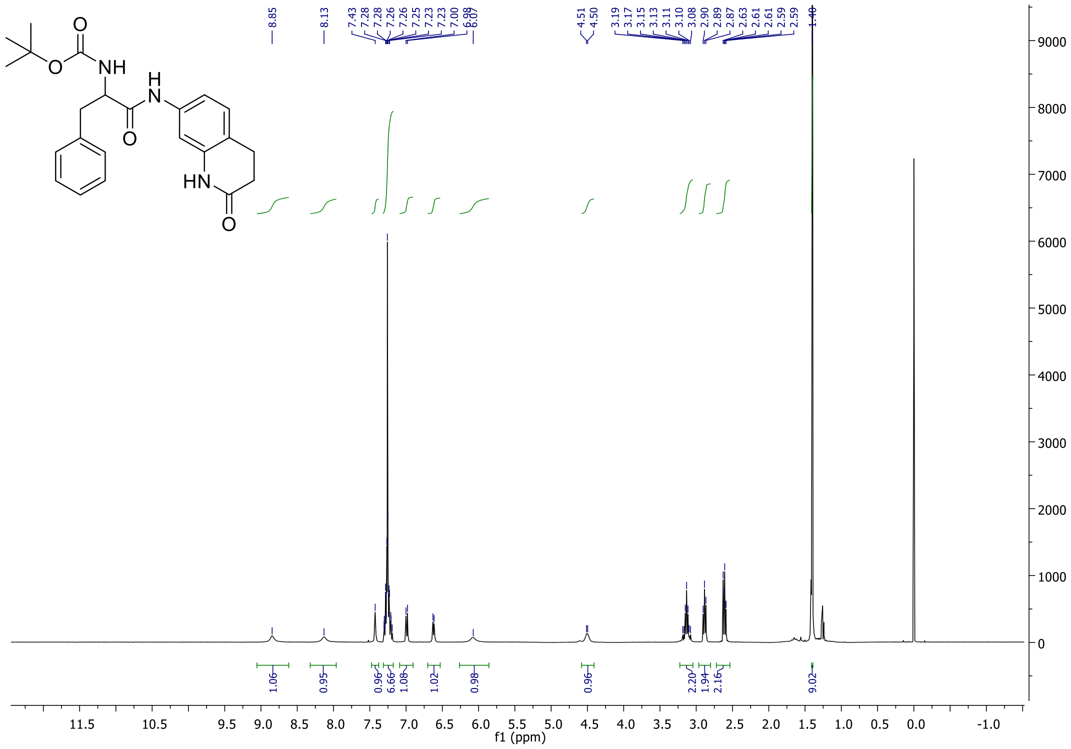

^{1}H NMR in $CDCl_3$.

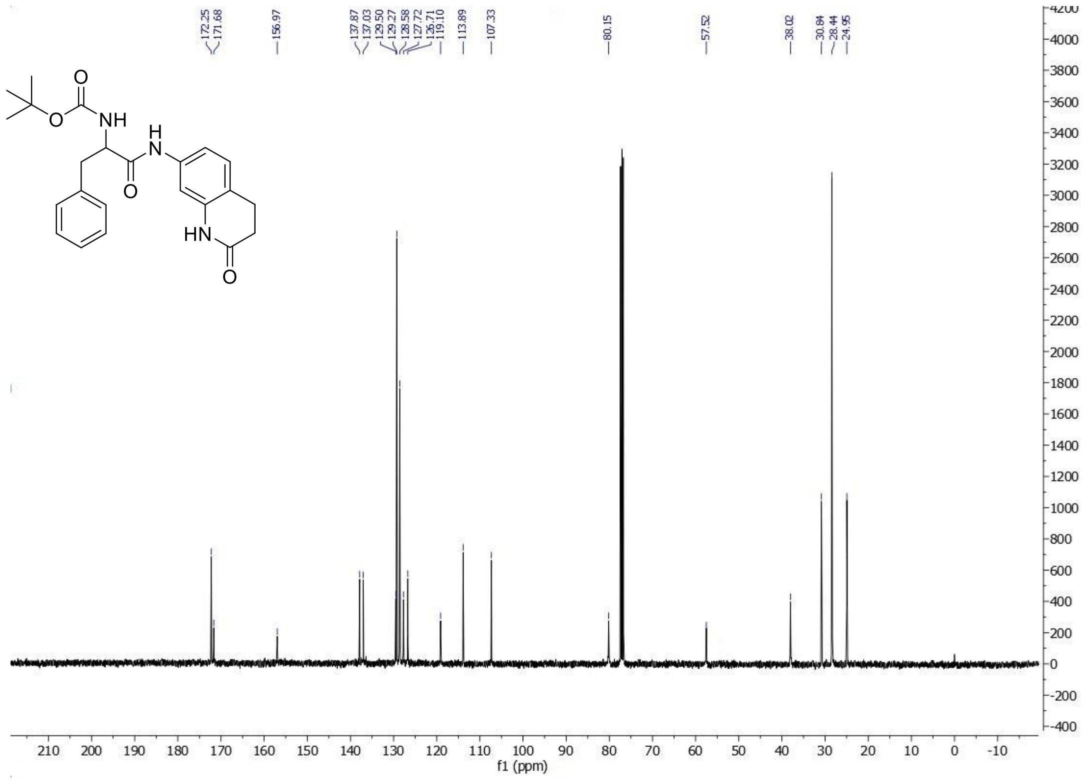

^{13}C NMR in $CDCl_3$.

2-(3-Acetyl-2,2-dimethylcyclobutyl)-*N*-(2-oxo-1,2,3,4-tetrahydroquinolin-7-yl)acetamide

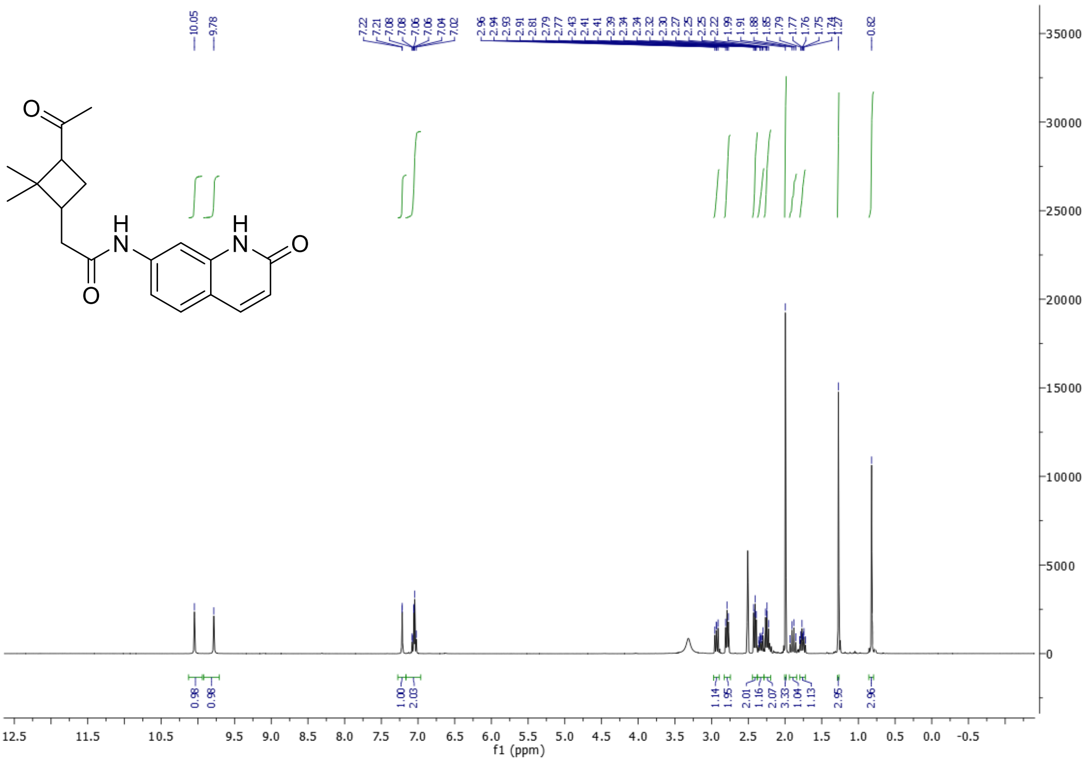

^{1}H NMR in DMSO-d6.

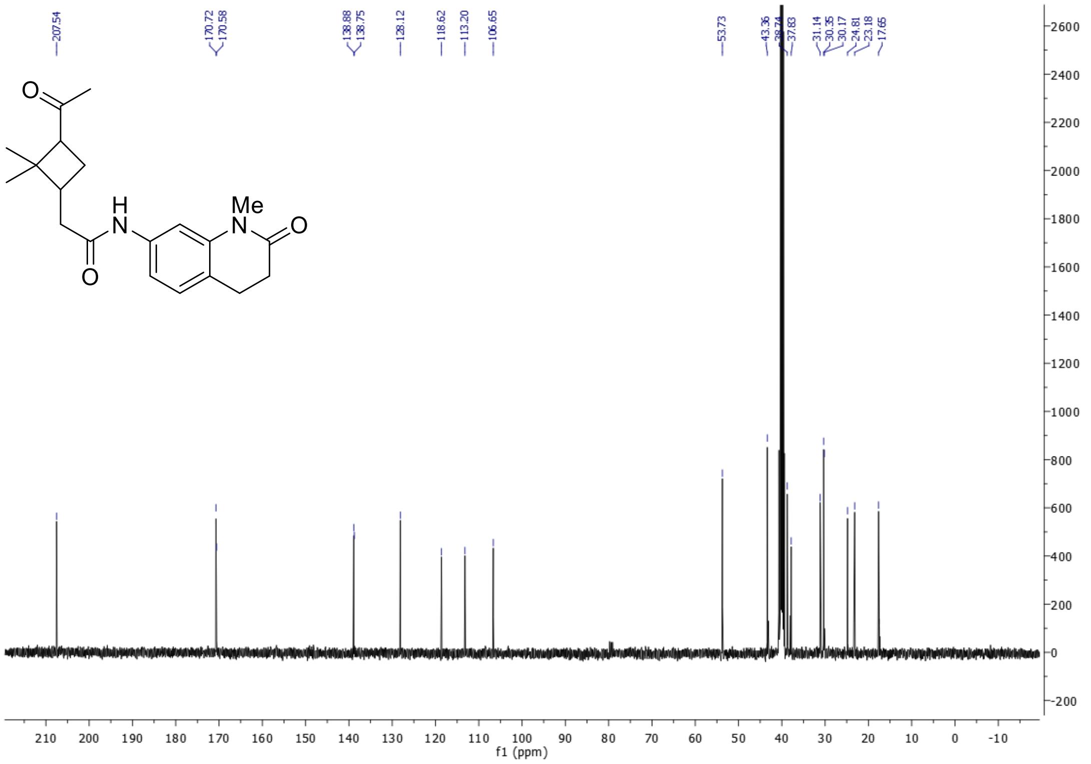

^{13}C NMR in DMSO-d6.

8-Fluoro-3-methyl-3,4-dihydroquinolin-2(1*H*)-one

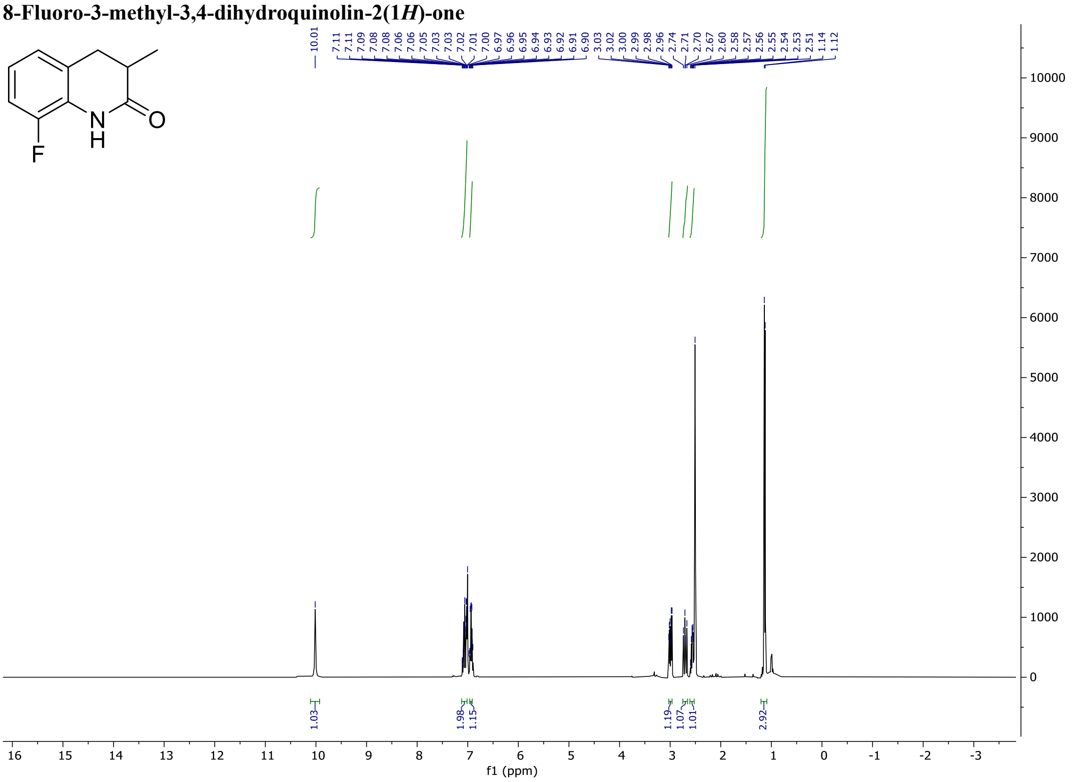

^{1}H NMR in DMSO-d6.

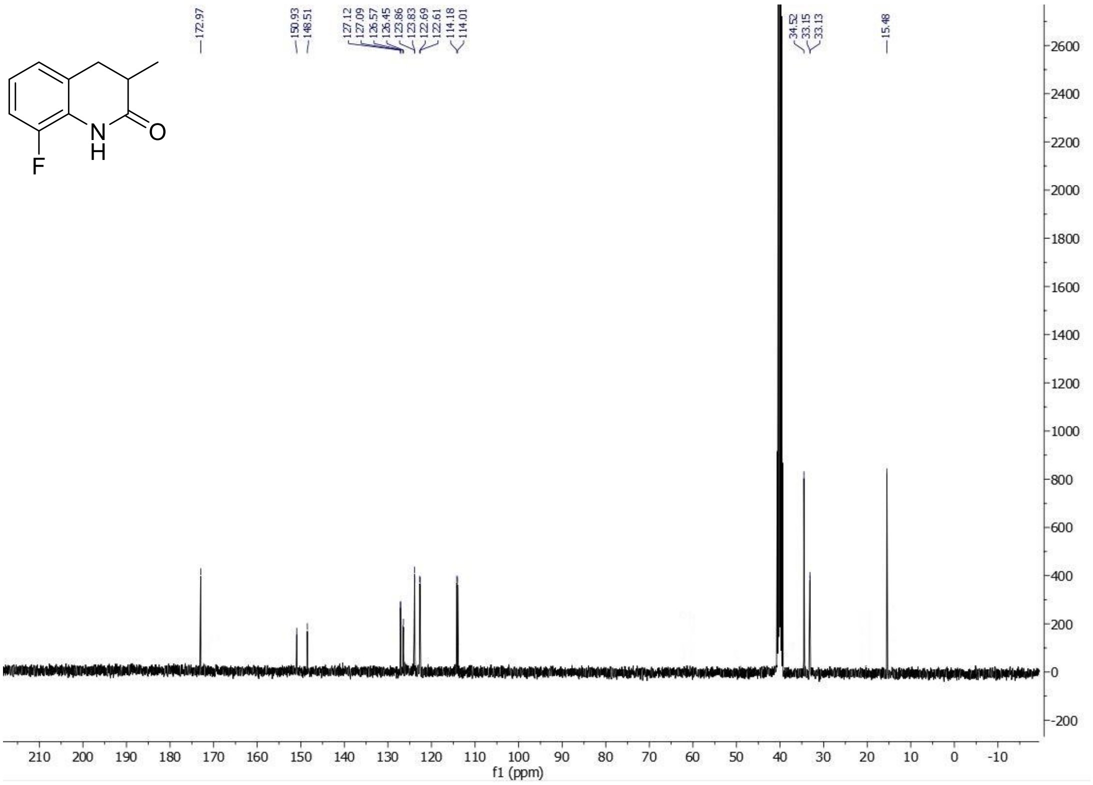

^{13}C NMR in DMSO-d6.

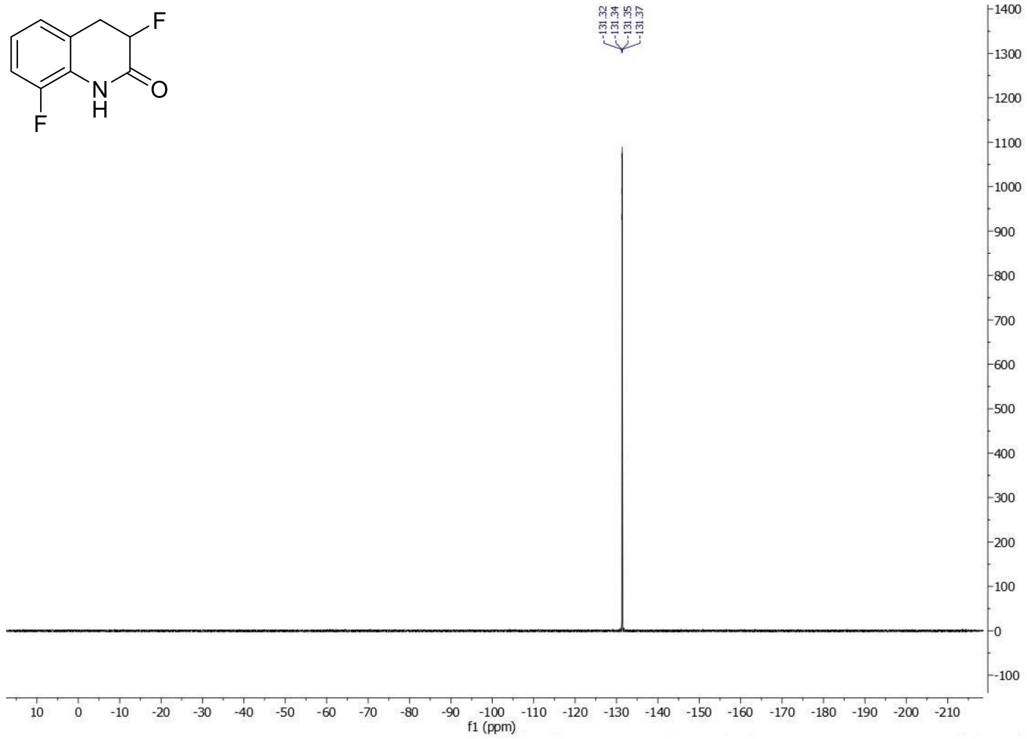

^{19}F NMR in DMSO-d6.

1-Methoxy-4-nitropyridine tetrafluoroborate

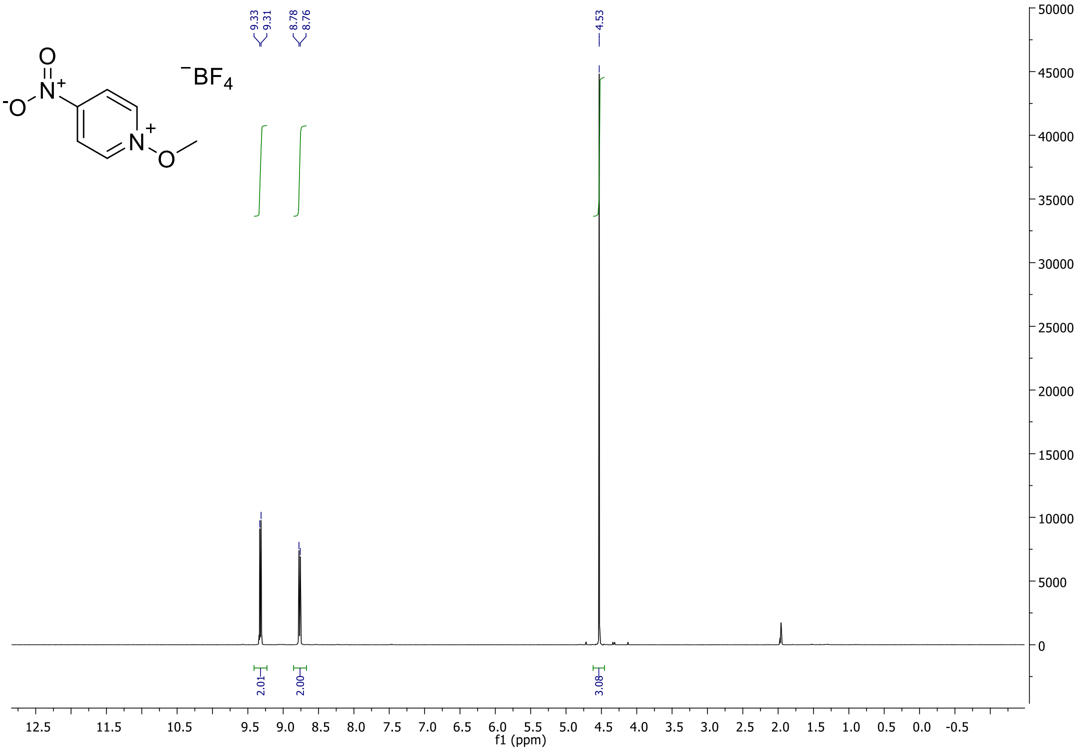

^{1}H NMR in CDCN.

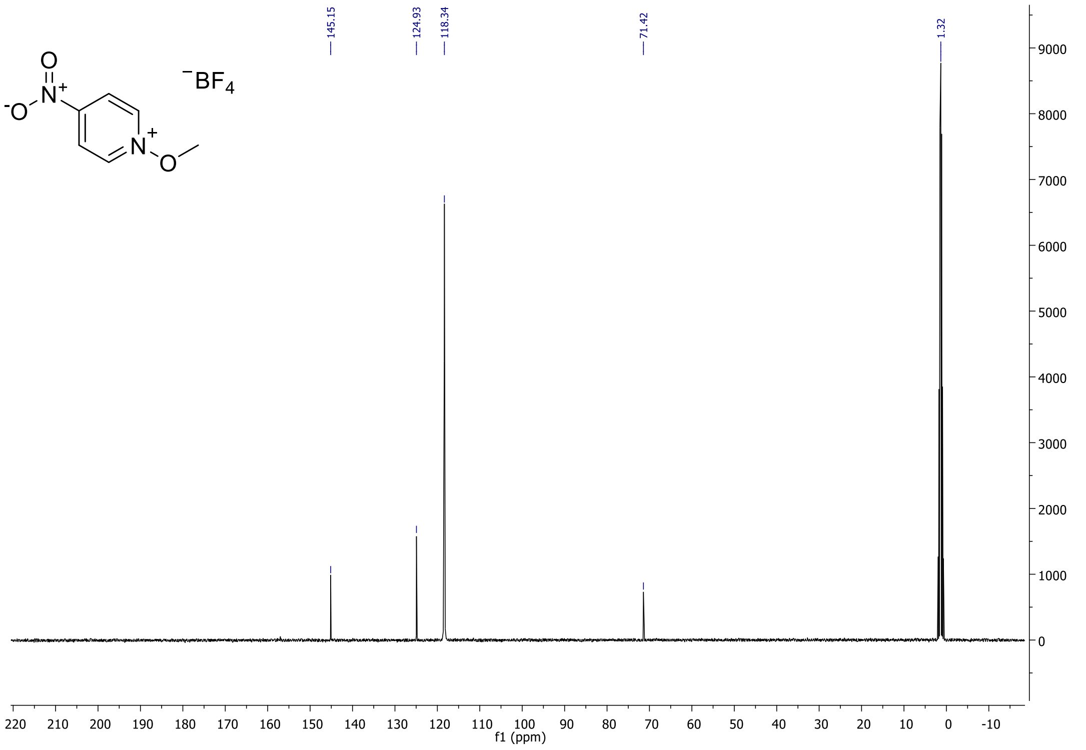

^{1}H NMR in CDCN.

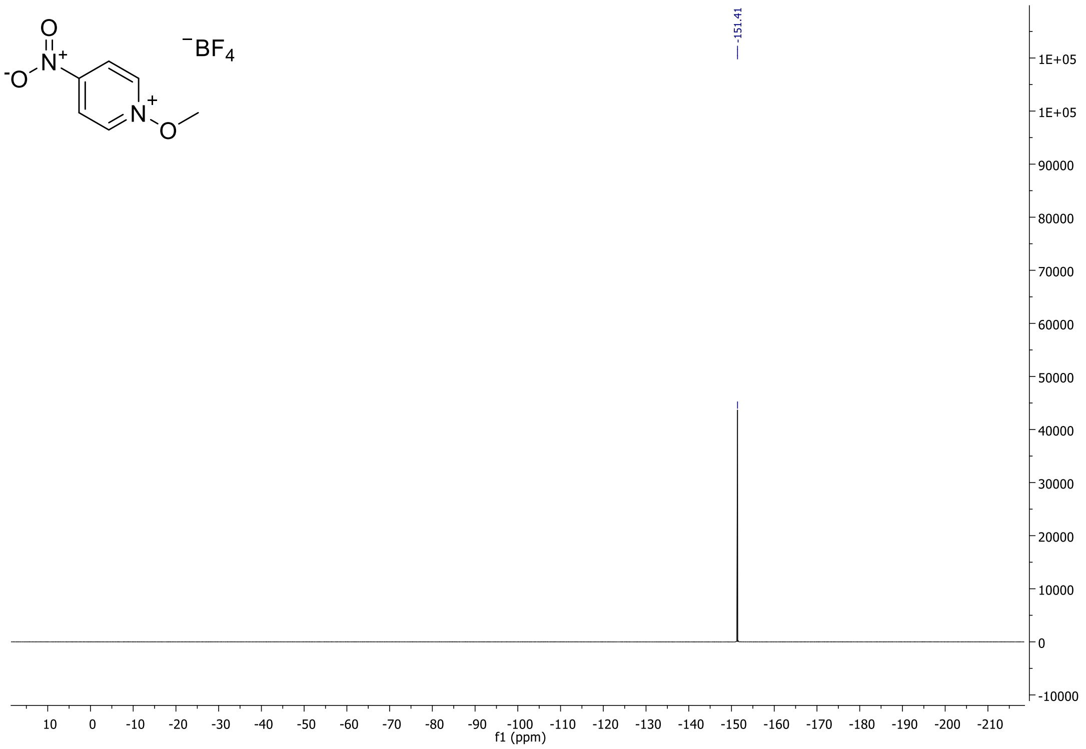

^{19}F NMR in CDCN.

NMR of products:

1-Ethylquinolin-2(1*H*)-one (3a)

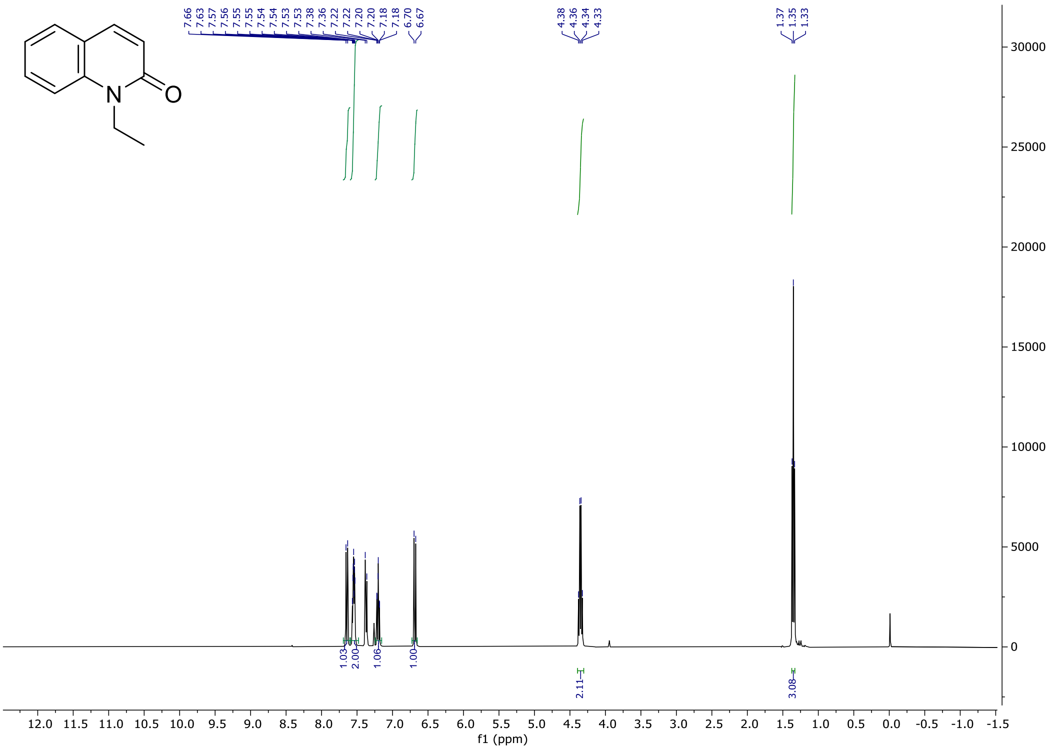

^{1}H NMR in $CDCl_3$.

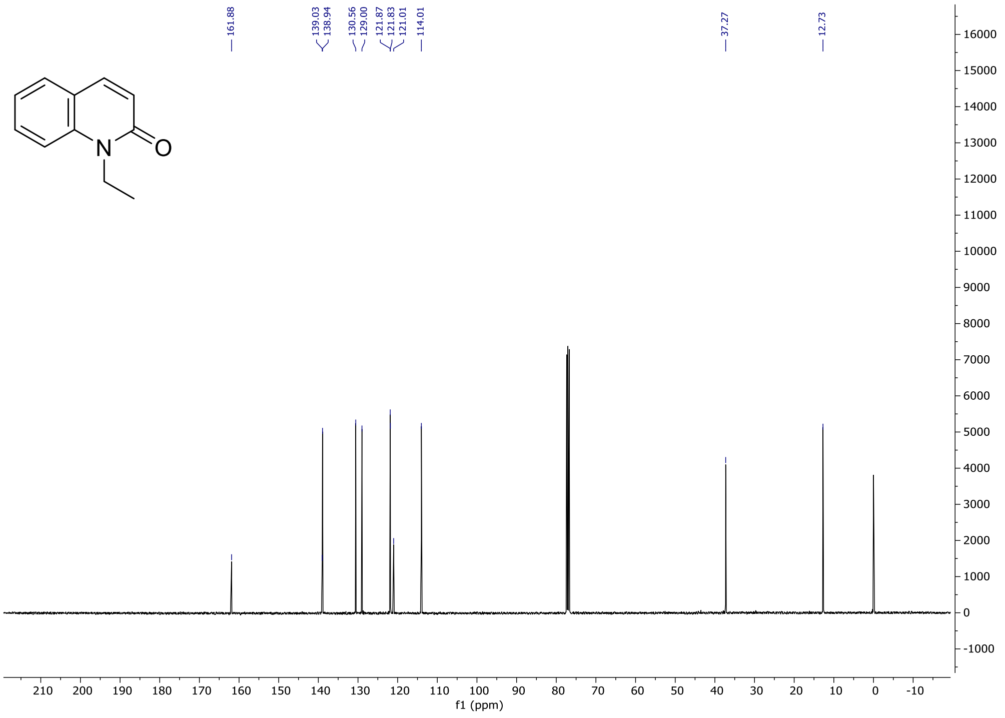

^{13}C NMR in $CDCl_3$.

1-Phenylquinolin-2(1*H*)-one (3b)

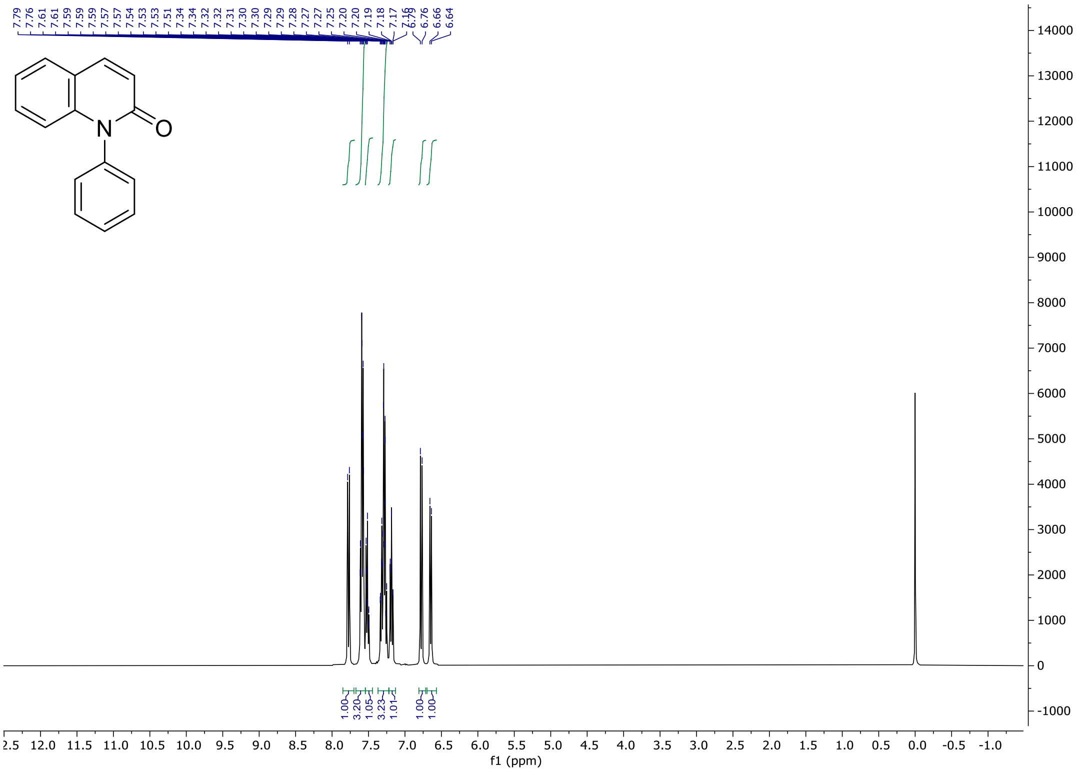

^{1}H NMR in $CDCl_3$.

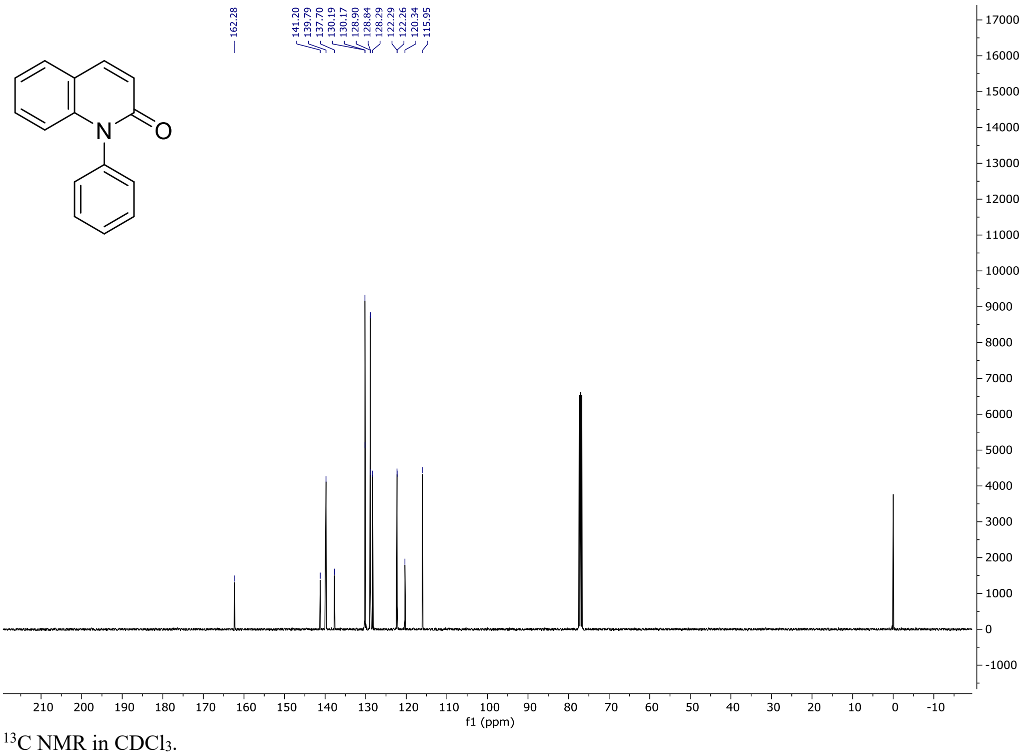

^{13}C NMR in $CDCl_3$.

1-(4-Methoxyphenyl)quinolin-2(1*H*)-one (3c)

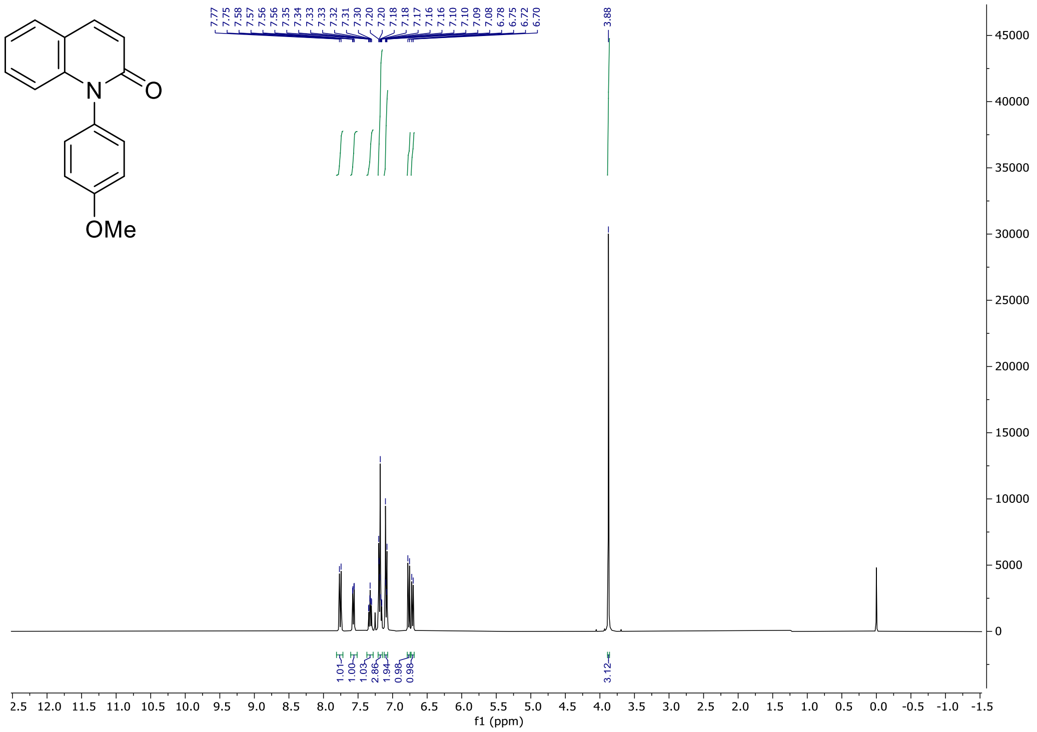

^{1}H NMR in $CDCl_3$.

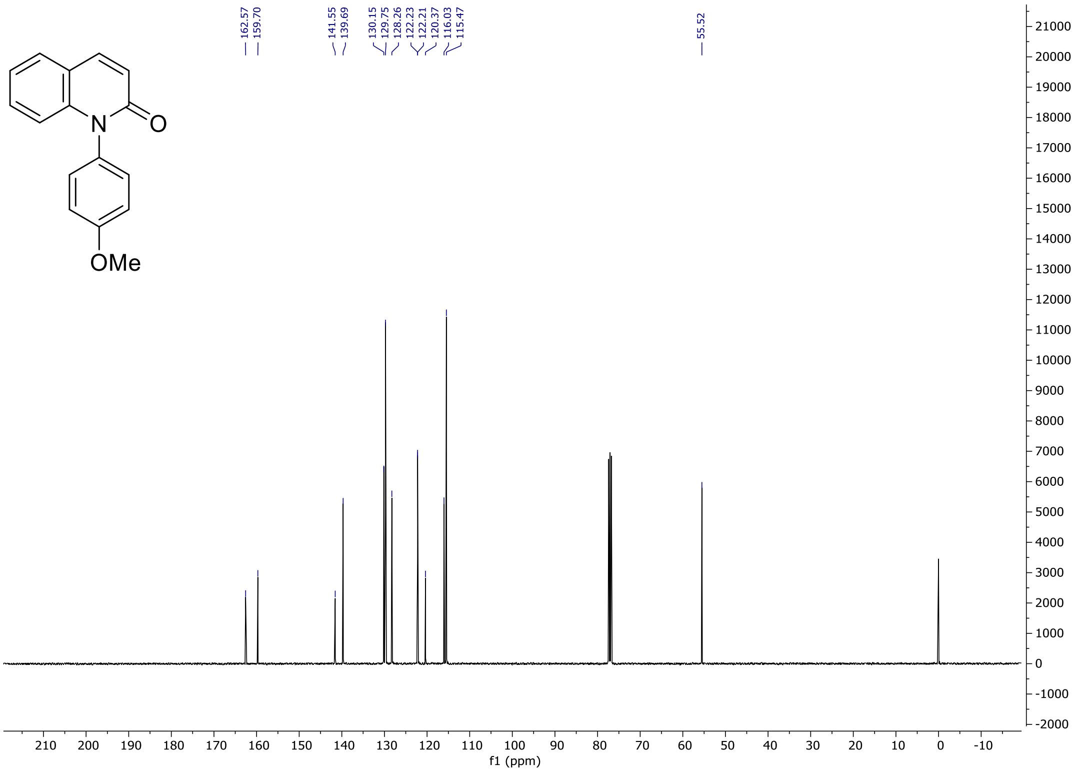

^{13}C NMR in $CDCl_3$.

1-(p-Tolyl)quinolin-2(1*H*)-one (3d)

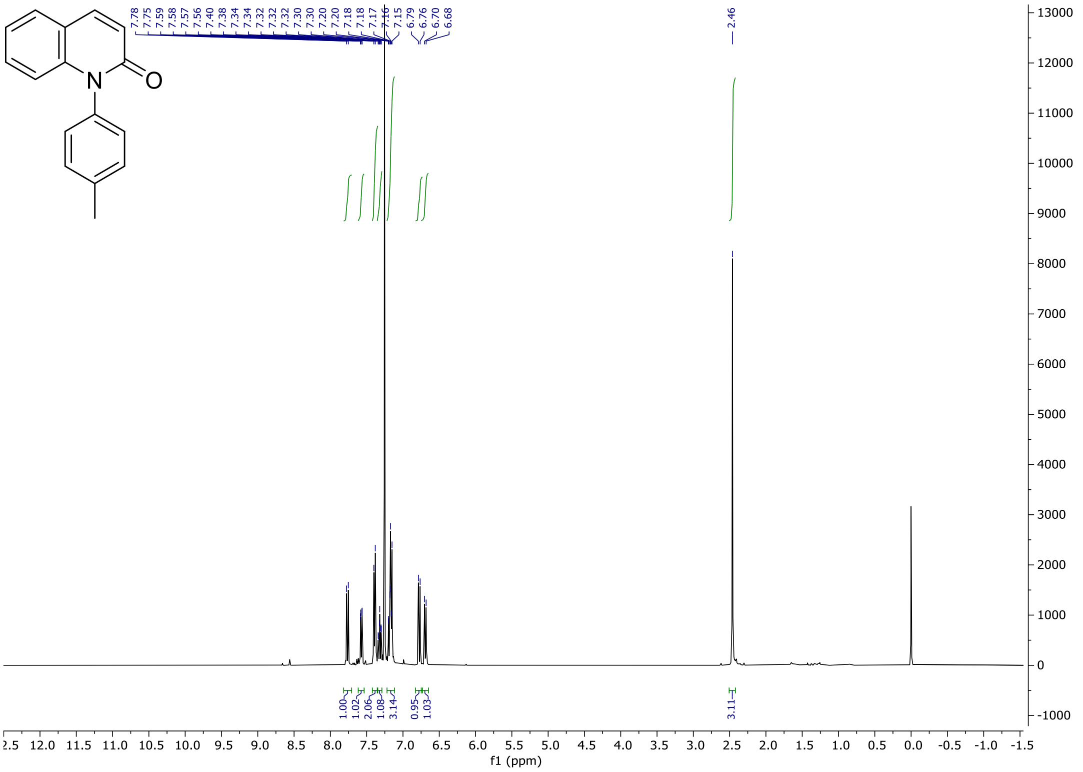

^{1}H NMR in $CDCl_3$.

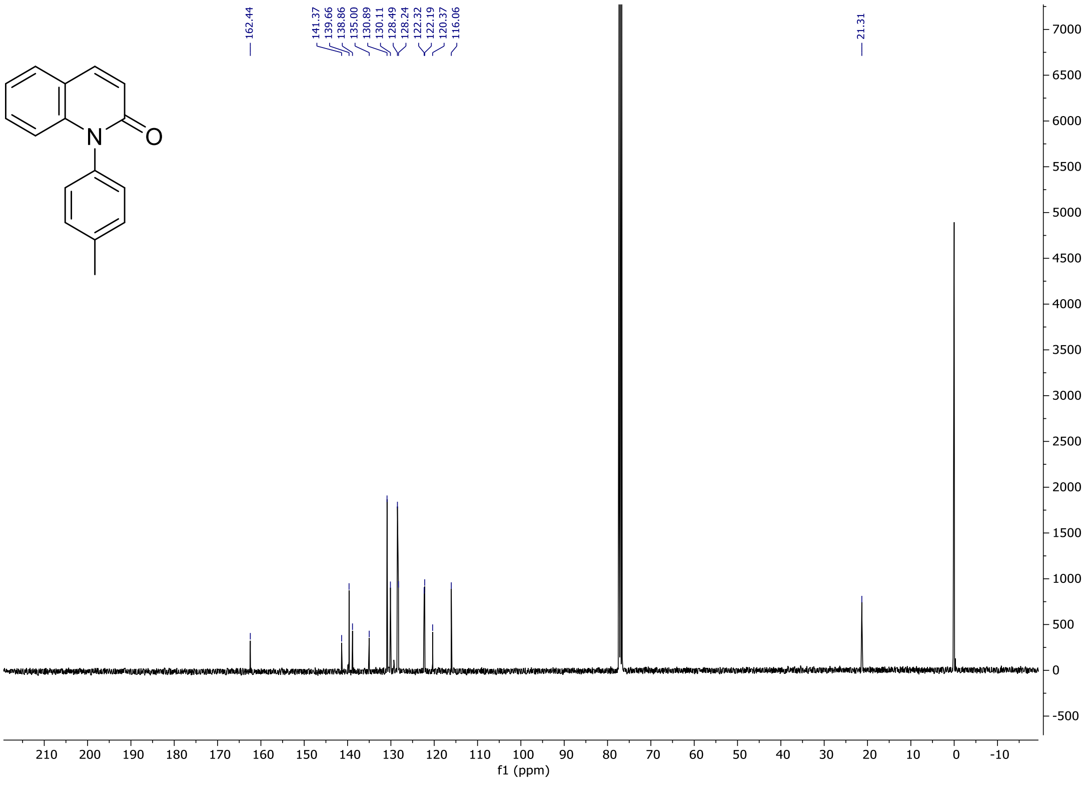

^{13}C NMR in CDCl$_3$.

1-(4-Fluorophenyl)quinolin-2(1*H*)-one (3e)

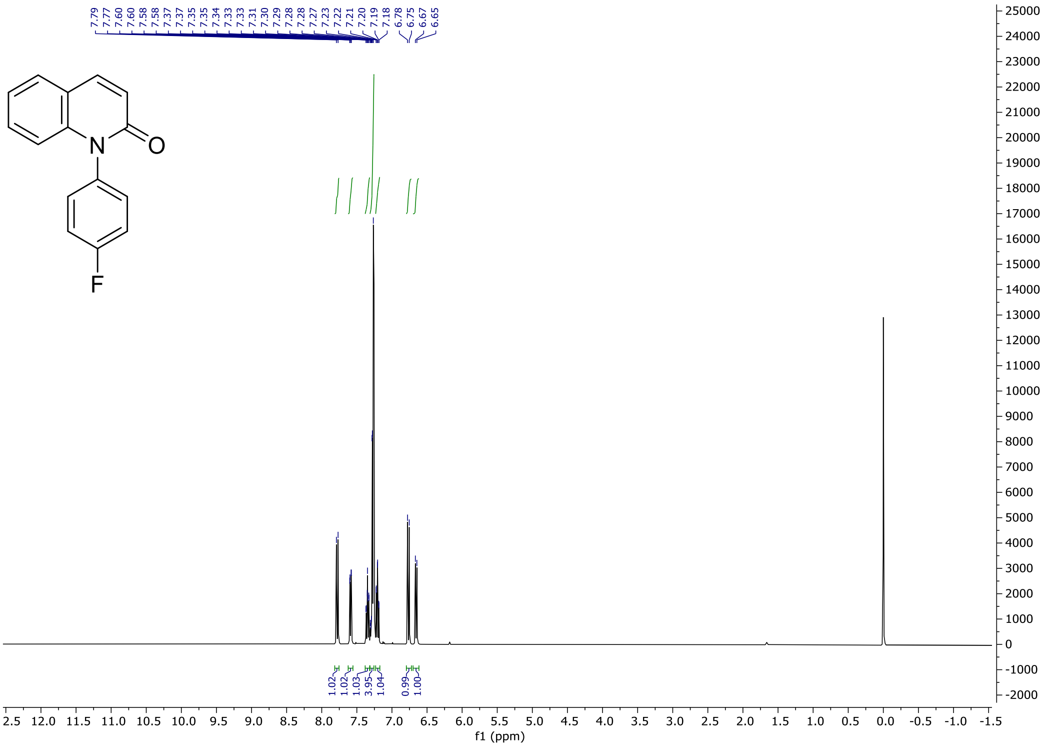

^{1}H NMR in CDCl$_3$.

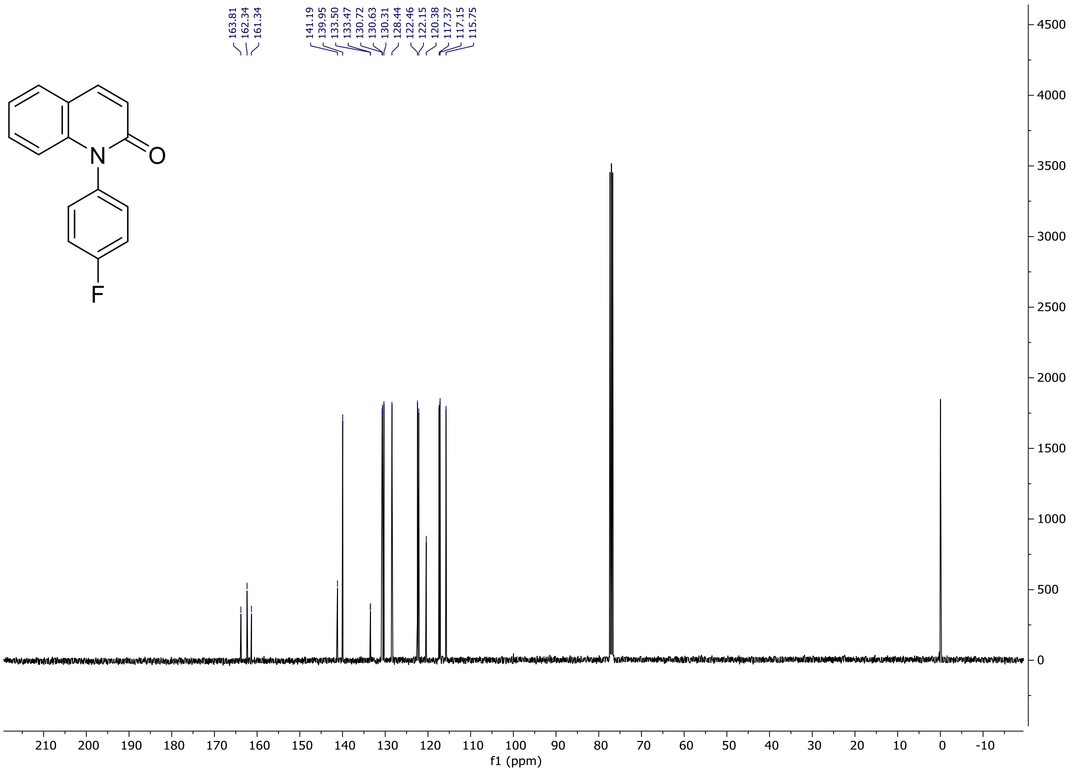

^{13}C NMR in CDCl$_3$.

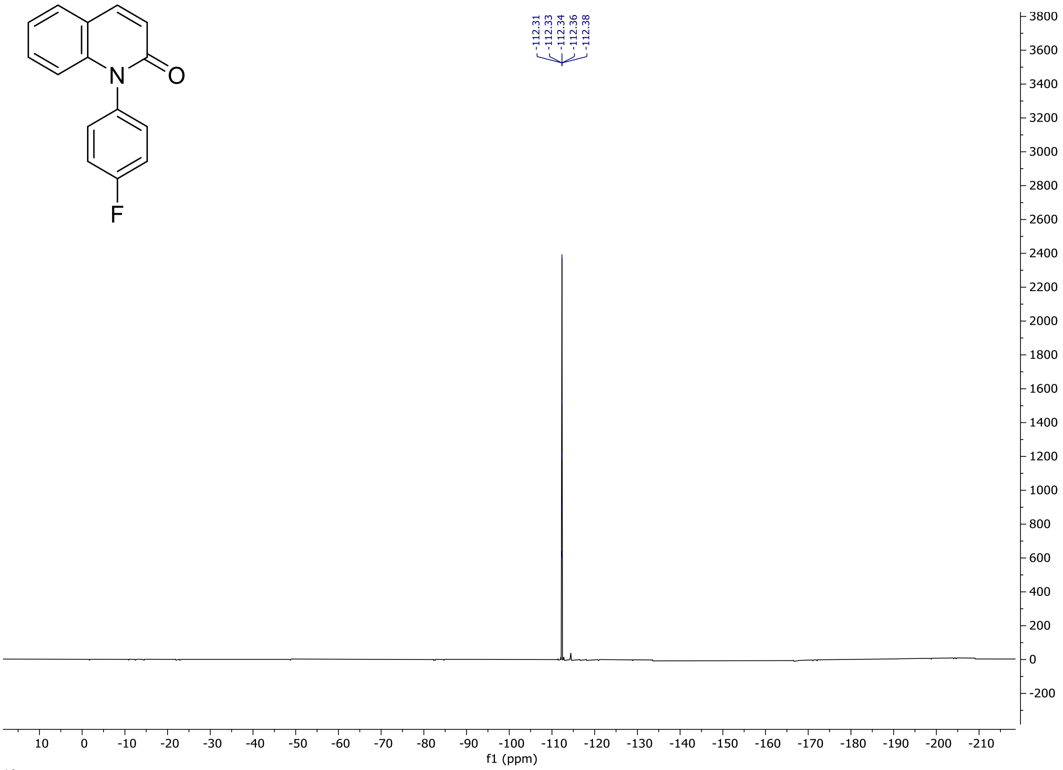

^{19}F NMR in $CDCl_3$.

1-(4-Cyanohenyl)quinolin-2(1*H*)-one (3f)

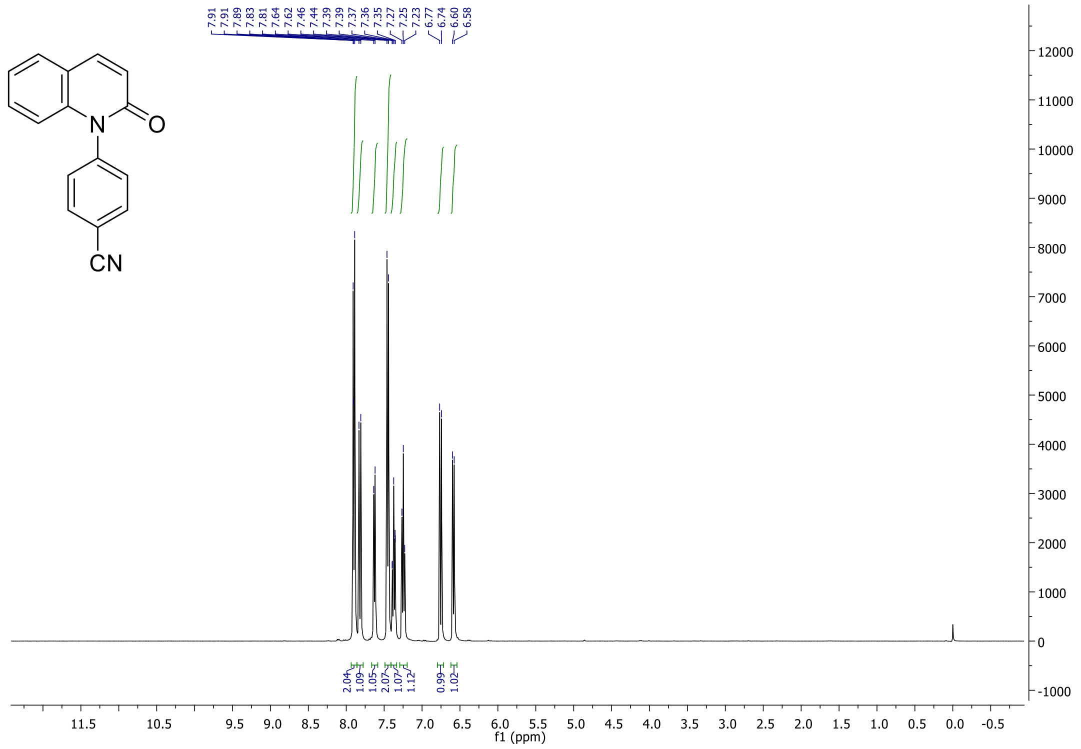

^{1}H NMR in $CDCl_3$.

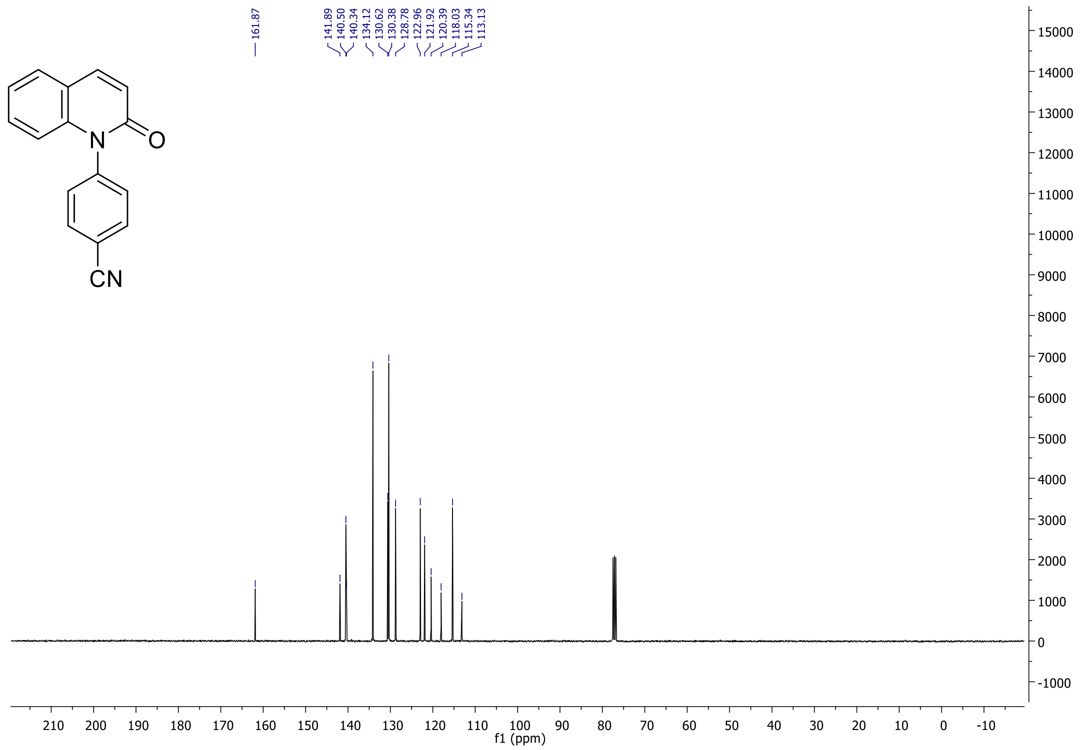

^{13}C NMR in $CDCl_3$.

1-(3-chlorophenyl)quinolin-2(1*H*)-one (3g)

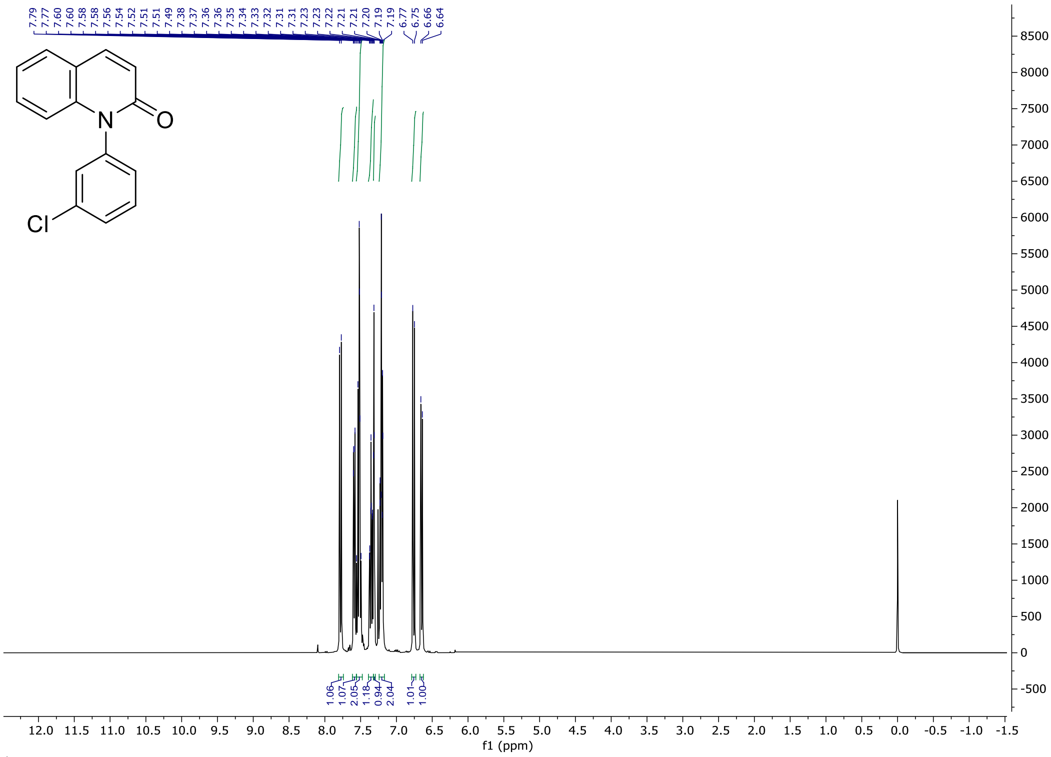

^{1}H NMR in $CDCl_3$.

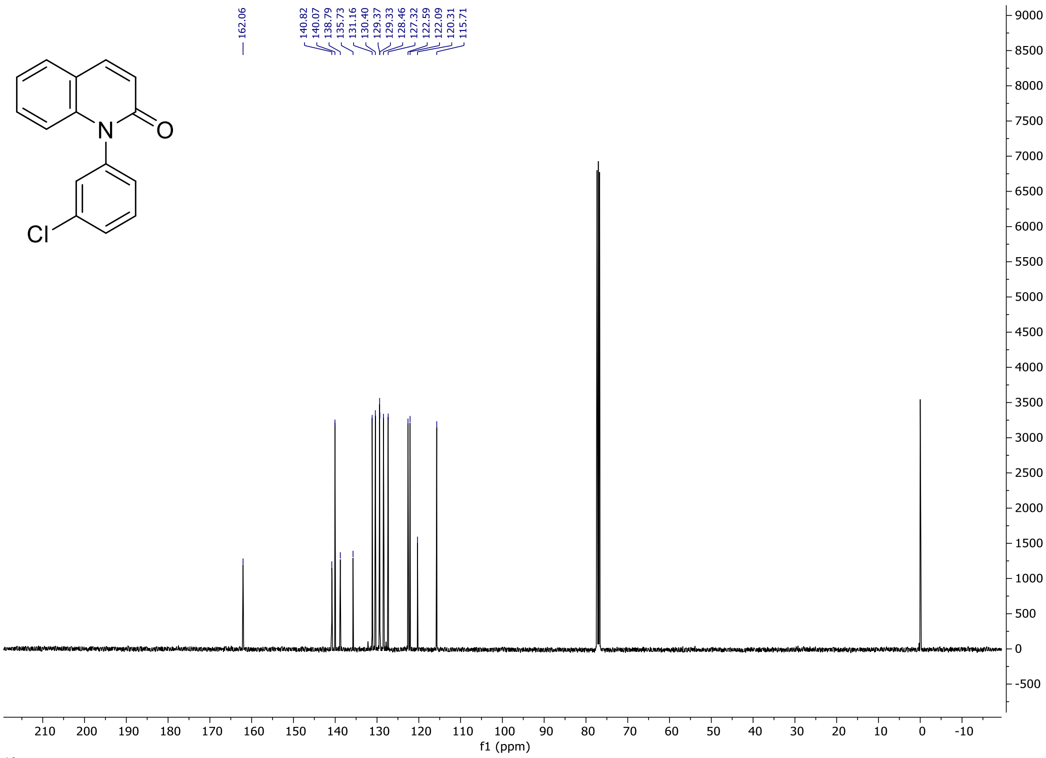

^{13}C NMR in $CDCl_3$.

6-Bromo-1-phenylquinolin-2(1*H*)-one (3h)

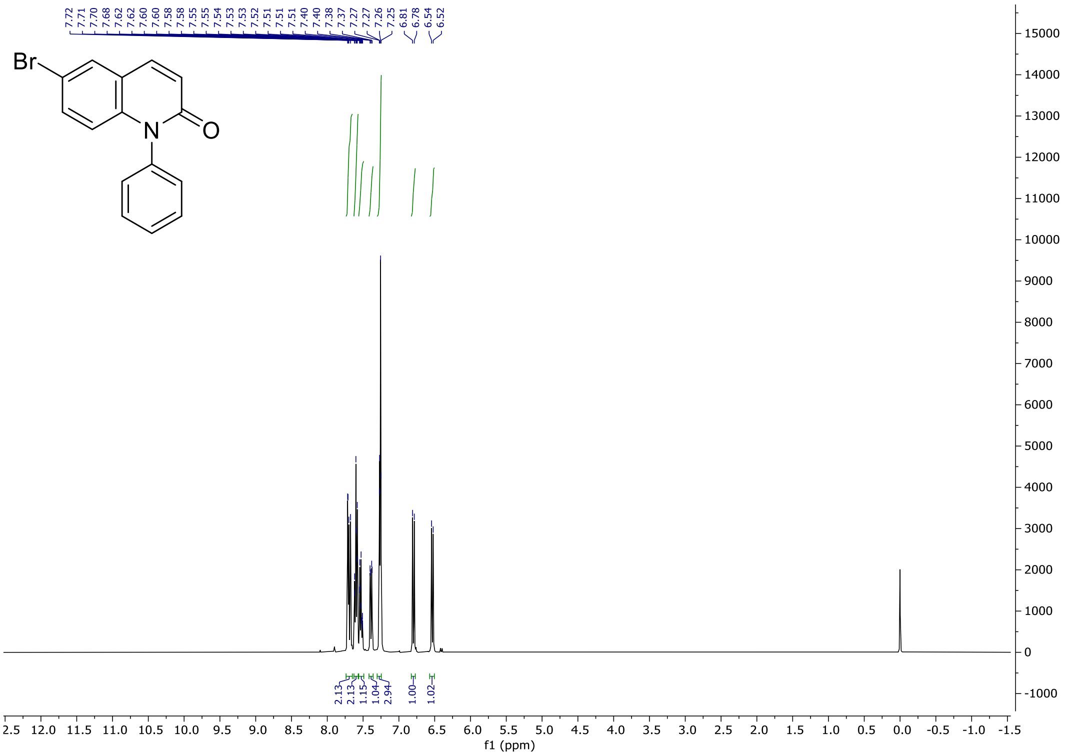

^{1}H NMR in $CDCl_3$.

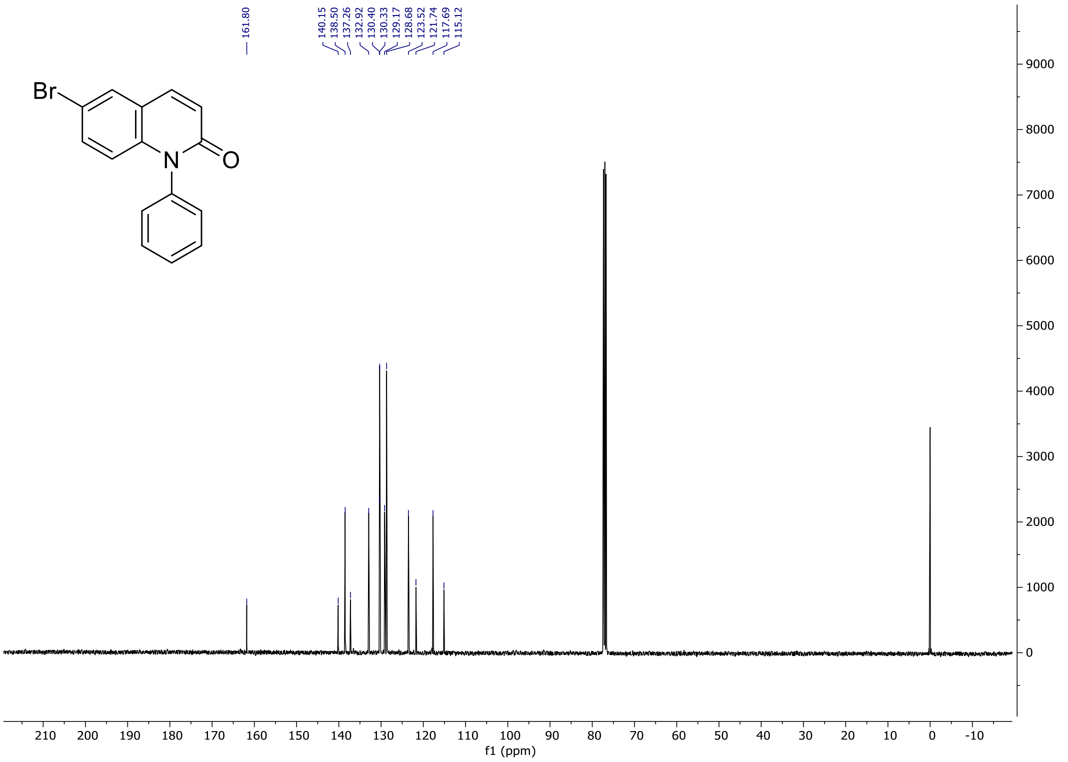

^{13}C NMR in $CDCl_3$.

Quinolin-2(1*H*)-one (3i)

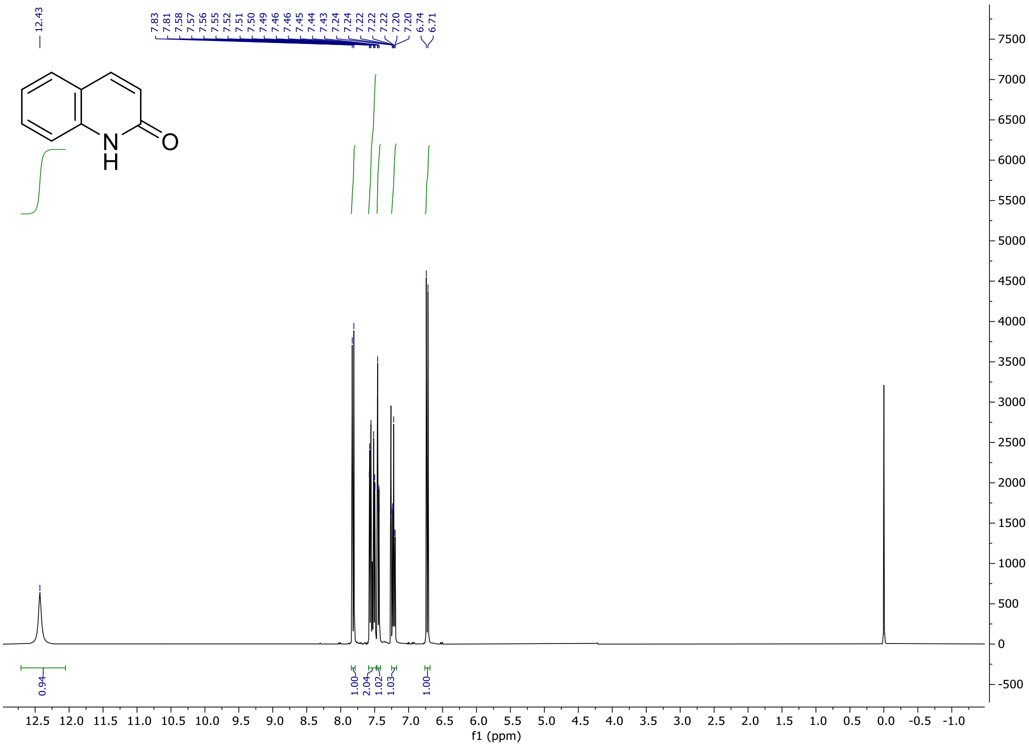

^{1}H NMR in $CDCl_3$.

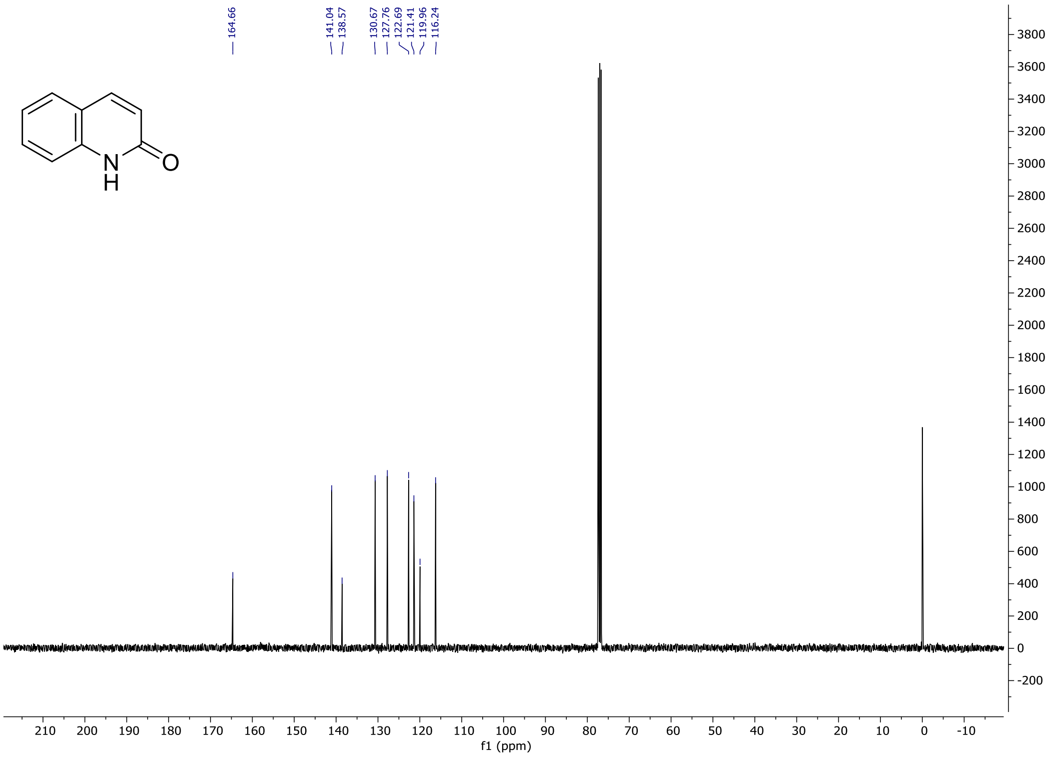

^{13}C NMR in $CDCl_3$.

6-Chloroquinolin-2(1*H*)-one (3j)

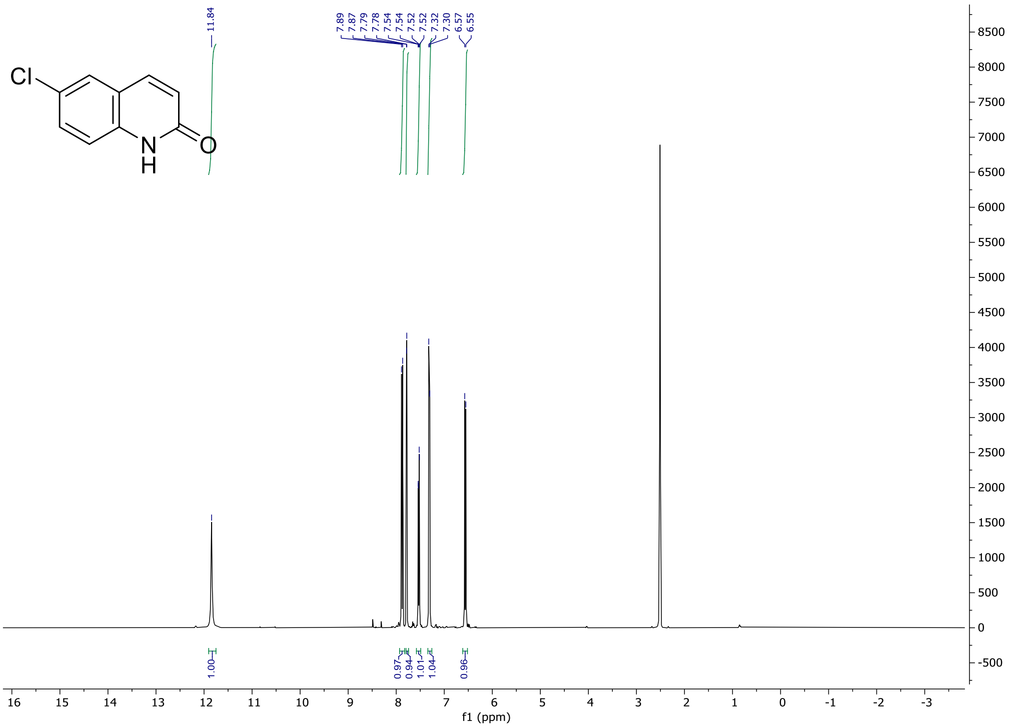

^{1}H NMR in DMSO-d6.

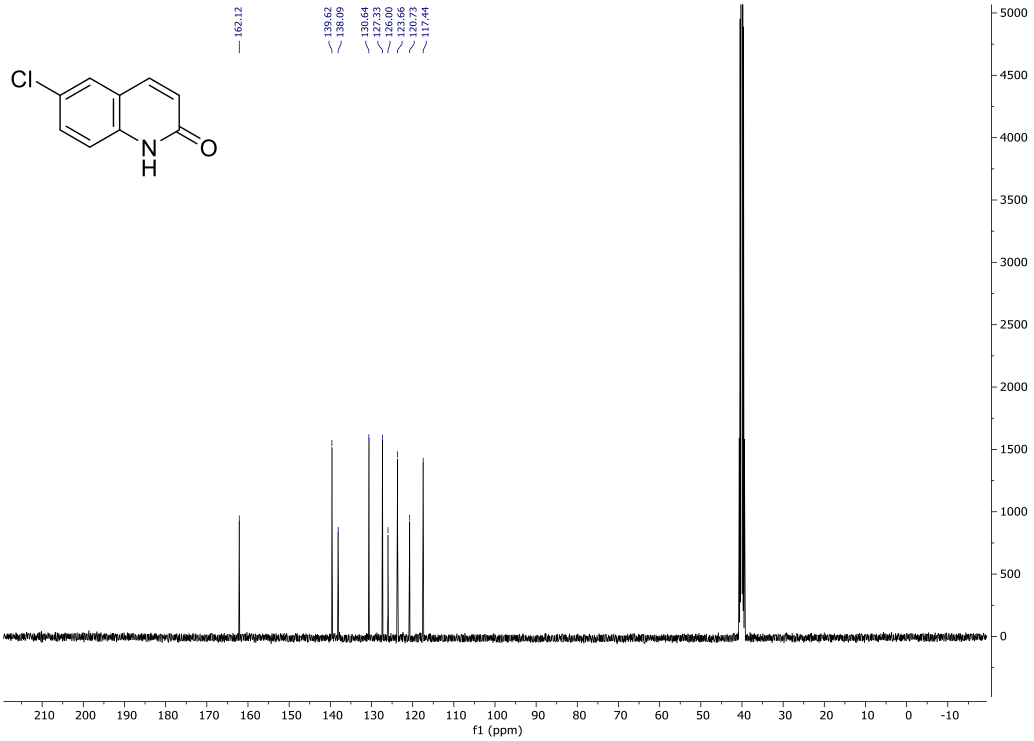

^{13}C NMR in $CDCl_3$.

7-Methoxyquinolin-2(1*H*)-one (3k)

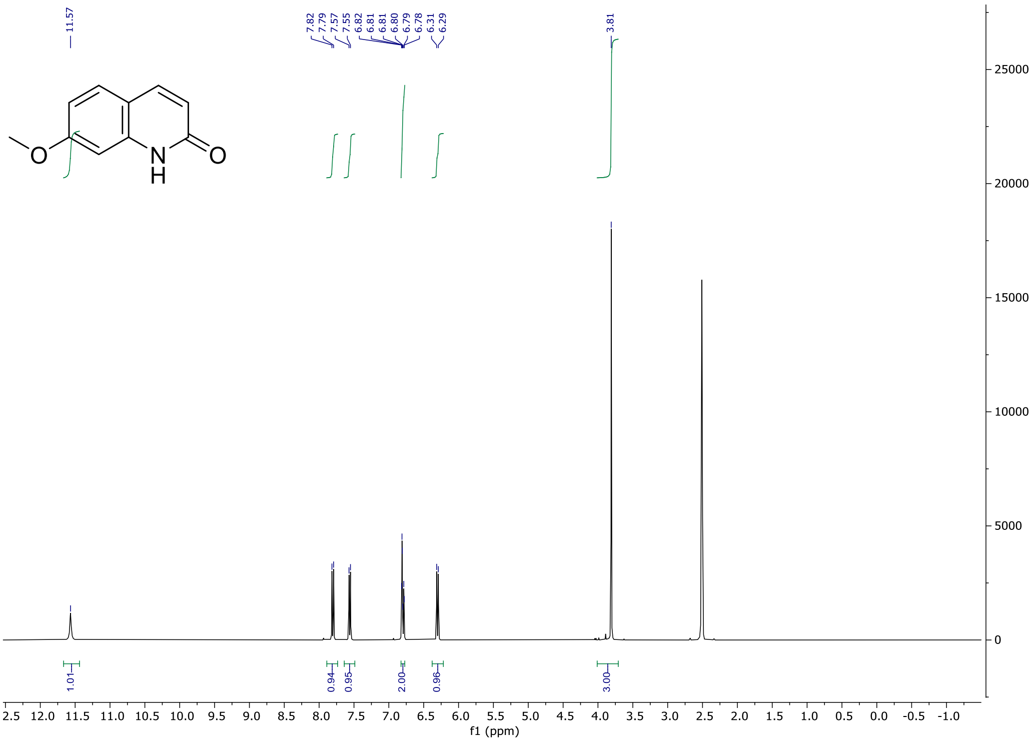

^{1}H NMR in DMSO-d6.

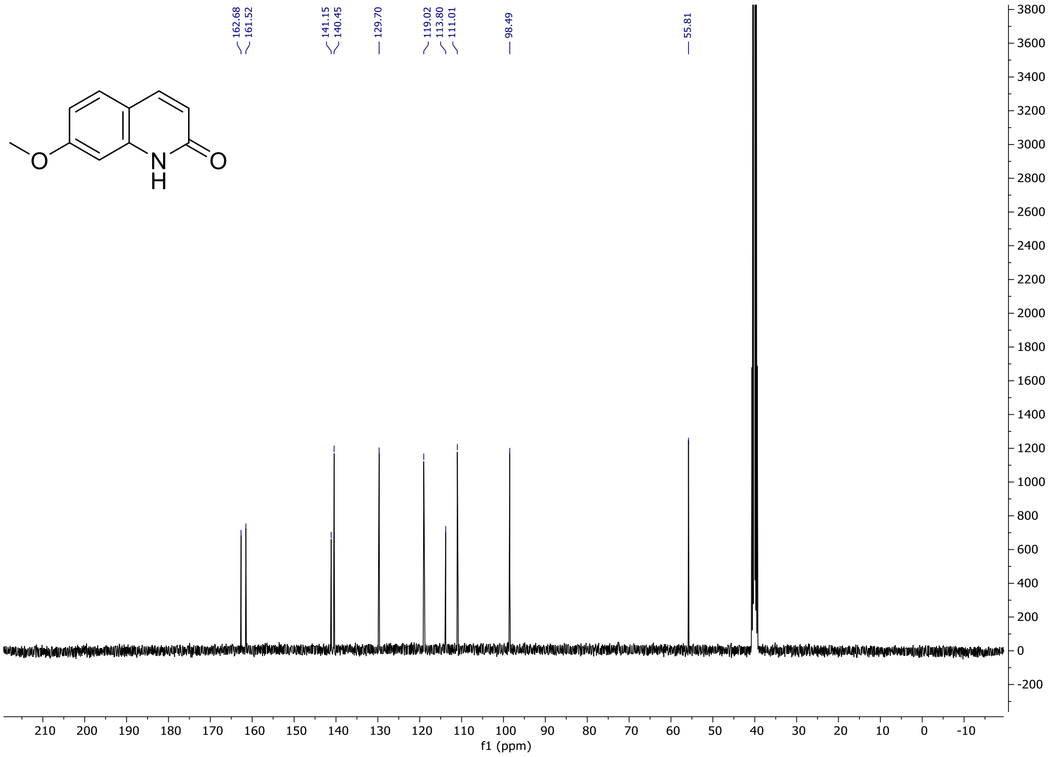

^{13}C NMR in $CDCl_3$.

7-(2-Oxo-2-phenylethoxy)quinolin-2(1*H*)-one (3l)

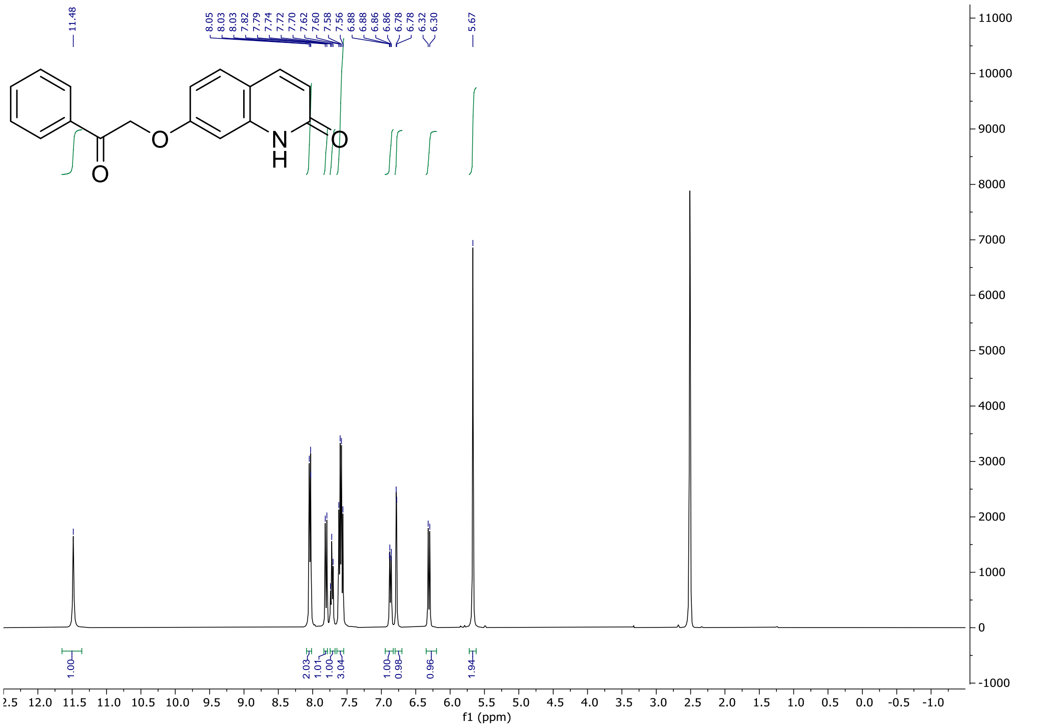

^{1}H NMR in DMSO-d6.

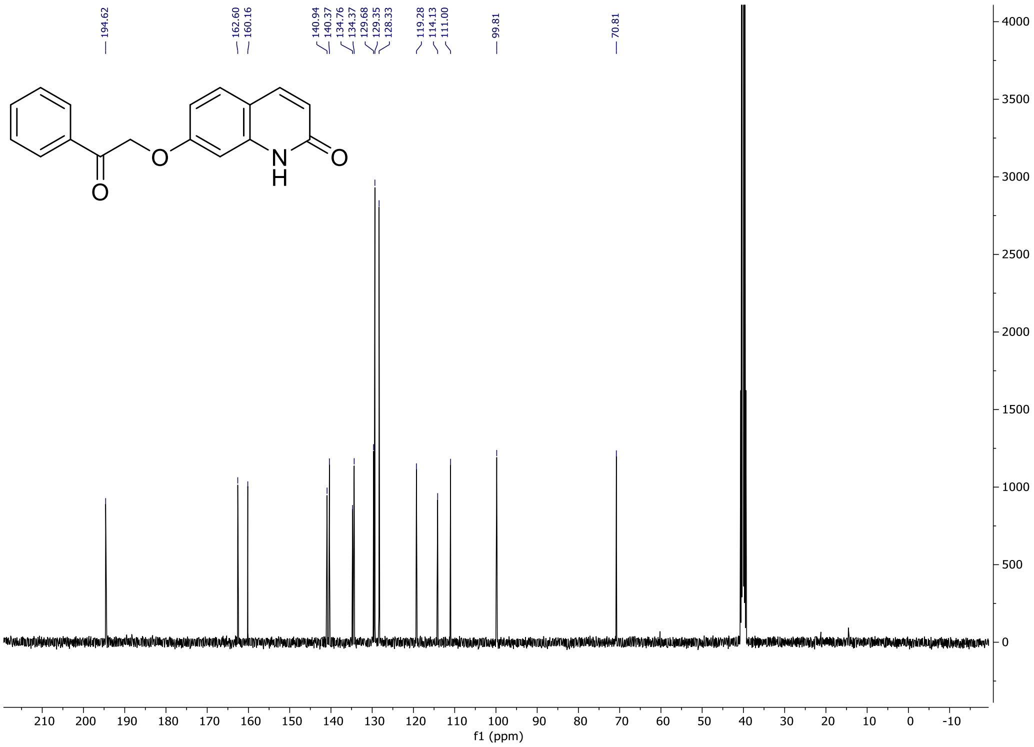

^{13}C NMR in $CDCl_3$.

8-Fluoro-3-methylquinolin-2(*1H*)-one (3m)

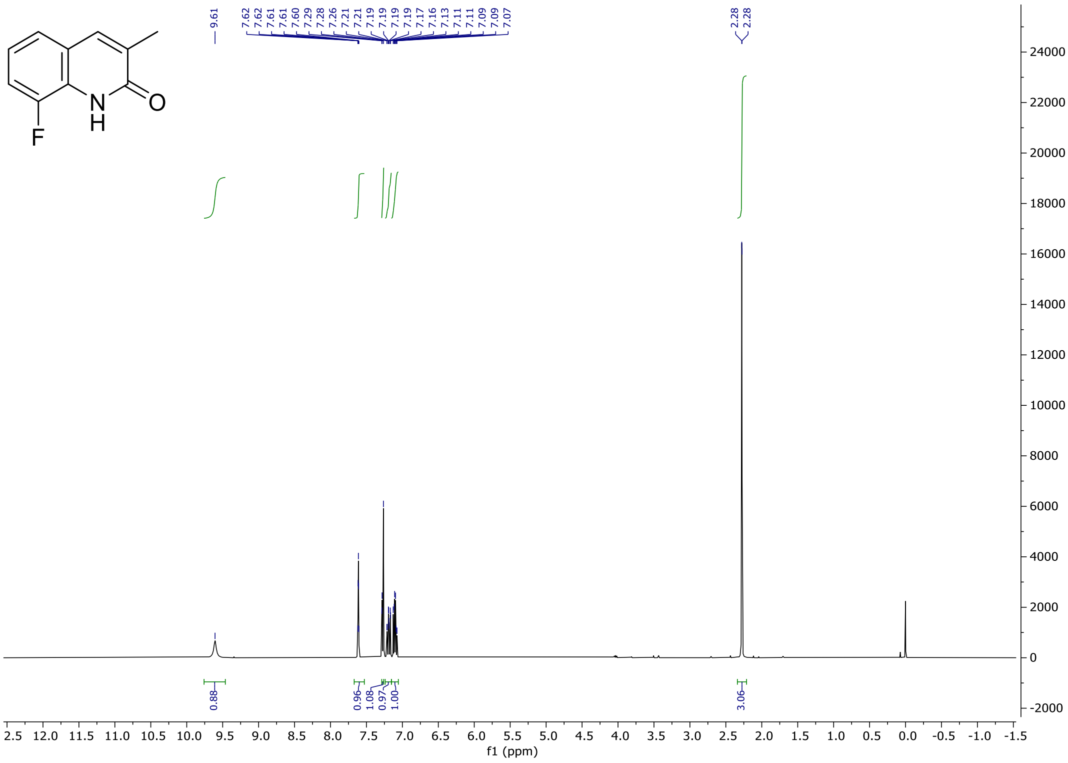

^{1}H NMR in $CDCl_3$.

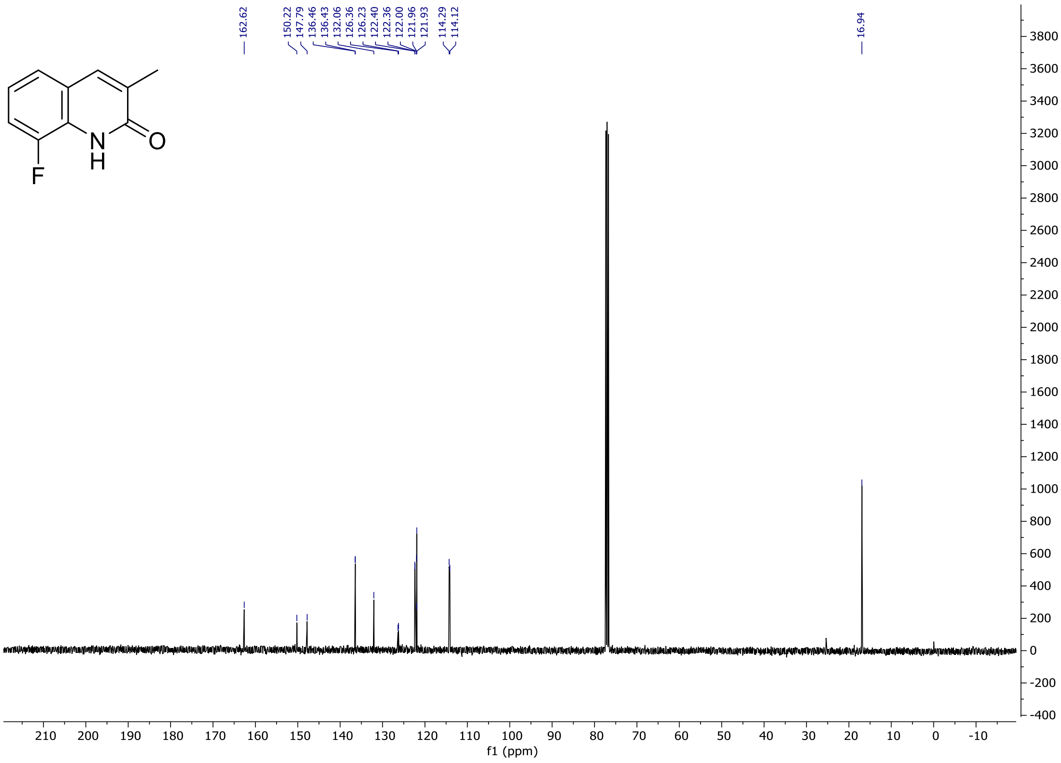

^{13}C NMR in $CDCl_3$.

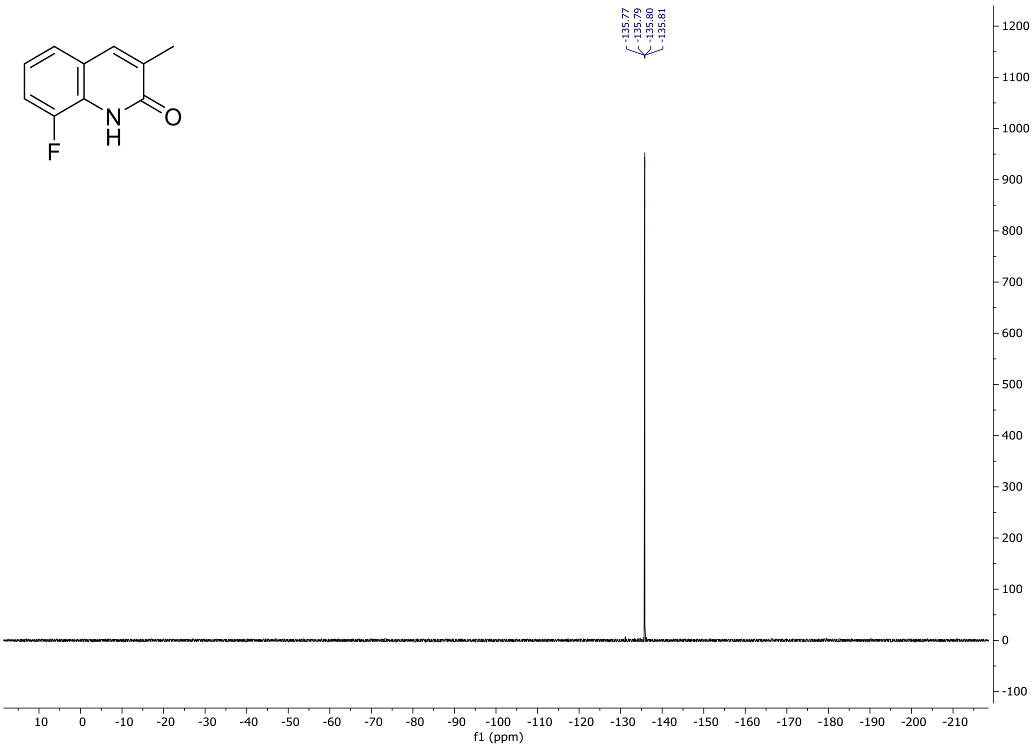

^{19}F NMR in $CDCl_3$.

7-(4-(4-(2,3-Dichlorophenyl)piperazin-1-yl)butoxy)quinolin-2(1*H*)-one (3n)

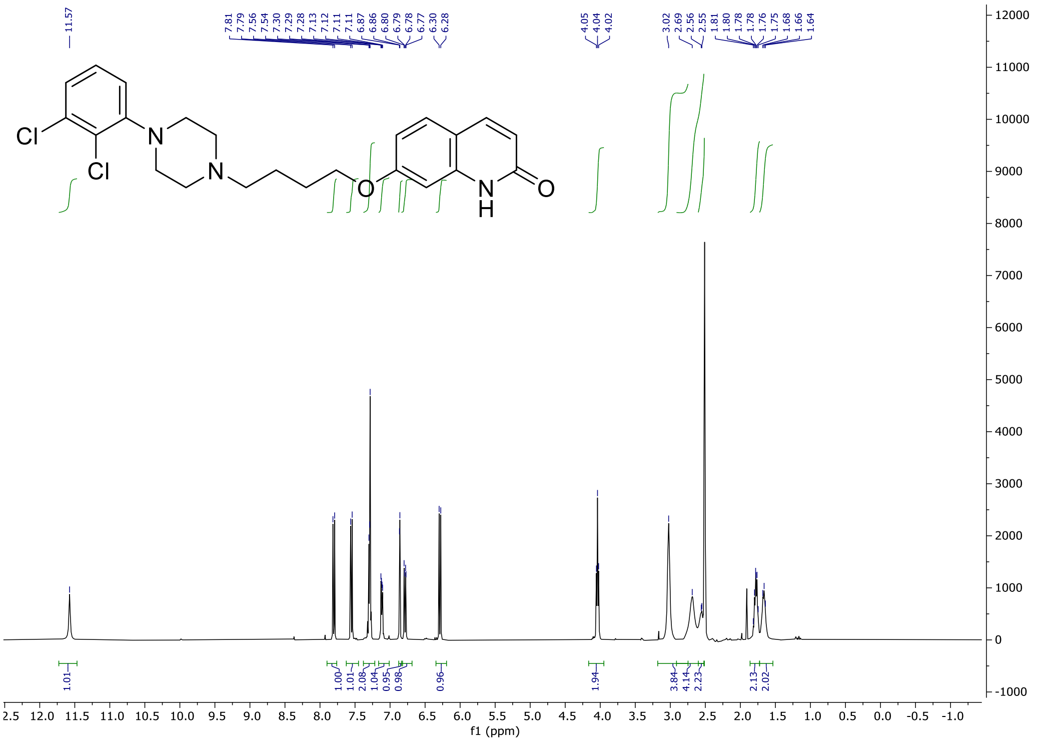

^{1}H NMR in DMSO-d6.

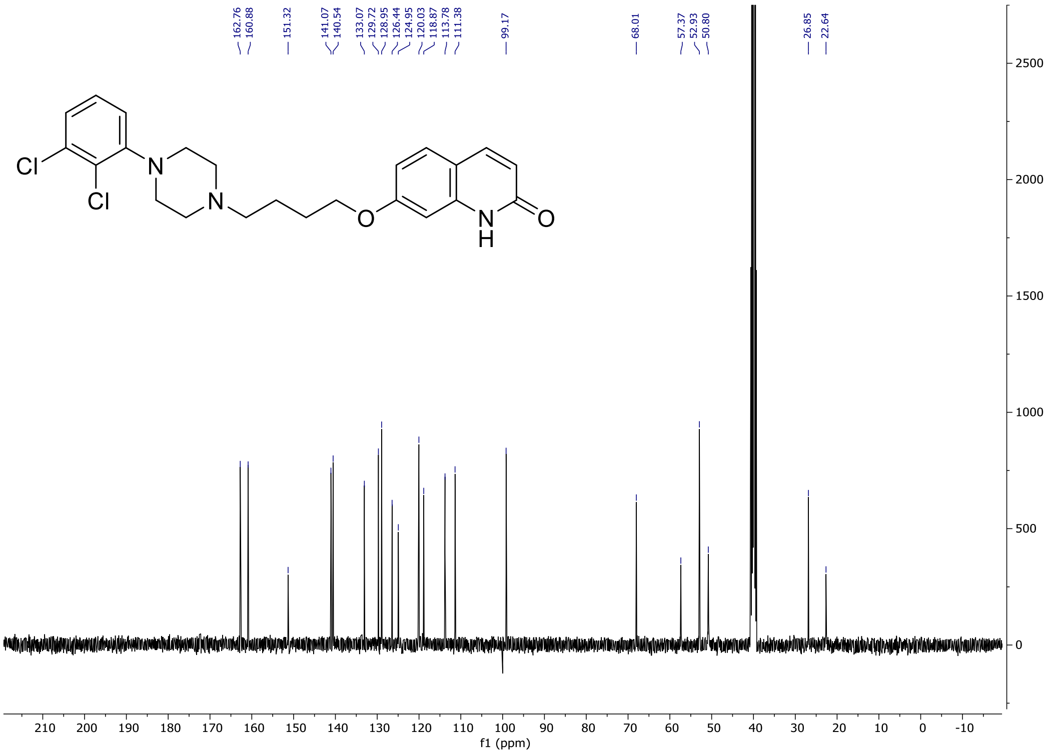

^{13}C NMR in $CDCl_3$.

6-(4-(1-Cyclohexyl-1*H*-tetrazol-5-yl)butoxy)quinolin-2(1*H*)-one (3o)

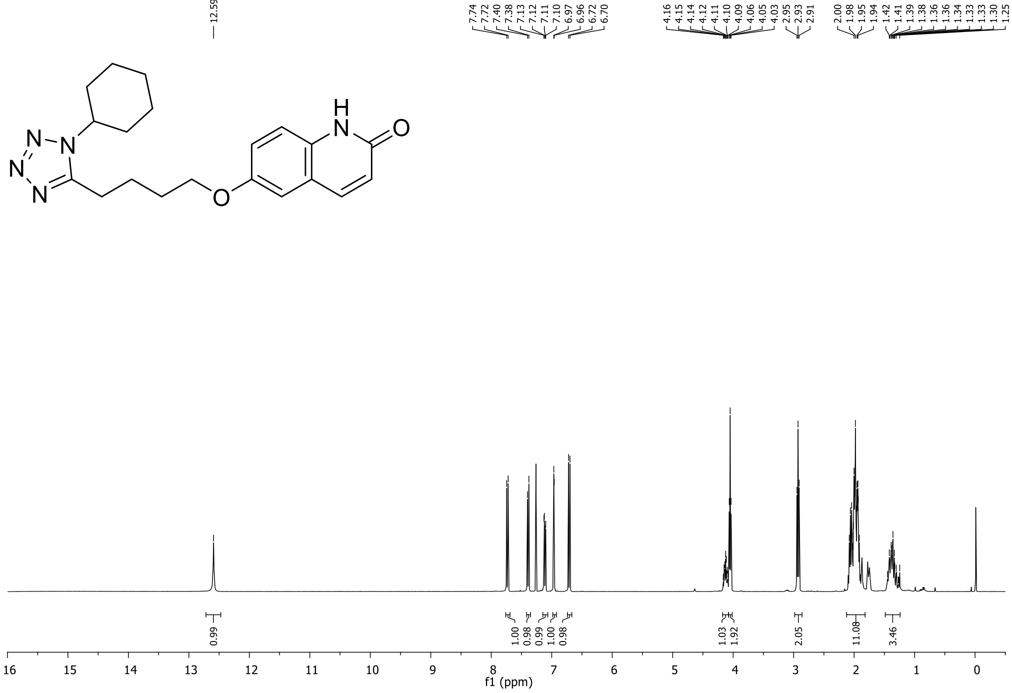

^{1}H NMR in $CDCl_3$.

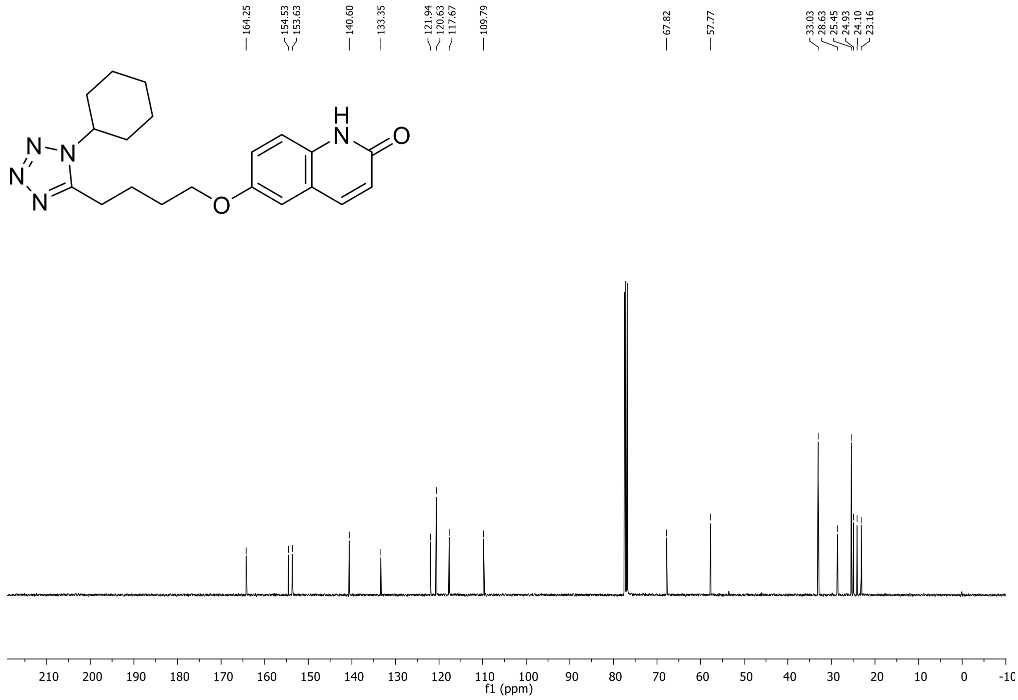

^{13}C NMR in $CDCl_3$

2-(4-Isobutylphenyl)-*N*-(2-oxo-1,2-dihydroquinolin-7-yl)propenamide (3p)

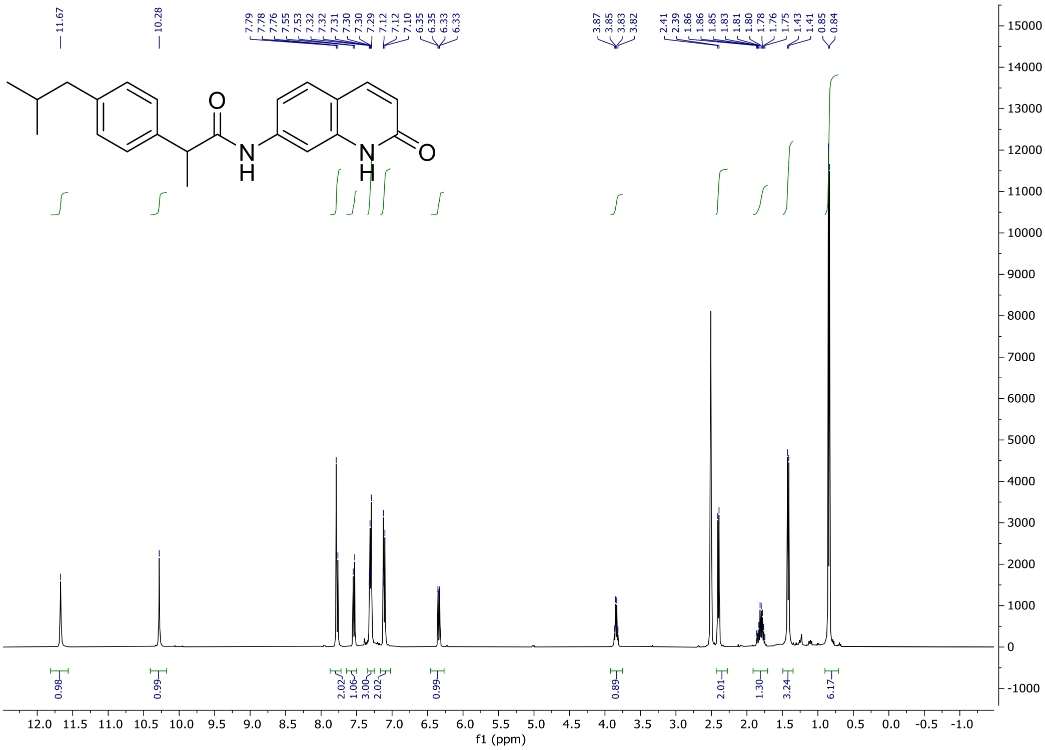

^{1}H NMR in DMSO-d6.

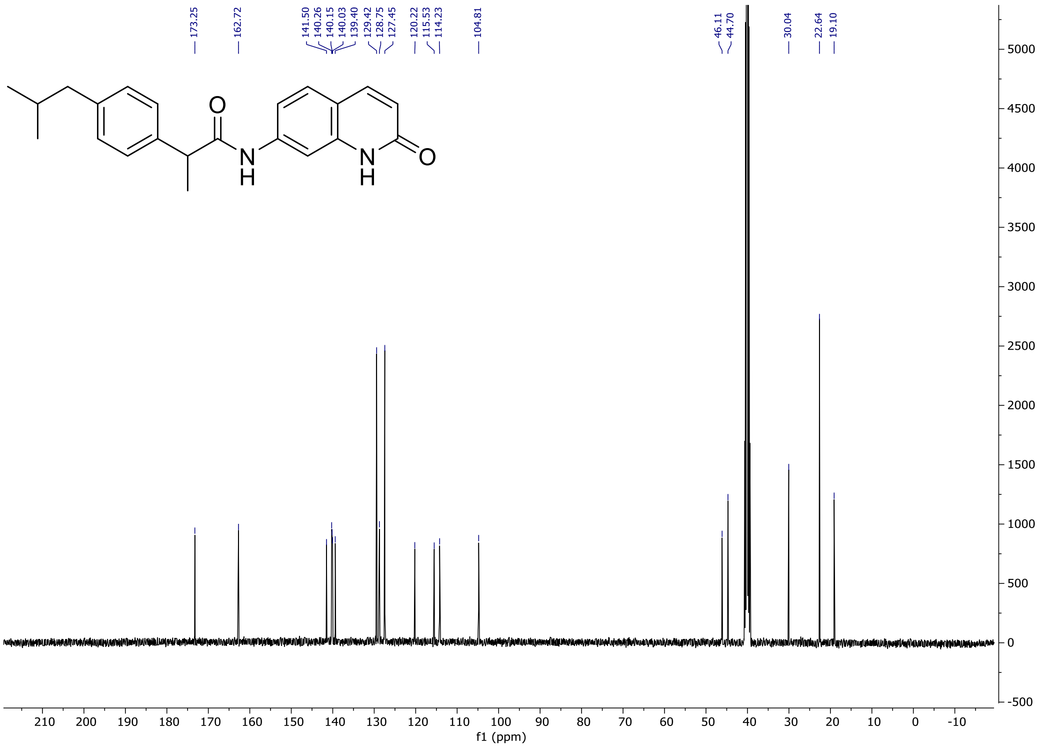

^{13}C NMR in DMSO-d6.

2-(3-Benzoylphenyl)-*N*-(2-oxo-1,2-dihydroquinolin-7-yl)propanamide (3q)

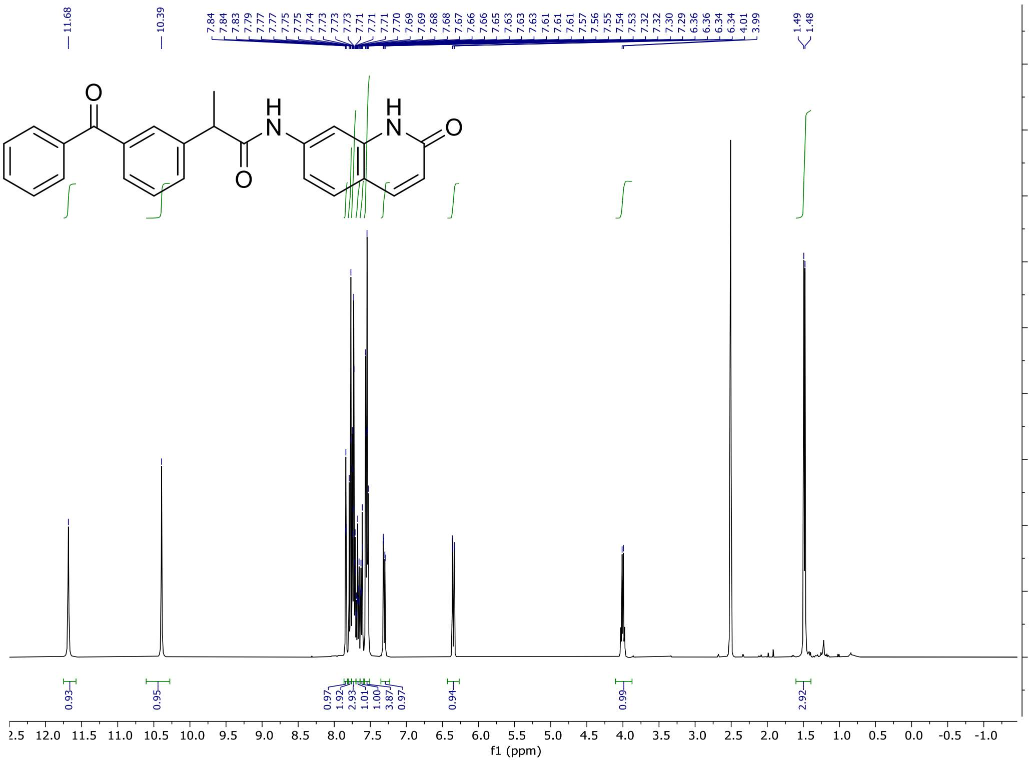

^{1}H NMR in DMSO-d6.

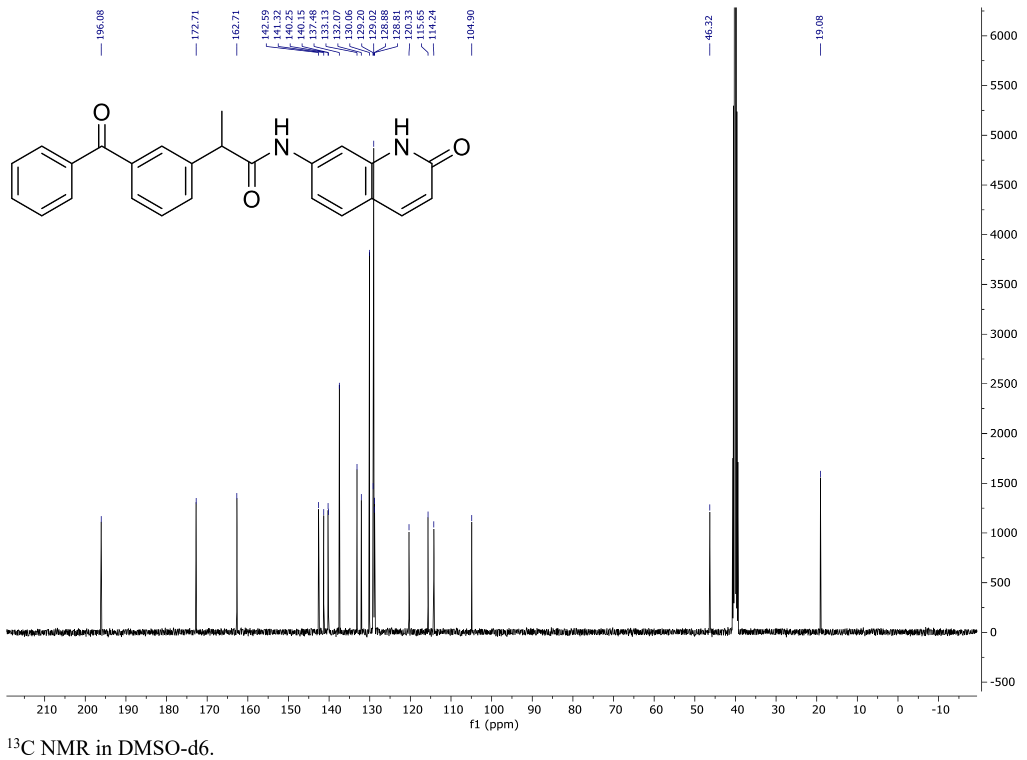

^{13}C NMR in DMSO-d6.

Tert-butyl (1-oxo-1-((2-oxo-1,2-dihydroquinolin-7-yl)amino)-3-phenylpropan-2-yl)carbamate (3r)

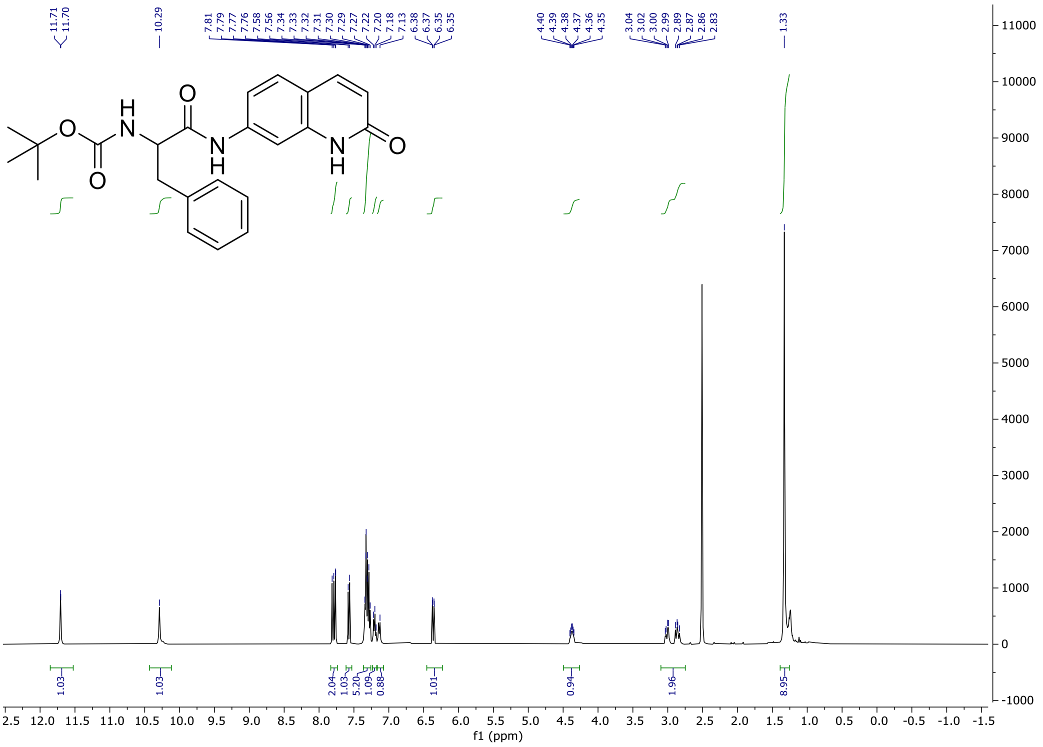

^{1}H NMR in $CDCl_3$.

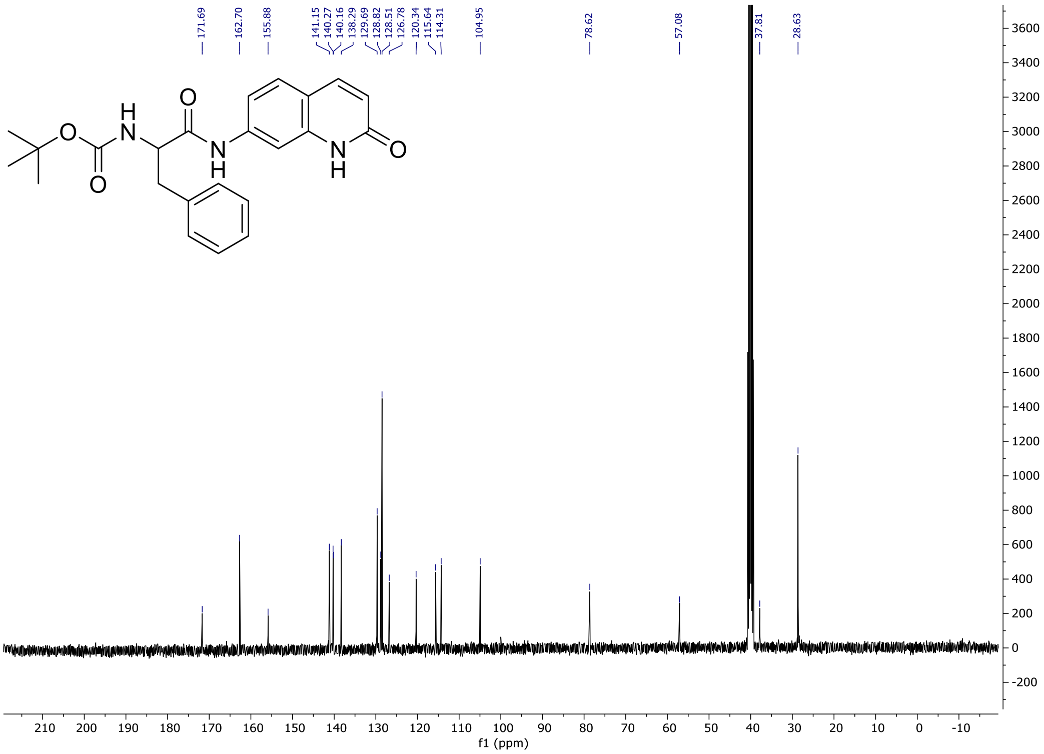

^{13}C NMR in $CDCl_3$.

1-Methyl-2-oxo-1,2-dihydroquinolin-7-yl 2-((1R,3R)-3-acetyl-2,2-dimethylcyclobutyl)acetate (3s)

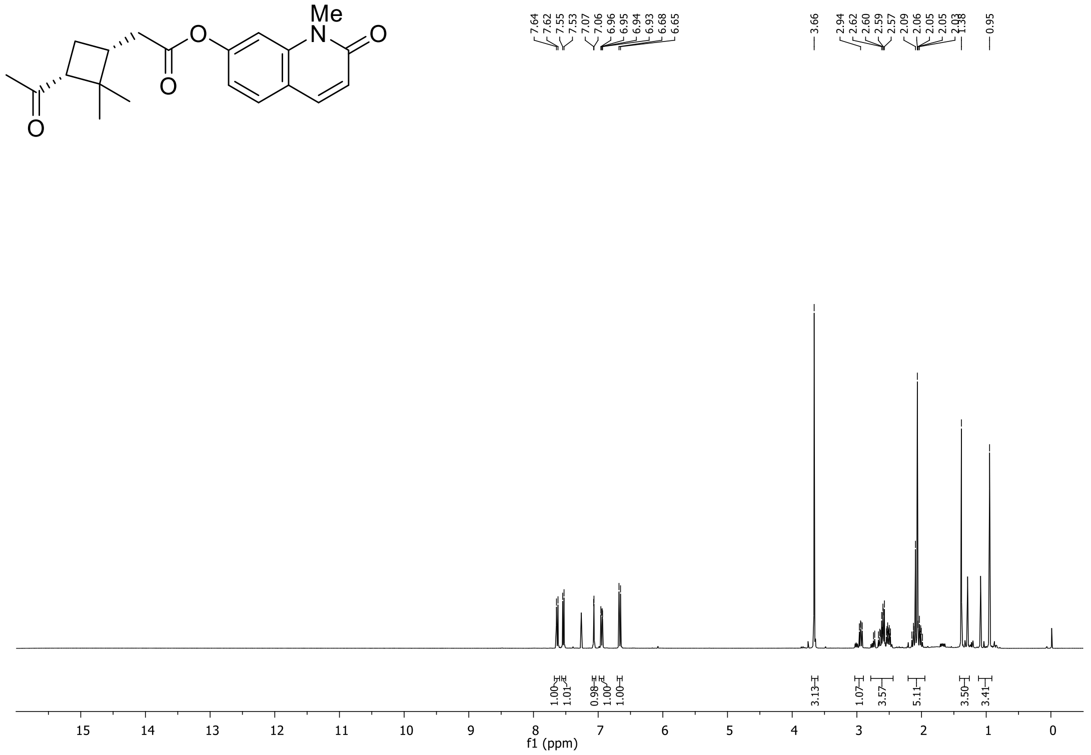

^{1}H NMR in $CDCl_3$.

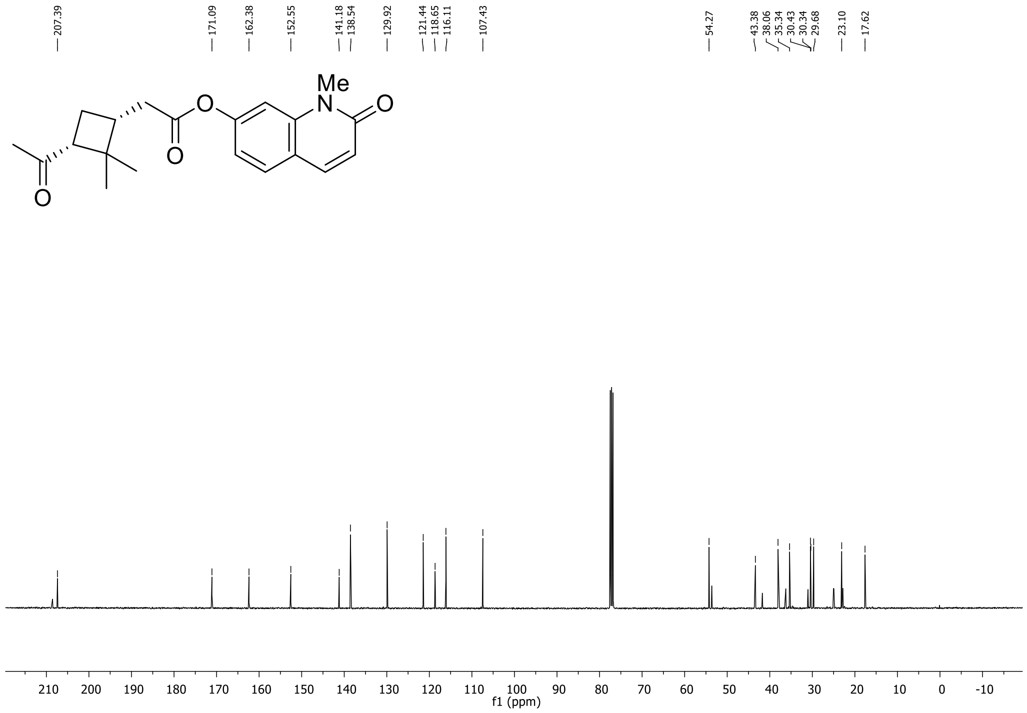

^{13}C NMR in $CDCl_3$

2*H*-Chromen-2-one (5a)

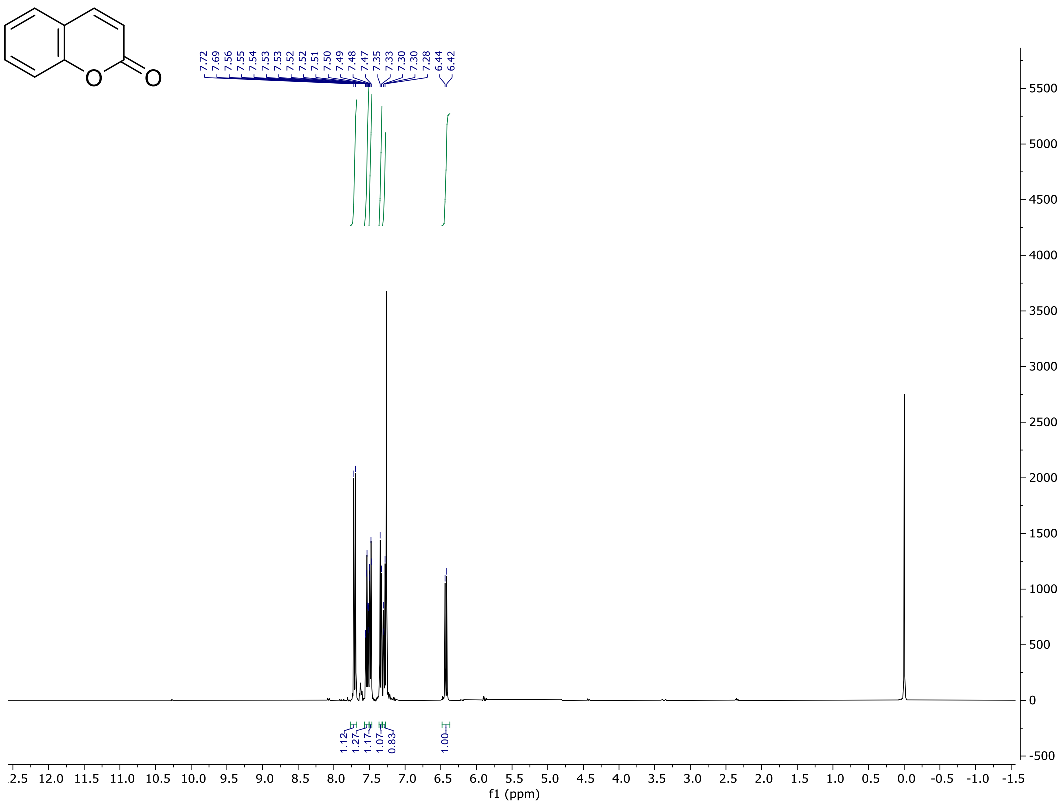

^{1}H NMR in $CDCl_3$.

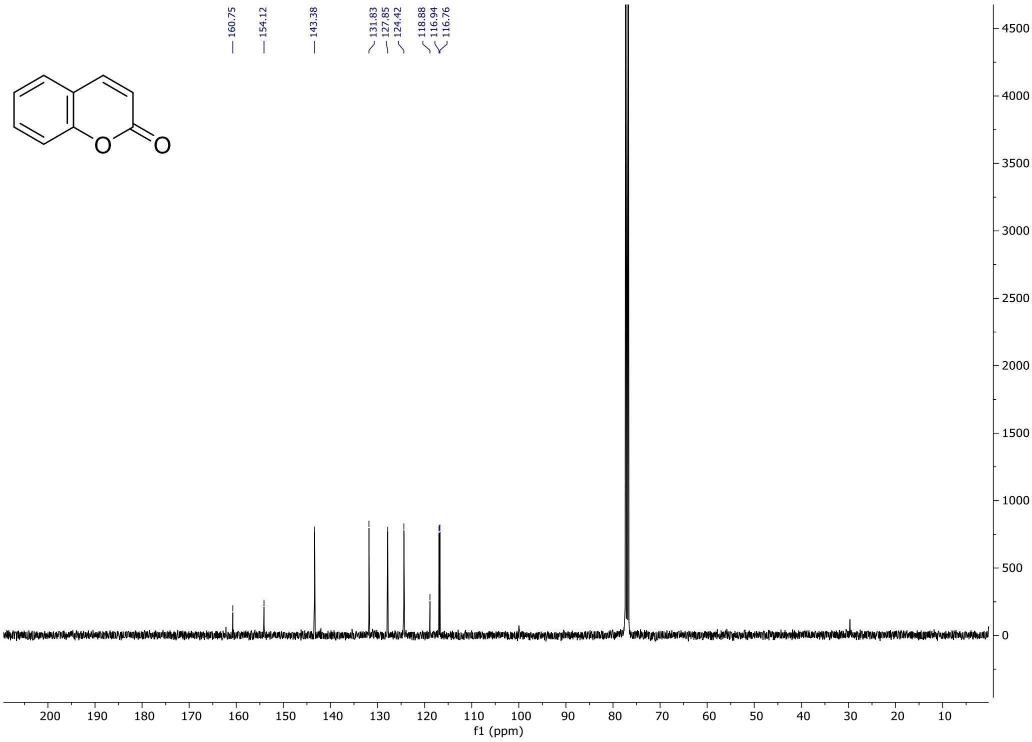

^{13}C NMR in $CDCl_3$.

3*H*-Benzo[*f*]chromen-3-one (5b)

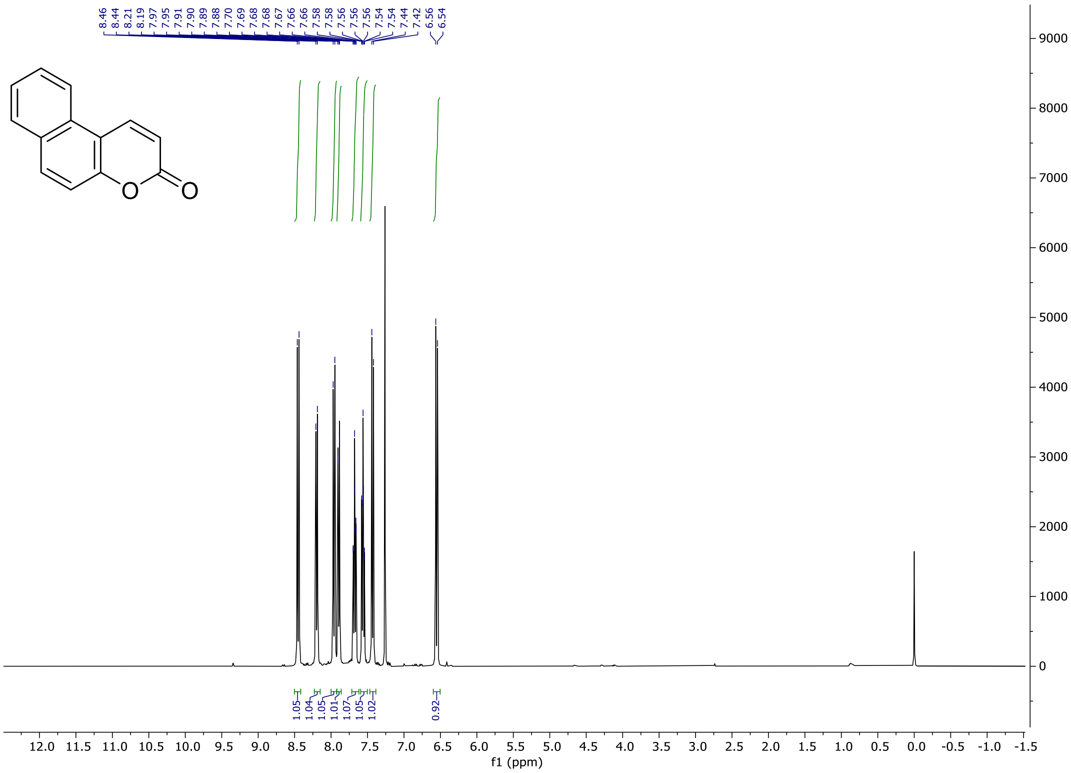

^{1}H NMR in $CDCl_3$.

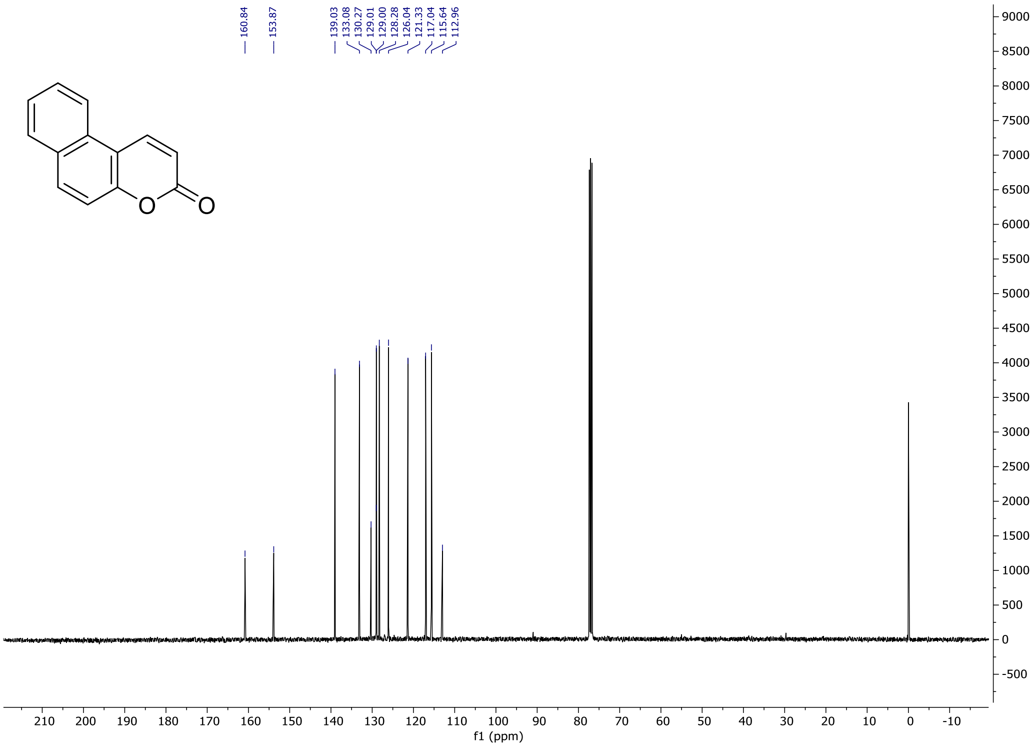

^{13}C NMR in $CDCl_3$.

2-Phenyl-4H-chromen-4-one (7a)

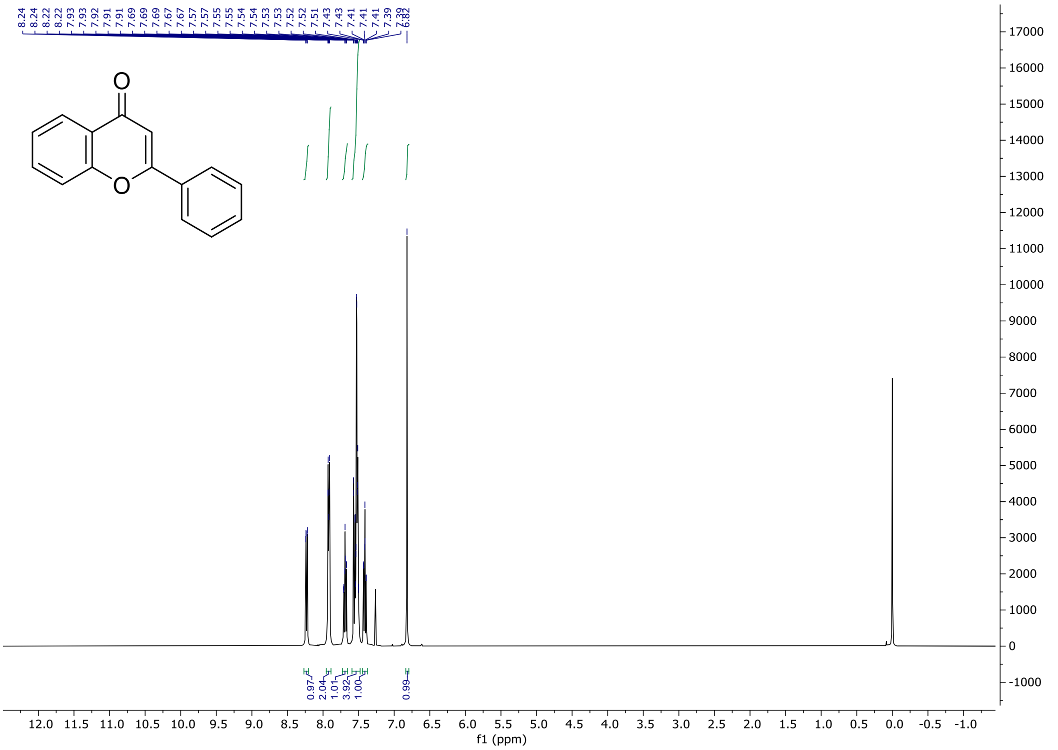

^{1}H NMR in $CDCl_3$.

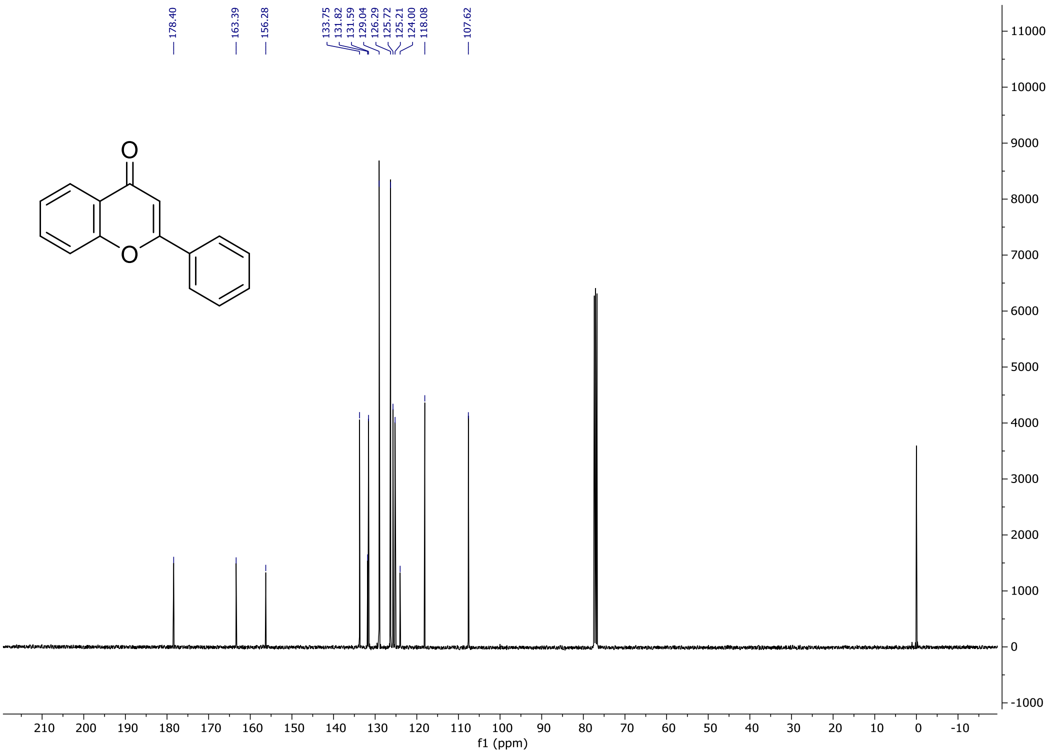

^{13}C NMR in $CDCl_3$.

6-Methoxy-2-phenyl-4*H*-chromen-4-one (7b)

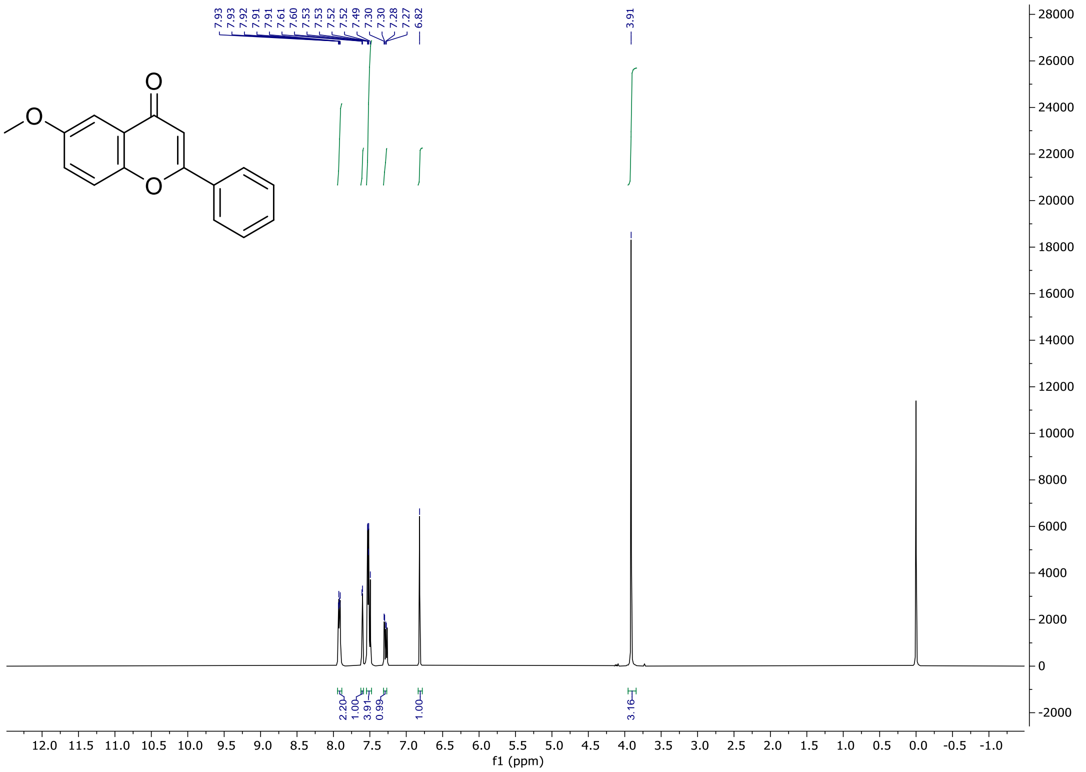

^{1}H NMR in $CDCl_3$.

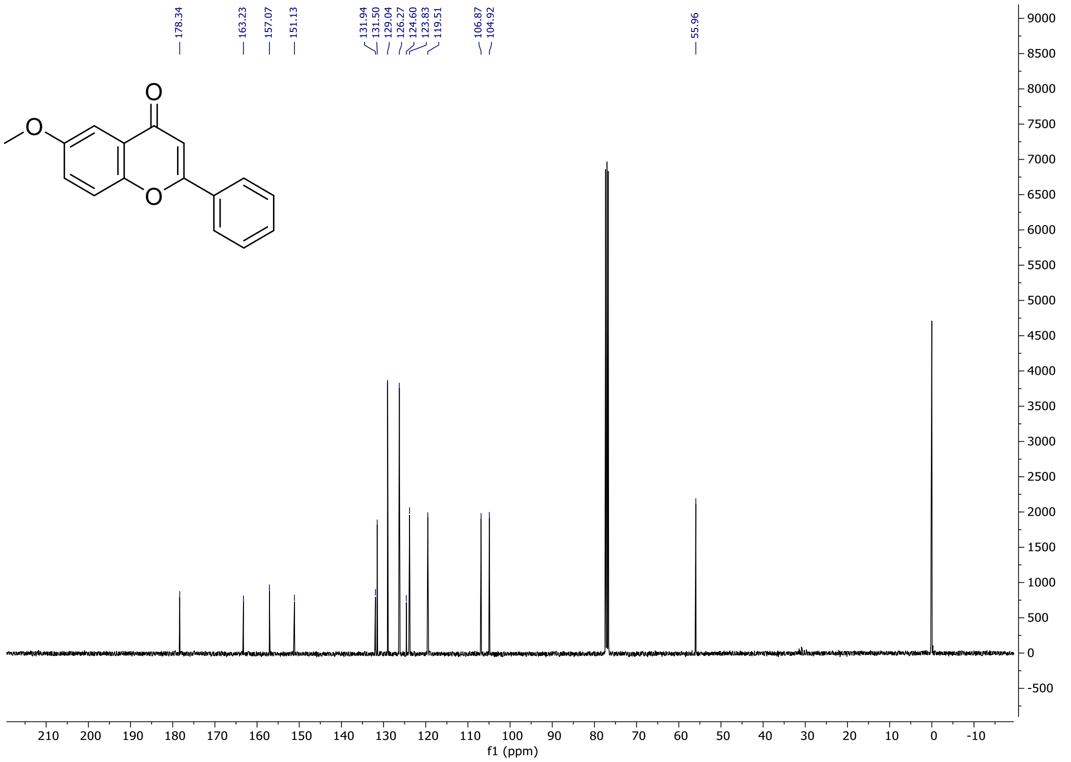

^{13}C NMR in $CDCl_3$.

7-Methoxy-2-phenyl-4*H*-chromen-4-one (7c)

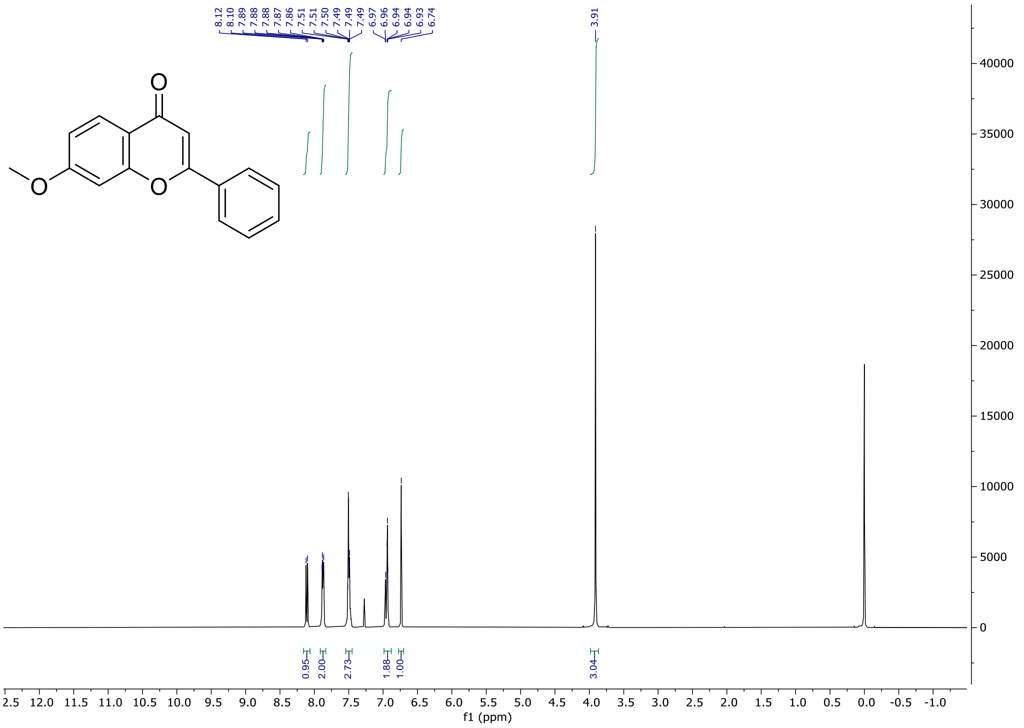

^{1}H NMR in $CDCl_3$.

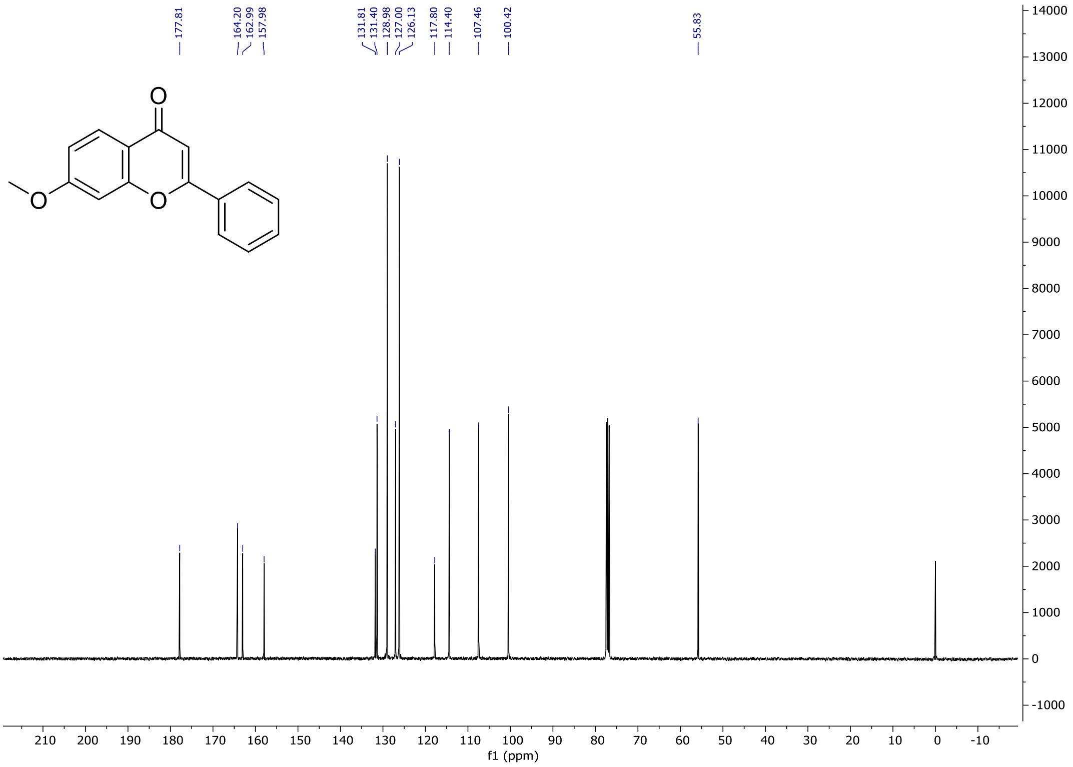

^{13}C NMR in $CDCl_3$.

4-Oxo-2-phenyl-4*H*-chromen-7-yl benzoate (7d)

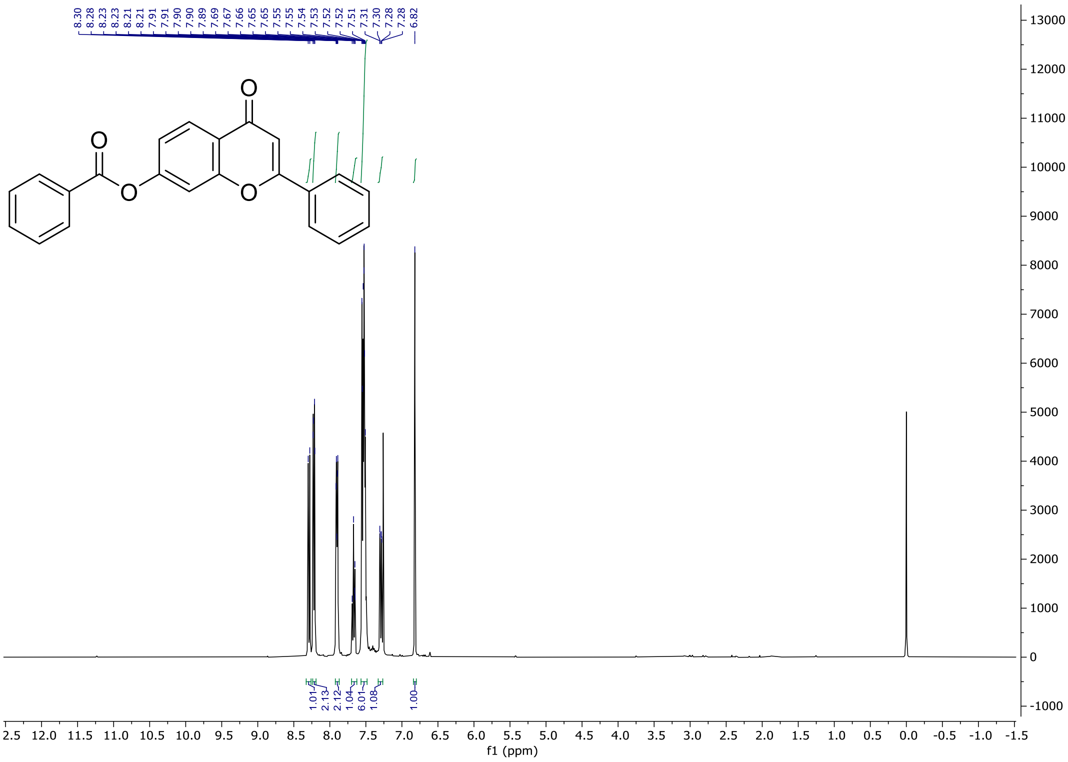

^{1}H NMR in $CDCl_3$.

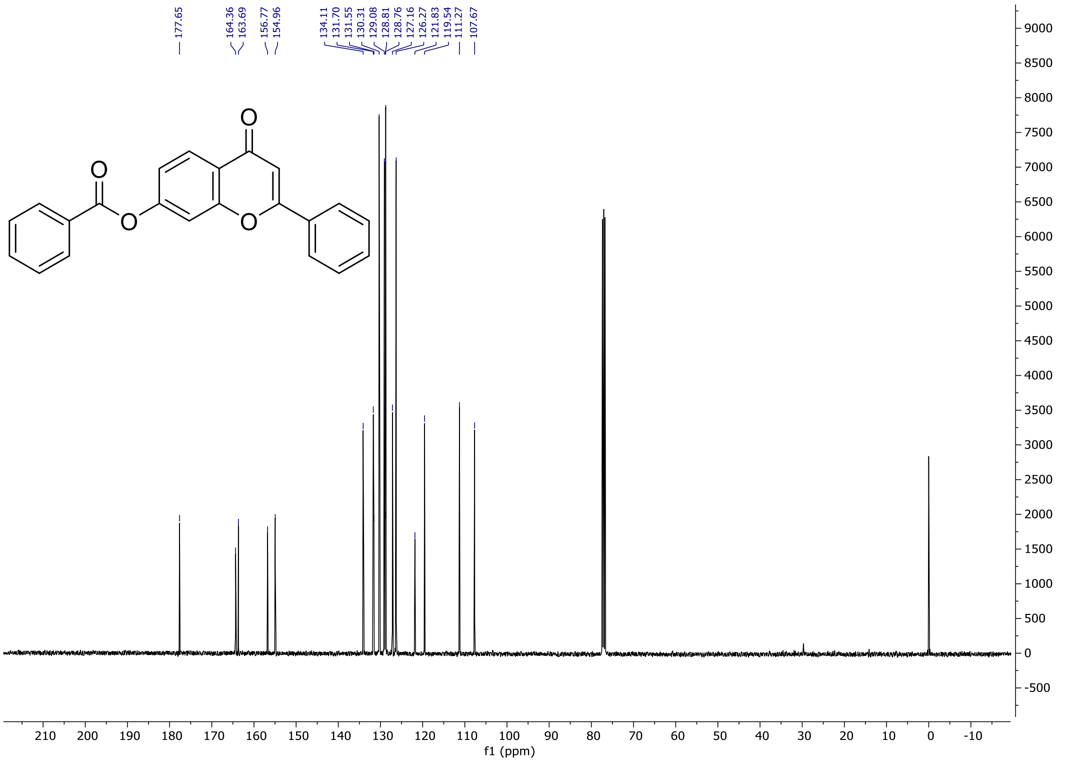

^{13}C NMR in $CDCl_3$.

10. References

[1] M. J. Frisch, Trucks, G.W., Schlegel, H.B., Scuseria, G.E., Robb, M.A., Cheeseman, J.R.; Scalmani, G.; Barone, V.; Petersson, G.A.; Nakatsuji, H.; Li, X.; Caricato, M.; Marenich, A.V.; Bloino, J., Janesko, B.G., Gomperts, R., Mennucci, B., Hratchian, H.P., Ortiz, J.V., Izmaylov, A.F., Sonnenberg, J.L., Williams-Young, D., Ding, F., Lipparini, F., Egidi, F., Goings, J., Peng, B., Petrone, A., Henderson, T., Ranasinghe, D., Zakrzewski, V.G., Gao, J., Rega, N., Zheng, G., Liang, W., Hada, M., Ehara, M., Toyota, K., Fukuda, R., Hasegawa, J., Ishida, M., Nakajima, T., Honda, Y., Kitao, O., Nakai, H., Vreven, T., Throssell, K., Montgomery Jr., J.A., Peralta, J.E., Ogliaro, F., Bearpark, M.J., Heyd, J.J., Brothers, E.N., Kudin, K.N., Staroverov, V.N., Keith, T.A., Kobayashi, R., Normand, J., Raghavachari, K., Rendell, A.P., Burant, J.C., Iyengar, S.S., Tomasi, J., Cossi, M., Millam, J.M., Klene, M., Adamo, C., Cammi, R., Ochterski, J.W., Martin, R.L., Morokuma, K., Farkas, O., Foresman, J.B., Fox, D.J. Gaussian,, *Gaussian 16 Rev. C.01, Wallingford, CT* **2016**.

[2] A. D. Becke, *The Journal of Chemical Physics* **1993**, *98*, 5648-5652.

[3] S. Grimme, S. Ehrlich, L. Goerigk, *J. Comput. Chem.* **2011**, *32*, 1456-1465.

[4] F. Weigend, R. Ahlrichs, *Phys. Chem. Chem. Phys.* **2005**, *7*, 3297-3305.

[5] A. V. Marenich, C. J. Cramer, D. G. Truhlar, *J. Phys. Chem. B* **2009**, *113*, 6378-6396.

[6] S. Grimme, *Chem. Eur. J.* **2012**, *18*, 9955-9964.

[7] P. Pracht, E. Caldeweyher, S. Ehlert, S. Grimme, *ChemRxiv*.

[8] S. Hoops, S. Sahle, R. Gauges, C. Lee, J. Pahle, N. Simus, M. Singhal, L. Xu, P. Mendes, U. Kummer, *Bioinformatics* **2006**, *22*, 3067-3074.

[9] R. A. Angnes, mechaSVG, *GitHub repository* **2020**.

[10] D. R. Chisholm, G.-L. Zhou, E. Pohl, R. Valentine, A. Whiting, *Beilstein J. Org. Chem.* **2016**, *12*, 1851-1862.

[11] L. Wu, Y. Hao, Y. Liu, Q. Wang, *Org. Biomol. Chem.* **2019**, *17*, 6762-6770.

[12] L. Wu, H. Zhou, L. Sun, J. Cui, W. Liu, Y. Wang, L. Xie, *Russ. J. Org. Chem.* **2024**, *60*, 459-466.

[13] J. C. Bieniek, B. Mashtakov, D. Schollmeyer, S. R. Waldvogel, *Chem. Eur. J.* **2024**, *30*, e202303388.

[14] C.-C. Tzeng, I.-L. Chen, Y.-L. Chen, T.-C. Wang, Y.-L. Chang, C.-M. Teng, *Helv. Chim. Acta* **2000**, *83*, 349-358.

[15] M. Kawasaki, Y. Asano, K. Katayama, A. Inoue, C. Hiraoka, H. Kakuda, A. Tanaka, M. Goto, N. Toyooka, T. Kometani, *J. Mol. Catal. B: Enzym.* **2008**, *54*, 93-102.

[16] Y. Chen, A. Turlik, T. R. Newhouse, *J. Am. Chem. Soc.* **2016**, *138*, 1166-1169.

[17] K. M. Gardinier; M.P. Healy; K. Jendza; Y. Pan; K. Y. Wang; F. Yang, Novel Cyclopental[C]pyrrol Negative Allosteric Modulators of NR2B. U.S. Patent WO 2022204336A1, September 29, 2022. (https://worldwide.espacenet.com/patent/search/family/083396075/publication/WO2022204336A1?q=WO2022204336A1).
